# Rhodium Indenyl NHC and Fluorenyl‐Tethered NHC Half‐Sandwich Complexes: Synthesis, Structures and Applications in the Catalytic C−H Borylation of Arenes and Alkanes

**DOI:** 10.1002/chem.202102961

**Published:** 2021-11-18

**Authors:** Kieren J. Evans, Paul A. Morton, Christian Luz, Callum Miller, Olivia Raine, Jason M. Lynam, Stephen M. Mansell

**Affiliations:** ^1^ Institute of Chemical Sciences Heriot-Watt University Edinburgh EH14 4AS UK; ^2^ Department of Chemistry University of York Heslington, York YO10 5DD UK

**Keywords:** C−H activation, C−H borylation, Rh N-heterocyclic carbene catalysts, rhodium indenyl, tethered NHC

## Abstract

Indenyl (Ind) rhodium N‐heterocyclic carbene (NHC) complexes [Rh(*η* ^5^‐Ind)(NHC)(L)] were synthesised for 1,3‐bis(2,6‐diisopropylphenyl)‐4,5‐dihydroimidazol‐2‐ylidene (SIPr) with L=C_2_H_4_ (**1**), CO (**2 a**) and cyclooctene (COE; **3**), for 1,3‐bis(2,4,6‐trimethylphenyl)‐4,5‐dihydroimidazol‐2‐ylidene (SIMes) with L=CO (**2 b**) and COE (**4**), and 1,3‐bis(2,4,6‐trimethylphenyl)imidazol‐2‐ylidene (IMes) with L=CO (**2 c**) and COE (**5**). Reaction of SIPr with [Rh(Cp*)(C_2_H_4_)_2_] did not give the desired SIPr complex, thus demonstrating the “indenyl effect” in the synthesis of **1**. Oxidative addition of HSi(OEt)_3_ to **3** proceeded under mild conditions to give the Rh silyl hydride complex [Rh(Ind){Si(OEt)_3_}(H)(SIPr)] (**6**) with loss of COE. Tethered‐fluorenyl NHC rhodium complexes [Rh{(*η* ^5^‐C_13_H_8_)C_2_H_4_N(C)C_2_H_*x*_NR}(L)] (*x*=4, R=Dipp, L=C_2_H_4_: **11**; L=COE: **12**; L=CO: **13**; R=Mes, L=COE: **14**; L=CO: **15**; *x*=2, R=Me, L=COE: **16**; L=CO: **17**) were synthesised in low yields (5–31 %) in comparison to good yields for the monodentate complexes (49–79 %). Compounds **3** and **1**, which contain labile alkene ligands, were successful catalysts for the catalytic borylation of benzene with B_2_pin_2_ (Bpin=pinacolboronate, 97 and 93 % PhBpin respectively with 5 mol % catalyst, 24 h, 80 °C), with SIPr giving a more active catalyst than SIMes or IMes. Fluorenyl‐tethered NHC complexes were much less active as borylation catalysts, and the carbonyl complexes were inactive. The borylation of toluene, biphenyl, anisole and diphenyl ether proceeded to give *meta* substitutions as the major product, with smaller amounts of *para* substitution and almost no *ortho* product. The borylation of octane and decane with B_2_pin_2_ at 120 and 140 °C, respectively, was monitored by ^11^B NMR spectroscopy, which showed high conversions into octyl and decylBpin over 4–7 days, thus demonstrating catalysed sp^3^ C−H borylation with new piano stool rhodium indenyl complexes. Irradiation of the monodentate complexes with 400 or 420 nm light confirmed the ready dissociation of C_2_H_4_ and COE ligands, whereas CO complexes were inert. Evidence for C−H bond activation in the alkyl groups of the NHC ligands was obtained.

## Graphical abstract

**Indenyl rhodium** NHC complexes with labile alkene ligands (ethene and cyclooctene) have been synthesized and shown to be better catalysts than the related fluorenyl‐tethered NHC rhodium complexes for the C−H activation and borylation of arenes and alkanes.

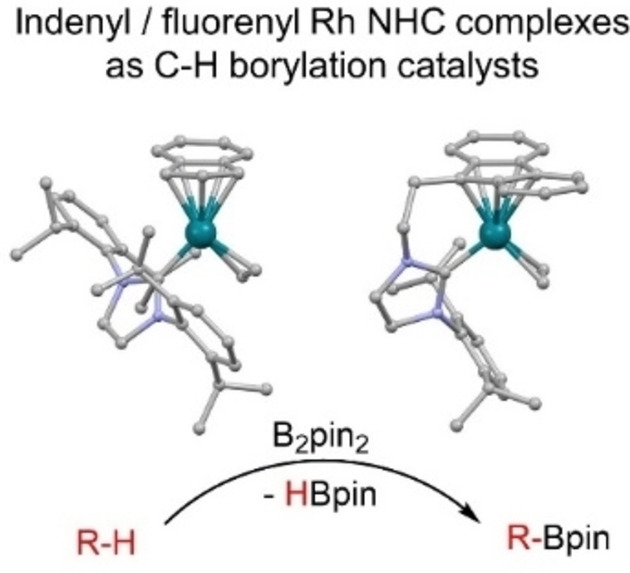

## Introduction

C−H activation is typically defined as the weakening or cleavage of a C−H bond at a metal centre.^[1]^ When followed by a functionalisation step, a new carbon‐element bond forms from a relatively inert C−H bond.^[2]^ The development of C−H activation has been particularly important in enabling the functionalisation of otherwise inert aryl and alkyl C−H bonds.[^2a^, ^2b^, ^3^] Although much progress has been made with “directed” C−H activation, where an additional group or ligand on the substrate helps enable the reaction,[^2c^, ^4^] “undirected” C−H bond activation still remains a challenge.[^1e^, ^1f^, ^2e^, ^5^] This includes achieving the high reactivity required to functionalise strong, nonpolar C−H bonds, while still controlling the selectivity in order to give the desired product. As always, increasing catalyst lifetimes and activities are key goals. Pt catalysts in combination with stoichiometric oxidants were amongst the first to demonstrate the successful functionalisation of alkanes,^[1b]^ with much research in this area still on‐going.^[6]^

**Scheme 1:**
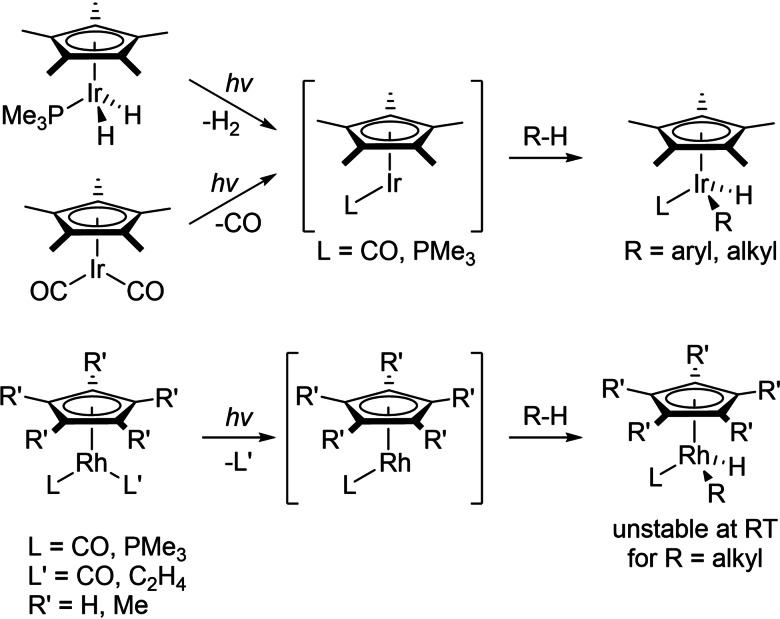
Stoichiometric CH activation with Group 9 complexes.

In 1982, ground‐breaking work by Bergman, Graham and co‐workers showed that 16 electron [Ir(Cp*)(L)] (L=PMe_3_, CO) fragments generated from photochemical loss of H_2_ or CO from suitable precursors could oxidatively add alkane and arene C−H bonds (Scheme 1).^[7]^ This reactivity was exciting because it had the potential to integrate with traditional mechanisms in organometallic chemistry that involve oxidative addition, reductive elimination and insertion steps, etc.^[8]^ Much work has since been performed in order to understand this reactivity,^[1a]^ encompassing Cp, Cp* and Tp (tris(pyrazolyI)borate) group 9 complexes that undergo these processes.^[9]^

**Scheme 2:**
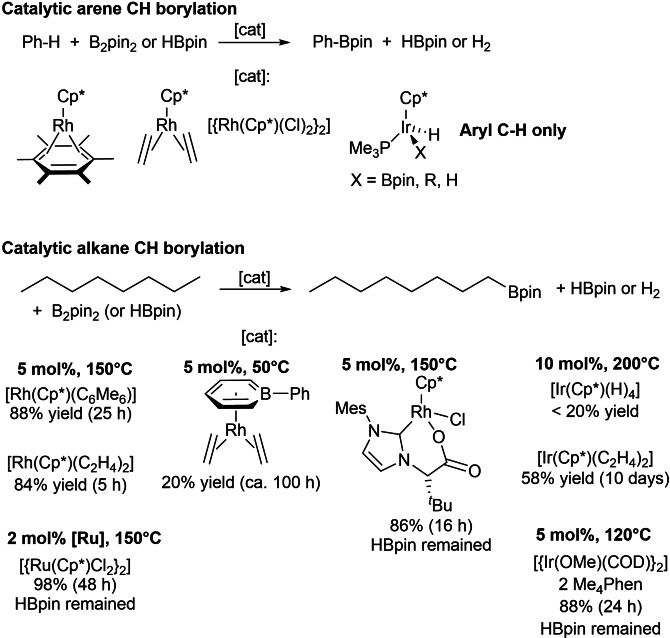
Catalytic arene and alkane CH borylation with selected Group 9 complexes.

Borylation has proven to be an extremely important C−H functionalisation route.^[10]^ The organoboron species produced are very useful for further conversion,^[11]^ as well as being important products in their own right as drug molecules^[12]^ (e. g., Bortezomib^[13]^ and Vaborbactam^[14]^). Initially, stoichiometric studies of Fe, Ru and W boryl complexes with Cp* ligands demonstrated photochemical reactivity with arenes and alkanes to form organoboranes.^[15]^ This work was subsequently extended to a [Re(Cp*)(CO)_3_] precatalyst,^[16]^ and then thermally to Rh and Ir precatalysts, also with Cp* ligands, which catalysed the C−H borylation of alkanes and arenes (Scheme 2).[^10d^, ^17^] Further investigation showed that alkane activation was mediated by [Rh(Cp*)(Bpin)_2_(H)_2_] and [Rh(Cp*)(Bpin)_3_(H)] intermediates (pin=1,2‐O_2_C_2_Me_4_),^[18]^ which can also be considered to be Rh^III^ complexes with elongated σ‐borane ligands.[^18^, ^19^] These catalytic C−H borylation conditions can tolerate heteroatoms including ether and amine substrates.^[20]^ Other catalysts have also been used, such as the Rh phosphine complex [RhCl(P^*i*^Pr_3_)_3_(N_2_)], which is an effective precatalyst for the borylation of benzene and aryl‐methyl groups.^[21]^ A Rh species containing a boratabenzene ligand (anionic, like Cp) also showed reactivity with B_2_Pin_2_ and octane, but deactivated much more quickly than [Rh(Cp*)(C_2_H_4_)_2_].^[22]^ Cp*Rh catalysts with carboxylate‐tethered NHC coligands have also been developed for directed *ortho* C−H borylation, with the borylation of octane with B_2_Pin_2_ also possible at 150 °C with 5 mol % catalyst.^[23]^ Subsequently, [Ru(Cp*)] complexes^[24]^ and Ir/3,4,7,8‐tetramethylphenanthroline complexes were also shown to catalytically borylate alkanes.^[25]^ Even methane has been shown to be borylated using some of the above‐mentioned Rh and Ir complexes.^[26]^ A tandem dehydrogenation/hydroboration of alkanes is also possible, but high temperatures (200 °C) and a sacrificial alkene were required.^[27]^ Highly active Ir systems are now favoured for arene borylation,[^10c^, ^28^] and the borylation of benzylic positions is also effectively accomplished using Ir catalysis,^[29]^ although Co catalysts based on diamine or NHC ligands have been described recently.^[30]^ However, the efficient borylation of alkanes is still an important challenge, with the focus of research still primarily on the development of Rh complexes as catalysts, although highly active Ir catalysts based on 2,2′‐dipyridylarylmethane ligands have been described recently.^[31]^

Indenyl (Ind) has previously been used in Ir‐mediated C−H activation,^[32]^ anticipating the potential enhanced reactivity from the haptotropic flexibility induced by the “indenyl effect”.^[33]^ [Ir(Ind)(PMe_3_)(R)(R′)] (R and R′=alkyl, aryl, hydride) complexes were significantly more reactive than the Cp* analogues, undergoing thermolysis reactions with the arene/alkane solvent at lower temperatures, coordination of L‐type ligands to form octahedral *η* ^1^‐indenyl complexes and insertion of R (R=alkyl, aryl) into CO, or H into alkynes or ethylene. Heptamethylindenylrhodium dichloride was found to be an improved catalyst for the diastereoselective coupling of O‐substituted arylhydroxamates and cyclopropenes compared to the Cp* analogue.^[34]^ Westcott, Marder and co‐workers have used Rh indenyl complexes in hydroboration catalysis,^[35]^ which also gave products from the dehydrogenative borylation of alkenes, and also demonstrated that [Ir(Ind)(COD)] was a precursor to [Ir(*η* ^6^‐arene)(Bcat)_3_].^[36]^ The analogous pinacolateboryl complex [Ir(*η* ^6^‐arene)(Bpin)_3_], in combination with phosphine ligands such as PMe_3_, Me_2_PCH_2_CH_2_PMe_2_ and Ph_2_PCH_2_CH_2_PPh_2_, functioned as an arene C−H borylation catalyst in reactions of HBpin at high temperatures (100–150 °C).^[28b]^ Thus, a diversity of reactivity is already evident for indenyl, including the relative importance of *η* ^1^, *η* ^3^ and *η* ^5^ coordination modes as well as potential loss of the indenyl ligand.

With recent evidence for the utility of NHC ligands in CH activation growing,[^23^, ^30a^, ^37^] we became interested in the idea of using indenyl and fluorenyl ligands in combination with strongly σ‐donating NHC ligands in order to increase the electron density at the metal centre whilst being able to tune the ligand properties through selecting different NHC ligands. Interestingly, with the propensity of indenyl to “ring slip”, turning it into a three‐electron donor, the combination of an *η* ^3^‐indenyl and NHC ligand set would lead to identical electron counts to the widely successful [M(Cp*)] fragment. Several Rh and Ir complexes with tethered ligands have been developed,^[38]^ including phosphine‐tethered Cp^[39]^ and hemilabile quinolyl‐Cp complexes^[40]^ that have been used in C−H activation. We also wanted to investigate complexes where the NHC is covalently linked to fluorenyl through the use of NHC‐tethered ligands in order to make comparisons with monodentate analogues, searching for possible enhancements in stability (through chelation), reactivity (through orbital effects from the constraint of the geometry)^[41]^ or selectivity (cf. constrained geometry catalysts used in the (co)polymerisation of alpha‐olefins)^[42]^ that could be applied to C−H activation. In this work, we describe the synthesis of indenyl Rh NHC and fluorenyl‐tethered NHC Rh complexes, their use in the catalytic C−H borylation of alkanes and arenes as well as describe their photochemistry and stoichiometric reactivity.

## Results and Discussion

### Synthesis of monodentate NHC complexes

**Scheme 3:**
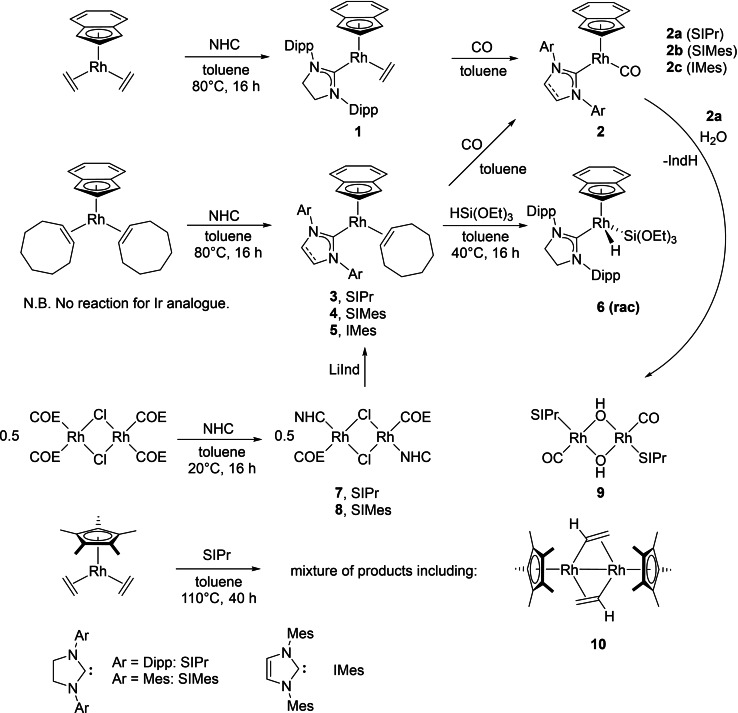
Synthesis and reactivity of rhodium complexes with monodentate NHC ligands.

[Rh(Ind)(alkene)_2_] complexes (alkene=ethylene^[43]^ and cyclooctene^[35]^) were used in reactions with free NHCs to produce the desired half‐sandwich complexes (Scheme 3). The reaction of [Rh(Ind)(alkene)_2_] with SIPr at 80 °C in toluene for 16 h gave [Rh(Ind)(SIPr)(alkene)] in good yields (alkene=C_2_H_4_, 63 % (**1**); alkene=COE, 70 % (**3**)). Reaction with CO was rapid and gave the mono carbonyl complex **2 a** (79 %). Due to higher yields of [Rh(Ind)(COE)_2_] and comparable catalysis results between C_2_H_4_ and COE complexes, only [Rh(Ind)(COE)] complexes of IMes and SIMes were synthesised. Using IMes, **5** was synthesised in reasonable yield (49 %) whereas reactions with SIMes produced by‐products, hampering purification. Thus, we investigated a complementary synthetic route by first reacting SIMes and SIPr with [{Rh(*μ*‐Cl)(COE)_2_}_2_] that reacts in both cases with loss of cyclooctene instead of loss of the bridging Cl motif, as seen previously for IPr and IMes with [{Rh(*μ*‐Cl)(COE)_2_}_2_].^[44]^ In contrast, the reaction of IMes with [{Rh(*μ*‐Cl)(C_2_H_4_)_2_}_2_] gave monomeric [Rh(Cl)(IMes)(C_2_H_4_)_2_].^[45]^ The dimeric complexes **7** and **8** were formed in good yields (74 and 63 % respectively). Addition of LiInd gave the desired half‐sandwich complexes for SIPr and SIMes (60 %). In order to compare the rates of ethylene substitution with SIPr for Ind and Cp* Rh(C_2_H_4_)_2_ complexes, the reaction of [Rh(Cp*)(C_2_H_4_)_2_]^[46]^ with SIPr was carried out at 110 °C for 40 h (analogous to the reported reaction for IMes),^[45]^ but this did not give the desired SIPr complex. Instead, only partial consumption of the starting materials was evident along with the generation of a complex mixture of products. This demonstrated that [Rh(Ind)(C_2_H_4_)_2_] reacts much more readily with SIPr than the Cp* analogue, a manifestation of the “indenyl effect” where auxiliary ligand substitution is accelerated as a result of *η* ^3^ coordination of the indenyl ligand driven by recovery of benzene ring aromaticity.^[33a]^ A single crystal X‐ray diffraction study identified **10** as one of products from the reaction and provided evidence for one of the pathways involving loss of one ethylene ligand, C−H activation of the other and loss of two H atoms to generate a Rh−Rh bonded dimer with bridging σ,π‐vinyl ligands (see the Supporting Information for structure). This structure is very similar to products formed in the reaction of [Rh(Ind)(C_2_H_4_)_2_] or [Rh(1‐MeInd)(C_2_H_4_)_2_] with Me−C≡C−Me, which gave a Rh dimer containing one bridging vinyl and one bridging 1,2‐dimethylvinyl ligand.^[47]^ The C−H activation of ethylene by [Ir(Cp*)(PR_3_)] fragments^[48]^ has been the subject of an extensive computational study.^[49]^

In order to study the differences between Ir and Rh, reactions between [Ir(Ind)(alkene)_2_] (alkene=COE, C_2_H_4_)[^32a^, ^50^] and SIPr were attempted, but led to no reaction at 80 °C with decomposition evident at 110 °C. A single crystal X‐ray diffraction study on [Ir(Ind)(COE)_2_] (see the Supporting Information) revealed C=C bond lengths for the COE ligands of 1.426(3) and 1.435(3) Å, longer than those seen in [Rh(Ind)(COE)_2_] (1.405(3) and 1.408(3) Å),^[35]^ representing greater π‐backbonding and a stronger Ir–COE bond, which is likely to be inhibiting the reaction.

Two additional reactions were carried out. Addition of water to [Rh(Ind)(SIPr)(CO)] generated the hydroxide‐bridged dimer **9** (see the Supporting Information for the structure) with loss of indene, identified by ^1^H NMR spectroscopy, demonstrating that Rh indenyl complexes are not water tolerant. Addition of triethoxysilane to [Rh(Ind)(SIPr)(COE)] (40 °C, 16 h) generated the Rh silyl hydride **6** in 67 % yield, thus demonstrating facile oxidative addition. In comparison, [Rh(Cp)(Si^*i*^Pr_3_)(H)(PR_3_)] (R=Me^[51]^ and Ph^[52]^) were synthesised by photolytic ejection of ethylene[^51^, ^52^] or *η* ^2^‐C_6_F_6_ ligands.^[53]^ [Rh(C_5_R_5_)(SiR_3_)_2_(H)_2_] complexes can be formed thermally,^[54]^ whilst *trans*‐[Rh(Cp*)(SiEt_3_)(H)(Bpin)(H)] was formed from the reaction of HBpin with [Rh(Cp*)(SiEt_3_)_2_(H)_2_].^[55]^

Characterisation of **1**–**5** by multinuclear NMR spectroscopy showed resonances for the indenyl and NHC ligands as expected, with the carbenic ^13^C resonance a doublet at 211–216 ppm (^1^ *J*_Rh−C_ ca. 70 Hz) for saturated NHCs, and at lower chemical shift for IMes. IR spectroscopy showed a clear CO stretch for the carbonyl complexes; 1944 cm^−1^ for **2 a**, 1939 cm^−1^ for **2 b** and 1938 cm^−1^ for **2 c**. Mass spectrometry revealed [*M*+H]^+^ ions for the carbonyl complexes **2 b** and **2 c**, with the alkene complexes notably failing to give molecular ion peaks. Interestingly, [*M*−L−H]^+^ ions were observed for **3**, and as fragments for the carbonyl complexes, pointing towards loss of the labile ligand and potential cyclometallation of the NHC.

**Figure 1:**
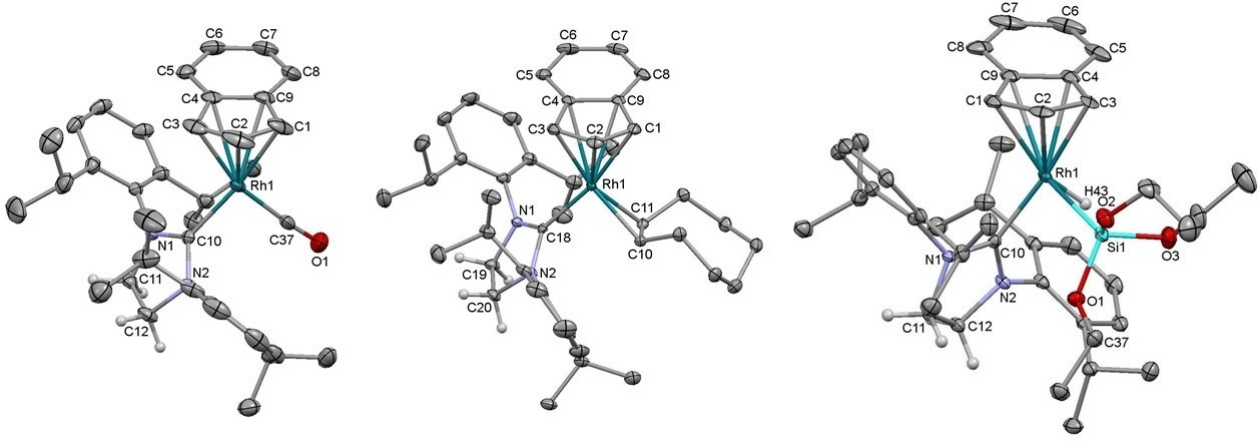
Molecular structures of **2 a** (left), **3** (middle) and **6** (right). Thermal ellipsoids are at 50 % probability, and all H atoms, except on the SIPr backbone and the Rh−H, have been removed for clarity. See the Supporting Information for additional data.

Single crystal X‐ray diffraction studies were used to characterise all of the indenyl Rh(I) half‐sandwich complexes **1**–**5**. Their molecular structures show indenyl *η* ^5^‐bound to Rh with terminal coordination of the NHC and either *η* ^2^ binding of the alkene ligand or *η* ^1^ coordination of CO (Figure 1). All of the five‐membered indenyl rings show a fold distortion^[56]^ of between 8° and 11° for C2 (i. e., the dihedral angle between the plane containing C1, C2 and C3 and the plane containing C1, C3, C4 and C9). This is similar to the values observed for the [Rh(Ind)(alkene)_2_] starting materials (between 8.6 and 9.3°).[^36^, ^57^] By looking at the individual values for the Rh−C_Ind_ bond lengths, which invariably have two longer bond lengths (those to the two benzannulated carbons), the indenyl rings can be described as falling between a coordination geometry with two very short, one intermediate, and two very long Rh−C interactions (e. g., [Rh(Ind)(SIPr)(COE)]), to geometries with three short and similar Rh−C and two long Rh−C interactions (e. g., [Rh(Ind)(SIPr)(CO)], see the Supporting Information for comparison). The Rh−NHC bond lengths are around 1.99 Å for SIPr and SIMes, and 2.0169(10) or 2.0124(9) Å for IMes. The coordinated COE C=C bond lengths are significantly lengthened (1.41–1.42 Å) from free COE (calcd: 1.331 Å using B3LYP/cc‐pVTZ),^[58]^ but identical within experimental uncertainty to those in [Rh(Ind)(COE)_2_]. This is similar for the ethylene complex **1** (1.399(2) Å) and [Rh(Ind)(C_2_H_4_)_2_] (average C=C: 1.377 [12] Å).^[57]^

^1^H NMR spectroscopic data for the silyl hydride complex **6** showed a doublet at −15.07 ppm with ^1^ *J*_RhH_=30.6 Hz and satellite peaks from coupling to ^29^Si (^2^ *J*_SiH_=14.8 Hz), similar to literature Rh silyl hydride complexes,[^51^, ^59^] for example, [RhCp(Si^*i*^Pr_3_)_2_(H)_2_] (−14.85 ppm, ^1^ *J*_RhH_=31.6 Hz, ^2^ *J*_SiH_=15.6 Hz)^[60]^ whereas [Rh(Cp)(Si^*i*^Pr_3_)(H)(PMe_3_)] showed ^2^ *J*_SiH_<2 Hz.^[51]^ The ^29^Si{^1^H} NMR spectrum showed a doublet at −13.77 ppm (^1^ *J*_Rh−Si_=67.3 Hz).

X‐ray diffraction experiments revealed the anticipated piano stool geometry of **6**, with the hydride located and refined (Figure 1). The indenyl coordination in **6** displayed two short, one intermediate and two longer Rh−C interactions, with a fold distortion of 8.8°. The Rh−Si bond length (2.2691(8) Å) is shorter than for [Rh(Cp)(Si^*i*^Pr_3_)(H)(PMe_3_)] (2.3617(3) Å)^[51]^ and [Rh(Cp)(Si^*i*^Pr_3_)(H)(PPh_3_)] (2.386(2) Å),^[52]^ however, the Rh−H distances (1.45(3) Å in **6**) were indistinguishable within experimental uncertainty. The Si1⋅⋅⋅H43 distance (2.239 Å) as well as the unequal H−Rh−C_carbene_ and H−Rh−Si angles suggest some residual H⋅⋅⋅Si interaction,^[61]^ as was concluded for [Rh(Cp)(Si^*i*^Pr_3_)(H)(PMe_3_)] (H⋅⋅⋅Si=2.278(17) Å).^[51]^ The Rh−NHC distance (2.014(3) Å) is marginally longer than in **1** (1.9886(12) Å) or **3** (1.9881(15) and 1.9946(15) Å). Comparisons between the indenyl complexes and the Cl bridged dimers **7** and **8** (see the Supporting Information) show slightly shorter Rh−NHC distances (1.9504(12) and 1.9572(15) Å respectively) for the dimers, but similar C=C bond distances (1.416(2) and 1.4071(18) Å respectively). The hydroxide bridged dimer **9** features a similar Rh−NHC bond length (1.961(2) Å) but with a closer Rh⋅⋅⋅Rh separation (3.24 Å compared to 3.73 Å in **7**) due to shorter Rh−O bond distances compared to Rh−Cl.

### Synthesis and characterisation of tethered complexes

**Scheme 4:**
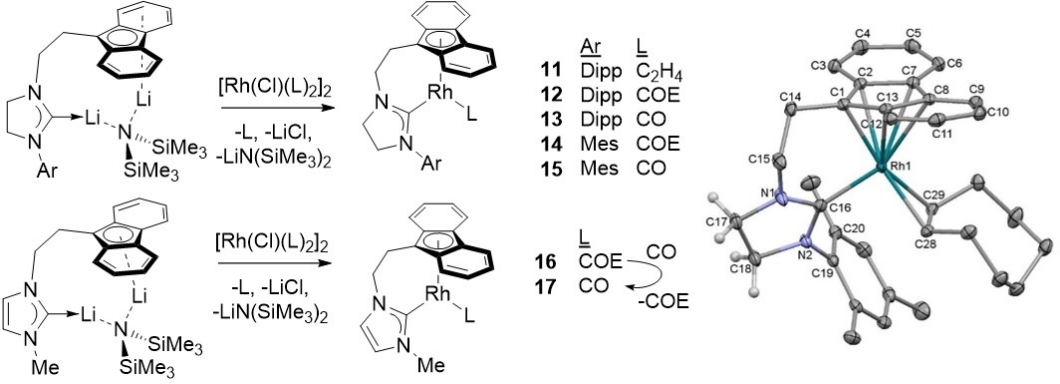
Left: Synthesis of fluorenyl‐tethered NHC rhodium complexes. Right: Molecular structure of **14**. Thermal ellipsoids are at 50 % probability, and all H atoms except for those on the NHC backbone have been removed for clarity. See the Supporting Information for additional data.

Fluorenyl‐tethered NHC complexes are an attractive target for three reasons: i) fluorenyl ligands show similar, or better, enhancements in reactivity compared to indenyl ligands,^[62]^ ii) tethering fluorenyl to an NHC enforces a geometric constraint on the complex that can change the energies of the frontier MOs,^[41]^ and iii) stability of the catalyst could be enhanced through the chelate effect. Fluorenyl‐tethered NHC Rh complexes were synthesised from the LiN(SiMe_3_)_2_ adducts recently reported (Scheme 4).^[63]^ The dimeric LiN(SiMe_3_)_2_‐free lithium salt^[63a]^ [Li{*μ*‐(*μ*‐*η* ^1^ : *η* ^1^‐(*η* ^5^‐C_13_H_8_)C_2_H_4_N(C)C_2_H_2_NMe}]_2_ was not a successful ligand transfer reagent and our progress towards indenyl‐tethered ligands is currently limited by their challenging synthesis.^[64]^ Despite many attempts, and the use of different conditions, yields for the tethered complexes (5–31 %) are much lower than the monodentate analogues, reminiscent of the low yields seen for the “fly‐trap” method of synthesising strained metallocenophanes.^[65]^ N substituents included Dipp, Mes and Me, with Me substitution selected to discourage intramolecular C−H activation (a 4‐membered chelate would result). Characterisation by ^1^H and ^13^C{^1^H} NMR spectroscopy revealed the presence of distinctive fluorenyl resonances in the aromatic region, separate resonances for the four methylene groups and the carbenic carbons as doublets between 211 (**12**) and 214 pm (**13**) for the saturated NHCs, and at 180 ppm for the unsaturated NHCs (**16** and **17**). The ^1^ *J*_C‐Rh_ coupling was between 78 and 84 Hz. IR spectroscopy showed carbonyl stretches at 1948 (**13**), 1965 (**15**) and 1959 cm^−1^ (**17**). For **13**, this was very similar to the monodentate SIPr complex (1944 cm^−1^ for **2 a**) but **15** was very different (cf. 1939 cm^−1^ for **2 b**). Mass spectrometry analysis of **13** and **15** revealed [*M*+H]^+^ ions together with [*M*−CO−H]^+^ ions, similar to the monodentate carbonyl complexes. Danopoulos and co‐workers studied the coordination chemistry of fluorenyl‐ and indenyl‐tethered unsaturated NHCs with Rh and Ir precursors.^[38b]^ Although they could generate the tethered 4,7‐dimethylindenyl piano‐stool carbonyl complex [Rh{κ: *η* ^1^,*η* ^5^‐(4,7‐Me_2_C_9_H_4_)C_2_H_4_N(C)C_2_H_2_NDipp}(CO)], the tethered‐fluorenyl analogue reacted with [{Rh(*μ*‐Cl)(COD)}_2_] in an unselective manner to give an NHC‐tethered fulvene complex through C−H activation of the ligand.^[38b]^

Compounds **11**, **13**–**15** and **17** were characterised by single crystal X‐ray diffraction studies, although the data for **15** were of low quality and consequently were only used for establishing connectivity. Prior to the initiation of this work there were only two known crystal structures of rhodium complexes with *η* ^5^‐fluorenyl ligands.[^64a^, ^66^] All complexes show approximate *η* ^5^‐binding of the fluorenyl group with the NHC chelating the Rh atom. The final coordination site is occupied with an *η* ^2^‐alkene ligand or CO. For the complexes with alkene ligands, the fluorenyl binding shows the shortest interaction to Rh from C1 where the tether is attached (2.160(3) Å for **11** and 2.1654(15) Å for **14**). The adjacent carbon atoms, C2 and C13, feature longer interactions with Rh (2.292(3)–2.3569(14) Å), and the Rh interactions with the final two, C7 and C8, are marginally longer (2.366(3)–2.4693(15) Å). The CO complexes show slightly longer Rh−C1 distances (2.185(3)–2.195(3) Å) and almost equivalent distances to the other C atoms. The Rh−NHC bond lengths are between 1.967(3) Å (for **11**) and 2.001(3) Å (**17**), and the alkene C=C distances are identical within error to the monodentate analogues.

### Catalytic C−H borylation

**Scheme 5:**
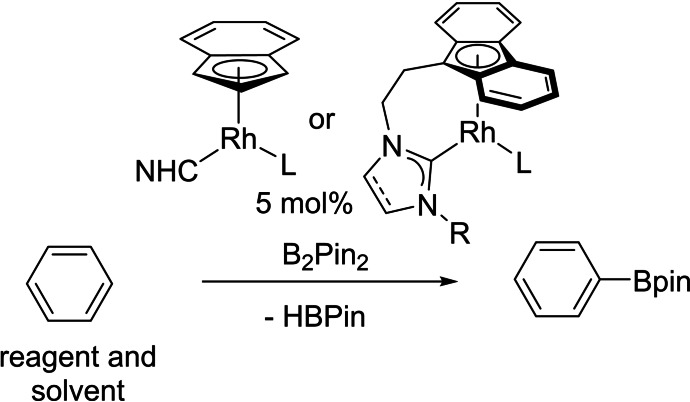
Catalyst screening for the borylation of benzene.

The above complexes were tested as catalysts for the C−H borylation of benzene using B_2_pin_2_ ^[67]^ with benzene in excess as the solvent (Scheme 5, Table 1). This was a good bench‐marking reaction and allowed us to ascertain that the indenyl/NHC and fluorenyl/NHC ligand sets were indeed capable of supporting a catalytic cycle. The two SIPr complexes with alkene ligands were the most active catalysts. The highest yield was achieved using the COE complex **3** (97 % in 24 h at 80 °C), although **1** containing ethylene was similar (93 %). [Rh(Ind)(COE)_2_] was the next most active catalyst (90 % in 40 h), with the IMes and SIMes complexes both showing marginally lower activity (ca. 90 % after 48 h). [RhCp*(C_2_H_4_)_2_], an active catalyst for alkane borylation,^[17a]^ was observed to be a very poor catalyst at 80 °C for benzene borylation yielding only 37 % PhBpin after 44 h. None of the carbonyl complexes showed any signs of catalysis, likely due to the strong Rh−CO bond inhibiting dissociation. Interestingly, tethering the NHC donor to fluorenyl did not enhance catalysis, producing much slower catalysts, although **16** did eventually reach a yield of 87 % after 168 h suggesting slow initiation might be a factor.

**Table 1:** Catalyst (5 mol %) screening for the borylation of benzene with B_2_pin_2_.

Complex	NHC	L	*T* [°C]	*t* [h]	Yield [%]^[a]^
**3**	SIPr	COE	80	24	97
**1**	SIPr	C_2_H_4_	80	24	93
**2**	SIPr	CO	80	48	0
[Rh(Ind)(COE)_2_]	–	COE	80	40	90
[RhCp*(C_2_H_4_)_2_]	–	C_2_H_4_	80	24	11
[RhCp*(C_2_H_4_)_2_]	–	C_2_H_4_	80	44	37
**4**	SIMes	COE	80	24	18
**4**	SIMes	COE	80	48	91
**5**	IMes	COE	80	24	21
**5**	IMes	COE	80	48	90
**11**	R=Dipp	C_2_H_4_	80	100	32
**13**	R=Dipp	CO	80	100	0
**14**	R=Mes	COE	75	100	14
**16**	R=Me unsaturated	COE	75	168	87

[a] Yield determined by ^1^H NMR spectroscopy through integration of the product resonance against ferrocene as an internal standard. Benzene was in excess and B_2_pin_2_ was the limiting reagent.

With the best catalyst system identified, preparative reactions were used to identify the substrate scope of the borylation reaction (Table 2). The substrate was used in excess as the solvent. Isolated yields of PhBpin were high using only 2.5 mol % catalyst (81 %). With 1 mol % a reduced yield of 40 % was achieved.

**Table 2:** Arene and alkane borylation using [Rh(Ind)(SIPr)(COE)].

Arene	*T* [°C], *t* [h], mol % of catalyst	Product	Yield [%]^[a]^	Isomer distribution^[b]^ (*o:m:p*)
benzene	80, 48, 2.5	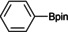	81	–
naphthalene	80, 48, 2.5	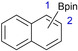	38^[c]^	0.07 (1‐Bpin) : 1 (2‐Bpin)
toluene	110, 48, 2.5	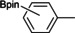	76^[d]^	0.07 : 1.00 : 0.42
mesitylene	150, 72, 2.5	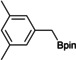	34	–
biphenyl	110, 72, 2.5	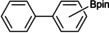	41	0.00 : 1.00 : 0.63
anisole	110, 72, 2.5	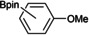	53	0.06 : 1.00 : 0.35
diphenyl ether	110, 72, 2.5	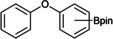	74	0.00 : 1.00 : 0.26
fluorobenzene	80, 48, 2.5	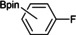	33^[e]^	0.91 : 1 : 0.18
*n*‐octane	130, 48, 5	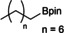	7	terminal only
*n*‐decane	150, 48, 10	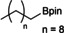	18	terminal only

[a] All yields are isolated yields after column chromatography. The arene or alkane was in excess and was used as the solvent. [b] Determined by ^1^H NMR spectroscopy. [c] 79 % yield by ^1^H NMR spectroscopy. [d] 95 % yield by ^1^H NMR spectroscopy. [e] 95 % yield was observed by NMR spectroscopy, but this compound was not completely stable to the column chromatography conditions.

**Figure 2:**
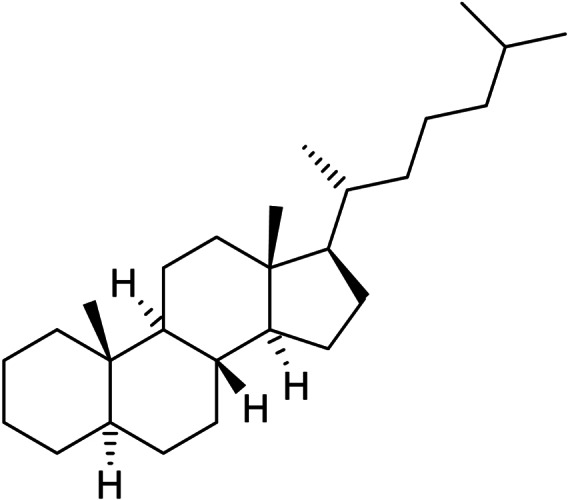
5‐α‐Cholestane.

Mass spectrometry analysis of 3 : 1 and 1 : 1 C_6_H_6_/C_6_D_6_ reaction mixtures confirmed the formation of PhBpin and [D_5_]PhBpin (*m*/*z* 204.1 and 209.2, respectively) without any isotopic scrambling. Borylation of toluene was achieved in 76 % yield, with the mixture of *ortho*, *meta*, *para* isomers (0.07 : 1.0 : 0.42) for tolylBpin similar to [IrCp* (H)(BPin)(PMe_3_)] (0.07 : 1 : 0.55) and [RhCp*(C_6_Me_6_)] (0.08 : 1 : 0.52).^[17c]^ Borylation of naphthalene was hampered by its propensity to sublimate, but produced mainly the 2‐isomer. Formation of the 2‐isomer was similarly seen with [Ir(OMe)COD]_2_ (5 mol %) and dtbpy (10 mol %) in cyclohexane, however, further reaction led to a mixture of doubly‐substituted products.^[28e]^ Borylation of mesitylene occurred at the benzylic position.^[21]^ Borylation of biphenyl, anisole and diphenyl ether gave the *meta* and *para* isomers as a mixture, whereas fluorobenzene produced a mixture predominantly of the *ortho* and *meta* isomers. Borylation using B_2_pin_2_ of *n*‐octane at 120 °C and *n*‐decane at 140 °C catalysed by **3** and **5** (5 mol %) was monitored using ^11^B NMR spectroscopy. The resonance for B_2_pin_2_ disappeared over the course of 4–7 days with concomitant production of HBpin and RBpin (see the Supporting Information). For alkane borylation, there was no evidence that HBpin was converted into RBpin. Borylation of decane was marginally faster at the higher temperature of 140 °C than the borylation of octane, and both reactions gave the terminal *n*‐alkylBPin products, confirmed by ^1^H NMR spectroscopy of chromatographically isolated product, although isolated yields were low. Mass spectrometry showed both mono‐ and diborylated products. Under these conditions, there was little difference between catalysts **3** and **5**, which, although slower at 120 °C compared to [RhCp*(C_2_H_4_)_2_] (*n*‐octane, 5 mol %, 5 h, 150 °C) and [RhCp*(C_6_Me_6_)] (*n*‐octane, 5 mol %, 25 h, 150 °C),^[17a]^ clearly remained active over these long reaction times. Application to a new substrate was achieved in the borylation of cholestane, a saturated tetracyclic hydrocarbon formed from cholesterol by diagenesis (Figure 2), with B_2_pin_2_ catalysed by **3**. Both mono and diborylated products were evident by mass spectrometry analysis, although, due to the complexity of the ^1^H and ^13^C NMR spectra, the position and selectivity of borylation could not be determined.

### Photolysis and stoichiometric reactions relevant to catalysis

**Scheme 6:**
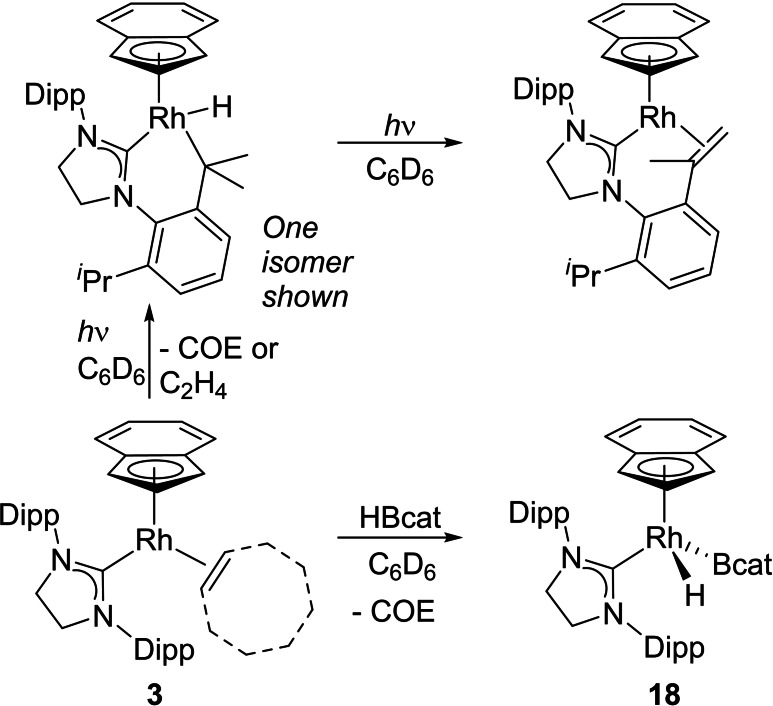
Spectroscopically investigated reactions of **3**.

With the above catalysts featuring several different ligands, and the possibility of multiple coordination modes for indenyl and fluorenyl ligands, the number of mechanistic possibilities for these systems is very large. Initially, we have focussed on the loss of a ligand from the precatalysts (Scheme 6). For [RhCp(C_2_H_4_)(CO)], UV light promotes preferential dissociation of ethene,^[9e]^ and photochemistry was used to promote silane oxidative addition to [RhCp(C_2_H_4_)_2_],^[59]^ so alkene ligands are clearly suitable for photolytic ejection. UV‐vis spectra (see the Supporting Information) show a distinct absorption for the [Rh(Ind)(SIPr)(L)] complexes (*λ*_max_: 404 nm for L=C_2_H_4_, 392 nm, for CO, 409 nm for COE) at longer wavelength compared to [Rh(Ind)(COE)_2_] (355 nm, shoulder). Changing SIPr for SIMes and IMes did not affect the wavelength of the absorption. Fluorenyl‐tethered complexes show the main absorption at shorter wavelength (ca. 350 nm), but with a shoulder at longer wavelengths. 400 and 420 nm LEDs were therefore used in photochemical studies of the monodentate complexes. CO complex **2 a** showed no change upon irradiation at these wavelengths, suggesting that CO is too strongly bound, and [Rh(Ind)(COE)_2_] also showed no change, as anticipated from its lack of absorption at 400–420 nm. Solutions of **1** and **3** in C_6_D_6_ showed changes upon irradiation, darkening in colour, and ^1^H NMR spectroscopy revealed the formation of free alkene. Also observed were resonances in the up‐field region of −15 to −17 ppm, which are indicative of the formation of Rh hydrides from C−H activation of one of the ligands. **1** showed one doublet resonance at −16.72 ppm (*J*=36.0 Hz) whereas **3** showed this doublet together with an additional doublet at −15.35 ppm (*J*=36.0 Hz) with lower integration. Continued irradiation did not lead to high conversions, hampering complete characterisation of the products. Liquid injected field desorption ionisation (LIFDI) mass spectrometry was then attempted and revealed the presence of peaks at *m*/*z* 608.26496 and 606.24958, which can be tentatively assigned to a cyclometallated rhodium hydride species ([*M*−COE]^+^, calcd: 608.26323) and its subsequent dehydrogenated product ([*M*−COE−H_2_]^+^, calcd: 606.24758). ^1^H NMR spectroscopic resonances for ethane and cyclooctane were observed for reactions of **1** and **3** respectively, demonstrating that the fate of the hydrogen is to reduce the alkenes present in the reaction mixture. The exact site of cyclometallation is not known; the Dipp substituent has isopropyl groups with both methine and methyl sites and subsequent dehydrogenation would form a propenyl donor capable of fulfilling the Rh coordination sphere and electron count.

Stoichiometric studies of these Rh complexes with B_2_pin_2_, HBpin and HBcat has proven more difficult to interpret, with several hydride resonances observed. The reaction between **3** and HBcat at room temperature was the cleanest with a single hydridic resonance observed in the ^1^H NMR spectrum (−14.84 ppm, 40.1 Hz). This resonance is broad in nature, but sharpened when a ^11^B‐decoupled ^1^H NMR spectrum was recorded, which suggests the product contains a Rh boryl hydride. In the ^11^B NMR spectrum, a broad resonance was observed at 43.9 ppm, which is consistent with a metal boryl species; resonances at 40.4 and 39.9 ppm were observed for [Rh(Cp*)(H)_2_(Bpin)_2_] and [Rh(Cp*)(H)(Bpin)_3_] respectively.^[18a]^ Due to spectral similarities with **6**, including the presence of indenyl peaks of the correct integrals, we tentatively assign the structure to [Rh(Ind)(H)(Bcat)(SIPr)] (**18**). Complex **18** was observed to be catalytically competent in the borylation of benzene with B_2_pin_2_ at 80 °C. Attempts to crystallise **18** led to degradation products, including the dinuclear complex [Rh(SIPr)(*μ*‐Bcat)_2_(*μ*‐B,O‐Bcat)Rh(H)(SiPr)] that featured a Rh−Rh bond (see the Supporting Information for details), providing evidence for a strong Rh−NHC bond and retention of this ligand in reactions with boranes.

## Conclusion

[Rh(Ind)(NHC)(L)] complexes have been synthesised for a variety of NHC (SIPr, SIMes, IMes) and L (CO, C_2_H_4_ and COE) ligands. Complexes with CO ligands were robust under APCI mass spectrometry conditions, did not react under photolysis to eject a CO ligand and did not act as catalysts for hydrocarbon borylation, thus giving evidence of a strong Rh−CO bond. Ethylene and cyclooctene complexes were more reactive, undergoing photochemically induced dissociation of the alkene ligand leading to onward reactivity through cyclometallation of the ligand. All of the [Rh(Ind)(NHC)(alkene)] complexes acted as catalysts for the borylation of arenes and alkenes, with [Rh(Ind)(SIPr)(COE)] (**3**) identified as the best precatalyst. Borylation of substituted arenes showed preference for *meta* products, driven by steric effects, except in the case of fluorobenzene. Borylation of octane and decane was achieved at 120 and 140 °C, respectively, with only B_2_pin_2_ and not HBpin acting as a boron source. Fluorenyl‐tethered NHC rhodium complexes proved to be harder to synthesise and poorer catalysts for arene borylation, thus demonstrating that indenyl rhodium NHC complexes are successful borylation catalysts, whereas tethering the NHC to a fluorenyl donor inhibits catalysis. These initial catalytic studies have identified that NHC rhodium complexes in combination with haptotropically flexible ligand sets are competent C−H borylation catalysts, and can be readily optimised in comparison to the previously identified RhCp* fragment.

## Experimental Section

Full experimental details and the general experimental description are available in the Supporting Information. General procedures are given below:

**General synthetic route to [Rh(Ind)(NHC)(alkene)]**: [Rh(Ind)(COE)_2_] (303 mg, 0.694 mol), SIPr (271 mg, 0.694 mol) and toluene (5 cm^3^) were combined in a flask equipped with a J. Young cap in a glovebox. The flask was then taken out of the glovebox and stirred at 80 °C for 16 h. The solvent was removed under vacuum giving a red‐orange wax. Pentane (5 cm^3^) was added, and the mixture stirred for 10 min before all volatiles were removed in vacuo. The residue was washed with pentane (3×10 cm^3^) and the solid dried under vacuum to afford the product [Rh(Ind)(SIPr)(COE)] (**3**) as a yellow‐orange solid (349 mg, 0.486 mmol, 70 %). Crystals suitable for X‐ray diffraction were obtained from a concentrated benzene solution.

**General synthetic routes to tethered complexes**: [Li_2_{*μ*‐N(SiMe_3_)_2_}{*μ*‐(*η* ^6^‐C_13_H_8_)C_2_H_4_N(κ‐C)N(C_2_H_4_)(Dipp)}] was formed in situ from spiro[(C_13_H_8_)C_2_H_4_N(CH)N(C_2_H_4_)(Dipp)] (211.3 mg, 0.5 mmol), Li[N(SiMe_3_)] (92.0 mg, 0.55 mmol) and LiPh (46.2 mg, 0.55 mmol) in toluene (5 cm^3^) by heating for 2 d at 80 °C.^[63b]^ [Rh(CO)_2_Cl]_2_ (97.2 mg, 0.25 mmol) in toluene (10 cm^3^) was then added at −78 °C and the reaction was allowed to warm up to room temperature and was stirred for 72 h. The reaction mixture was filtered and the solvent removed in vacuo. The product was extracted with toluene then crystallised from toluene/pet ether to yield [Rh(Flu‐Dipp)(CO)] (**13**) as red‐orange crystals (48.2 mg, 0.087 mmol, 17 %).

### General procedure for borylation reactions

**i) NMR‐scale reactions of the borylation of benzene**: In a glovebox, the Rh complex (4.0 μmol, 5 mol %), ferrocene (internal standard, 1.4 mg, 7.66 μmol) and B_2_pin_2_ (19.5 mg, 76.7 μmol) were combined in C_6_H_6_/C_6_D_6_ (0.7 mL) and added to an NMR tube equipped with a J. Young valve. The sample was then heated at 75 or 80 °C and monitored using ^1^H NMR spectroscopy.

**ii) Preparative‐scale reactions**: [Rh(Ind)(SIPr)(COE)] (13 mg, 18 μmol, 2.5 mol %), B_2_pin_2_ (199 mg, 0.784 mmol) and the substrate were combined in a flask equipped with a J. Young tap in a glovebox. The flask was then removed from the glovebox and heated using a silicone oil bath. For benzene, 5 cm^3^ was used and the reaction heated at 80 °C for 48 h. The reaction was then cooled to room temperature and excess solvent was removed under reduced pressure. The resulting crude product was then extracted using CH_2_Cl_2_ and purified using flash column chromatography on silica (16×3 cm); CH_2_Cl_2_ was used as the eluting solvent for PhBpin.

Deposition Numbers 2091749 (for **1**), 2091750 (for **2 a**), 2091751 (for **3**), 2091752 (for **4**), 2091753 (for **5**), 2091754 (for **6**), 2091755 (for **7**), 2091756 (for **8**), 2091757 (for **9**), 2091758 (for **10**), 2091759 (for **11**), 2091760 (for **13**), 2091761 (for **14**), 2091762 (for **15**), 2091763 (for **17**), 2091764 (for [Rh(SIPr)(*μ*‐Bcat)_2_(*μ*‐B,O‐Bcat)Rh(H)(SiPr)]) and 2091765 (for [Ir(Ind)(COE)_2_]) contain the supplementary crystallographic data for this paper. These data are provided free of charge by the joint Cambridge Crystallographic Data Centre and Fachinformationszentrum Karlsruhe Access Structures service.

## Conflict of interest

The authors declare no conflict of interest.

## Supporting information

As a service to our authors and readers, this journal provides supporting information supplied by the authors. Such materials are peer reviewed and may be re‐organized for online delivery, but are not copy‐edited or typeset. Technical support issues arising from supporting information (other than missing files) should be addressed to the authors.

Supporting Information
