# Supplementary material for: Rhodium Indenyl NHC and Fluorenyl‐Tethered NHC Half‐Sandwich Complexes: Synthesis, Structures and Applications in the Catalytic C−H Borylation of Arenes and Alkanes
Source: Chemistry. 2021 Nov 18;27(71):17824–33. doi: 10.1002/chem.202102961 (PMC9299238; doi:10.1002/chem.202102961)
Supplement: Supplementary file 1 — Supporting Information [file CHEM-27-17824-s001.pdf]

Chemistry–A European Journal

Supporting Information

Rhodium Indenyl NHC and Fluorenyl-Tethered NHC Half-Sandwich Complexes: Synthesis, Structures and Applications in the Catalytic C–H Borylation of Arenes and Alkanes

Kieren J. Evans, Paul A. Morton, Christian Luz, Callum Miller, Olivia Raine, Jason M. Lynam, and Stephen M. Mansell*

1 Table of Contents

2 Experimental Details 3
2.1 General details 3
2.2 General synthetic route to [Rh(Ind)(NHC)(alkene)] 4
2.2.1 [Rh(Ind)(SIPr)(COE)] (**3**) 4
2.2.2 [Rh(Ind)(SIPr)(C_2H_4)] (**1**) 4
2.3 [Rh(Ind)(IMes)(COE)] (**5**) 5
2.4 [Rh(Ind)(SIPr)(CO)] (**2a**) 5
2.5 [Rh(Ind)(SIMes)(CO)] (**2b**) 5
2.6 [Rh(Ind)(IMes)(CO)] (**2c**) 6
2.7 [Rh(Ind){Si(OEt)$_3$}(H)(SIPr)] (**6**) 6
2.8 [{Rh(μ-Cl)(SIPr)(COE)}$_2$] (**7**) 6
2.9 [{Rh(μ-Cl)(SIMes)(COE)}$_2$] (**8**) 7
2.10 [Rh(Ind)(SIMes)(COE)] (**4**) 7
2.11 [{Rh(μ-OH)(SIPr)(CO)}$_2$] (**9**) 7
2.12 Reaction of [Rh(Cp*)(C_2H_4)$_2$] with SIPr: formation of [{RhCp*(μ:η^1:η^2-C_2H_3)}$_2$] (**10**) 7
2.13 General synthetic routes to tethered complexes 8
2.13.1 [Rh(Flu-Dipp)(CO)] (**13**) 8
2.13.2 [Rh(Flu-Dipp)(C_2H_4)] (**11**) 8
2.13.3 [Rh(Flu-Dipp)(COE)] (**12**) 8
2.13.4 [Rh(Flu-Mes)(COE)] (**14**) 9
2.13.5 [Rh(Flu-Mes)(CO)] (**15**) 9
2.13.6 [Rh(Flu-Me)(COE)] (**16**) 10
2.13.7 [Rh(Flu-Me)(CO)] (**17**) 10
2.14 General procedure for borylation reactions 11
2.14.1 Borylation of benzene: PhBpin 11
2.14.2 Borylation of naphthalene: naphthylBpin 11
2.14.3 Borylation of toluene: pinB(Me-C_6H_4) 11
2.14.4 Borylation of mesitylene: pinBCH$_2$(3,5-Me$_2$)C_6H_3 11
2.14.5 Borylation of biphenyl: pinB(Ph-C_6H_4) 12
2.14.6 Borylation of anisole: pinB(OMe-C_6H_4) 12
2.14.7 Borylation of diphenylether: pinB(OPh-C_6H_4) 12
2.14.8 Borylation of fluorobenzene: pinB(F-C_6H_4) 12
2.14.9 Borylation of octane: octylBpin 13
2.14.10 Borylation of decane: decylBPin 13

2.14.11 Borylation of cholestane 13
2.15 Borylation with other complexes (NHC = IMes and SIMes, and tethered ligands) 14
2.15.1 Borylation of benzene with [Rh(Ind)(IMes)(COE)]: PhBpin 14
2.15.2 Borylation of benzene with [Rh(Ind)(SIMes)(COE)]: PhBpin 14
2.15.3 Borylation of benzene with [Rh(Flu-Mes)(COE)]: PhBpin 14
2.15.4 Borylation of benzene with [Rh(Flu-Me)(COE)]: PhBpin 14
2.15.5 Borylation of decane with [Rh(Ind)(IMes)(COE)]: DecylBpin 14
2.15.6 Borylation of octane with [Rh(Ind)(IMes)(COE)]: OctylBpin 14
2.16 General procedure for the photolysis of Rh complexes 15
2.17 General procedure for NMR scale reactions 15
3 Additional molecular structures determined by single crystal X-ray diffraction 15
3.1 Crystallographic details 15
3.2 Structures of monodentate complexes 16
3.2.1 [Rh(Ind)(SIPr)(C_2H_4)] (**1**) 16
3.2.2 [Rh(Ind)(SIPr)(COE)] (**3**): two molecules in the asymmetric unit 16
3.2.3 [Rh(Ind)(SIMes)(COE)] (**4**) 17
3.2.4 [Rh(Ind)(IMes)(COE)] (**5**): two molecules in the asymmetric unit 17
3.2.5 [{Rh(Cp*)(μ:η^1,η^2-CH=CH_2)}$_2$] (**10**) 18
3.2.6 [Ir(Ind)(COE)$_2$] 18
3.2.7 [Rh(SIPr)(μ-Bcat)$_2$(μ-B,O-Bcat)Rh(H)(SiPr)] 19
3.3 Structures of fluorenyl-tethered complexes 20
3.3.1 Molecular structure of [Rh(Flu-Dipp)(C_2H_4] (**11**) 20
3.3.2 Molecular structure of [Rh(Flu-Dipp)(CO)] (**13**) 20
3.3.3 Molecular structure of [Rh(Flu-Mes)(CO)] (**15**) 21
3.3.4 [Rh(Flu-Me)(CO)] (**17**): two molecules in the asymmetric unit 21
3.4 Comparsion of bond lengths and angles for monodentate and fluorenyl-tethered complexes 22
3.5 Molecular structures of dimeric complexes 24
3.5.1 [Rh(μ-Cl)(SIPr)(COE)]$_2$ (**7**) 24
3.5.2 [Rh(μ-Cl)(SIMes)(COE)]$_2$ (**8**) 24
3.5.3 [Rh(μ-OH)(SIPr)(COE)]$_2$ (**9**) 25
3.6 Comparison of bond lengths for the dimeric complexes 25
3.7 Crystallographic tables of data 26
4 High resolution mass spectrometry data 32
4.1 Rh complexes 32
4.1.1 [Rh(Ind)(SIPr)(COE)] (**3**) 32

4.1.2 [Rh(Ind)(SIMes)(CO)] (**2b**) 33
4.1.3 [Rh(Ind)(IMes)(CO)] (**2c**) 34
4.1.4 [Rh(Flu-Dipp)(CO)] (**13**) 36
4.1.5 [Rh(Flu-Mes)(CO)] (**15**) 37
4.2 Boron compounds 39
4.2.1 Borylation of C_6H_6/C_6D_6 mixture: PhBpin and C_6D_5Bpin 39
4.2.2 Borylation of toluene 40
4.2.3 Borylation of fluorobenzene 41
4.2.4 Borylation of mesitylene 42
4.2.5 Borylation of anisole 44
4.2.6 Borylation of biphenyl 45
4.2.7 Borylation of diphenyl ether 46
4.2.8 octylBpin and pinBC_8H_{16}BPin 47
4.2.9 decylBpin and pinB$C_{10}H_{20}$BPin 49
4.2.10 Borylation of cholestane 51
5 NMR spectra 53
5.1 Monodentate rhodium complexes 53
5.2 Fluorenyl-tethered Rh complexes 67
5.3 Arene borylation 75
5.4 Alkane borylation 84
6 UV-vis spectra 87
7 IR spectra 89
8 References 91

2 Experimental Details

2.1 General details

All reactions requiring inert condition were performed under an oxygen free nitrogen atmosphere by using standard Schlenk line techniques or by using an MBRUAN UNILab Plus glovebox, unless otherwise noted. Dry toluene, CH_2Cl_2 and THF were obtained from a solvent purification system (MBraun SP-300) and stored over 4 Å molecular sieves prior to use. Benzene was dried over molten potassium and distilled, or dried over activated 4 Å molecular sieves prior to use. Toluene-d_8 was freeze-pump-thawed and dried over activated 4 Å molecular sieves prior to use. Non-dry solvents were used as received from Fisher Scientific. For the borylation reactions, benzene, toluene, fluorobenzene, decane and octane were dried over molecular sieves. Naphthalene was dried under vacuum. The NHCs SIPr,[1] SIMes[2] and IMes[3] were synthesised according to literature methods. Alkali metal salts of the fluorenide-tethered NHC proligands $[Li_2\{\mu\text{-}N(SiMe_3)_2\}\{\mu\text{-}(\eta^5\text{-}C_{13}H_8)C_2H_4N(\kappa\text{-}C)N(C_2H_2)(Me)\}]$,[4] $[Li_2\{\mu\text{-}N(SiMe_3)_2\}\{\mu\text{-}(\eta^5\text{-}C_{13}H_8)C_2H_4N(\kappa\text{-}C)N(C_2H_4)(Mes)\}]$,[4] and $[Li_2\{\mu\text{-}N(SiMe_3)_2\}\{\mu\text{-}(\eta^5\text{-}C_{13}H_8)C_2H_4N(\kappa\text{-}C)N(C_2H_4)(Dipp)\}]$[5]were synthesised as previously described and either used as

isolated salts or formed in-situ. $RhCl_3$ hydrate was purchased from Johnson Matthey or Fisher Scientific and used to make the following starting materials, according to literature methods: [{Rh(μ-Cl)(C_2H_4)$_2$}$_2$],[6] [{Rh(μ-Cl(CO)$_2$}$_2$],[7] [{Rh(μ-Cl(COE)$_2$}$_2$],[8] [Rh(Ind)(C_2H_4)$_2$],[9] [Rh(Ind)(COE)$_2$][10] and [Rh(Cp*)(C_2H_4)$_2$][11]. [Ir(Ind)(COE)$_2$][12] and [Ir(Ind)(C_2H_4)$_2$][13] were synthesised as previously described using $IrCl_3$ hydrate purchased from ACROS. NMR spectra were obtained using either a Bruker AVIII 300 (300 MHZ), AVIII400 (400MHz) or AVIIHD (400 MHz) spectrometer. ^1H NMR spectra were recorded at either 300 MHz or 400 MHz and referenced to the residual solvent peak (7.24 for $CDCl_3$, 2.08 for toluene-d_8, and 7.16 for C_6D_6). $^{13}C\{^1H\}$ NMR spectra were recorded at 101 MHz and referenced to the residual solvent peak (77.16 for $CDCl_3$ and 128.06 for C_6D_6). $^{11}B\{^1H\}$ NMR spectra were recorded at 128.4 MHz and ^{19}F NMR spectra were recorded at 282.4 MHz, with both referenced to external samples of the appropriate standard. FTIR was performed on a Thermo Scientific Nicolet iS5/iD5 ATR spectrometer. Mass spectrometry was conducted at the National Mass Spectrometry Facility at Swansea University using the techniques stated. Electron ionization mass spectrometry (EIMS) was carried out using a Thermo MAT900XP-Trap mass spectrometer at the University of Edinburgh. Elemental analyses were performed by Dr Brian Hutton (Heriot-Watt University, non air-sensitive), Mr Stephen Boyer (London Metropolitan University, air sensitive) and Elemental Microanalysis Ltd (Okehampton, air sensitive).

2.2 General synthetic route to [Rh(Ind)(NHC)(alkene)]

[Rh(Ind)(COE)$_2$] (303 mg, 0.694 mol), SIPr (271 mg, 0.694 mol) and toluene (5 cm^3) were combined in a flask equipped with a J. Young cap in a glovebox. The flask was then taken out of the glovebox and stirred at 80 °C for 16 hours. The solvent was removed under vacuum giving a red-orange wax. Pentane (5 cm^3) was added and the mixture stirred for 10 mins before all volatiles were removed in vacuo. The residue was washed with pentane (3 x 10 cm^3) and the solid dried under vacuum to afford the product. **[Rh(Ind)(SIPr)(COE)]** (**3**) as a yellow-orange solid (349 mg, 0.486 mmol, 70 %). Crystals suitable for X-ray diffraction were obtained from a concentrated benzene solution.

2.2.1 [Rh(Ind)(SIPr)(COE)] (3)

^1H NMR (400 MHz, C_6D_6, 298 K): δ = 7.25 (d, 1H, *J* = 8.3 Hz, ArH), 7.23 (d, 1H, J = 8.3 Hz, ArH), 7.14 (d, 4H, *J* = 8.2 Hz, ArH), 6.74-6.78 (m, 2H, IndH) 6.39-6.41 (m, 3H, IndH), 4.95 (d, 2H, *J* = 2.9 Hz, IndH), 3.50 (appr. sept., 4H, *J* = 6.7 Hz, $CHMe_2$), 3.28 (s, 4H, NCH_2CH_2N), 2.58-2.60 (m, 2H, alkene), 1.89-1.93 (m, 2H, COE CH_2), 1.57-1.72 (m, 6H, COE CH_2), 1.41 (d, 12H, *J* = 6.7 Hz, $CH(CH_3)_2$), 1.27-1.37 (m, 4H, COE CH_2), 1.10 (d, 12H, *J* = 6.8 Hz, $CH(CH_3)_2$); **$^{13}C\{^1H)$ NMR (101 MHz, C_6D_6, 298 K):** δ = 214.9 (d, *J* = 70.1 Hz, carbene), 146.7, 140.2, 128.5, 124.9, 123.1, 121.0, 117.0, 97.4 (d, *J* = 6.3 Hz, Ind), 74.2 (d, *J* = 4.1 Hz, Ind), 61.5 (d, *J* = 15.3 Hz, alkene), 54.5 (d, J = 2.0 Hz, NCH_2CH_2N), 33.1 (COE CH_2), 32.4 (COE CH_2), 28.9 ($CHMe_2$), 27.1 (COE CH_2), 26.3 ($CH(CH_3)_2$), 23.8 ($CH(CH_3)_2$); **Elemental analysis** calcd (%) for $C_{44}H_{59}N_2Rh$: C 73.52, H 8.27, N 3.90; found C 73.48, H 8.29, N 4.00; **HRMS (ASAP/TOF):** Calcd. for $C_{36}H_{44}N_2Rh^+$: 607.2560, $[M-COE-H]^+$, Found: 607.2562 m/z; **UV-vis**: 7251.6 $dm^3mol^{-1}cm^{-1}$ at λ_{max} 404 nm.

2.2.2 [Rh(Ind)(SIPr)(C_2H_4)] (1)

Using [Rh(Ind)(C_2H_4)$_2$] and SIPr: [Rh(Ind)(SIPr)(C_2H_4)] (**1**) as an orange solid (140 mg, 0.220 mmol, 63%). **^1H NMR (400 MHz, C_6D_6, 298 K):** δ = 7.23 (d, 1H, *J* = 8.3 Hz, Ar*H*), 7.21 (d, 1H, *J* = 8.3 Hz, Ar*H*), 7.11 (d, 4H, *J* = 8.1 Hz, Ar*H*), 6.69-6.71 (m, 2H, Ind*H*), 6.39-6.42 (m, 2H, Ind*H*), 6.35 (q, 1H, *J* = 2.7 Hz, Ind*H*),

4.92 (d, 2H, *J* = 2.8Hz, Ind*H*), 3.39 (apparent sept., 4H, *J* = 6.7 Hz, C*H*Me_2), 3.25 (s, 4H, NCH_2CH_2N), 2.27 (br s., 4H, C_2H_4), 1.38 (d, 12H, *J* = 6.8 Hz, CH(CH_3)$_2$), 1.10 (d, 12H, *J* = 6.9 Hz, CH(CH_3)$_2$); **$^{13}C\{^1H\}$ NMR (101 MHz, C_6D_6, 298 K):** δ = 215.5 (d, *J* = 68.6 Hz, carbene), 147.0, 139.5, 128.7, 124.9, 121.4, 120.9, 116.4, 96.0 (d, *J* = 6.1 Hz, Ind), 72.9 (d, *J* = 3.9 Hz, Ind), 54.0 (d, *J* = 2.0 Hz, NCH_2CH_2N), 35.6 (d, *J* = 15.2, C_2H_4), 28.9 (*C*HMe_2), 26.3 (CH(CH_3)$_2$), 23.5 (CH(CH_3)$_2$); **Elemental analysis** calcd (%) for $C_{38}H_{49}N_2Rh$: C 71.68, H 7.76, N 4.40; found C 71.73, H 7.85, N 4.45; **UV-vis**: 5391.6 $dm^3mol^{-1}cm^{-1}$ at λ_{max} 409 nm.

2.3 [Rh(Ind)(IMes)(COE)] (5)

[Rh(Ind)(COE)$_2$] (235.3 mg, 0.517 mmol), IMes (163.8 mg, 0.538 mmol) and toluene (5 cm^3) were combined in a flask equipped with a J. Young cap in a glovebox. The flask was then taken out of the glovebox and stirred at 60 °C for 16 hours. The solvent was removed under vacuum giving a red-orange glass. The residue was dissolved in pentane (15 cm^3), filtered and then concentrated in volume under reduced pressure. After storage at -25°C for 16 h, the product was obtained as an orange microcrystalline ppt that was dried under vacuum (160 mg, 0.253 mmol, 49%). Single crystals suitable for X-ray diffraction were grown from pentane solution.

^1H NMR (400 MHz, C_6D_6, 298 K): δ = 7.01 (s, 4H, Ar), 6.79 (s, 4H, Ar), 6.07 (s, 2H, Ar), 5.84 (q, 1H, Ar), 4.43 (d, 2H, Ar), 2.14 (s, 6H, CH_3), 2.04 (s, 12H, CH_3). **$^{13}C\{^1H\}$ NMR (400 MHz, C_6D_6,298 K):** δ = 185.7 (d, *J* = 70.9 Hz, carbene C), 138.7 (Ar C), 138.1 (Ar C), 135.9 (Ar C), 129.2 (Ar CH), 123.0 (d, J = 1.4 Hz, NCHCHN, 2 x CH), 119.2 (Ar C), 117.6 (Ar CH), 96.6 (d, J= 6.0 Hz, Ar CH), 72.2 (d, J= 4.3 Hz, Ar CH), 57.7 (d, J = 16.3 Hz, COE HC=CH), 32.8 (COE CH_2), 27.3 (COE CH_2), 21.0 (CH_3), 17.2 (CH_3); **Elemental analysis** calcd (%) for $C_{38}H_{45}N_2Rh$: C 72.14, H 7.17, N 4.43; found C 72.15, H 7.24, N 4.68.

2.4 [Rh(Ind)(SIPr)(CO)] (2a)

To a solution of [Rh(Ind)(SIPr)(C_2H_4)] (270 mg, 0.424 mmol) in toluene (5 cm^3), CO was bubbled through at the rate of 1 bubble per second for 30 mins. The solution lightened from orange to yellow-orange. The solvent was removed in vacuo and the product was obtained as a yellow solid (214 mg, 0.336 mmol, 79 %). Crystals suitable for X-ray diffraction were obtained from a concentrated benzene solution.

^1H NMR (C_6D_6, 400 MHz, 298 K): δ = 7.24 (d, 1H, *J* = 8.2 Hz, ArH), 7.22 (d, 1H, *J* = 8.2 Hz, ArH), 7.11 (d, 4H, *J* = 7.9 Hz, ArH), 6.75-6.78 (m, 2H, IndH), 6.56-6.59 (m, 2H, IndH), 6.01 (q, 1H, *J* = 2.8 Hz, IndH), 5.06 (d, 2H, *J* = 2.9 Hz, IndH), 3.34 (s, 4H, NCH_2CH_2N), 3.25 (appr. sept., 4H, *J* = 6.8 Hz, C*H*Me_2), 1.31 (d, 12H, *J* = 6.8, CH(CH_3)$_2$), 1.13 (d, 12H, J = 6.9 Hz, CH(CH_3)$_2$); **$^{13}C\{^1H)$ NMR (C_6D_6, 101 MHz, 298 K):** δ = 214.8 (d, *J* = 68.8 Hz, carbene), 195.1 (d, *J* = 93.3 Hz, CO), 147.3, 139.2, 129.2, 125.1, 122.8, 122.0, 116.8, 99.9 (d, J = 6.5, IndH), 71.1 (IndH), 54.0 (d, J = 2.0 Hz, NCH_2CH_2N), 29.0 (*C*HMe_2), 26.1 (CH(CH_3)$_2$), 23.6 (CH(CH_3)$_2$); **IR (thin film)/ cm^{-1}:** 1944 (CO); **Elemental analysis calcd** (%) for $C_{37}H_{45}N_2ORh$: C 69.80, H 7.12, N 4.40; found C 69.72, H 7.21, N 4.29; **UV-vis**: 6909.4 $dm^3mol^{-1}cm^{-1}$ at λ_{max} 392 nm.

2.5 [Rh(Ind)(SIMes)(CO)] (2b)

A solution of [Rh(Ind)(SIMes)(COE)] (**4**) (15 mg, 0.024 mmol) in C_6D_6 (0.7 cm^3) was degassed at -78°C and CO (1 atm in NMR tube) was added. The solution was allowed to return to room temperature with shaking and turned orange in colour.

^{1}H NMR (400 MHz, C_6D_6, 298 K): δ = 6.91 (s, 4H, Ar), 6.77 (s, 4H, Ar), 5.83 ppm (q, d= 2.7 Hz, 1H, CH), 4.75 (s, 2H, CH), 3.09 (s, 4H, H_2C-CH_2), 2.15 (s, 12H, CH_3), 2.08 (s, 6H, CH_3). **^{13}C{^{1}H} NMR (400 MHz, C_6D_6, 298 K):** δ = 211.1 (d, J = 66 Hz, carbene C), 195.4 (d, J = 94 Hz, -CO) 138.5 (Ar C), 137.6 (Ar C), 130.3 (Ar C), 129.7 (2 Ar CH), 121.4 (Ar CH), 117.4 (Ar CH), 98.4 (d, J = 6.2 Hz, Ar CH), 71.0 (Ar CH), 50.8 (d, J = 1.8 Hz, NCH_2CH_2N), 21.0 (CH_3), 18.5 (CH_3).IR (toluene)/ cm^{-1}: 1939 (CO). **HRMS (ASAP/TOF):** Calcd. for $C_{31}H_{34}N_2O^{103}Rh^+$: 553.1726, $[M+H]^+$, Found: 553.1735 m/z.

2.6 [Rh(Ind)(IMes)(CO)] (2c)

A solution of [Rh(Ind)(IMes)(COE)] (**5**) (15 mg, 0.024 mmol) in C_6D_6 (0.7 cm^3) was degassed at -78°C and CO (1 atm in NMR tube) was added. The solution was allowed to return to room temperature with shaking and turned orange in colour.

^{1}H NMR (400 MHz, C_6D_6, 298 K): δ = 6.91 (br s, 4H, Ar), 6.77 (s, 4H, Ar), 6.18 (s, 2H, NCH=CHN), 5.97 ppm (br s, 1H, CH), 4.75 (s, 2H, CH), 2.09 (s, 12H, CH_3), 2.01 (s, 6H, CH_3). **^{13}C{^{1}H} NMR (400 MHz, C_6D_6, 298 K):** δ = 195.0 (d, J = 93 Hz, carbene C), 195.4 (d, J = 70 Hz, CO) 138.9 (Ar C), 137.9 (Ar C), 130.4 (Ar C), 129.4 (Ar CH), 122.3 (d, 1.4 Hz, NCHCHN), 121.3 (Ar CH), 117.2 (Ar CH), 98.1 (d, J = 6.1 Hz, Ar CH), 71.0 (br s, Ar CH), 21.1 (CH_3), 18.6 (CH_3). IR (toluene)/ cm^{-1}: 1938 (CO). **HRMS (ASAP/TOF):** Calcd. for $C_{31}H_{32}N_2O^{103}Rh^+$: 551.1570, $[M+H]^+$, Found: 551.1565 m/z.

2.7 [Rh(Ind){Si(OEt)$_3$}(H)(SIPr)] (6)

In the glovebox, [Rh(Ind)(SIPr)(COE)] (**3**) (75.0 mg, 0.104 mmol) was dissolved in toluene (5 cm^3) and added to a Schlenk flask. To this solution, $(EtO)_3SiH$ (77 µL, 0.313 mmol, 3 eq.) was added. The reaction mixture was heated to 40 °C and stirred for 16 h. The volatiles were then removed in vacuo and the residue washed with cold pentane (3 x 5 cm^3), giving the product as a yellow solid (54.3 mg, 0.0703 mmols, 67 %). Crystals suitable for X-ray diffraction were obtained from a concentrated benzene solution. It was noted that in both the ^{1}H and ^{13}C NMR spectra, broad peaks were observed which point to a dynamic process in solution.

^{1}H NMR (400 MHz, C_6D_6, 298 K): δ = 7.3 (br, 6H), 7.0 (br m, 2H), 6.9 (br m, 2H) 5.77 (td, 1H, *J* = 2.8, 1.4 Hz, IndH), 4.90 (br ca 1H), 4.49 (br, ca 1H), 3.83 (br, ca 8H), 3.63 (br, 2H), 3.29 (br 2H), 3.07 (br, ca 1H), 2.88 (br, ca 1H), 1.62 (d, J = 6.1 Hz), 1.21 (t, J =7.0 Hz), 1.18 (br d), 1.03 (br), 0.71 (br), -15.07 (d, 1H, $^1J_{Rh\text{-}H}$ = 30.6 Hz, Si satellites $^2J_{Si\text{-}H}$ = 15.8 Hz, Rh-H); **^{13}C{^{1}H) NMR (101 MHz, C_6D_6, 298 K)**: δ = 214.5 (d, *J* = 60.5 Hz, carbene), 147.8 (br), 140.0, 128.3, 128.6, 124.9, 124.3, 122.0, 121.0 100.8 (d, *J* = 3.8 Hz, IndH), 82.1 (br), 64.0 (br), 58.3 (O*C*H_2CH_3), 53.4 (NCH_2CH_2N), 27.7 (*C*HMe$_2$), 26.5 (CH(*C*H_3)$_2$), 23.1 (CH(*C*H_3)$_2$), 18.5 (OCH_2*C*H_3); **^{29}Si{^{1}H} NMR (79.5 MHz, C_6D_6, 298 K):** δ = -13.77 (J = 67.3 Hz).

2.8 [{Rh(µ-Cl)(SIPr)(COE)}$_2$] (7)

In the glovebox, SIPr (109.0 mg, 0.279 mmol, 2 eq.) and $[Rh(COE)_2Cl]_2$ (100 mg, 0.139 mmol) were added to a Schlenk flask along with toluene (5 cm^3). The reaction mixture was stirred for 16 h at room temperature. The initial reaction mixture turned from cloudy-orange to clear yellow-orange, and then cloudy yellow. The volatiles were removed in vacuo and the residue was washed with pentane (3 x 5 cm^3) and dried thoroughly under vacuum to afford the product as a yellow solid (131.3 mg, 0.103 mmol, 74 %). Crystals suitable for X-ray diffraction were obtained from a concentrated benzene solution. It was noted that [{Rh(µ-Cl)(SIPr)(COE)}$_2$] (**7**) was poorly soluble in C_6D_6, hampering characterisation by NMR spectroscopy, and decomposes in $CDCl_3$.

^{1}H NMR (400 MHz, C_6D_6, 298 K): δ = 7.40-7.27 (m), 7.00-6.96 (m), 3.59 (br. sept., J = 6.7 Hz, C*H*(CH_3)$_2$), 3,25 (br. s, NCH_2CH_2N), 3.81 (br d, COE alkene), 1.81-1.34 (br m, COE CH_2), 1.15 (d, J = 6.7 Hz, CH(C*H*$_3$)$_2$).

2.9 [{Rh(μ-Cl)(SIMes)(COE)}$_2$] (8)

[Rh(μ-Cl)(COE)$_2$]$_2$ (108 mg, 0.150 mmol) and SIMes (90.0 mg, 0.294 mmol) were added to a Schlenk flask in a glovebox with toluene (10 cm^3) and the mixture was stirred overnight. The solvent was removed under vacuum. Crude product was redissolved in toluene, concentrated and placed overnight at -25°C. The solution was filtered away from the precipitate, the product was then dried under vacuum giving a yellow solid (93 mg, 0.0933 mmol, 63%). Single crystals suitable for X-ray diffraction were grown from a benzene solution.

^{1}H NMR (400 MHz, toluene-d_8, 298 K): δ = 6.86 (s, 4H, Ar), 6.81 (s, 4H, Ar), 2.99 (d, J= 6.8 Hz, 8H, NCH_2CH_2N), 2.74 (t, J = 4.3 Hz, 4H, HC=CH COE), 2.44 (s, 12H, CH_3) 2.32 (s, 12H, CH_3), 2.28 (s, 12H, CH_3), 1.86-1.76 (m, 8H, CH_2 COE), 171-1.61 (m, 8H, CH_2 COE), 1.50-1.39 (m, 4H, CH_2 COE) 1.39-1.26 (m, 4H, CH_2 COE). **^{13}C{^{1}H} NMR (101 MHz, toluene-d_8, 298 K):** δ = 210.3 (d, J = 57.3 Hz, carbene C), 138.7 (Ar C), 137.7 (Ar C), 136.4 (Ar C), 130.4 (Ar CH), 60.4 (d, J= 16.3 Hz, HC=CH, COE), 51.5 (NCH_2CH_2N), 30.9 (COE CH_2), 29.8 (COE CH_2), 27.5 (COE CH_2), 21.2 (CH_3), 18.6 (CH_3).

2.10 [Rh(Ind)(SIMes)(COE)] (4)

[Rh(μ-Cl)(SIMes)(COE)]$_2$ (**8**) (93.0 mg, 0.084 mmol) and lithium indenide (29.3 mg, 0.239 mmol) were added to a Schlenk in the glovebox together with toluene (10 cm^3) and the mixture stirred overnight. The solvent was removed under vacuum and the solid was washed with pentane. The orange solid was dried under vacuum (64 mg, 0.101 mmol, 60 %).

^{1}H NMR (400 MHz, C_6D_6, 298 K): δ = 6.94 (m, 4H, Ar), 6.81 (s, 4H, Ar), 5.84 ppm (m, 1H, CH), 4.55 (m, 2H, CH), 2.96 (s, 4H, CH_2), 2.18 (s, 12H, CH_3), 2.13 (s, 6H, CH_3). **^{13}C{^{1}H} NMR (400 MHz, C_6D_6, 298 K):** δ = 214.7 (d, J = 68 Hz, carbene C), 139.6 (Ar C), 137.1 (Ar C), 136.4 (Ar C), 129.6 (Ar CH), 120.4 (Ar CH), 119.7 (Ar C), 117.7 (Ar CH), 97.9 (d, J= 5.9 Hz, Ar CH), 73.9 (d, J = 4.1 Hz, Ar CH), 59.0 (d, J= 16 Hz, HC=CH COE), 51.7 (d, J = 1.8 Hz, NCH_2CH_2N), 32.8 (d, J = 1.8 Hz, COE CH_2), 27.3 (COE CH_2), 21.0 (CH_3), 18.5 (CH_3)

2.11 [{Rh(μ-OH)(SIPr)(CO)}$_2$] (9)

To a solution of [Rh(Ind)(SIPr)(COE)] (**3**) (15 mg) in C_6D_6 (0.7 cm3), water (50 μL) was added. The reaction was monitored by ^{1}H NMR spectroscopy over the course of 3 days. The formation of free indene was observed and new resonances for the product of this reaction. Crystals suitable for X-ray diffraction were obtained from a concentrated benzene solution. **^{1}H NMR (400 MHz, C_6D_6, 298 K):** δ = 7.32-7.2 (m, ArH), 7.15-6.95 (m, ArH), 3.33 (s, NCH2CH2N), 3.32 (sept., J = 6.9 Hz, CHMe2), 1.61 (d, 24H, J = 6.7 Hz, CH(CH3)2), 1.13 (d, 24H, J = 6.9 Hz, CH(CH3)2), -2.6 (s, 2H).

2.12 Reaction of [Rh(Cp*)(C_2H_4)$_2$] with SIPr: formation of [{RhCp*(μ:η^1:η^2-C_2H_3)}$_2$] (10)

[Rh(Cp*)(C2H4)$_2$] (372.7 mg, 1.266 mmol) and SIPr (494.7 mg, 1.266 mmol) were combined in toluene (5 cm^3) and the reaction mixture was heated at 110 °C for 40 hrs. The solvent was removed in vacuo and pentane (5 cm^3) was added. After stirring for 15 mins, the mixture was filtered and the residue washed with pentane (3 x 3 cm^3). The filtrate was concentrated to a third and placed in the freezer for

1 week. During this time crystals had formed and were isolated by filtration of the supernatant solution. The crystals were found to be a mixture of [Rh(Cp*)(C_2H_4)$_2$] and [{RhCp*(C_2H_3)}$_2$] (**10**). Due to similar solubilities it was not possible to purify the product.

2.13 General synthetic routes to tethered complexes

[Li$_2${μ-N(SiMe$_3$)$_2$}{μ-(η^6-$C_{13}H_8$)C_2H_4N(κ-C)N(C_2H_4)(Dipp)}] was formed in-situ from spiro[($C_{13}H_8$)C_2H_4N(CH)N(C_2H_4)(Dipp)] (211.3 mg, 0.5 mmol), Li[N(SiMe$_3$)] (92.0 mg, 0.55 mmol) and LiPh (46.2 mg, 0.55 mmol) in toluene (5 cm^3) by heating for 2 d at 80°C.[5] [Rh(CO)$_2$Cl]$_2$ (97.2 mg, 0.25 mmol) in toluene (10 cm^3) was then added at -78 °C and the reaction was allowed to warm up to room temperature and stir for 72 h. The reaction mixture was filtered and the solvent removed in vacuo. The product was extracted with toluene then crystallised from toluene/pet ether to yield [Rh(Flu-Dipp)(CO)] (**13**) as red-orange crystals (48.2 mg, 0.087 mmol, 17%).

2.13.1 [Rh(Flu-Dipp)(CO)] (13)

^{1}H NMR (C_6D_6, 400 MHz, 298 K): δ = 7.70-7.72 (m, 2H, FluH), 7.26-7.28 (m, 2H, FluH), 7.03-7.12 (m, 5H, overlap of FluH + Dipp ArH), 6.93 (d, 2H, *J* = 7.7 Hz, Dipp ArH), 3.30-3.33 (m, 2H, CH$_2$), 3.14-3.19 (m, 2H, CH$_2$), 2.92-2.96 (m, 2H, CH$_2$), 2.59-2.70 (m, 4H, overlap of C*H*Me$_2$ + CH$_2$), 1.02 (d, 6H, *J* = 7.0 Hz, CH(C*H*$_3$)$_2$), 0.92 (d, 6H, *J* = 6.7 Hz, CH(C*H*$_3$)$_2$); **^{13}C{^{1}H} NMR (C_6D_6, 101 MHz, 298 K):** δ = 214.1 (d, *J* = 78.8 Hz, carbene), 192.9 (d, *J* = 90.9 Hz, CO), 147.3, 137.5, 129.3, 128.7, 124.7, 122.5, 121.5, 119.8, 118.1 (d, *J* = 3.2 Hz), 116.6, 102.0, 70.6, 53.8, 51.4, 28.5(*C*H(CH$_3$)$_2$), 25.4 (CH(*C*H$_3$)$_2$), 23.4 (CH(*C*H$_3$)$_2$); **IR (thin film)/cm^{-1}:** 1948; **HRMS (ASAP/TOF):** Calcd. for $C_{31}H_{34}N_2ORh^+$: 553.1721, [M+H]$^+$, Found: 553.1714 m/z. Calcd. for $C_{30}H_{33}N_2Rh^+$: 524.1699, [M-CO]$^+$, Found: 524.1687 m/z. Calcd. for $C_{31}H_{34}N_2O_2Rh^+$: 569.1670, [M+OH]$^+$, Found: 569.1666 m/z.

2.13.2 [Rh(Flu-Dipp)(C_2H_4)] (11)

Using [Rh(C_2H_4)$_2$Cl]$_2$ (97.2 mg, 0.25 mmol): yellow crystals of [Rh(Flu-Dipp)(C_2H_4)] (15 mg, 0.024 mmol, 5 %).

^{1}H NMR (400 MHz, C_6D_6, 298 K): δ = 7.48-7.50 (m, 2H, FluH), 7.35-7.37 (m, 2H, FluH), 7.11-7.15 (m, 4H, FluH, partial overlap with residual C_6H_6 peak),7.00 (t, 1H, *J* = 7.8 Hz, Dipp ArH), 6.84 (d, 2H, J = 7.6 Hz, Dipp ArH), 3.40-3.44 (m, 2H, CH$_2$), 2.99-3.07 (m, 2H, CH$_2$), 2.73-2.87 (m, 6H, overlap of 2x CH$_2$ and 2x C*H*Me$_2$), 1.29 (br. s., 4H, C_2H_4), 0.99 (d, 6H, *J* = 6.7 Hz, CH(C*H*$_3$)$_2$), 0.96 (d, 6H, J = 6.9 Hz, CH(C*H*$_3$)$_2$); **^{13}C{^{1}H} NMR (101 MHz, C_6D_6, 298 K):** δ = 213.0 (d, *J* = 79.5 Hz, carbene), 147.0, 137.6, 128.6, 124.3, 122.2, 120.8, 118.8, 117.3, 116.3, 116.1, 103.3, 72.0 (d, *J* = 5.4 Hz, Flu), 55.1 (d, *J* = 3.0 Hz, CH$_2$), 51.9 (CH$_2$), 50.8 (CH$_2$), 44.7 (d, *J* = 13.6 Hz, ethene), 28.4 (CHMe$_2$), 26.3 (CH(CH$_3$)$_2$), 23.3 (CH(CH$_3$)$_2$).

2.13.3 [Rh(Flu-Dipp)(COE)] (12)

[Li$_2${μ-N(SiMe$_3$)$_2$}{μ-(η^6-$C_{13}H_8$)C_2H_4N(κ-C)N(C_2H_4)(Dipp)}] was formed in-situ from spiro[($C_{13}H_8$)C_2H_4N(CH)N(C_2H_4)(Dipp)] (211.3 mg, 0.5 mmol), Li[N(SiMe$_3$)] (92.0 mg, 0.55 mmol) and LiPh (46.2 mg, 0.55 mmol) in toluene (5 cm^3) by heating for 2 d at 80°C.[5] [Rh(COE)Cl]$_2$ (179.4 mg, 0.25 mmol) in toluene (10 cm^3) was then added at -78 °C and the reaction was allowed to warm up to room temperature and stir for 72 h. The reaction mixture was filtered and the solvent removed in vacuo. The ^{1}H and ^{13}C NMR spectra of crude product confirmed the presence of the expected product and spiro[($C_{13}H_8$)C_2H_4N(CH)N(C_2H_4)(Dipp)]. The product was found to be soluble in alkane solvents such as

pentane and hexanes and thus could not be separated from the spirocycle impurity by washing due to the similar solubility between the two species. Attempts to filter the crude mixture through either silica or alumina led to no recoverable product.

Also attempted:

$[Li_2\{\mu\text{-}N(SiMe_3)_2\}\{\mu\text{-}(\eta^6\text{-}C_{13}H_8)C_2H_4N(\kappa\text{-}C)N(C_2H_4)(Dipp)\}]$ (100 mg, 0.168 mmol) and $[Rh(COE)Cl]_2$ (60.2 mg, 0.084 mmol) were combined as solids in a glovebox, toluene (5 ml) was added and the reaction mixture was stirred. The reaction mixture was filtered and the solvent removed in vacuo. Attempted purification via filtering though alumina gave no isolable product

Spiro$[(C_{13}H_8)C_2H_4N(CH)N(C_2H_4)(Dipp)]$ (150 mg, 0.355 mmol), $Na[N(SiMe_3)]$ (68.3 mg, 0.373 mmol) and NaBn (43.5 mg, 0.373 mmol) were stirred at rt for 24hrs in toluene (5 cm^3).[5] $[Rh(COE)Cl]_2$ (127.3 mg, 0.177 mmol) in toluene (10 cm^3) was then added at -78 °C and the reaction was allowed to warm up to room temperature and stir for 72 h. The reaction mixture was filtered through celite and the solvent removed in vacuo. Attempted purification via filtering though silica gave no isolable product.

Selected $^{13}C\{^1H\}$ NMR resonances (101 MHz, C_6D_6, 298 K): δ = 210.8 (*J* = 83.0 Hz), 61.4 (*J* = 13.5 Hz).

2.13.4 [Rh(Flu-Mes)(COE)] (14)

To a toluene solution of the Mes-spirocycle[4] (190 mg, 0.50 mmol 1 equiv.) and $LiN(SiMe_3)_2$ (84 mg, 0.50 mmol, 1.1 equiv.), n-BuLi (0.3 mL, 0.387 mmol 0.77 equiv.) was added at -78°C, and upon warming to room temperature, the reaction was left to stir for 24 h. $[Rh(COE)_2Cl]_2$ (179 mg, 0.25 mmol, 0.5 equiv.) was added at -78°C. The solution was left to warm to room temperature and subsequently stirred for 2 d. All solvent was moved under reduced pressure and the resulting solid was washed with pentane (5 cm^3). The product was extracted with toluene and the solvent removed under reduced pressure to yield [Rh(Flu-Mes)(COE)] (**14**) as a brown powder (77 mg, 0.13 mmol, 26%). Single crystals of the complex were obtained from slow evaporation of a benzene solution.

^1H NMR (400 MHz, $CDCl_3$, 298 K): δ = 7.58 -7.55 (m, 2H, Flu H), 7.48 -7.45 (m,2H, Flu H), 7.22 – 7.18 (m, 4H, Flu H), 6.52 (s, 2H, Mes H), 3.44 – 3.40 (m, 2H, NHC-CH_2), 2.81 – 2.77 (m, 4H, NHC-CH_2 and ethyl tether CH_2), 2.73 – 2.69 (m, 2H, ethyl tether CH_2), 1.99 (s, 3H, CH_3), 1.93 (s, 6H, CH_3), 1.65-1.40 (m, 7H, COE), 1.36-1.18 (m, 5H, COE), 1.14-1.04 (m, 2H, COE); **$^{13}C\{^1H\}$ NMR (101 MHz, $CDCl_3$, 298K):** δ = 212.6 (d, J= 81.3 Hz, carbenic C), 137.0 (Ar C), 136.2 (Ar C), 129.1 (Ar C), 127.4 (Ar CH), 121.8 (Ar CH), 120.0 (Ar CH) 119.7 (Ar CH), 117.8 (ArCH), 117.4 (d, J= 3.3, Ar CH) 106.1 (ArC), 71.7 (d, J= 5.0,Flu-9), 61.7 (d, J=13.43, Rh(HC=CH)), 52.4 (d, J= 4.1, NCH_2CH_2N), 51.4 (d, J=1.70, NCH_2CH_2N) 50.7 (d, J= 1.70, NCH_2CH_2), 32.5 (CH_2CH_2Flu), 29.5 (CH_2), 27.0 (CH_2), 24.1(CH_2), 20.8(CH_3), 18.9(CH_3).

2.13.5 [Rh(Flu-Mes)(CO)] (15)

Using $[Rh(CO)_2Cl]_2$ (194.4 mg, 0.50 mmol) yielded [Rh(Flu-Mes)(CO)], **15**, as an orange powder (18 mg, 0.03 mmol, 7%).

^1H NMR (400 MHz, $CDCl_3$, 298 K): δ = 7.78 -7.72 (m, 2H, FluH), 7.33 -7.30 (m,2H, FluH), 7.05 – 7.00 (m, 4H, FluH), 6.67 (s, 2H, MesH), 3.91 – 3.88 (m, 2H, NCH_2CH_2N), 3.82 – 3.77 (m, 2H, NCH_2CH_2N), 3.59 – 3.55 (m, 2H, CH_2CH_2), 2.98 – 2.95(m, 2H, CH_2CH_2Flu), 2.16 (s, 3H, CH_3), 1.77 (s, 6H, CH_3); **$^{13}C\{^1H\}$ NMR (101 MHz, $CDCl_3$, 298 K):** δ = 211.0 (d, J= 78.3, RhC), 191.2 (d, J = 90.7, RhCO), 137.6 (Ar C), 136.7 (Ar C), 129.2 (Ar CH), 129.1 (Ar CH), 122.4 (Ar CH), 120.9 (Ar CH), 119.7 (Ar CH) 117.9 (d, J= 2.9 Hz, Ar C), 116.5 (ArCH), 101.9 (ArC), 71.0 (d, J= 3.8 Hz, Flu-9), 51.6 (d, J= 1.5 Hz, NCH_2CH_2N), 51.5 (d, J= 2.75,

NCH_2CH_2), 23.6 (CH_2CH_2Flu), 21.0 (CH_3), 18.3 (CH_3); **HRMS (ASAP/TOF):** Calcd. for $C_{28}H_{28}N_2O^{103}Rh^+$: 511.1257, [M+H]$^+$, Found: 511.1255 m/z. Calcd. for $C_{27}H_{26}N_2{}^{103}Rh^+$: 481.1151, [M-H-CO]$^+$, Found: 481.1151 m/z; **IR (thin film)/ cm^{-1}:** 1965 (CO).

2.13.6 [Rh(Flu-Me)(COE)] (16)

To a solution of $Li_2\{\mu\text{-}N(SiMe_3)_2\}\{\mu\text{-}(\eta^5\text{-}C_{13}H_8)C_2H_4N(\kappa\text{-}C)N(C_2H_2)(Me)\}]$[4] (80 mg, 0.18 mmol, 2 eq.) in toluene 5 cm^3) was added a solution of $[Rh(COE)_2Cl]_2$ (63 mg, 0.09 mmol, 1 eq.) in toluene (5 cm^3) at -78°C. After stirring for 48 hours at room temperature, the reaction mixture was filtered through Celite® and the solvent was removed, before the residue was washed with pentane (3 x 3 cm^3). The solvent was removed, and the product was dried under high vacuum yielding [Rh(Flu-Me)(COE)] (**16**) as an orange powder (27.0 mg, 0.055 mmol, 31%).

^{1}H NMR (400 MHz, C_6D_6, 298 K): δ = 7.75 (d, 2H, J = 8.1 Hz, ArCH), 7.28-7.20 (m, 4H, ArCH), 7.15-7.10 (m, 2H, ArCH), 6.11 (d, 1H, J = 1.9 Hz, HC=CH), 5.74 (d, 1H, J = 1.7 Hz, HC=CH), 3.90-3.80 (m, 2H, NCH_2CH_2), 2.81-2.72 (m, 2H, CH_2CH_2Flu), 2.57-2.50 (m, 2 H, RhCOE), 2.41 (s, 3H, RhCOE), 1.76-1.31 (m, 12H, RhCOE); **$^{13}C\{^1H\}$ NMR (101 MHz, C_6D_6, 298 K):** δ = 180.2 (d, J = 84.1 Hz, carbenic C), 129.3 (s, CH arom.), 121.5 (d, J = 2.4 Hz, C arom.), 120.2 (d, J = 2.2 Hz, C arom.), 119.9 (s, ArCH), 119.3 (s, ArCH), 117.8 (d, J = 2.9 Hz, ArC), 116.8 (s, ArCH), 105.8 (s, ArC), 67.7 (d, J = 4.9 Hz, Flu-9), 60.0 (d, J = 14.2 Hz, Rh(HC=CH)), 53.2 (d, J = 1.5 Hz, NCH_2CH_2), 36.3 (d, J = 3.7 Hz, CH_2CH_2Flu), 32.8 (s, CH_2,COE), 30.0 (s, CH_2,coe), 27.1 (s, Me), 24.9 (s, CH_2, COE), 21.4 (s, CH_2, COE).

2.13.7 [Rh(Flu-Me)(CO)] (17)

A solution of [Rh(Flu-Me)(COE)] (**16**) (18 mg, 0.037 mmol) in C_6D_6 (0.7 cm^3) was degassed at -78°C and CO (1 atm in NMR tube) was added. The solution was allowed to return to room temperature with shaking, turning at first orange in colour, and the complex then precipitated out as an orange suspension. Crystals suitable for x-ray diffraction were grown from a solution of **17** in a mixture of dichloromethane and petroleum ether at -25°C.

^{1}H NMR (400 MHz, C_6D_6, 298 K): δ = 7.96-7.92 (m, 2H, ArCH), 7.19-7.00 (m, 6H, ArCH), 6.05 (d, 1H, J = 1.9 Hz, HC=CH), 5.75 (d, 1H, J = 1.3 Hz, HC=CH), 3.69-3.65 (m, 2H, NCH_2CH_2), 2.68 (s, 3H, Me), 2.46-2.42 (m, 2H, CH_2CH_2Flu); **$^{13}C\{^1H\}$ NMR (101 MHz, C_6D_6, 298 K):** δ = 193.2 (d, J = 89.3 Hz, RhCO), 180.0 (d, J = 81.3 Hz, RhC), 125.7 (s, ArC), 122.4 (s, ArCH), 121.7 (s, HC=CH), 121.3 (s, HC=CH), 121.0 (s, ArCH), 120.0 (s, ArCH), 116.7 (s, ArCH), 102.5 (s, ArC), 70.6 (d, J = 3.0 Hz, Flu-9), 52.3 (d, J = 1.0 Hz, NCH_2CH_2), 38.0 (d, J = 1.8 Hz, CH_2CH_2Flu), 24.0 (s, Me); **IR (thin film)/ cm^{-1}:** 1959 (CO).

2.14 General procedure for borylation reactions

The reactants were mixed in a flask equipped with a J. Young tap in a glovebox. The flask was then removed from the glovebox and heated using a silicon oil bath at either 80 °C, 110 °C or 150 °C for either 24 hours or 72 hours. The reaction was then cooled to room temperature and excess solvent was removed under reduced pressure. The resulting crude product was then extracted using CH_2Cl_2 and then purified using flash column chromatography on silica (16 cm x 3 cm) and the appropriate solvent as eluting agent.

2.14.1 Borylation of benzene: PhBpin

[Rh(Ind)(SIPr)(COE)] (12.5 mg, 17.4 µmol), B_2pin_2 (199 mg mg, 0.784 mmol) and benzene (5 cm^3) were stirred at 80°C for 48 h. The purified product was isolated via column chromatography using CH_2Cl_2as the eluting agent, giving PhBpin as a colourless oil (130 mg, 0.637 mmol, 81%). **^{1}H NMR (400 MHz, CDCl$_3$, 298 K):** δ = 7.81 (dd, 2H, J = 8.0, 1.4 Hz, ortho ArH), 7.46 (tt, 1H, J = 7.4, 1.5 Hz, para ArH), 7.36 (tt, 2H, J = 7.2, 1.2 Hz, meta ArH), 1.35 (s, 12H, Me). ^{1}H and ^{13}C{^{1}H} NMR spectral data match literature values.[14] **^{11}B{^{1}H} NMR (128 MHz, CDCl$_3$, 298 K):** δ = 30.97 (s).

MS experiments using C_6H_6/C_6D_6 (EI/MS): Calcd. for $C_{12}H_{17}BO_2^+$: 204.1 $[M]^+$, Found: 204.1 $[M]^+$ and 189.1 $[M-CH_3]^+$ m/z. Calcd. for $C_{12}H_{12}D_5BO_2^+$: 209.2 $[M]^+$, Found: 209.2 $[M]^+$ and 194.1 $[M-CH_3]^+$ m/z.

2.14.2 Borylation of naphthalene: naphthylBpin

Following the general procedure using naphthalene (201.2 mg, 1.57 mmol, 2 eq.), the product was obtained as colourless oil (76 mg, 0.299 mmol, 38 %). **^{1}H NMR** spectral data match literature values as a mixture of isomers (1-position: 0.07, 2-position: 1).[15-16] **^{11}B{^{1}H} NMR (128 MHz, CDCl$_3$, 298 K):** δ = 31.1 (s). **MS (EI/MS):** Calcd. for $C_{16}H_{19}BO_2^+$: 254.1 $[M]^+$, Found: 254.1 $[M]^+$ and 239.1 $[M-CH_3]^+$ m/z.

2.14.3 Borylation of toluene: pinB(Me-C_6H_4)

Following the general procedure using toluene (5 cm^3) and 110°C, the product was obtained as colourless oil (130.0 mg, 0.596 mmol, 76 %). ^{1}H NMR spectral data match literature values as a mixture of isomers (o: 0.07, m: 1.00, p: 0.42).[17-18] **^{1}H NMR (CDCl$_3$, 400 MHz, 298 K):** (*meta*- isomer) δ = 7.56 (s, 1H, Ar), 7.55-7.50 (m, 1H, Ar), 7.21-7.18 (m, 2H, Ar), 2.28 (s, 3H, $ArCH_3$), 1.27 (s, 12H, $BpinCH_3$). (*para*-isomer) δ = 7.63 (d, 2H, *J = 7.90 Hz, Ar), 7.11 (*d, 2H, *J* = 7.49 Hz, Ar), 2.29 (s, 3H, $ArCH_3$), 1.26 (s, 12H, $BpinCH_3$). **^{11}B NMR (128 MHz, CDCl$_3$, 298 K):** δ = 31.0 (s). **MS (EI/MS):** Calcd. for $C_{13}H_{19}BO_2^+$: 218.1 $[M]^+$, Found: 218.1 $[M]^+$ and 203.1 $[M-CH_3]^+$ m/z.

2.14.4 Borylation of mesitylene: pinBCH$_2$(3,5-Me$_2$)C$_6$H$_3$

Using the general conditions, [Rh(COE)(SIPr)Ind] (13.7 mg, 1.91 µmol) and B_2pin_2 (210.6 mg, 0.829 mmol) were dissolved in mesitylene (330 µL, 2.49 mmol) and stirred at 150 °C for 72 h. The purified product was collected via column chromatography using pet ether 40-60°C:ethyl acetate (6.5:1) as the eluting agent, giving product pinBCH$_2$(3,5-Me$_2$)C$_6$H$_3$ as a colourless oil (69.3 mg, 0.282 mmol 34%). **^{1}H NMR (CDCl$_3$, 400 MHz, 298 K):** δ = 6.80 (s, 2H, Ar), 6.77 (s, 1H, Ar), 2.26 (s, 6H, $ArCH_3$), 2.17 (s, 2H, $ArCH_2Bpin$), 1.24 (s, 12H, $BpinCH_3$).[19] **^{11}B{^{1}H} NMR (CDCl$_3$, 96.3 MHz, 298 K):** δ = 32.88. **HRMS (ASAP/Orbitrap):** Calcd. for $C_{15}H_{24}{}^{10}BO_2^+$: 246.1900, $[M+H]^+$, Found: 246.1897 m/z. Also present was the coupled product; calcd. for $C_{24}H_{34}{}^{10}BO_2^+$: 364.2683, $[M+H]^+$, Found: 364.2676 m/z.

2.14.5 Borylation of biphenyl: pinB(Ph-C_6H_4)

[Rh(Ind)(SIPr)(COE)] (12.7 mg, 17.7 µmol), B_2pin_2 (210 mg, 0.827 mmol) and biphenyl (250 mg, 1.62 mmol) were stirred at 110 °C for 72 h. The purified product was collected via column chromatography using CH_2Cl_2 as the eluting agent, giving product pinB(Ph-C_6H_4) as a colourless oil (95.8 mg, 0.342 mmol, 41%). **^{1}H NMR ($CDCl_3$, 400 MHz, 298 K):** (*meta*- isomer) δ = 7.98 (s, 1H, Ar), 7.72 (dt, 1H *J* = 7.33, 1.16 Hz, Ar), 7.62 (ddd, 1H, *J* = 7.78, 1.16, 1.16 Hz, Ar), 7.57-7.54 (m, 2H, Ar), 7.41-7.34 (m, 3H, Ar), 7.34-7.29 (m, 1H, Ar), 1.29 (s, 12H, BpinCH_3). (*para*- isomer) δ = 7.83-7.79 (m, 2H, Ar), 7.58-7.55 (m, 4H, Ar), 7.53-7.51 (m, 2H, Ar), 7.27-7.23 (m, 1H, Ar), 1.29 (s, 12H, BpinCH_3). **^{11}B{^{1}H} NMR ($CDCl_3$, 96.3 MHz, 298 K):** δ = 31.54. Spectral data match literature values as a mixture of isomers (o: 0, m: 1.00, p: 0.63).[20] **HRMS (ASAP/Orbitrap):** Calcd. for $C_{18}H_{22}{}^{10}BO_2^+$: 280.1744 [M+H]$^+$, Found: 280.1742 m/z.

2.14.6 Borylation of anisole: pinB(OMe-C_6H_4)

[Rh(Ind)(SIPr)(COE)] (12.2 mg, 17.0 µmol), B_2pin_2 (200 mg, 0.788 mmol) and dry anisole (3 cm^3) were stirred at 110 °C for 72 h. The purified product was collected via column chromatography using CH_2Cl_2 as the eluting agent, giving product pinB(OMe-C_6H_4) as a colourless oil (97.3 mg, 0.42 mmol, 53%). **^{1}H NMR ($CDCl_3$, 400 MHz, 298 K):** (*meta*- isomer) δ = 7.44 (d, 1H, *J* = 7.26 Hz, Ar), 7.36 (s, 1H, Ar), 7.05 (m, 1H, Ar), 7.00-6.87 (m, 1H, Ar), 3.87 (s, 3H, OMe), 1.38 (s, 12H, BpinCH_3). (*para*- isomer) δ = 7.79 (d, 2H, *J* = 7.57 Hz, Ar), 6.93 (d, 2H, *J* = 7.57 Hz, Ar), 3.82 (s, 3H, OMe), 1.37 (s, 12H, BpinCH_3). **^{11}B{^{1}H} NMR ($CDCl_3$, 96.3 MHz, 298 K):** δ = 30.86. Spectral data match literature values as a mixture of isomers (o: 0.06, m: 1.00, p: 0.35).[21] **HRMS (ASAP/TOF):** Calcd. for $C_{13}H_{19}{}^{10}BO_3^+$: 233.1464 [M]$^+$, Found: 233.1459 m/z.

2.14.7 Borylation of diphenylether: pinB(OPh-C_6H_4)

[Rh(Ind)(SIPr)(COE)] (11.5 mg, 1.60 µmol), B_2pin_2 (209 mg, 0.824 mmol) and diphenylether (249 µL, 1.58 mmol) were stirred at 110 °C for 72 h. The purified product was collected via column chromatography using pet ether 40-60°C:ethyl acetate (19:1) as the eluting agent, giving pinB(OPh-C_6H_4) as a colourless oil (181 mg,0.61 mmol, 74%). **^{1}H NMR ($CDCl_3$, 400 MHz, 298 K):** δ = 7.71 (d, 1H, *J* = 8.53 Hz, Ar, *para*- isomer), 7.49 (dt, 1H, *J* = 7.32, 1.12 Hz, Ar, *meta*- isomer), 7.42-7.40 (m, 1H, Ar, *para*- isomer), 7.30-7.22 (m, 8H, Ar), 7.06-7.00 (m, 7H, Ar). **^{11}B{^{1}H} NMR ($CDCl_3$, 96.3 MHz, 298 K):** δ = 30.82. The spectral data were complicated, but matched literature values as a mixture of isomers (o: 0, m: 1.00, p: 0.26).[20] **HRMS (ASAP/Orbitrap):** Calcd. for $C_{18}H_{22}{}^{10}BO_3^+$: 296.1693 [M+H]$^+$, Found: 296.1690 m/z.

2.14.8 Borylation of fluorobenzene: pinB(F-C_6H_4)

Following the general procedure using fluorobenzene (5 cm^3), the product was obtained as colourless oil (58.1 mg, 0.262 mmol, 33 %). ^{1}H NMR spectral data match literature values as a mixture of isomers (o: 0.91, m: 1, p: 0.18).[17] **^{11}B NMR (128 MHz, $CDCl_3$, 298 K):** δ = 30.5 (s). **^{19}F NMR ($CDCl_3$, 377 MHz, 298 K):** δ = -102.63 (dt, J = 9.2, 5.9 Hz, ortho), -108.46 (tt, J = 9.2, 6.2 Hz, para), -114.2 (tt, J = 13.6, 5.6 Hz, meta). MS (EI/MS): Calcd. for $C_{12}H_{16}BFO_2^+$: 222.1 [M]$^+$, Found: 222.1 [M]$^+$ and 207.1 [M-CH_3]$^+$ m/z.

2.14.9 Borylation of octane: octylBpin

In a glovebox, B_2pin_2 (121 mg, 0.476 mmol) and [Rh(Ind)(SIPr)(COE)] (17.2 mg, 0.024 mmol, 5 mol%) were dissolved in octane (5 cm^3) and added to a J. Young tapped flask. The reaction mixture was heated at 130 °C and left for 48 hours. The reaction was concentrated in vacuo and the residue extracted with CH_2Cl_2 (2 x 5 cm^3). All volatile were then removed in vacuo to give the crude mixture. The product was purified by column chromatography (SiO_2, CH_2Cl_2) affording the product as a colourless oil (8 mg, 0.033 mmol, 7 %). **^{1}H NMR (400 MHz, CDCl$_3$, 298 K):** δ = 1.40-1.20 (m, 24H), 0.87 (t, 3H, J = 6.9 Hz, C*H*$_3$CH$_2$), 0.76 (t, 2H, J = 7.7 Hz, C*H*$_2$Bpin). **^{13}C{^{1}H) NMR (101 MHz, CDCl$_3$, 298 K):** δ = 83.0 (O*C*Me$_2$), 32.6, 32.0, 29.6, 29.4, 24.9(C*Me*$_2$), 24.2, 22.8, 14.3 (CH$_3$CH$_2$), 11.5 (br, CH2B). **^{11}B{^{1}H} NMR** (128 MHz, CDCl$_3$, 298 K): δ = 34.27. The ^{1}H and ^{11}B NMR data match those reported in the literature.[22] **HRMS (ASAP/TOF):** Calcd. for $C_{14}H_{30}{}^{10}BO_2{}^+$: 240.2375, [M+H]$^+$, Found: 240.2375 m/z. Also, diborylated product present: pinBC$_8$H$_{16}$Bpin. HRMS (ASAP/TOF): Calcd. for $C_{20}H_{41}{}^{10}BO_4{}^+$: 365.3264, [M+H]$^+$, Found: 365.3260 m/z.

2.14.10 Borylation of decane: decylBPin

In a glovebox, B_2pin_2 (121 mg, 0.476 mmol) and [Rh(Ind)(SIPr)(COE)] (34.4 mg, 0.0479 mmol, 10 mol%) were dissolved in decane (5 cm^3) and added to a J. Young tapped flask. The reaction mixture was heated at 150 °C and left for 48 hours. The reaction was concentrated in vacuo and the residue extracted with CH_2Cl_2 (2 x 5 cm^3). All volatile were then removed in vacuo to give the crude mixture. The product was purified by column chromatography (SiO_2, CH_2Cl_2) affording the product as a colourless oil (23 mg, 0.086 mmol, 18 %). **^{1}H NMR (400 MHz, CDCl$_3$, 298 K):** δ = 1.44-1.13 (m, 28 H), 0.87 (t, 3 H, J = 6.9 Hz, C*H*$_3$CH$_2$), 0.76 (t, 2H, J = 7.8 Hz, C*H*$_2$Bpin). **^{13}C{^{1}H) NMR (101 MHz, CDCl$_3$, 298 K):** δ = 83.0 (OCMe$_2$), 32.6, 32.1, 29.8, 29.7, 29.6, 29.5, 24.9 (CMe$_2$), 24.2, 22.8, 14.3 (CH$_3$CH$_2$), 11.3 (br, CH$_2$B). **^{11}B{^{1}H} NMR (128 MHz, CDCl$_3$, 298 K):** δ = 34.27. **HRMS (ASAP/TOF):** Calcd. for $C_{16}H_{34}{}^{10}BO_2{}^+$: 268.2688, [M+H]$^+$, Found: 268.2688 m/z. Also, diborylation product present: pinBC$_{10}$H$_{20}$Bpin. **HRMS (ASAP/TOF):** Calcd. for $C_{22}H_{45}{}^{10}BO_4{}^+$: 394.3540, [M+H]$^+$, Found: 394.3539 m/z.

2.14.11 Borylation of cholestane

[Rh(Ind)(SIPr)(COE)] (21.7 mg, 3.01 x10^{-5} mol), B_2pin_2 (85 mg, 0.335 mmol) and 5α-cholestane (125.7 mg, 0.337 mmol) were added to a young's flask and stirred at 150 °C for 168 h. Due to the complexity of the ^{1}H NMR spectra, product identification was not possible. ^{11}B{^{1}H} NMR (CDCl$_3$, 96.3 MHz, 298 K): δ = 34.35. HRMS (ASAP/Orbitrap): Calcd. for $C_{33}H_{60}{}^{10}BO_2{}^+$: 498.4717 [cholestylBpin+H]$^+$, Found: 498.4703 m/z. Also present is the diborylated product: Calcd. for $C_{39}H_{71}{}^{10}BO_4{}^+$: 623.5606 [cholestyl(Bpin)$_2$+H]$^+$, Found: 623.5600 m/z.

2.15 Borylation with other complexes (NHC = IMes and SIMes, and tethered ligands)

2.15.1 Borylation of benzene with [Rh(Ind)(IMes)(COE)]: PhBpin

In the glovebox, [Rh(Ind)(IMes)(COE)] (2.5 mg, 4.0 µmol, 5 mol%), ferrocene (1.4 mg, 7.66 µmol) and B_2pin_2 (19.5 mg, 76.7 µmol) were combined in C_6H_6/C_6D_6 (0.7 mL) and added to an NMR tube with a J. Young's valve; the sample was then heated at 75°C. **^{1}H NMR (400 MHz, C_6D_6, 298 K):** δ = 8.17-8.12 (m, 2H, ortho ArH), 7.46 (m, 1H, para ArH), 7.36 (m, 2H, meta ArH), 1.35 (s, 12H, Me). **^{11}B{^{1}H} NMR (128 MHz, C_6D_6, 298 K):** δ = 31.43 (s).

2.15.2 Borylation of benzene with [Rh(Ind)(SIMes)(COE)]: PhBpin

In the glovebox, [Rh(Ind)(SIMes)(COE)] (2.5 mg, 4.0 µmol, 5 mol%), ferrocene (1.4 mg, 7.66 µmol) and B_2pin_2 (19.5 mg, 76.7 µmol) were combined in C_6H_6/C_6D_6 (0.7 mL) and added to an NMR tube with a J. Young's valve; the sample was then heated at 75°C. **^{1}H NMR (400 MHz, C_6D_6, 298 K):** δ = 8.17-8.12 (m, 2H, ortho ArH), 7.46 (m, 1H, para ArH), 7.36 (m, 2H, meta ArH), 1.35 (s, 12H, Me). **^{11}B{^{1}H} NMR (128 MHz, C_6D_6, 298 K):** δ = 31.43 (s).

2.15.3 Borylation of benzene with [Rh(Flu-Mes)(COE)]: PhBpin

In the glovebox, [Rh(Flu-Mes)(COE)] (2.5 mg, 4.0 µmol, 5 mol%), ferrocene (1.4 mg, 7.66 µmol) and B_2pin_2 (19.5 mg, 76.7 µmol) were combined in C_6H_6/C_6D_6 (0.7 mL) and added to an NMR tube with a J. Young's valve; the sample was then heated at 75°C. **^{1}H NMR (400 MHz, C_6D_6, 298 K):** δ = 8.17-8.12 (m, 2H, ortho ArH), 7.46 (m, 1H, para ArH), 7.36 (m, 2H, meta ArH), 1.35 (s, 12H, Me). **^{11}B{^{1}H} NMR (128 MHz, C_6D_6, 298 K):** δ = 31.43 (s).

2.15.4 Borylation of benzene with [Rh(Flu-Me)(COE)]: PhBpin

In the glovebox, [Rh(Flu-Me)(COE)] (2.5 mg, 0.00514 mmol), ferrocene (1.9 mg, 10.3 µmol) and B_2pin_2 (26.1 mg, 0.1028 mmol) were combined in C_6H_6/C_6D_6 (0.7 mL) and added to an NMR tube with a J. Young's valve; the sample was then heated at 75°C. **^{1}H NMR (400 MHz, C_6D_6, 298 K):** δ = 8.17-8.12 (m, 2H, ortho ArH), 7.46 (m, 1H, para ArH), 7.36 (m, 2H, meta ArH), 1.35 (s, 12H, Me). **^{11}B{^{1}H} NMR (128 MHz, C_6D_6, 298 K):** δ = 31.43 (s).

2.15.5 Borylation of decane with [Rh(Ind)(IMes)(COE)]: DecylBpin

In the glovebox, [Rh(Ind)(IMes)(COE)] (2.5 mg, 4.0 µmol, 5 mol%), and B_2pin_2 (19.5 mg, 76.7 µmol) were combined in decane (0.7 mL) and added to an NMR tube with a J. Young's valve; the sample was then heated at 140°C. **^{11}B{^{1}H} NMR (128 MHz, decane, 298 K):** δ = 28.63 (s).

2.15.6 Borylation of octane with [Rh(Ind)(IMes)(COE)]: OctylBpin

In the glovebox, [Rh(Ind)(IMes)(COE)] (2.5 mg, 4.0 µmol, 5 mol%) and B_2pin_2 (19.5 mg, 76.7 µmol) were combined in octane (0.7 mL) and added to an NMR tube with a J. Young's valve; the sample was then heated at 120°C. **^{11}B{^{1}H} NMR (128 MHz, octane, 298 K):** δ = 30.68 (s).

2.16 General procedure for the photolysis of Rh complexes

Example: A solution of **3** (15.0 mg, 0.0209 mmol) in C_6D_6 (0.5 cm^3) was added to an NMR tube equipped with a J. Young's tap. The tube was suspended adjacent to a blue LED (Method A: 400 nm at 5 W or B: 420 nm at 33 W) at a distance of 3 cm. An appropriately-sized box lined with aluminium foil was used to contain the set-up. The duration of photolysis was initially 5 mins for A and 30 s for B. The reaction was followed by ^{1}H NMR spectroscopy.

2.17 General procedure for NMR scale reactions

Example: In the glovebox, **3** (15.0 mg, 0.0209 mmol) was dissolved in C_6D_6 (0.5cm^3) and added to an NMR tube equipped with a J. Young's tap. To the sample, HBcat (1-3 eq.) was added and the reaction was monitored by ^{1}H and ^{11}B NMR spectroscopy.

3 Additional molecular structures determined by single crystal X-ray diffraction

3.1 Crystallographic details

Single crystals suitable for X-ray diffraction were covered in inert oil and placed under the cold stream of a Bruker D8 Venture at 100 K or an Oxford Diffraction four-circle Super nova diffractometer (University of Edinburgh) at 120 K (**1**). Exposures were collected using Mo-K_α radiation (λ =0.71073) or Cu- K_α radiation (λ = 1.54178). Indexing, data collection and absorption corrections were performed. The structures were then solved using SHELXT[23] and refined by full-matrix least-squares refinement (SHELXL)[23] interfaced with the programme OLEX2.[24] The backbone of several cyclooctene ligands (**3, 4**, **5**, **7**, **14**, **[Ir(Ind)(COE)$_2$]**) were modelled successfully over two positions. The structure of [Rh(SIPr)(μ-Bcat)$_2$(μ-B,O-Bcat)Rh(H)(SiPr)] had a disordered O,B-bridging Bcat ligand (0.69:0.31 occupancies), and the presence of a disordered hydride ligand best fit the data and anticipated bonding model for two Rh atoms connected by a Rh-Rh bond (H atom was freely refined with a constrained U_{iso} and the same occupancies as the disordered Bcat ligands). [Ir(Ind)(COE)$_2$] featured a large Q peak above the indenyl ring which could not be successfully modelled. Whilst we could not spot the obvious presence of twinning, a small twinned component might explain this large residual electron density. The data for **15** were of poor quality because the crystal did not diffract well, especially at large angle. However, the data were good enough to unambiguously established connectivity. CCDC deposition numbers: 2091749-2091765.

3.2 Structures of monodentate complexes

3.2.1 [Rh(Ind)(SIPr)(C_2H_4)] (1)

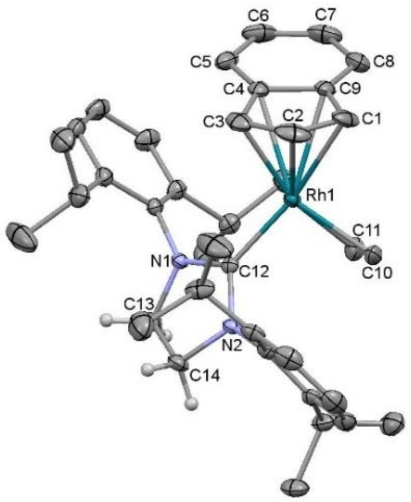

Figure S1. Molecular structure of [Rh(Ind)(SIPr)(C_2H_4)] (**1**) with thermal ellipsoids at 50% probability. All H atoms except for those on the NHC backbone have been removed for clarity.

3.2.2 [Rh(Ind)(SIPr)(COE)] (3): two molecules in the asymmetric unit

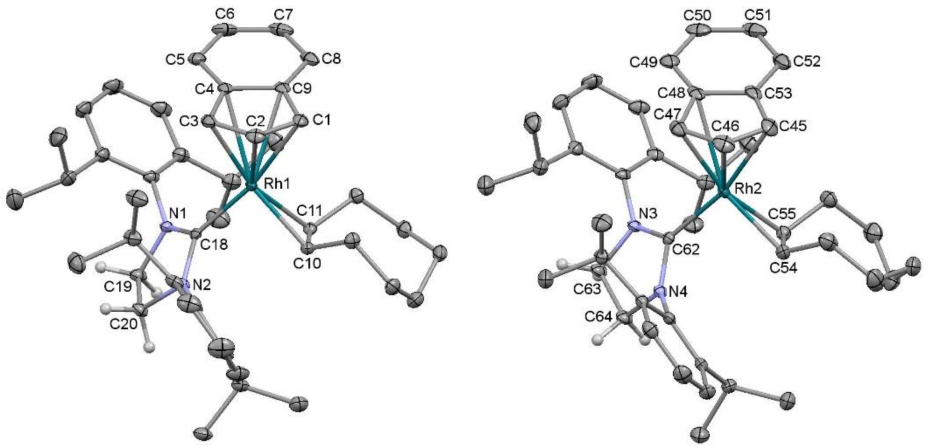

Figure S2. Comparison of the two molecules in the asymmetric unit for [Rh(Ind)(SIPr)(COE)] (**3**). Thermal ellipsoids at 50% probability and all H atoms, except those on the NHC backbone, have been removed for clarity.

3.2.3 [Rh(Ind)(SIMes)(COE)] (4)

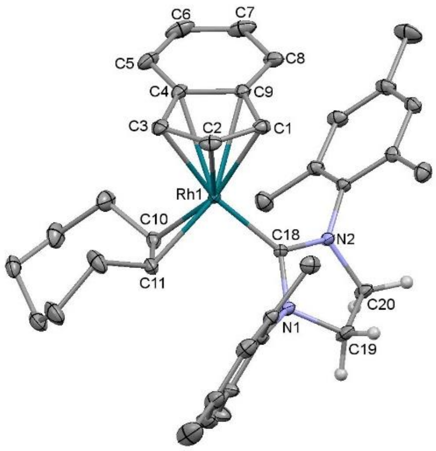

Figure S3. Molecular structure of [Rh(Ind)(SIMes)(COE)] with thermal ellipsoids at 50% probability. All H atoms, except those on the NHC backbone, have been removed for clarity.

3.2.4 [Rh(Ind)(IMes)(COE)] (5): two molecules in the asymmetric unit

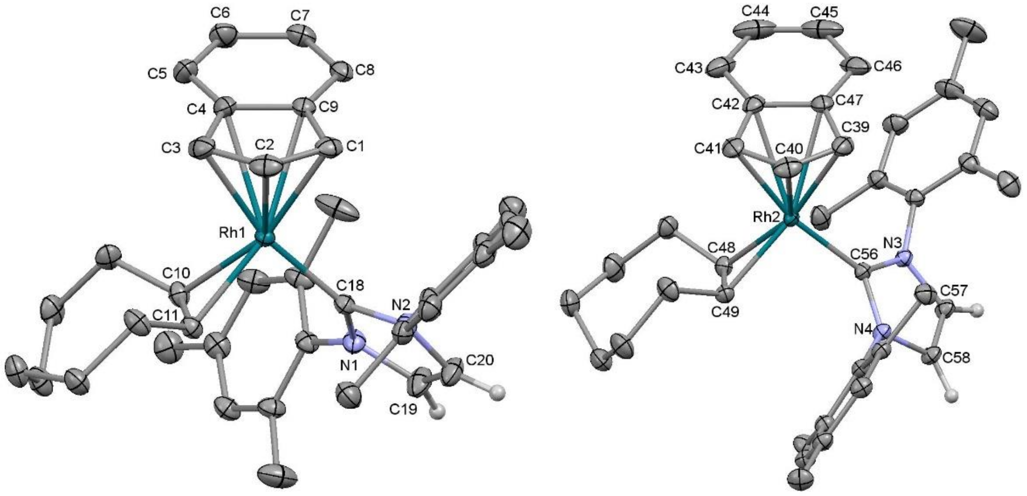

Figure S4. Comparison of the two molecules in the asymmetric unit for [Rh(Ind)(IMes)(COE)] (**5**). Thermal ellipsoids at 50% probability and all H atoms, except those on the NHC backbone, have been removed for clarity.

3.2.5 [{Rh(Cp*)(μ:η^1,η^2-CH=CH_2)}$_2$] (10)

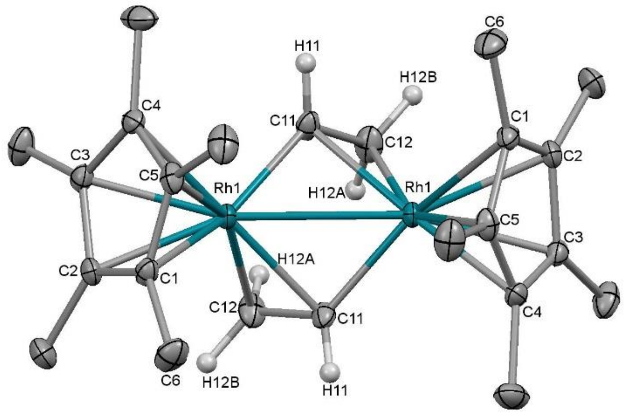

Figure S5. Molecular structure of [{Rh(Cp*)(μ:η^1,η^2-CH=CH_2)}$_2$] (**10**) with thermal ellipsoids at 50% probability. All H atoms, except those on the vinyl ligand, have been removed for clarity. Selected bond lengths (Å): Rh1-Rh1′ 2.6946(5), Rh1-C1 2.272(3), Rh1-C2 2.208(3), Rh1-C3 2.178(3), Rh1-C4 2.195(3), Rh1-C5 2.266(3), Rh1-C11 (σ-bond) 2.001(3), Rh1-C11′ (π-bond) 2.120(3), Rh1-C12′ (π-bond) 2.157(3), C11-Rh1′ 2.120(3), C11-C12 1.410(4), C12-Rh11 2.157(3), Rh-Rh′ 2.695. Symmetry operator for symmetry generated atoms: 1-X, + Y, 3/2 – Z.

3.2.6 [Ir(Ind)(COE)$_2$]

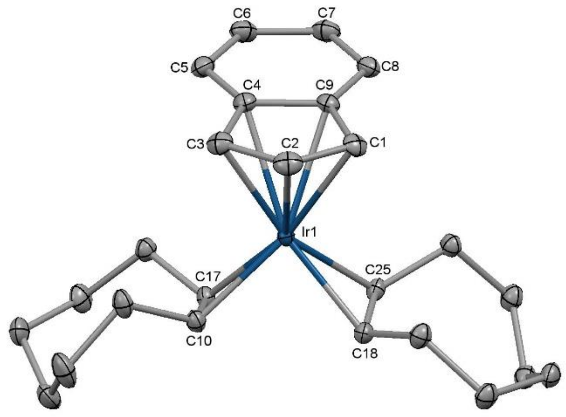

Figure S6. Molecular structure of [Ir(Ind)(COE)$_2$] with thermal ellipsoids at 50% probability. All H atoms have been removed for clarity. Selected bond lengths (Å): Ir1-C1 2.229(3), Ir1-C2 2.225(3), Ir1-C3 2.235(3), Ir1-C4 2.406(2), Ir1-C9 2.397(2), Ir1-C10 2.123(2), Ir1-C17 2.127(2), Ir1-C18 2.131(2), Ir1-C25 2.140(2), C10-C17 1.426(3), C18-C25 1.435(3).

3.2.7 [Rh(SIPr)(μ-Bcat)$_2$(μ-B,O-Bcat)Rh(H)(SiPr)]

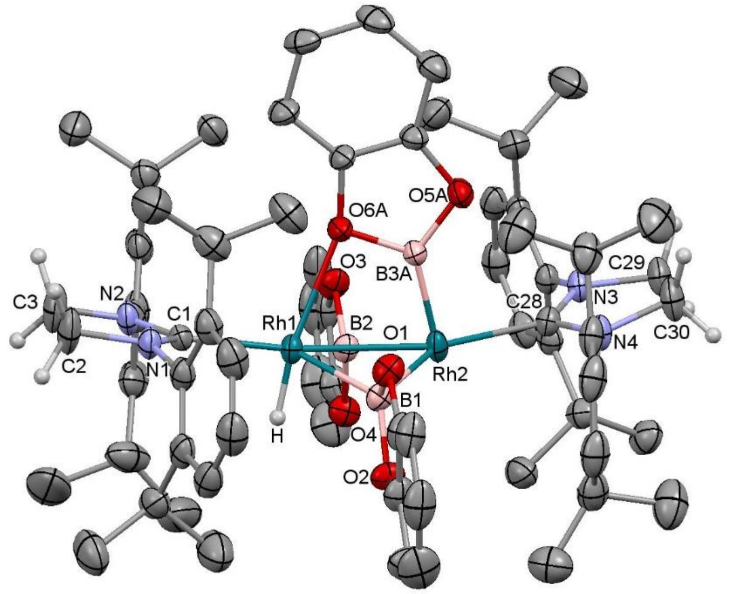

Figure S7. Molecular structure of [Rh(SIPr)(μ-Bcat)$_2$(μ-B,O-Bcat)Rh(H)(SiPr)]. Thermal ellipsoids are at 50% probability and all H atoms except for those on the NHC backbone have been removed for clarity.

3.3 Structures of fluorenyl-tethered complexes

3.3.1 Molecular structure of [Rh(Flu-Dipp)(C_2H_4)] (11)

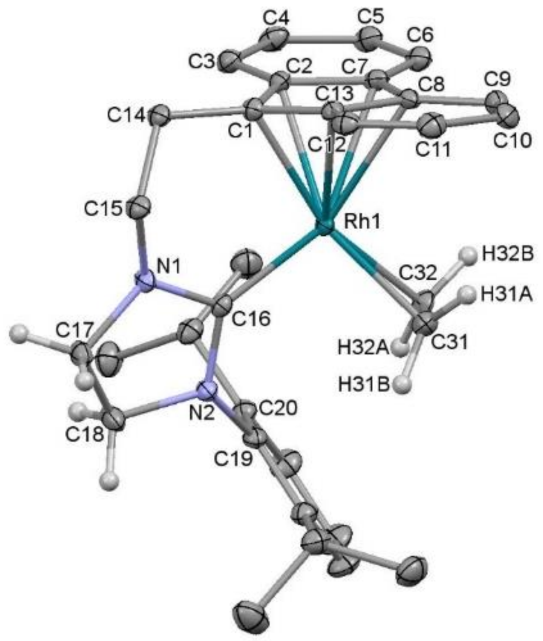

Figure S8. Molecular structure of [Rh(Flu-Dipp)(C_2H_4)] (**11**). Thermal ellipsoids are at 50% probability and all H atoms except for those on the NHC backbone and on the C_2H_4 ligand have been removed for clarity.

3.3.2 Molecular structure of [Rh(Flu-Dipp)(CO)] (13)

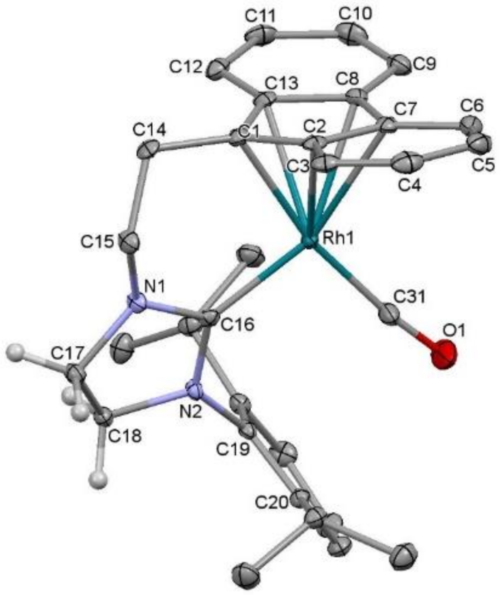

Figure S9. Molecular structure of [Rh(Flu-Dipp)(CO)] (**13**). Thermal ellipsoids are at 50% probability and all H atoms except for those on the NHC backbone have been removed for clarity.

3.3.3 Molecular structure of [Rh(Flu-Mes)(CO)] (15)

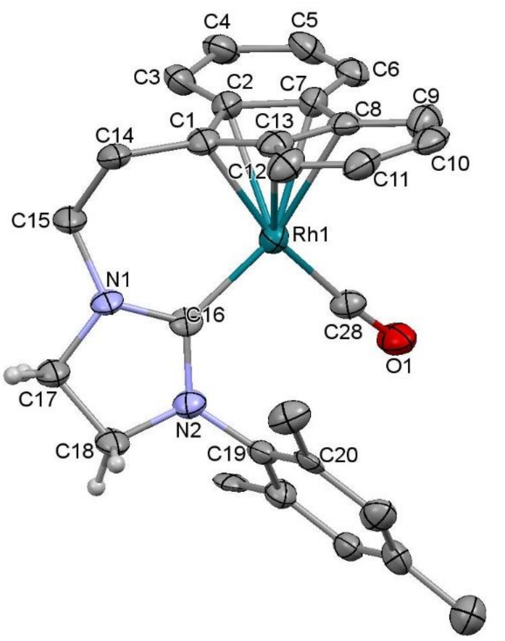

Figure S10. Molecular structure of [Rh(Flu-Mes)(CO)] (**15**). Thermal ellipsoids at 50% probability and all H atoms, except those on the NHC backbone, have been removed for clarity

3.3.4 [Rh(Flu-Me)(CO)] (17): two molecules in the asymmetric unit

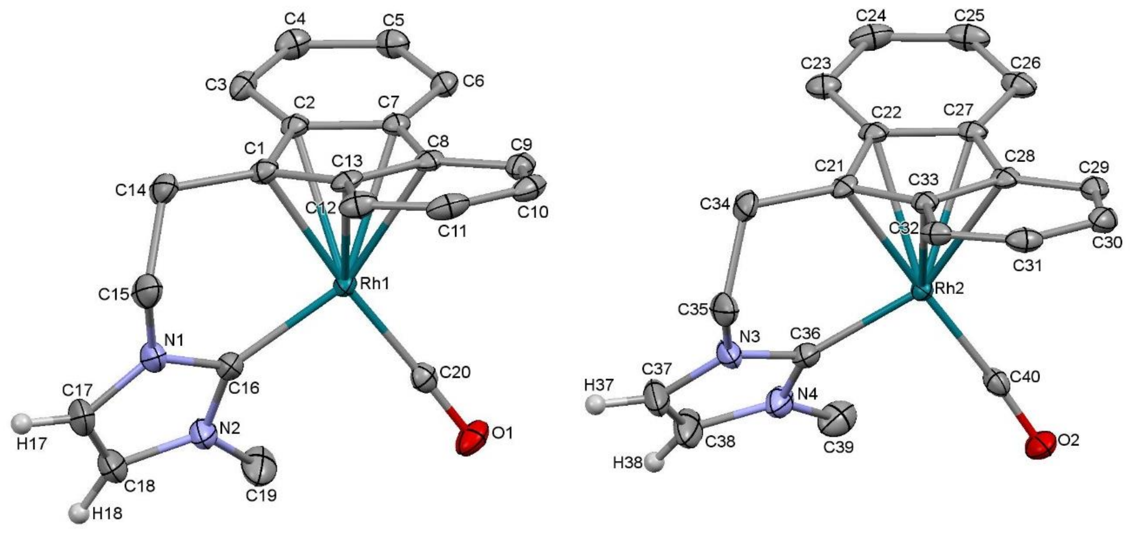

Figure S11. Comparison of the two molecules in the asymmetric unit for [Rh(Flu-Me)(CO)] (**17**). Thermal ellipsoids at 50% probability and all H atoms, except those on the NHC backbone, have been removed for clarity.

3.4 Comparsion of bond lengths and angles for monodentate and fluorenyl-tethered complexes

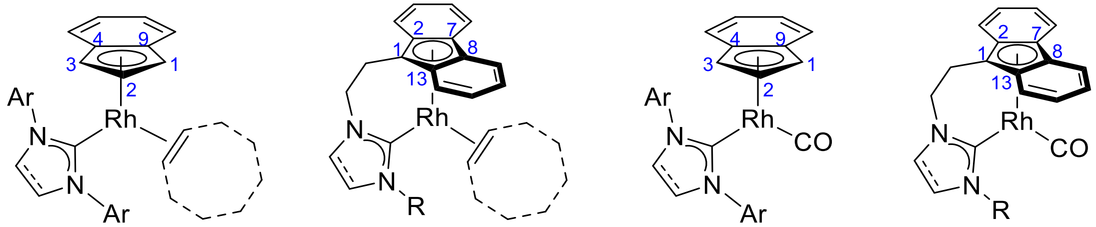

Table S1. Comparison of bond lengths (Å) for the piano-stool rhodium complexes.

**Compound**	**C1**	**C2**	**C3**	**C4**	**C9**	**Fold angle /°**	**Rh-NHC**	**M-C$_{COE}$**		**C=C (COE)**
Ir(Ind)(COE)$_2$	2.229(3)	2.225(3)	2.235(3)	2.406(2)	2.397(2)	7.7		2.123(2)	2.127(2)	1.426(3)
								2.131(2)	2.140(2)	1.435(3)
Rh(Ind)(COE)$_2$ [10]	2.230(2)	2.245(2)	2.2385(19)	2.4148(17)	2.4126(18)	9.0		2.1495(19)	2.1513(19)	1.405(3)
								2.1520(19)	2.1659(19)	1.408(3)
Rh(Ind)(SIPr)(COE)	2.2955(16)	2.2034(16)	2.2310(16)	2.5277(16)	2.5674(16)	10.7	1.9881(15)	2.1161(16)	2.1401(16)	1.409(2)
	2.2803(17)	2.1968(17)	2.2271(18)	2.5572(17)	2.5806(17)		1.9946(15)	2.1376(17)	2.1370(17)	1.407(2)
Rh(Ind)(SIMes)(COE)	2.2148(7)	2.2162(7)	2.2833(7)	2.5209(7)	2.4857(7)	9.8	1.9877(6)	2.1331(7)	2.1237(7)	1.4109(11)
Rh(Ind)(IMes)(COE)	2.2566(10)	2.2331(11)	2.2540(10)	2.4720(10)	2.4683(10)	9.3	2.0169(10)	2.1302(10)	2.1253(10)	1.4156(14)
	2.2293(11)	2.2109(11)	2.2547(10)	2.4985(10)	2.4765(10)	8.8	2.0124(9)	2.1371(9)	2.1143(10)	1.4169(13)
**Compound**	**C8**	**C13**	**C1**	**C2**	**C7**		**Rh-NHC**	**M-C$_{alkene}$**		**C=C (alkene)**
Rh(Flu-Mes)(COE)	2.4298(14)	2.3361(14)	2.1654(15)	2.3569(14)	2.4693(15)		1.9808(15)	2.1473(14)	2.1711(14)	1.404(2)
Rh(Flu-Dipp)(C_2H_4)	2.366(3)	2.292(3)	2.160(3)	2.335(3)	2.397(2)		1.967(3)	2.118(3)	2.168(3)	1.403(4)
**Compound**	**C1**	**C2**	**C3**	**C4**	**C9**		**Rh-NHC**	**M-C$_{ethene}$**		**C=C (C_2H_4)**
Rh(Ind)(C_2H_4)$_2$ [25] Mol. A:	2.176(8)	2.215(8)	2.206(7)	2.358(7)	2.362(7)	9.3		2.164(8)	2.147(8)	1.387(13)

								2.119(9)	2.134(8)	1.368(13)
Mol. B:	2.182(8)	2.224(8)	2.214(7)	2.355(7)	2.352(7)	8.6		2.148(7)	2.147(8)	1.387(12)
								2.142(8)	2.155(8)	1.366(14)
Rh(Ind)(SIPr)(C_2H_4)	2.2717(15)	2.2125(16)	2.2109(15)	2.4929(14)	2.5035(14)	9.8	1.9886(12)	2.1197(14)	2.1205(15)	1.399(2)
Rh(Ind){Si(OEt_3)}(H)(SIPr)	2.340(3)	2.248(3)	2.220(3)	2.462(3)	2.552(3)	8.8	2.014(3)			
**Compound**	**C1**	**C2**	**C3**	**C4**	**C9**		**Rh-NHC**	**M-C_{CO}**		**C≡O**
Rh(Ind)(SIPr)(CO)	2.2485(17)	2.2209(19)	2.2358(18)	2.4854(16)	2.4762(16)	9.7	2.0103(14)	1.8277(19)		1.154(2)
**Compound**	**C8**	**C13**	**C1**	**C2**	**C7**		**Rh-NHC**	**M-C_{CO}**		**C≡O**
Rh(Flu-Dipp)(CO)	2.375(3)	2.332(3)	2.185(3)	2.342(3)	2.377(3)		1.987(2)	1.846(3)		1.147(4)
Rh(Flu-Mes)(CO)	2.390(12)	2.348(14)	2.158(13)	2.331(13)	2.353(13)		1.997(14)	1.846(14)		1.141(15)
Rh(Flu-Me)(CO)	2.346(3)	2.326(3)	2.189(3)	2.364(3)	2.391(3)		2.001(3)	1.841(3)		1.150(4)
	2.329(3)	2.322(3)	2.195(3)	2.378(3)	2.405(3)		1.990(3)	1.833(3)		1.156(4)

3.5 Molecular structures of dimeric complexes

3.5.1 [Rh(μ-Cl)(SIPr)(COE)]$_2$ (7)

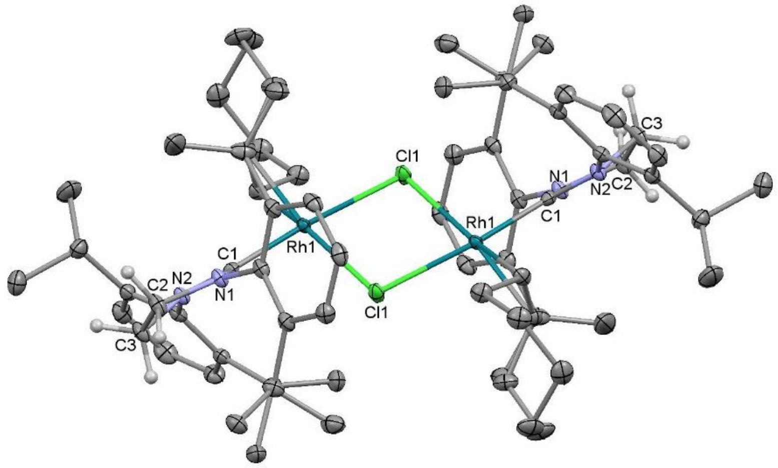

Figure S12. Molecular structure of [Rh(μCl)(SIPr)(COE)]$_2$ (**7**). Thermal ellipsoids at 50% probability and all H atoms, except those on the NHC backbone, have been removed for clarity.

3.5.2 [Rh(μ-Cl)(SIMes)(COE)]$_2$ (8)

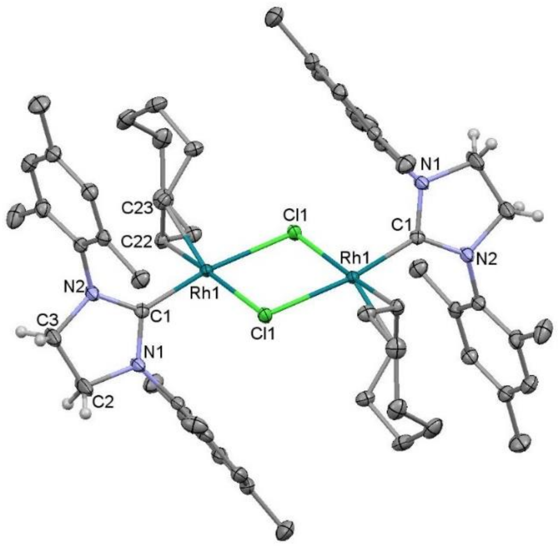

Figure S13. Molecular structures of [Rh(μCl)(SIMes)(COE)]$_2$ (**8**). Thermal ellipsoids are at 50% probability and all H atoms, except for those on the NHC backbone, have been removed for clarity.

3.5.3 [Rh(μ-OH)(SIPr)(COE)]$_2$ (9)

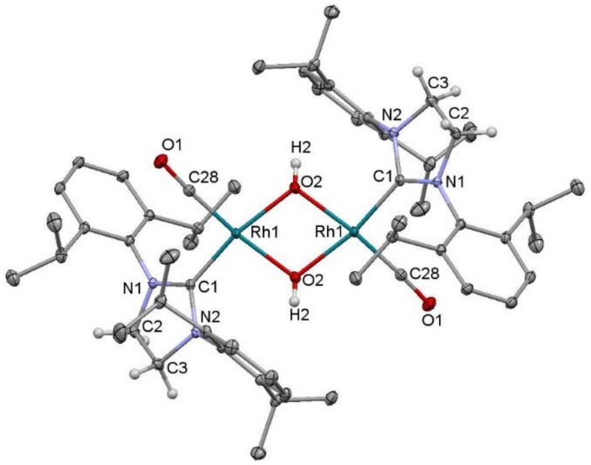

Figure S14. Molecular structures of [Rh(μ-OH)(SIPr)(COE)]$_2$ (**9**). Thermal ellipsoids are at 50% probability and all H atoms, except for the OH and those on the NHC backbone, have been removed for clarity.

3.6 Comparison of bond lengths for the dimeric complexes

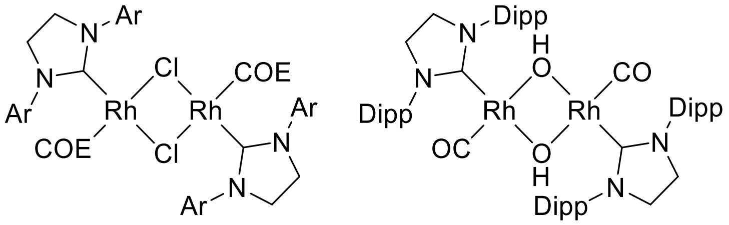

Table S2. Comparison of bond lengths (Å) for the chloride and hydroxide bridged dimers.

**Compound**	**Rh-NHC**	**Rh-Cl**	**Rh-C**	**C=C$_{COE}$**	**Rh…Rh**
[Rh(μ-Cl)(SIMes)(COE)]$_2$	1.9572(15)	2.3990(4)*	2.1302(15)	1.416(2)	3.731
		2.3991(4)*	2.1045(15)		
		2.4637(4)			
[Rh(μ-Cl)(SIPr)(COE)]$_2$	1.9504(12)	2.3911(3)	2.1067(12)	1.4071(18)	3.756
		2.4621(3)*	2.1333(12)		
		2.4621(3)*			
**Compound**	**Rh-NHC**	**Rh-OH**	**Rh-C**	**CO**	
[Rh(μ-OH)(SIPr)(CO)]$_2$	1.961(2)	2.0728(16)	1.792(2)	1.156(2)	3.244
		2.0918(16)*			
		2.0919(16)*			

* One of the atoms are symmetry generated.

3.7 Crystallographic tables of data

Table S3. Additional crystallographic data.

	[Rh(Ind)(SIPr)(C_2H_4)] (**1**)	[Rh(Ind)(SIPr)(CO)] (**2a**)	[Rh(Ind)(SIPr)(COE)] (**3**)
Empirical formula	$C_{38}H_{49}N_2Rh$	$C_{37}H_{45}N_2ORh$	$C_{44}H_{59}N_2Rh$
Formula weight	636.70	636.66	718.84
T/K	120.01(10)	100.0	100.0
Crystal system	monoclinic	Monoclinic	orthorhombic
Space group	$P2_1/c$	$P2_1/c$	Pbca
a/Å	10.04226(19)	10.0182(2)	18.4246(6)
b/Å	19.1446(3)	19.3666(5)	21.4379(7)
c/Å	17.3168(3)	17.1516(4)	37.0393(10)
α/°	90	90	90
β/°	106.327(2)	106.8980(10)	90
γ/°	90	90	90
Volume/$Å^3$	3194.98(11)	3184.05(13)	14630.0(8)
Z	4	4	16
$\rho_{calc}g/cm^3$	1.324	1.328	1.305
μ/mm^1	0.563	4.561	0.500
F(000)	1344.0	1336.0	6112.0
Crystal size/mm^3	0.394 × 0.312 × 0.215	0.42 × 0.22 × 0.18	0.5 × 0.5 × 0.05
Radiation	MoKα (λ = 0.71073)	CuKα (λ = 1.54178)	MoKα (λ = 0.71073)
2Θ range for data collection/°	5.852 to 65.942	7.06 to 144.256	5.498 to 54.996
Index ranges	-15 ≤ h ≤ 15, -28 ≤ k ≤ 28, -26 ≤ l ≤ 25	-12 ≤ h ≤ 12, -23 ≤ k ≤ 23, -19 ≤ l ≤ 21	-21 ≤ h ≤ 23, -27 ≤ k ≤ 16, -48 ≤ l ≤ 45
Reflections collected	82040	45288	73947
Independent reflections	11529 [R_{int} = 0.0519, R_{sigma} = 0.0354]	6185 [R_{int} = 0.0255, R_{sigma} = 0.0167]	16736 [R_{int} = 0.0270, R_{sigma} = 0.0227]
Data/ restraints/ parameters	11529/0/390	6185/0/378	16736/0/882
Goodness-of-fit on F^2	1.061	1.069	1.025
Final R indexes [I>=2σ (I)]	R_1 = 0.0312, wR_2 = 0.0684	R_1 = 0.0224, wR_2 = 0.0594	R_1 = 0.0270, wR_2 = 0.0599
Final R indexes [all data]	R_1 = 0.0390, wR_2 = 0.0718	R_1 = 0.0227, wR_2 = 0.0596	R_1 = 0.0358, wR_2 = 0.0634
Largest diff. peak/hole (e $Å^{-3}$)	0.58/-0.62	0.41/-0.70	0.41/-0.42

Table S3 continued. Additional crystallographic data.

	[Rh(Ind)(IMes)(COE)] (**5**)	[Rh(Ind)(SIMes)(COE)] (**4**)
Empirical formula	$C_{38}H_{45}N_2Rh$	$C_{38}H_{47}N_2Rh$
Formula weight	632.67	634.68
T/K	100.0	100.0
Crystal system	Triclinic	Triclinic
Space group	P-1	P-1
a/Å	11.3295(3)	11.1017(3)
b/Å	16.1752(4)	11.4125(3)
c/Å	18.1265(5)	14.3566(4)
α/°	74.5280(10)	68.0730(10)
β/°	86.9440(10)	84.5560(10)
γ/°	88.8310(10)	73.2020(10)
Volume/Å^3	3196.81(15)	1615.20(8)
Z	4	2
ρ_{calc}g/cm^3	1.315	1.305
μ/mm^1	0.562	0.557
F(000)	1328.0	668.0
Crystal size/mm^3	0.36 × 0.32 × 0.3	0.5 × 0.46 × 0.36
Radiation	MoKα (λ = 0.71073)	MoKα (λ = 0.71073)
2Θ range for data collection/°	5.404 to 72.626	5.854 to 72.656
Index ranges	-18 ≤ h ≤ 18, -26 ≤ k ≤ 26, -30 ≤ l ≤ 30	-18 ≤ h ≤ 18, -19 ≤ k ≤ 19, -23 ≤ l ≤ 23
Reflections collected	189403	148855
Independent reflections	30939 [R_{int} = 0.0305, R_{sigma} = 0.0230]	15592 [R_{int} = 0.0231, R_{sigma} = 0.0119]
Data/ restraints/ parameters	30939/0/807	15592/0/395
Goodness-of-fit on F^2	1.050	1.092
Final R indexes [I>=2σ (I)]	R_1 = 0.0258, wR_2 = 0.0616	R_1 = 0.0183, wR_2 = 0.0497
Final R indexes [all data]	R_1 = 0.0353, wR_2 = 0.0666	R_1 = 0.0192, wR_2 = 0.0503
Largest diff. peak/hole (e Å^{-3})	0.96/-0.78	1.01/-0.67

Table S3 continued. Additional crystallographic data.

	$[Ir(Ind)(COE)_2]$	$[\{Rh(\mu\text{-}Cl)(SIMes)(COE)\}_2]$ (**8**)	$[\{Rh(\mu\text{-}Cl)(SIPr)(COE)\}_2]$ (**7**)
Empirical formula	$C_{25}H_{35}Ir$	$C_{70}H_{92}Cl_2N_4Rh_2$	$C_{82}H_{116}Cl_2N_4Rh_2$
Formula weight	527.73	1266.19	1434.50
T/K	100.0	100.0	100.0
Crystal system	orthorhombic	Monoclinic	monoclinic
Space group	$P2_12_12_1$	C2/c	C2/c
a/Å	7.3367(2)	22.2383(5)	16.6682(7)
b/Å	13.3763(4)	12.9453(3)	21.4373(8)
c/Å	20.5843(5)	21.8918(5)	20.5528(9)
α/°	90	90	90
β/°	90	98.6520(10)	93.971(2)
γ/°	90	90	90
Volume/$Å^3$	2020.10(10)	6230.5(2)	7326.3(5)
Z	4	4	4
$\rho_{calc}g/cm^3$	1.735	1.350	1.301
μ/mm^1	6.615	0.660	4.653
F(000)	1048.0	2656.0	3040.0
Crystal size/mm^3	0.36 × 0.24 × 0.14	0.2 × 0.1 × 0.1	0.2 × 0.2 × 0.1
Radiation	MoKα (λ = 0.71073)	MoKα (λ = 0.71073)	CuKα (λ = 1.54178)
2Θ range for data collection/°	5.896 to 72.77	5.442 to 54.222	6.728 to 150.182
Index ranges	-11 ≤ h ≤ 12, -22 ≤ k ≤ 22, -34 ≤ l ≤ 34	-28 ≤ h ≤ 28, -16 ≤ k ≤ 16, -28 ≤ l ≤ 28	-20 ≤ h ≤ 20, -26 ≤ k ≤ 26, -25 ≤ l ≤ 25
Reflections collected	74245	61882	134922
Independent reflections	9806 [R_{int} = 0.0312, R_{sigma} = 0.0240]	6885 [R_{int} = 0.0334, R_{sigma} = 0.0168]	7509 [R_{int} = 0.0293, R_{sigma} = 0.0106]
Data/ restraints/ parameters	9806/3/254	6885/0/358	7509/0/469
Goodness-of-fit on F^2	1.039	1.031	1.047
Final R indexes [I>=2σ (I)]	R_1 = 0.0156, wR_2 = 0.0403	R_1 = 0.0216, wR_2 = 0.0470	R_1 = 0.0179, wR_2 = 0.0426
Final R indexes [all data]	R_1 = 0.0163, wR_2 = 0.0404	R_1 = 0.0274, wR_2 = 0.0489	R_1 = 0.0184, wR_2 = 0.0428
Largest diff. peak/hole (e $Å^{-3}$)	4.42/-1.46	0.53/-0.56	0.31/-0.41
Flack parameter	0.003(3)		

Table S3 continued. Additional crystallographic data.

	[Rh(Ind)(SIPr)(H){Si(OEt)$_3$}] (**6**)	[{Rh(μ-OH)(SIPr)(COE)}$_2$] (**9**)	[Rh(SIPr)(μ-Bcat)$_2$(μ-B,O-Bcat)Rh(H)(SiPr)]
Empirical formula	$C_{42}H_{61}N_2O_3RhSi$	$C_{68}H_{90}N_4O_4Rh_2$	$C_{78.5}H_{101.5}B_3N_4O_6Rh_2$
Formula weight	772.92	1233.25	1435.38
T/K	100.0	100.0	100.0
Crystal system	monoclinic	Triclinic	orthorhombic
Space group	Cc	P-1	Pbca
a/Å	20.4826(9)	9.1518(18)	24.8674(4)
b/Å	11.1294(4)	12.442(3)	21.7758(4)
c/Å	18.2980(6)	14.300(3)	28.6884(5)
α/°	90	74.87(3)	90
β/°	107.733(2)	78.00(3)	90
γ/°	90	78.25(3)	90
Volume/Å^3	3973.0(3)	1518.1(6)	15535.0(5)
Z	4	1	8
ρ_{calc}g/cm^3	1.292	1.349	1.227
μ/mm^1	4.062	0.595	3.830
F(000)	1640.0	648.0	6028.0
Crystal size/mm^3	0.58 × 0.22 × 0.06	0.40 × 0.24 × 0.08	0.42 × 0.28 × 0.06
Radiation	CuKα (λ = 1.54184)	MoKα (λ = 0.71073)	CuKα (λ = 1.54178)
2Θ range for data collection/°	11.116 to 149.146	5.984 to 63.366	6.162 to 149.662
Index ranges	-25 ≤ h ≤ 25, -13 ≤ k ≤ 13, -21 ≤ l ≤ 22	-13 ≤ h ≤ 13, -17 ≤ k ≤ 18, 0 ≤ l ≤ 21	-29 ≤ h ≤ 31, -27 ≤ k ≤ 27, -35 ≤ l ≤ 35
Reflections collected	59950	9781	197875
Independent reflections	7781 [R_{int} = 0.0451, R_{sigma} = 0.0333]	9781 [R_{int} = ?, R_{sigma} = 0.0395]	15899 [R_{int} = 0.0451, R_{sigma} = 0.0193]
Data/ restraints/ parameters	7781/2/457	9781/0/365	15899/48/1010
Goodness-of-fit on F^2	1.045	1.051	1.045
Final R indexes [I>=2σ (I)]	R_1 = 0.0200, wR_2 = 0.0513	R_1 = 0.0278, wR_2 = 0.0522	R_1 = 0.0359, wR_2 = 0.1023
Final R indexes [all data]	R_1 = 0.0202, wR_2 = 0.0514	R_1 = 0.0333, wR_2 = 0.0542	R_1 = 0.0395, wR_2 = 0.1054
Largest diff. peak/hole (e Å^{-3})	0.42/-0.48	0.50/-0.72	1.22/-1.24
Flack parameter	0.034(6)		

Table S3 continued. Additional crystallographic data.

	[{RhCp*(C_2H_3)}$_2$] (**10**)	[Rh(Flu-Dipp)(C_2H_4)] (**11**)	[Rh(Flu-Dipp)(CO)] (**13**)
Empirical formula	$C_{24}H_{36}Rh_2$	$C_{32}H_{37}N_2Rh$	$C_{31}H_{33}N_2ORh$
Formula weight	530.35	552.54	552.50
T/K	100.0	100.0	100.0
Crystal system	monoclinic	monoclinic	orthorhombic
Space group	C2/c	$P2_1$	$P2_12_12_1$
a/Å	11.5282(15)	8.6176(5)	11.3032(6)
b/Å	12.5724(16)	13.6545(8)	13.6808(7)
c/Å	15.699(2)	11.1334(6)	16.3132(8)
α/°	90	90	90
β/°	105.622(5)	91.346(2)	90
γ/°	90	90	90
Volume/Å^3	2191.4(5)	1309.69(13)	2522.6(2)
Z	4	2	4
ρ_{calc}g/cm^3	1.608	1.401	1.455
μ/mm^1	1.511	0.675	0.704
F(000)	1080.0	576.0	1144.0
Crystal size/mm^3	0.25 × 0.20 × 0.12	0.34 × 0.2 × 0.04	0.44 × 0.34 × 0.3
Radiation	MoKα (λ = 0.71073)	MoKα (λ = 0.71073)	MoKα (λ = 0.71073)
2Θ range for data collection/°	6.508 to 66.312	5.592 to 72.774	5.3 to 64.926
Index ranges	-17 ≤ h ≤ 17, 0 ≤ k ≤ 19, 0 ≤ l ≤ 24	-14 ≤ h ≤ 14, -22 ≤ k ≤ 22, -18 ≤ l ≤ 18	-17 ≤ h ≤ 12, -20 ≤ k ≤ 19, -24 ≤ l ≤ 16
Reflections collected	5672	95195	18129
Independent reflections	5672 [R_{int} = ?, R_{sigma} = 0.0136]	12756 [R_{int} = 0.0709, R_{sigma} = 0.0508]	8590 [R_{int} = 0.0410, R_{sigma} = 0.0634]
Data/ restraints/ parameters	5672/0/133	12756/1/336	8590/0/320
Goodness-of-fit on F^2	1.200	1.022	0.997
Final R indexes [I>=2σ (I)]	R_1 = 0.0185, wR_2 = 0.0533	R_1 = 0.0360, wR_2 = 0.0730	R_1 = 0.0333, wR_2 = 0.0636
Final R indexes [all data]	R_1 = 0.0209, wR_2 = 0.0651	R_1 = 0.0481, wR_2 = 0.0781	R_1 = 0.0391, wR_2 = 0.0659
Largest diff. peak/hole (e Å^{-3})	0.49/-0.76	1.19/-1.67	0.47/-0.47
Flack parameter		-0.016(11)	-0.035(18)

Table S3 continued. Additional crystallographic data.

	[Rh(Flu-Mes)(CO)] (**15**)	[Rh(Flu-Mes)(COE)] (**14**)	[Rh(Flu-Me)(CO)] (**17**)
Empirical formula	$C_{31}H_{29.5}F_{0.5}N_2ORh$	$C_{35}H_{41}N_2Rh$	$C_{20}H_{17}N_2ORh$
Formula weight	558.47	592.61	404.26
T/K	100.0	100.0	100.0
Crystal system	orthorhombic	monoclinic	orthorhombic
Space group	Pbca	$P2_1/c$	$Pca2_1$
a/Å	17.181(2)	13.9747(3)	16.8545(4)
b/Å	14.2963(15)	10.1295(3)	11.3374(2)
c/Å	20.496(3)	19.8369(5)	17.1497(4)
α/°	90	90	90
β/°	90	94.9450(10)	90
γ/°	90	90	90
Volume/Å^3	5034.5(11)	2797.59(12)	3277.07(12)
Z	8	4	8
ρ_{calc}g/cm^3	1.474	1.407	1.639
μ/mm^1	5.721	5.121	8.485
F(000)	2296.0	1240.0	1632.0
Crystal size/mm^3	0.38 × 0.04 × 0.02	0.36 × 0.18 × 0.08	0.18 × 0.12 × 0.06
Radiation	CuKα (λ = 1.54178)	CuKα (λ = 1.54178)	CuKα (λ = 1.54178)
2Θ range for data collection/°	8.628 to 101.43	9.812 to 144.466	7.798 to 149.416
Index ranges	$-17 \le h \le 17$, $-14 \le k \le 14$, $-20 \le l \le 20$	$-17 \le h \le 17$, $-12 \le k \le 12$, $-24 \le l \le 24$	$-21 \le h \le 21$, $-14 \le k \le 14$, $-21 \le l \le 21$
Reflections collected	66163	80325	80320
Independent reflections	2666 [R_{int} = 0.5631, R_{sigma} = 0.1423]	5456 [R_{int} = 0.0332, R_{sigma} = 0.0147]	6551 [R_{int} = 0.0313, R_{sigma} = 0.0149]
Data/ restraints/ parameters	2666/342/328	5456/0/365	6551/1/436
Goodness-of-fit on F^2	1.071	1.031	1.052
Final R indexes [I>=2σ (I)]	R_1 = 0.0771, wR_2 = 0.1471	R_1 = 0.0193, wR_2 = 0.0498	R_1 = 0.0147, wR_2 = 0.0365
Final R indexes [all data]	R_1 = 0.1522, wR_2 = 0.1819	R_1 = 0.0196, wR_2 = 0.0500	R_1 = 0.0149, wR_2 = 0.0367
Largest diff. peak/hole (e Å^{-3})	0.71/-1.10	0.45/-0.38	0.32/-0.36
Flack parameter			0.347(6)

4 High resolution mass spectrometry data

N.B. ChemDraw structures are indicative only of MW, not structure.

4.1 Rh complexes

4.1.1 [Rh(Ind)(SIPr)(COE)] (3)

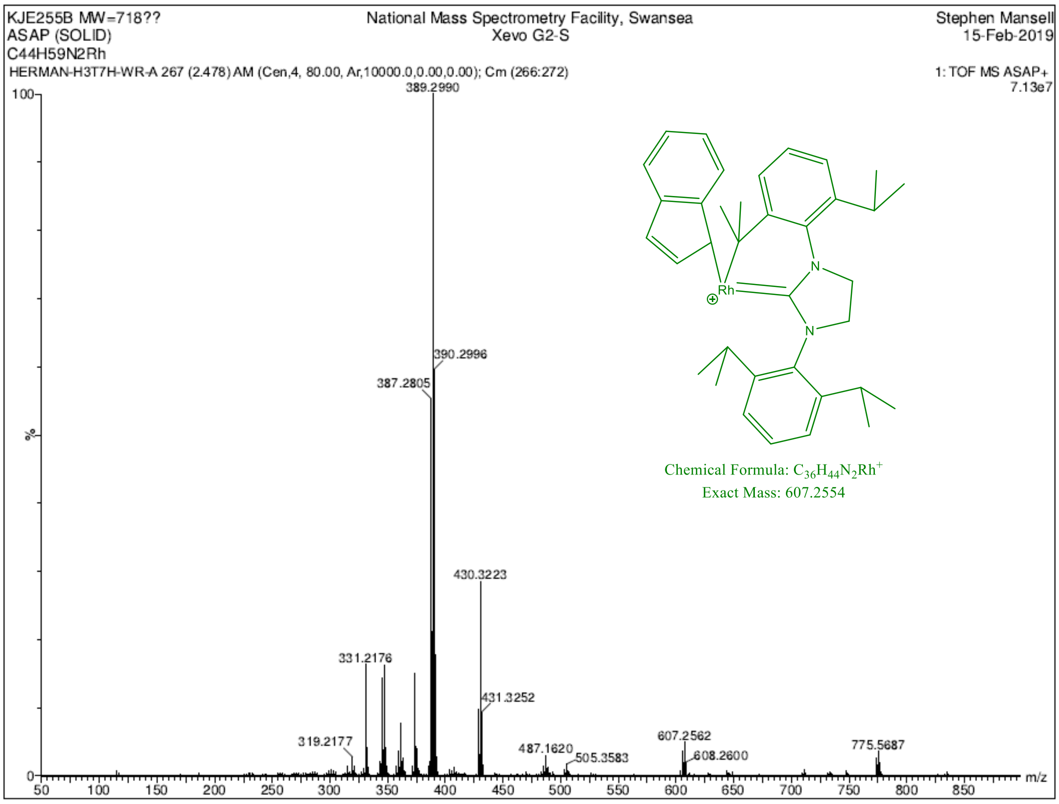

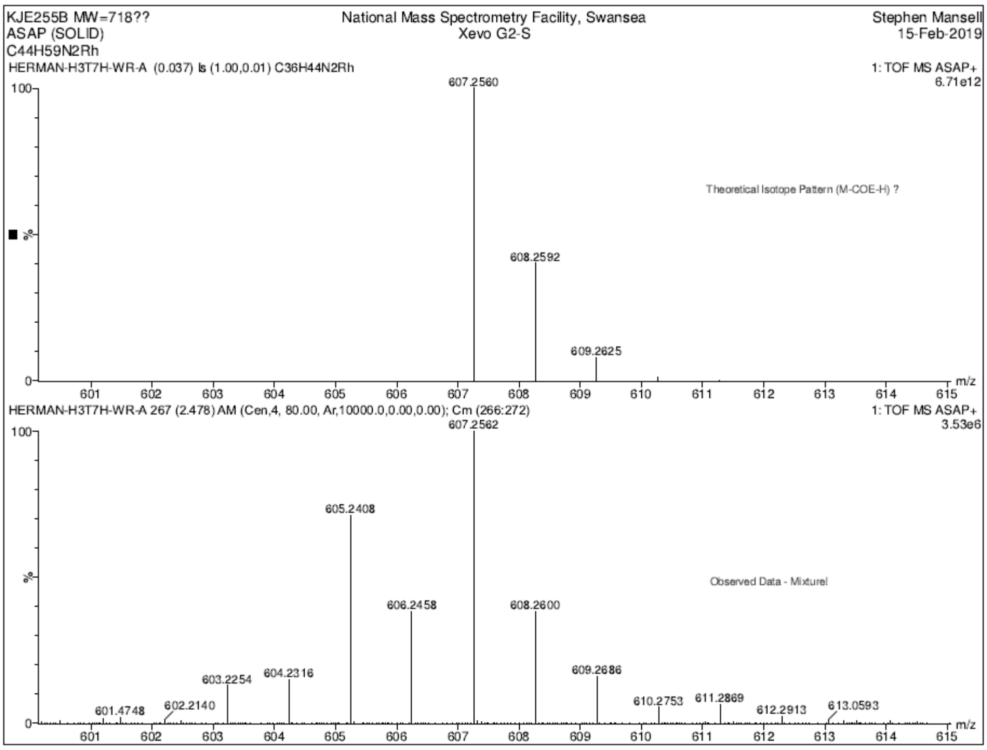

Figure S15. Mass spectrum of **3** (top) and isotopic analysis (bottom)

4.1.2 [Rh(Ind)(SIMes)(CO)] (2b)

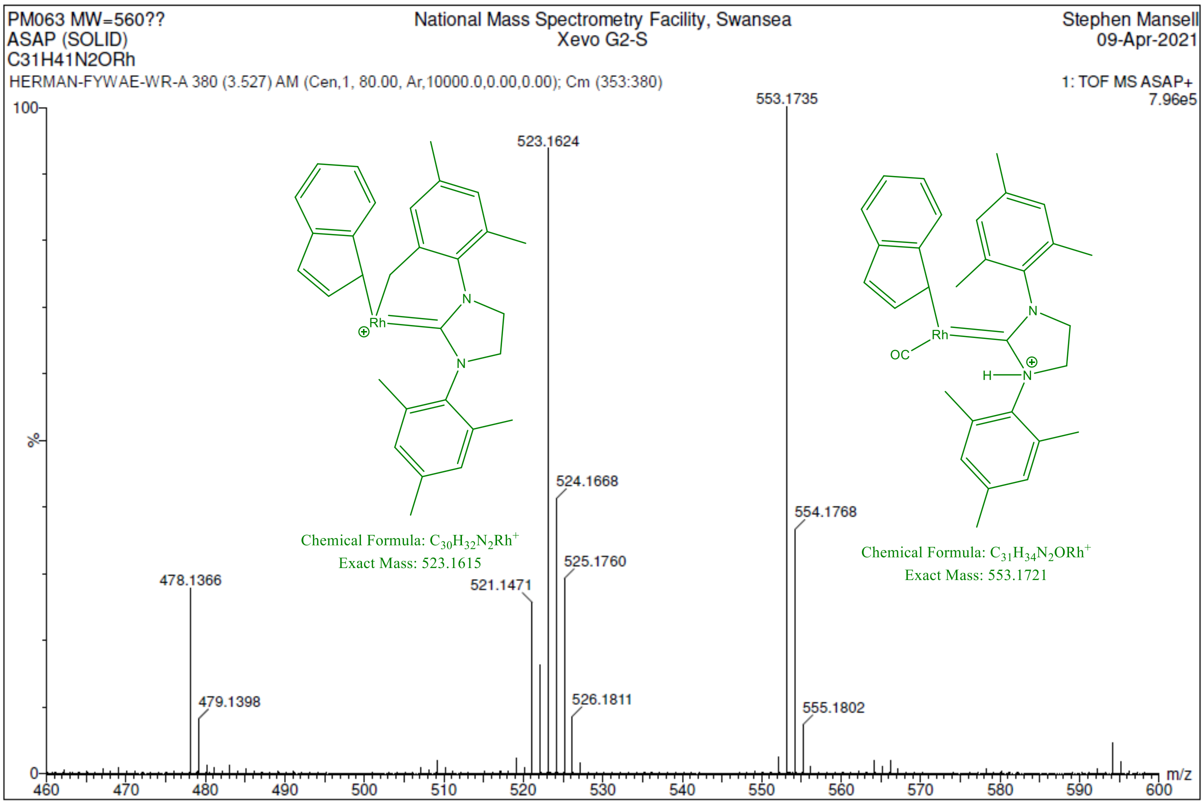

Figure S16. Mass spectrum of **2b**.

4.1.3 [Rh(Ind)(IMes)(CO)] (2c)

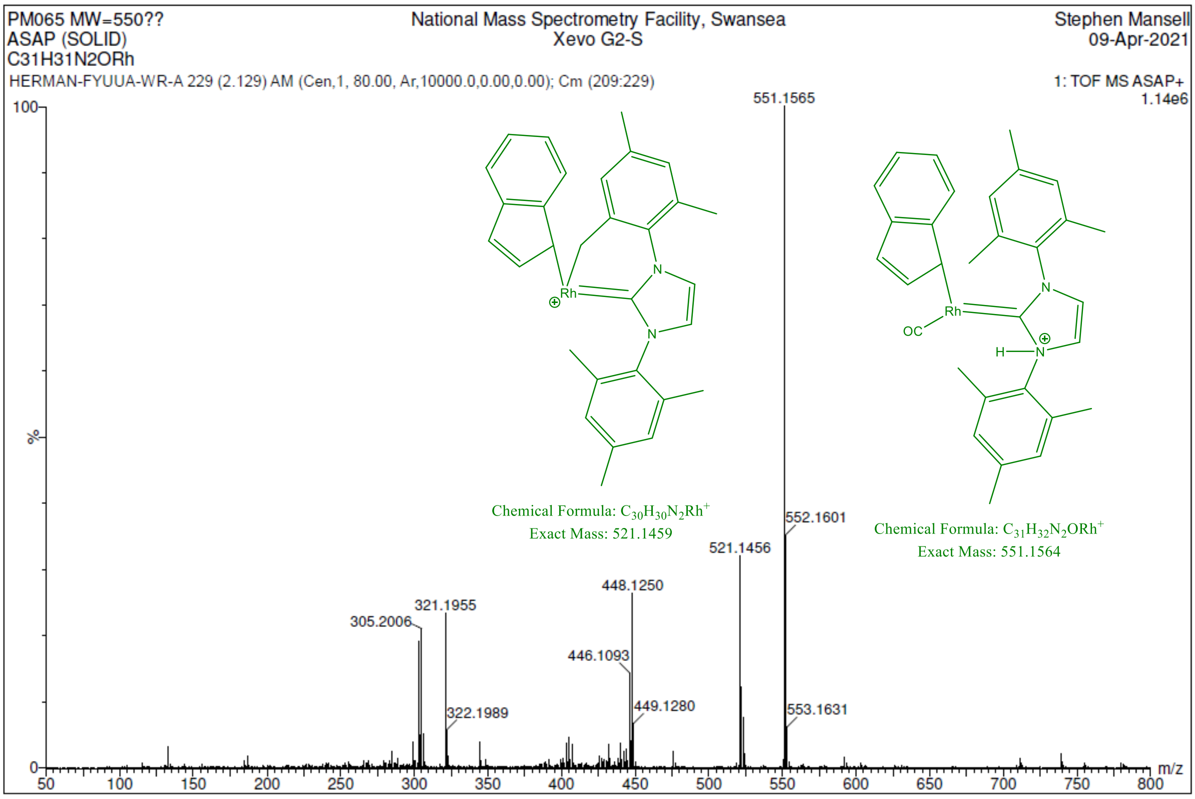

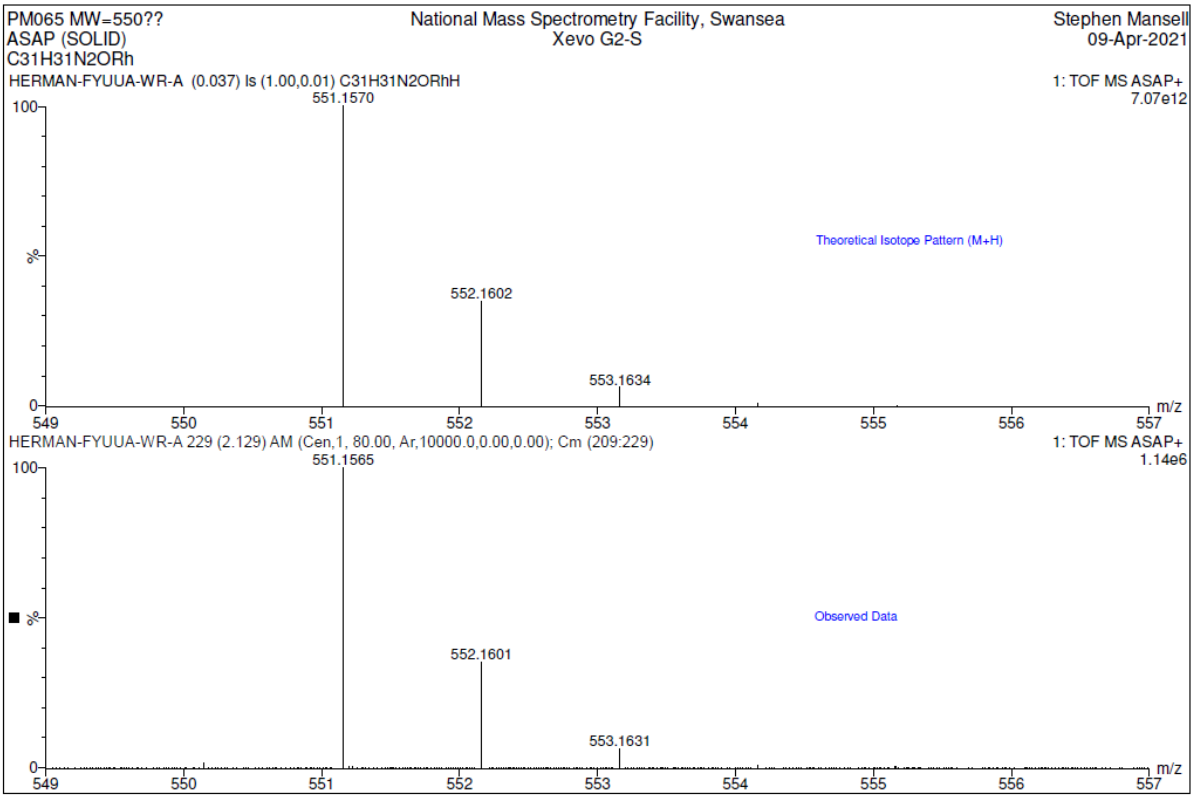

Figure S17. Mass spectrum of [Rh(Ind)(IMes)(CO)] (**2c**, top) and isotopic analysis for $[M+H]^+$ (bottom).

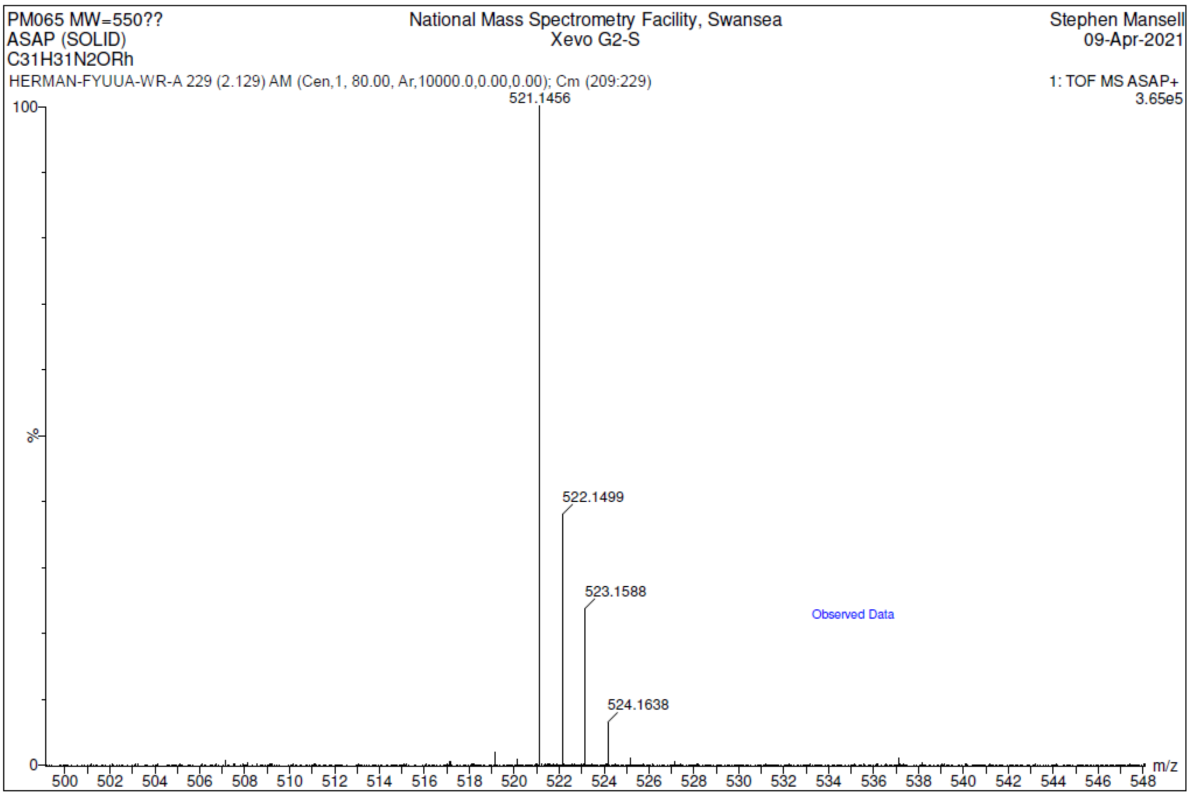

Figure S18. Mass spectral data for **2c** for $[M-CO-H]^+$.

4.1.4 [Rh(Flu-Dipp)(CO)] (13)

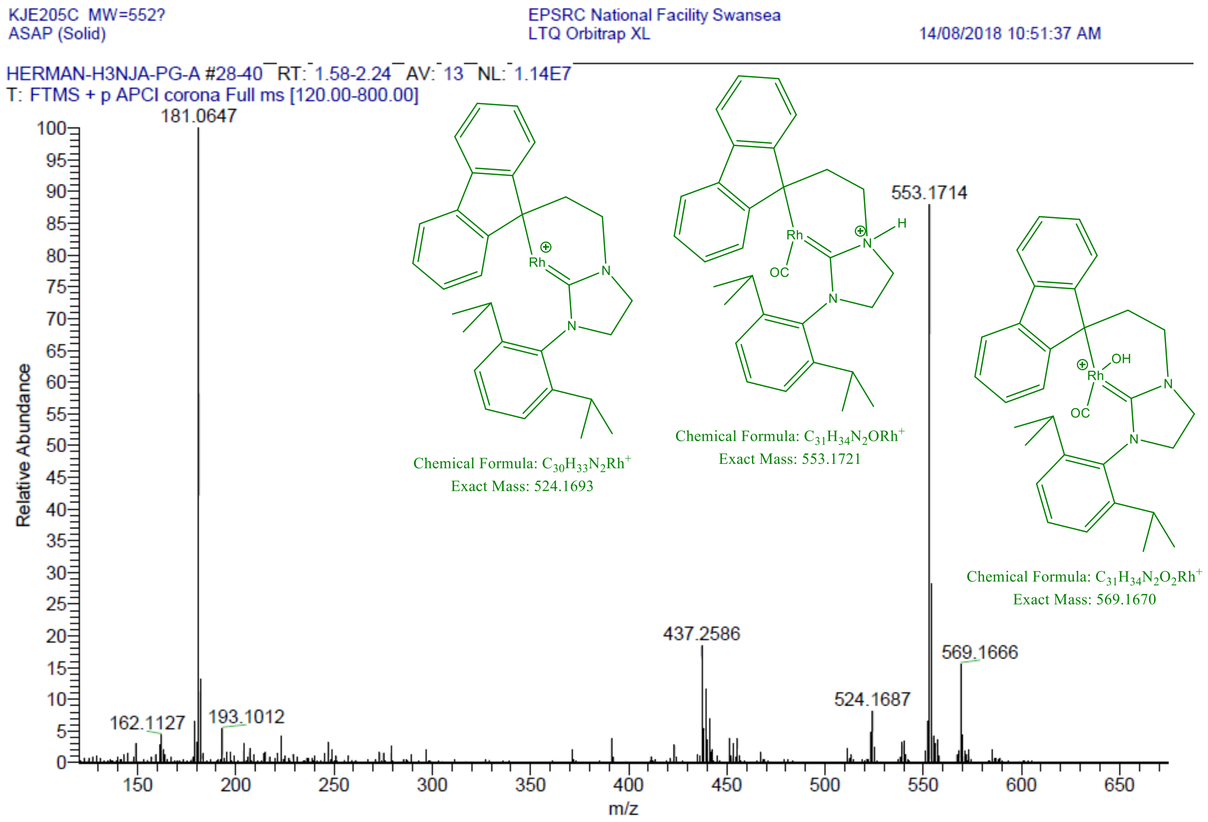

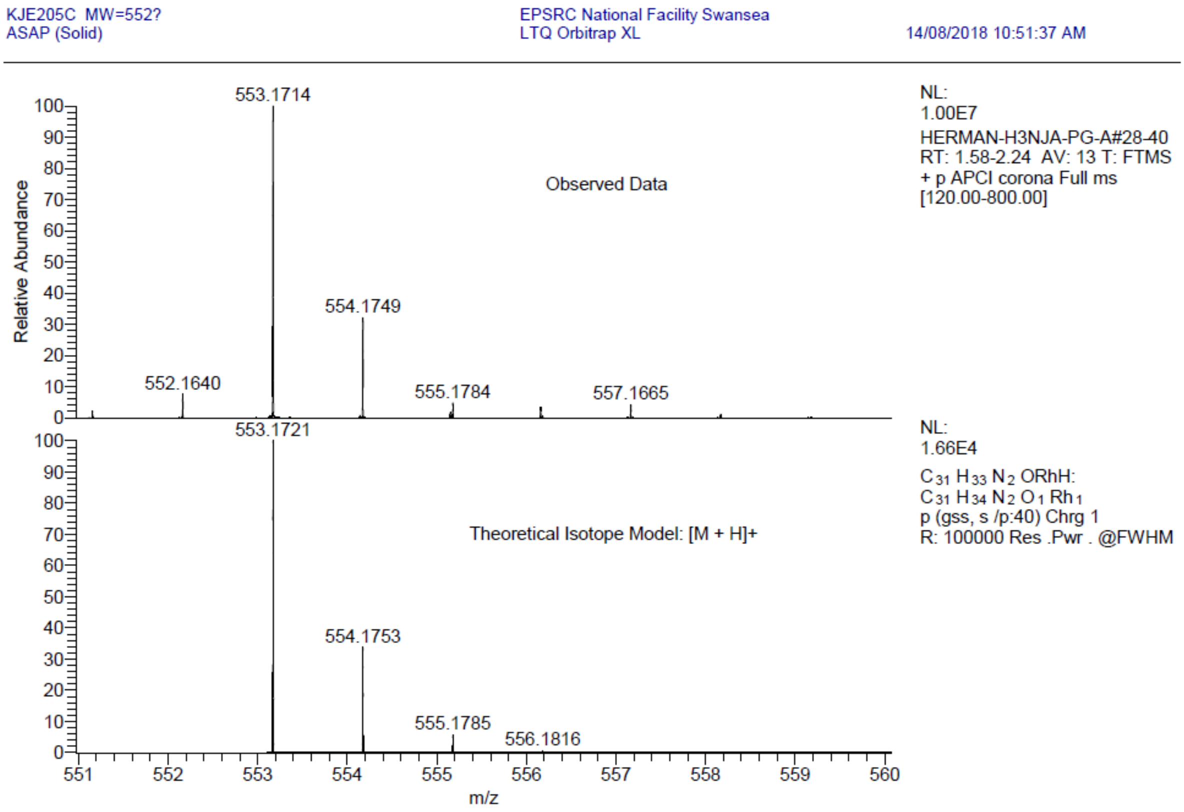

Figure S19. Mass spectrum of **13** (top) and isotopic analysis for $[M+H]^+$ (bottom)

4.1.5 [Rh(Flu-Mes)(CO)] (15)

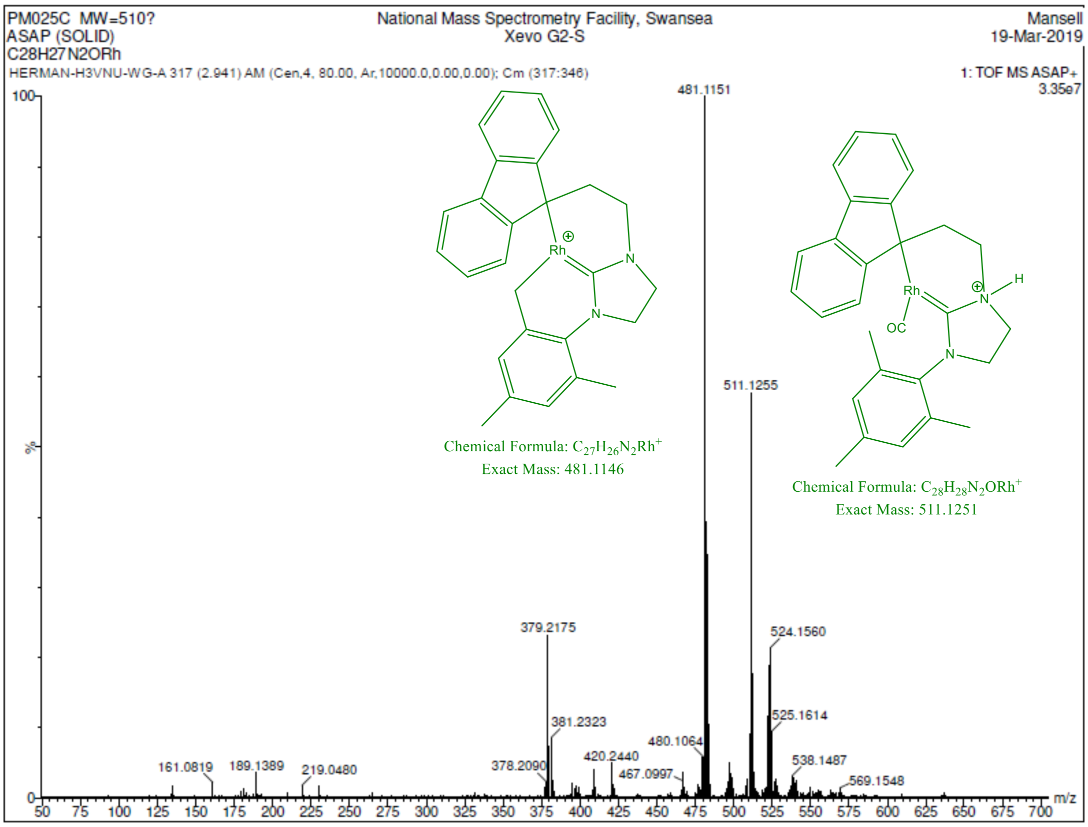

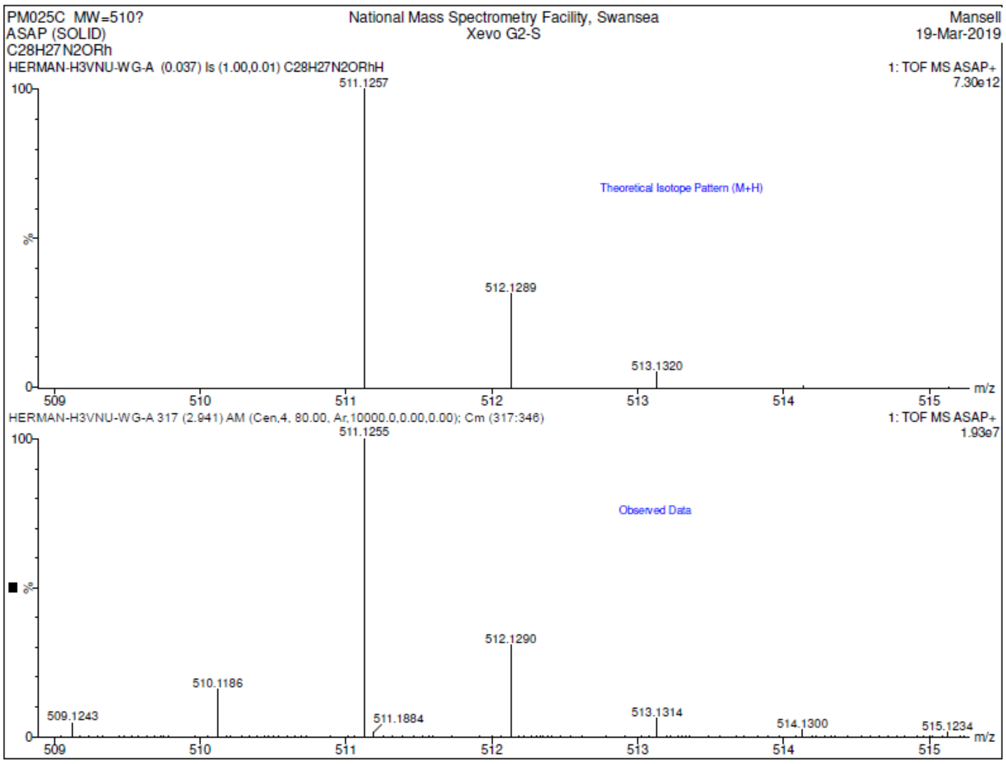

Figure S20. Mass spectrum for **15** (top) and isotope analysis for $[M+H]^+$.

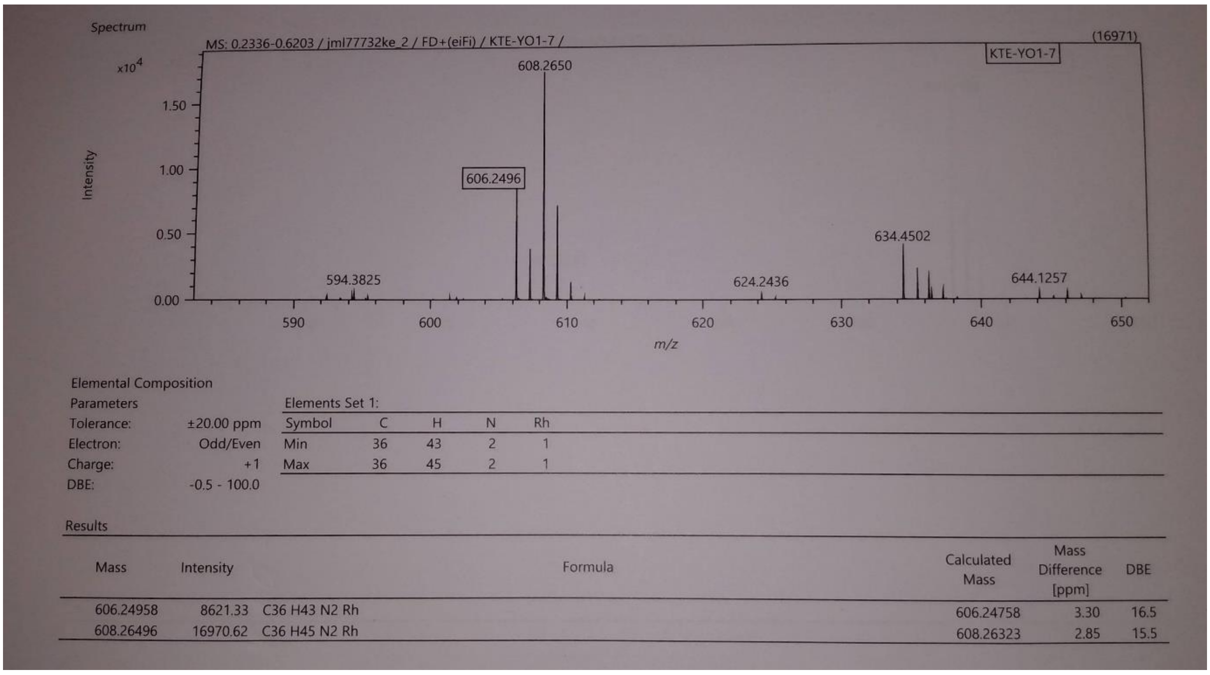

Elemental Composition

Parameters		Elements Set 1:				
Tolerance:	±20.00 ppm	Symbol	C	H	N	Rh
Electron:	Odd/Even	Min	36	43	2	1
Charge:	+1	Max	36	45	2	1
DBE:	-0.5 - 100.0					

Results

Mass	Intensity	Formula	Calculated Mass	Mass Difference [ppm]	DBE
606.24958	8621.33	C36 H43 N2 Rh	606.24758	3.30	16.5
608.26496	16970.62	C36 H45 N2 Rh	608.26323	2.85	15.5

Figure S21. LIFDI mass spectrum for the photoproducts from the reaction of **3** with 400 nm light.

4.2 Boron compounds

4.2.1 Borylation of C_6H_6/C_6D_6 mixture: PhBpin and C_6D_5Bpin

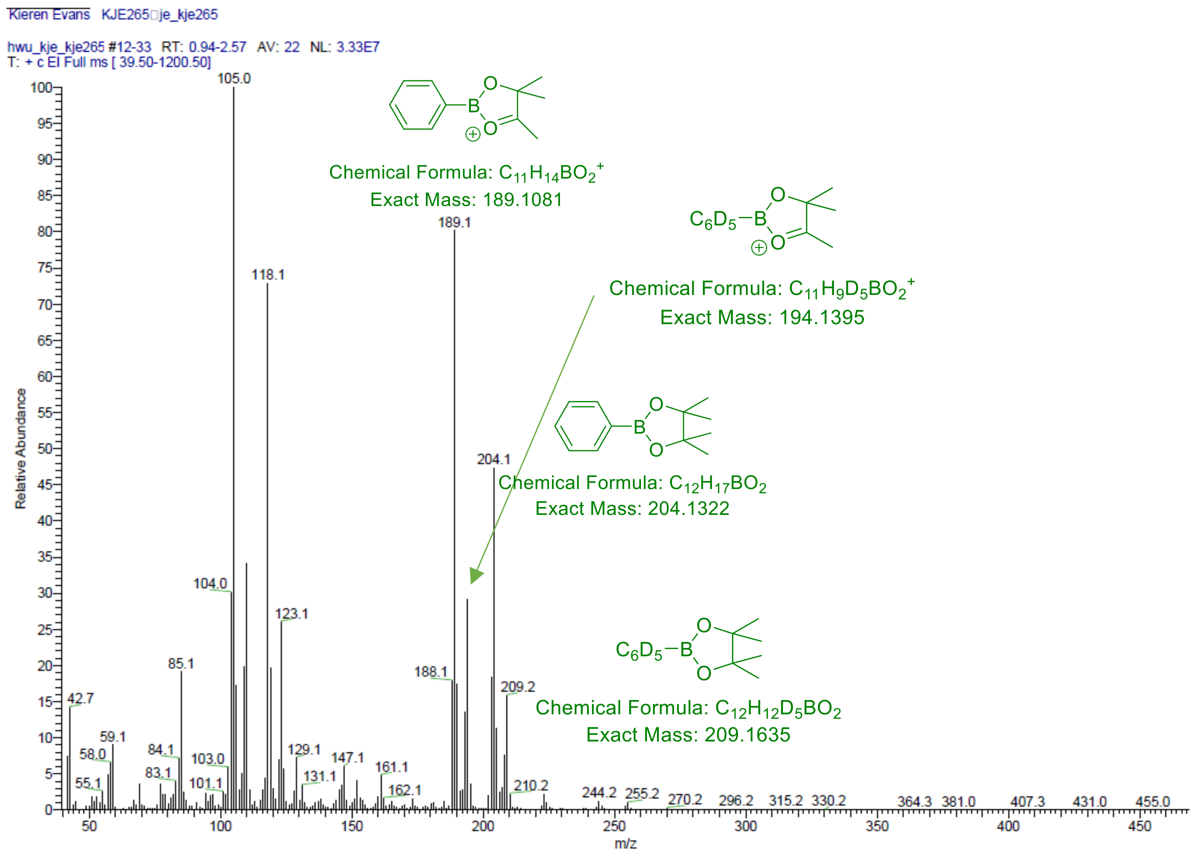

Figure S22. Mass spectrum for PhBpin/C_6D_5Bpin mixture.

4.2.2 Borylation of toluene

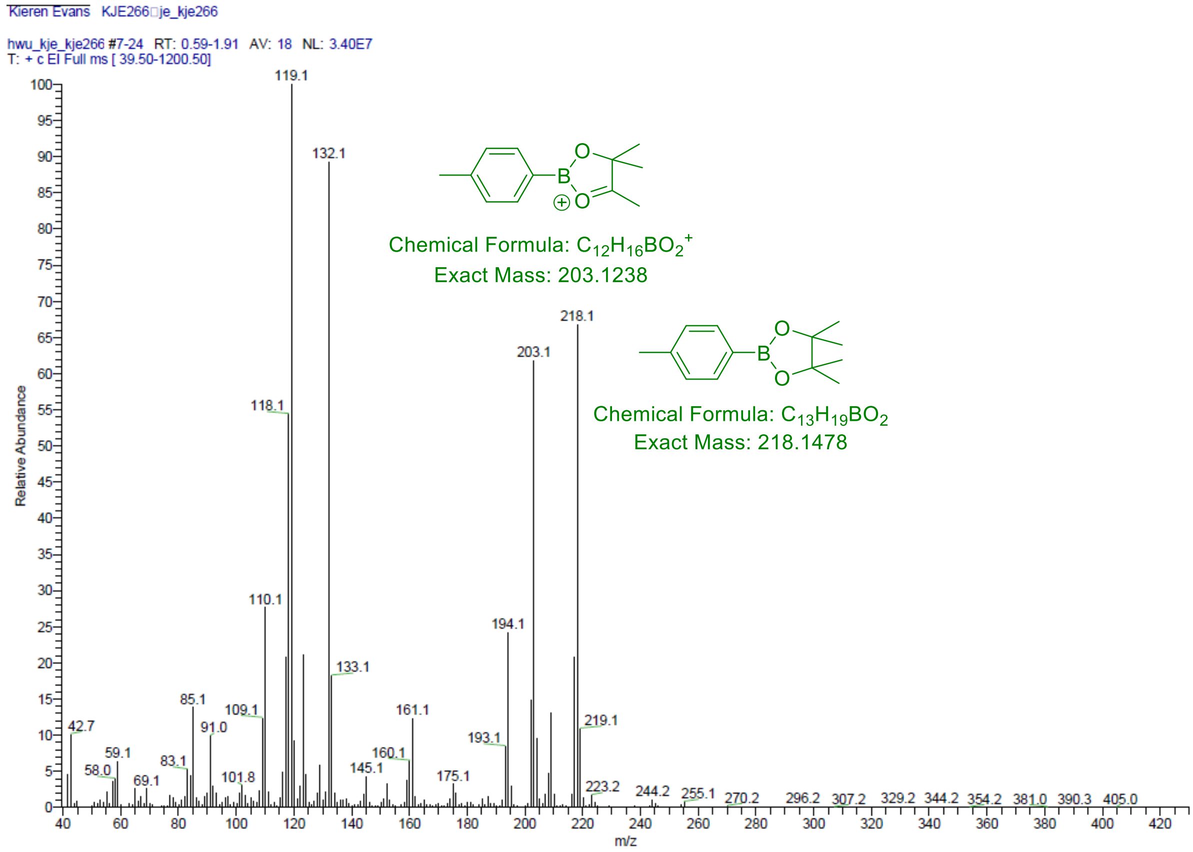

Figure S23. Mass spectrum of tolylBin.

4.2.3 Borylation of fluorobenzene

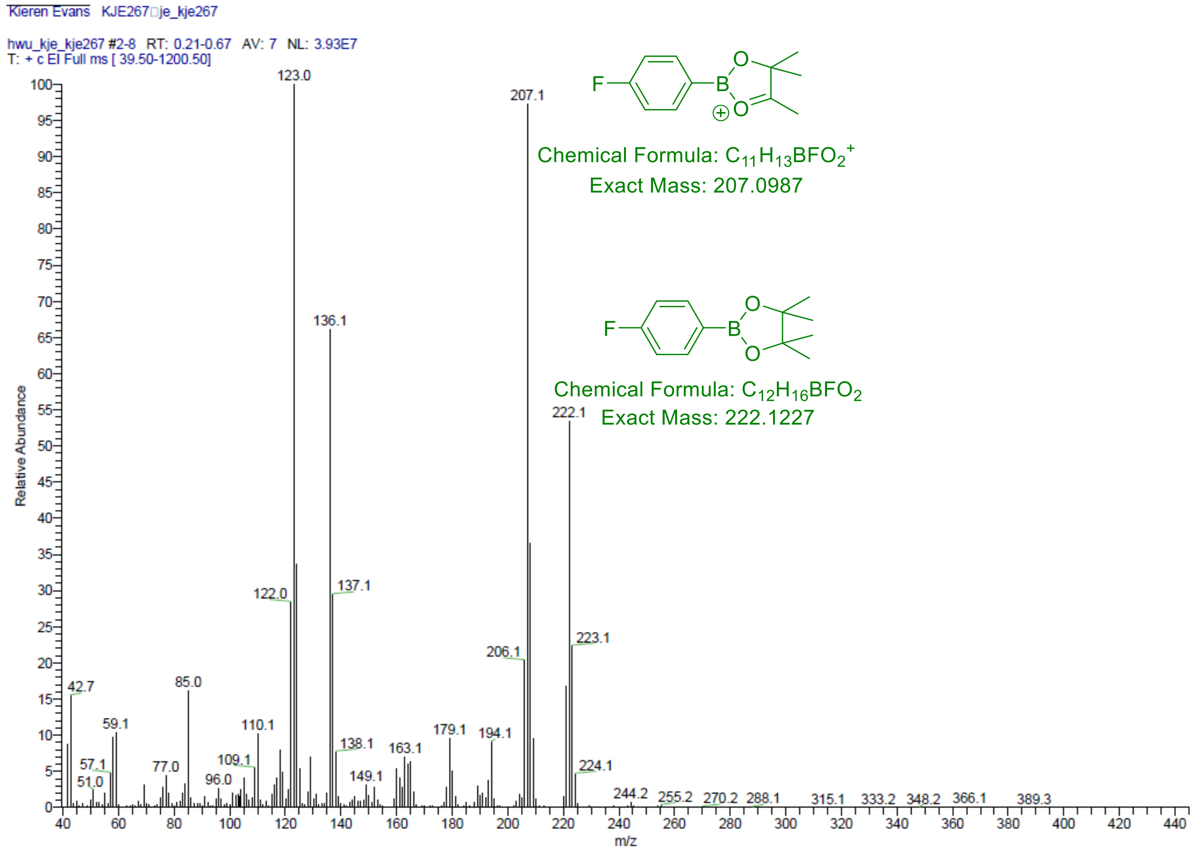

Figure S24. Mass spectrum for C_6H_4FBpin.

4.2.4 Borylation of mesitylene

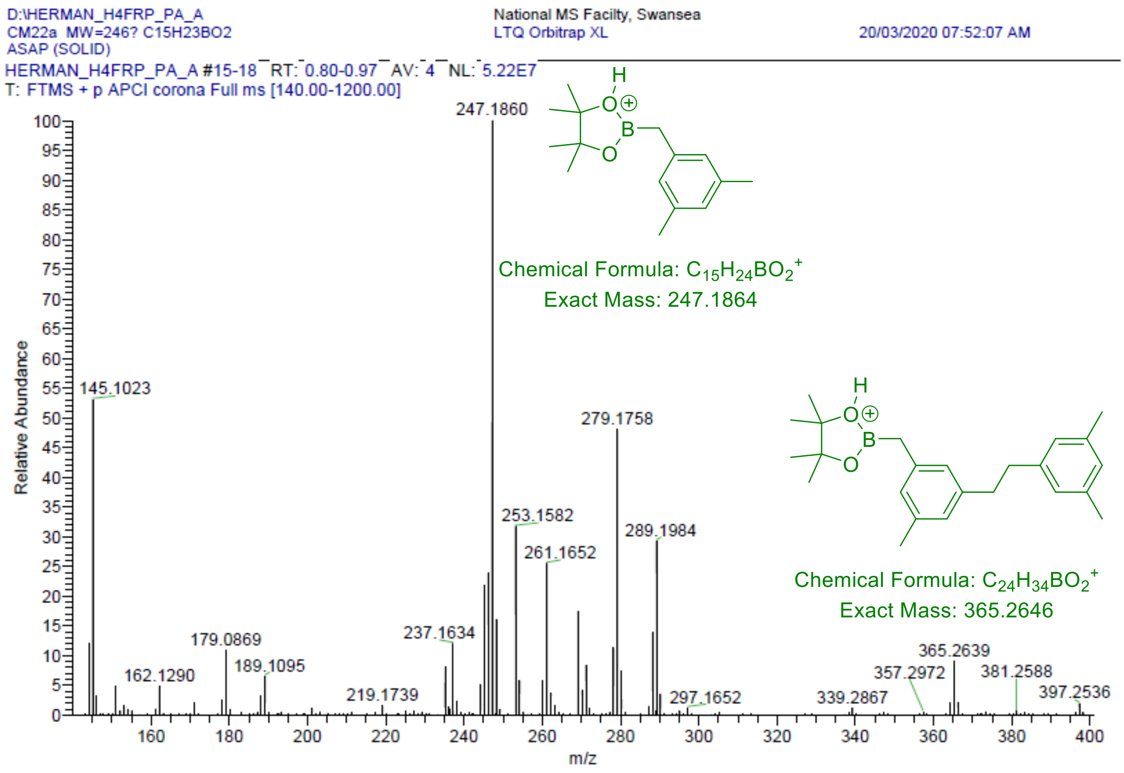

Figure S25. Mass spectrum for 3,5-$Me_2C_6H_3CH_2$Bpin.

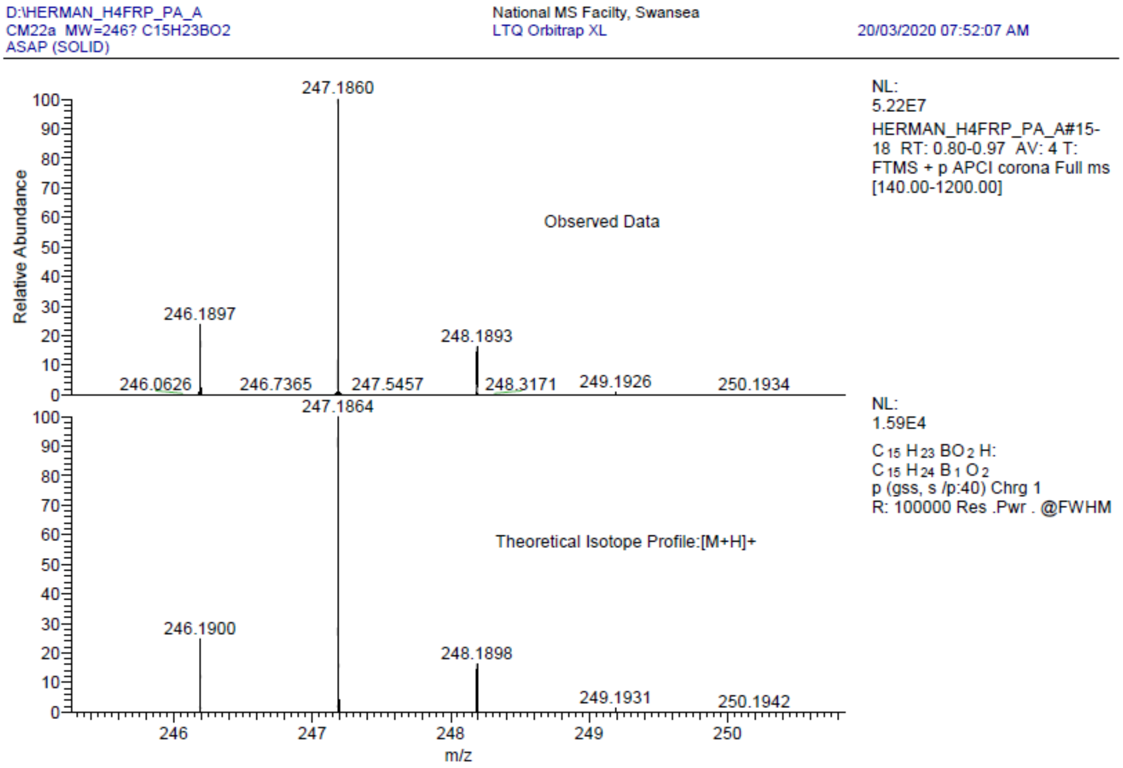

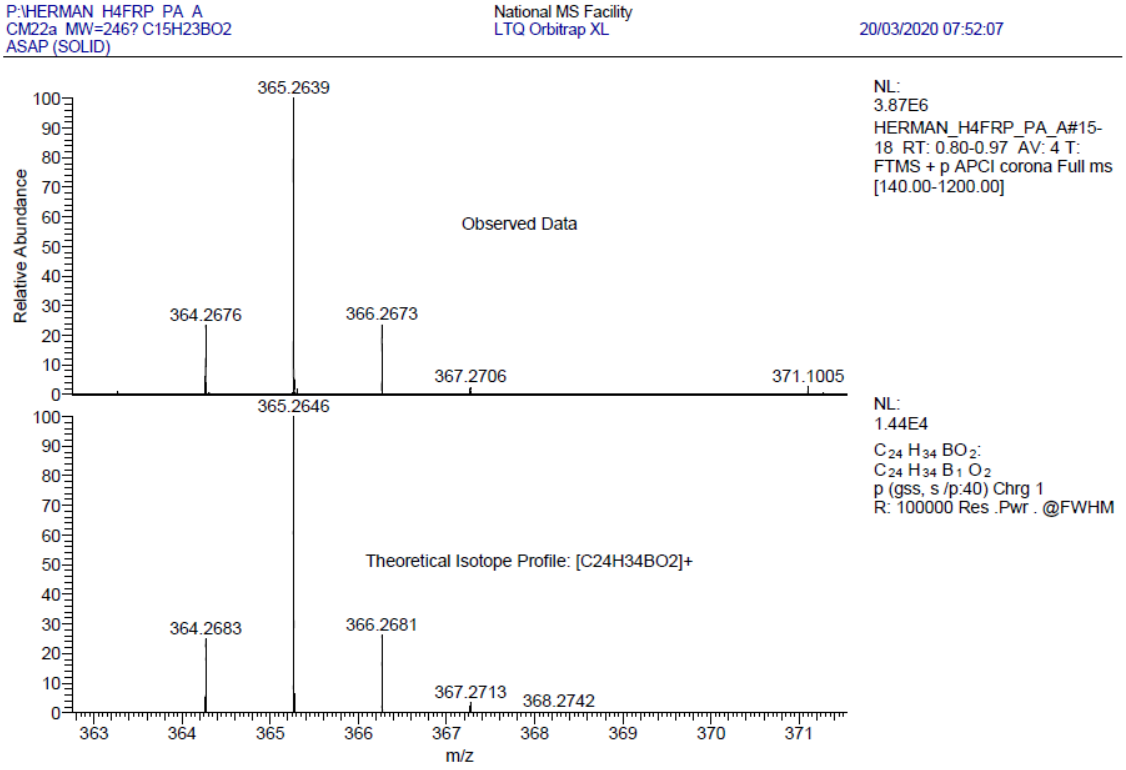

Figure S26. Isotope analysis for monoborylated species 3,5-$Me_2C_6H_3CH_2Bpin$ (top) and borylated + coupled species (bottom).

4.2.5 Borylation of anisole

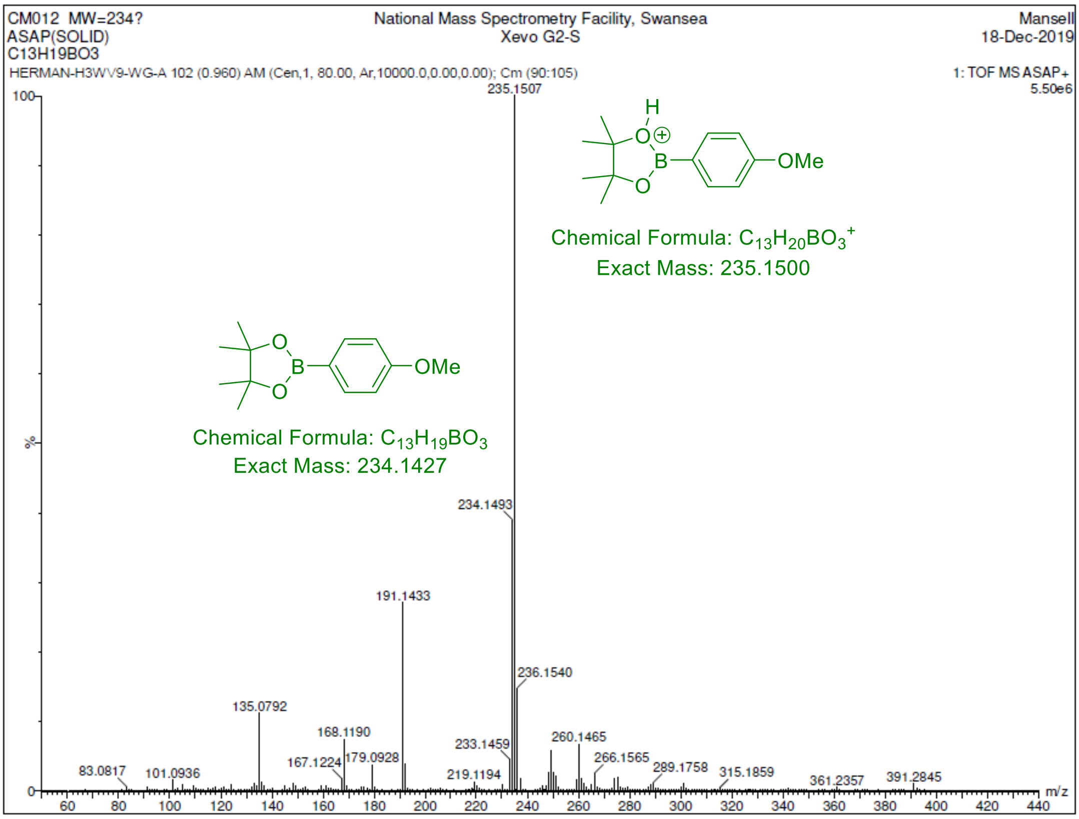

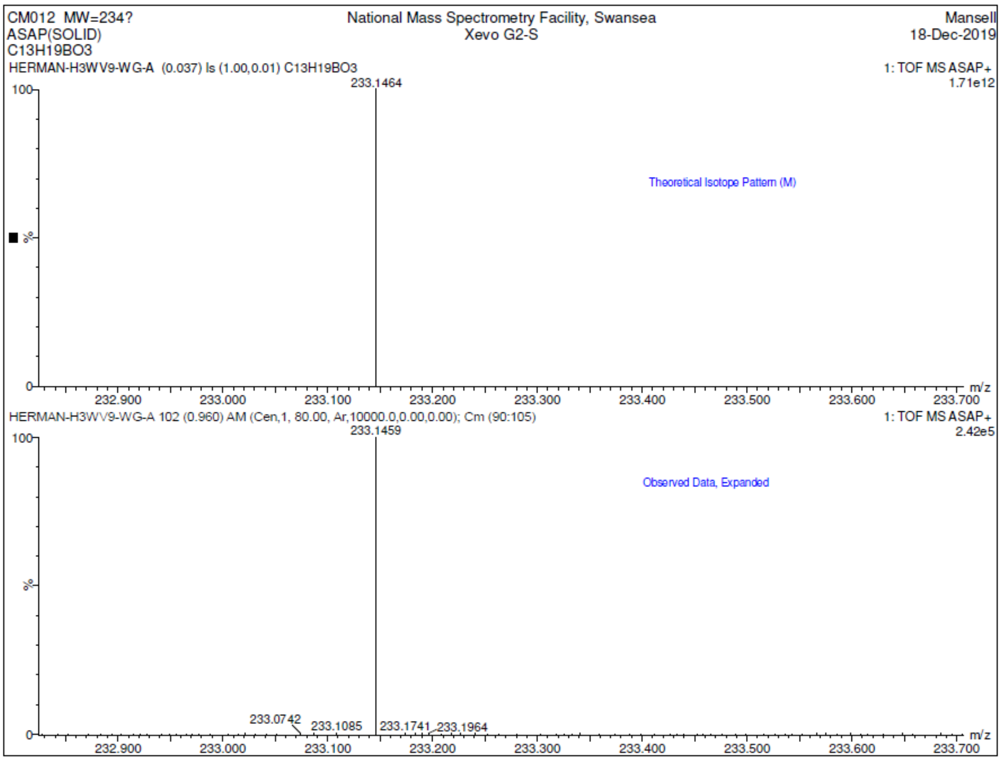

Figure S27. Mass spectrum of $C_6H_3OMeBpin$ (top) and isotope analysis (bottom).

4.2.6 Borylation of biphenyl

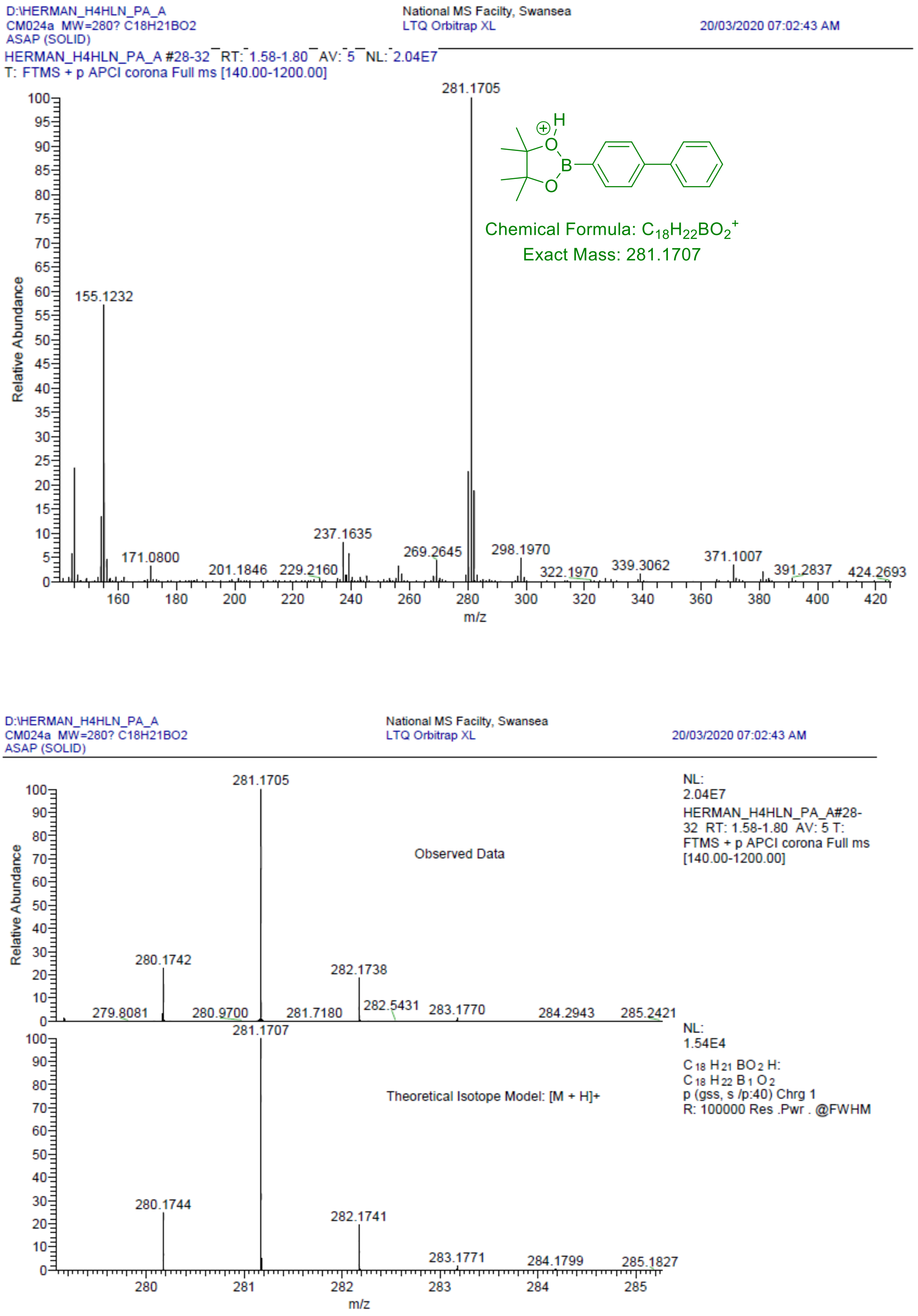

Figure S28. Mass spectrum of $C_6H_3PhBpin$ (top) and isotope analysis (bottom).

4.2.7 Borylation of diphenyl ether

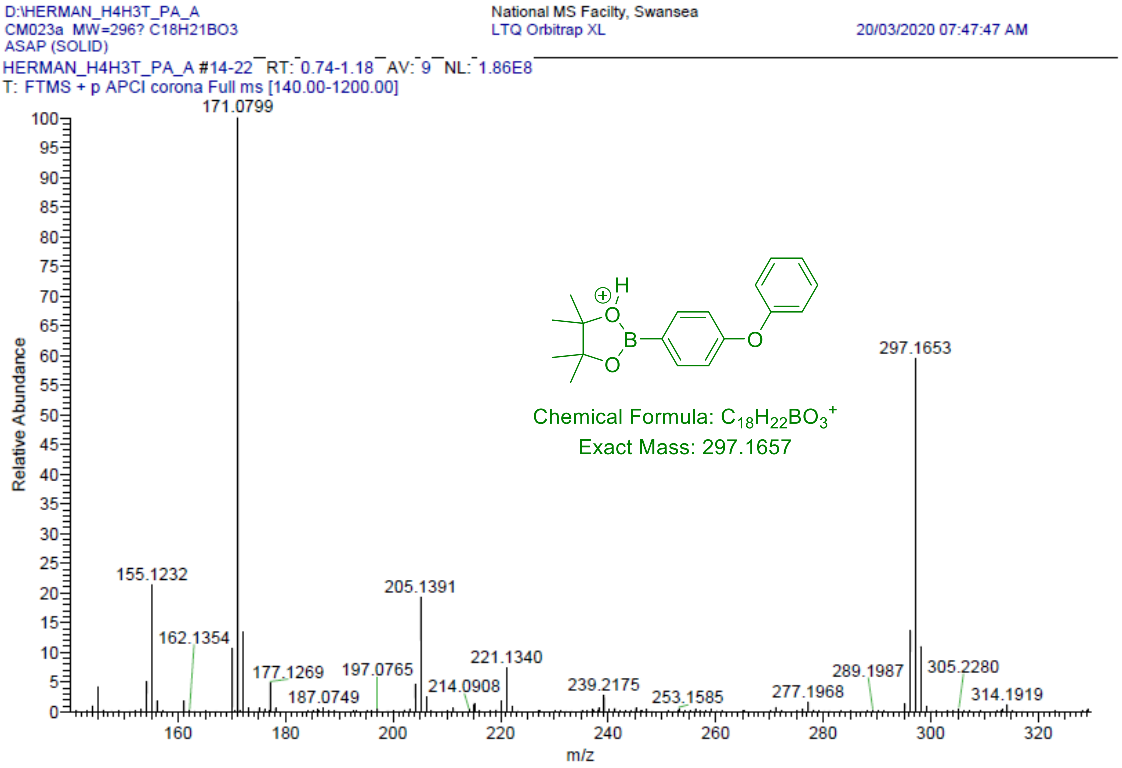

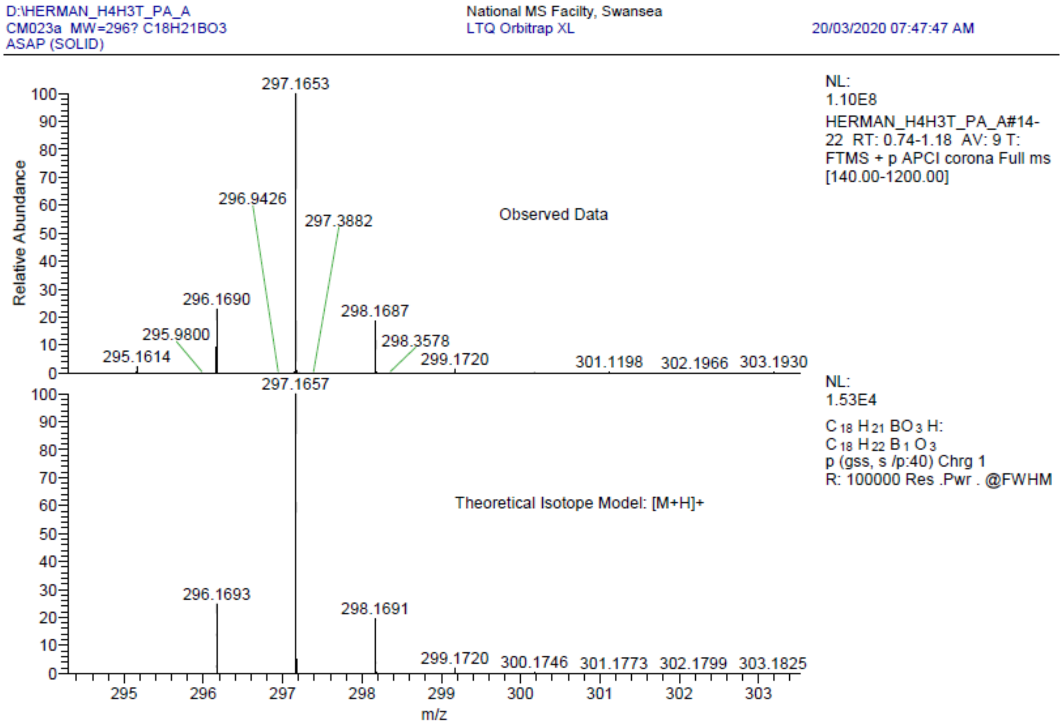

Figure S29. Mass spectrum of $C_6H_3OPhBpin$ (top) and isotope analysis (bottom).

4.2.8 octylBpin and pinBC$_8$H$_{16}$BPin

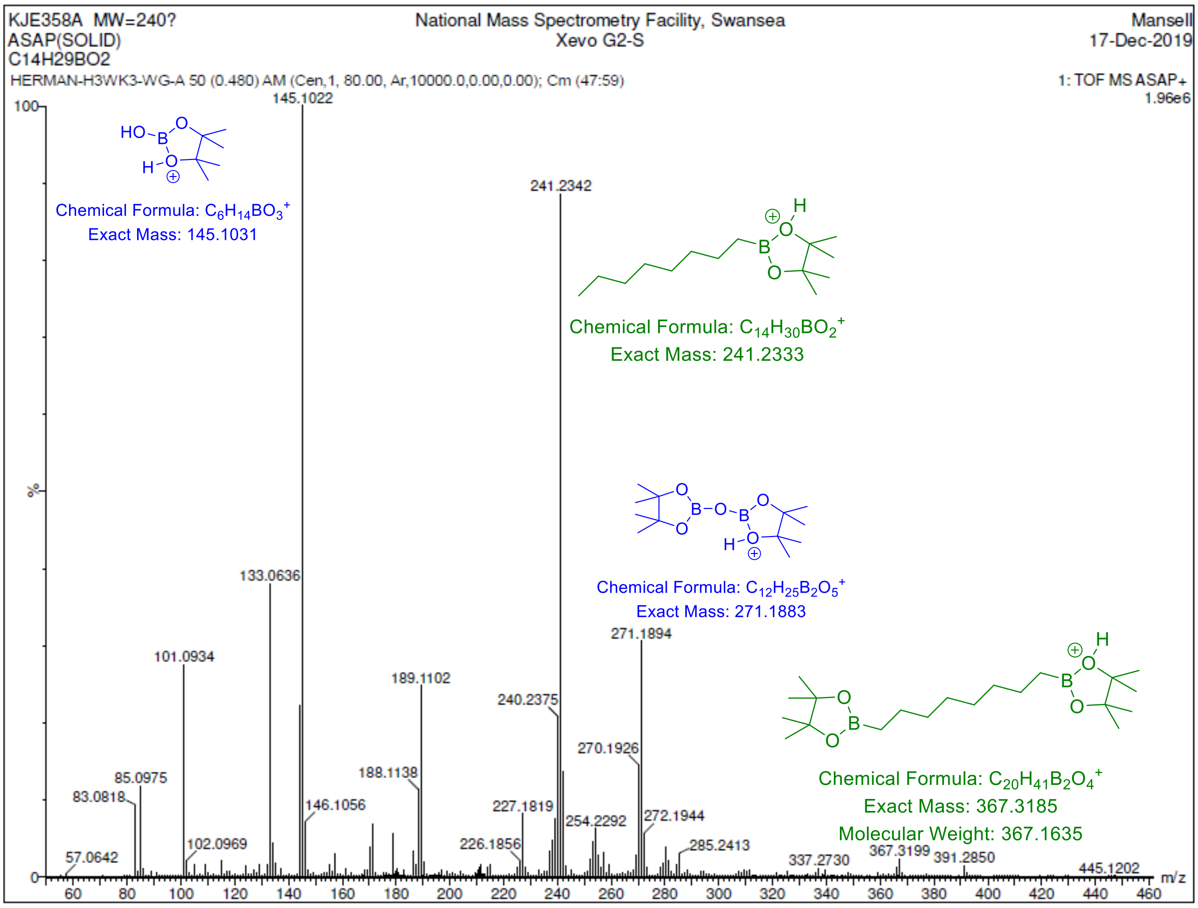

Figure S30. Mass spectrum of octylBpin.

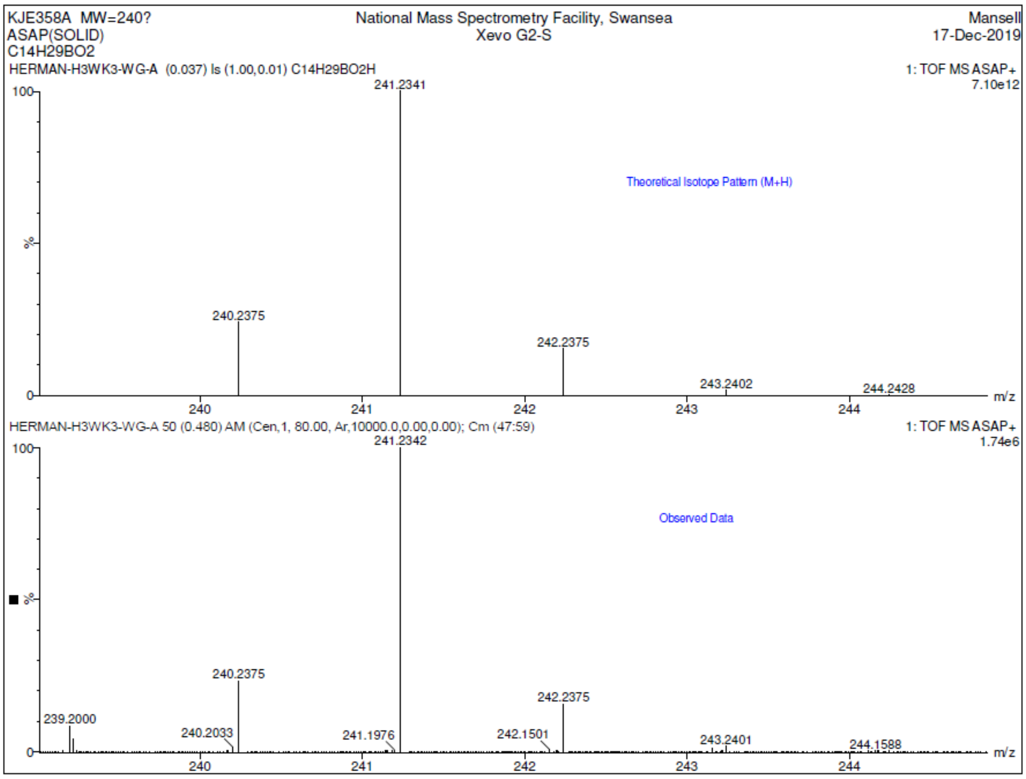

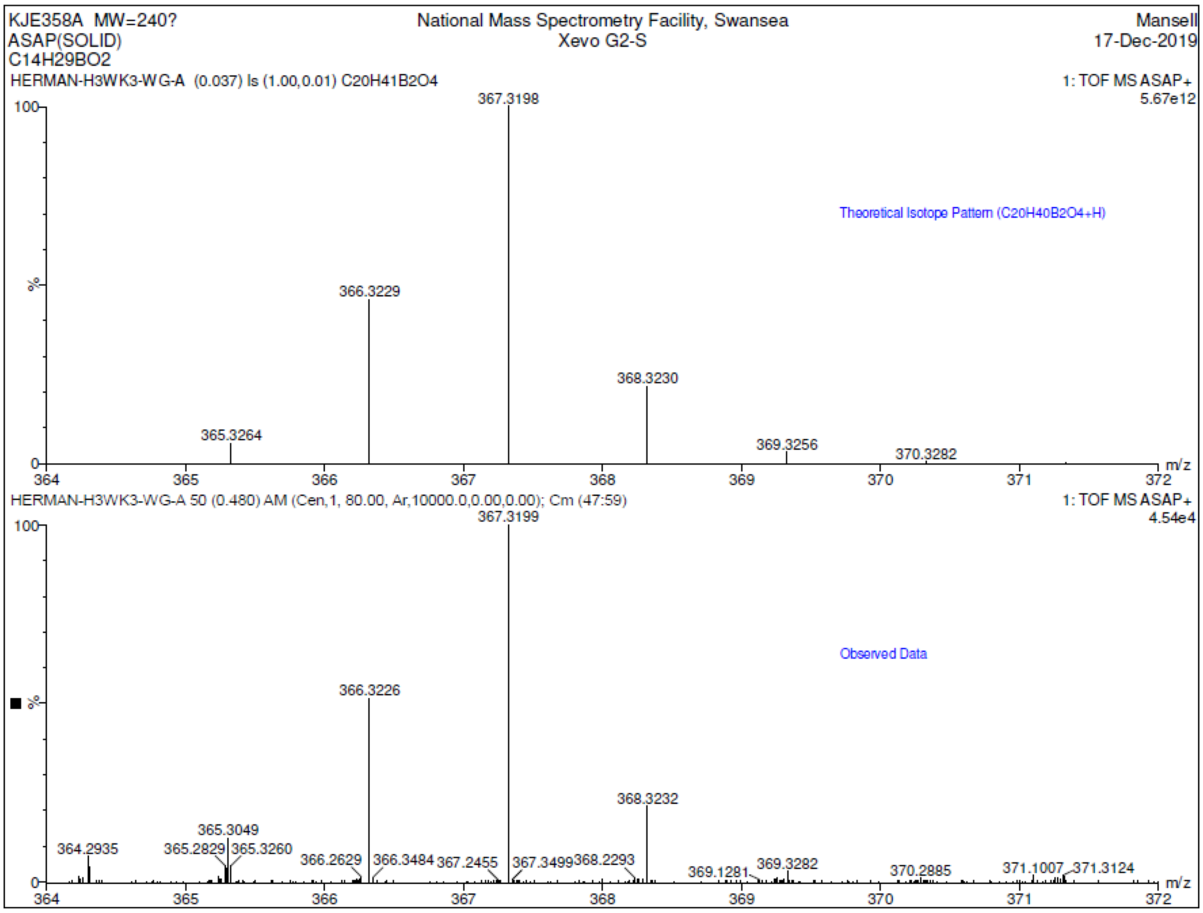

Figure S31. Isotope analysis for octylBpin (top) and diborylated product (bottom).

4.2.9 decylBpin and pinBC$_{10}$H$_{20}$BPin

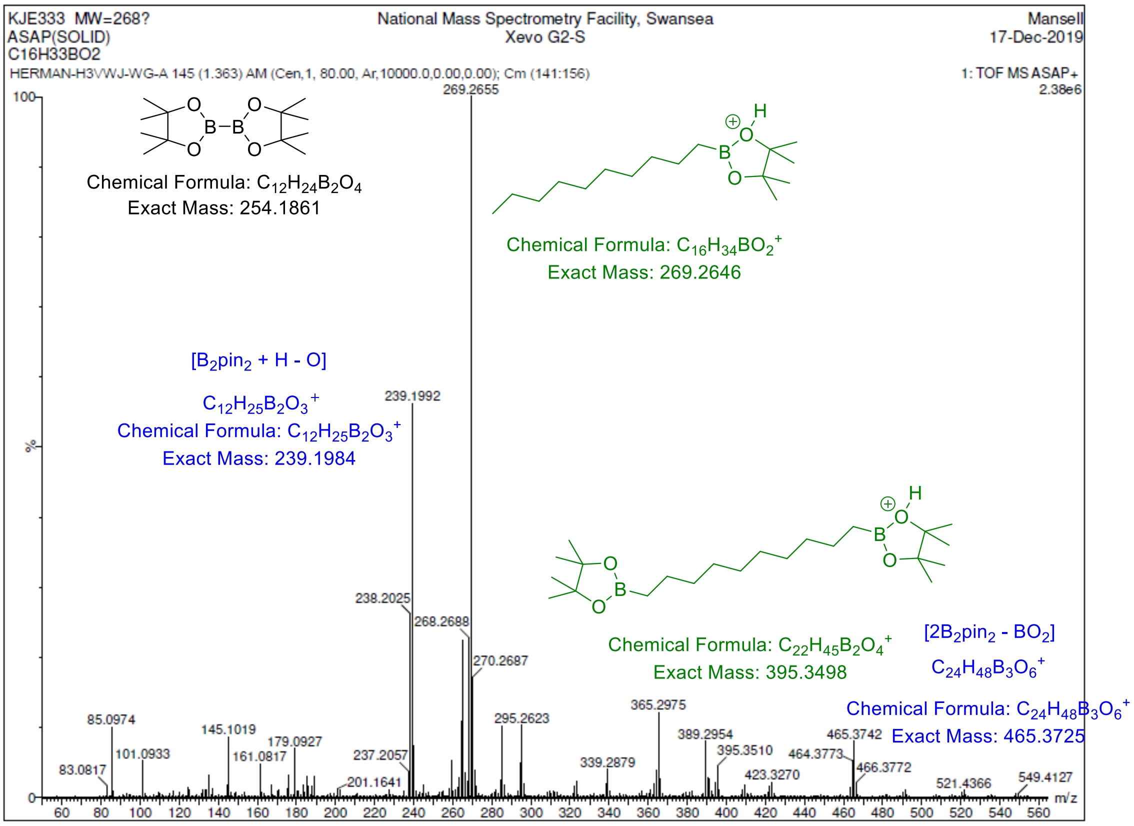

Figure S32. Mass spectrum of decylBpin.

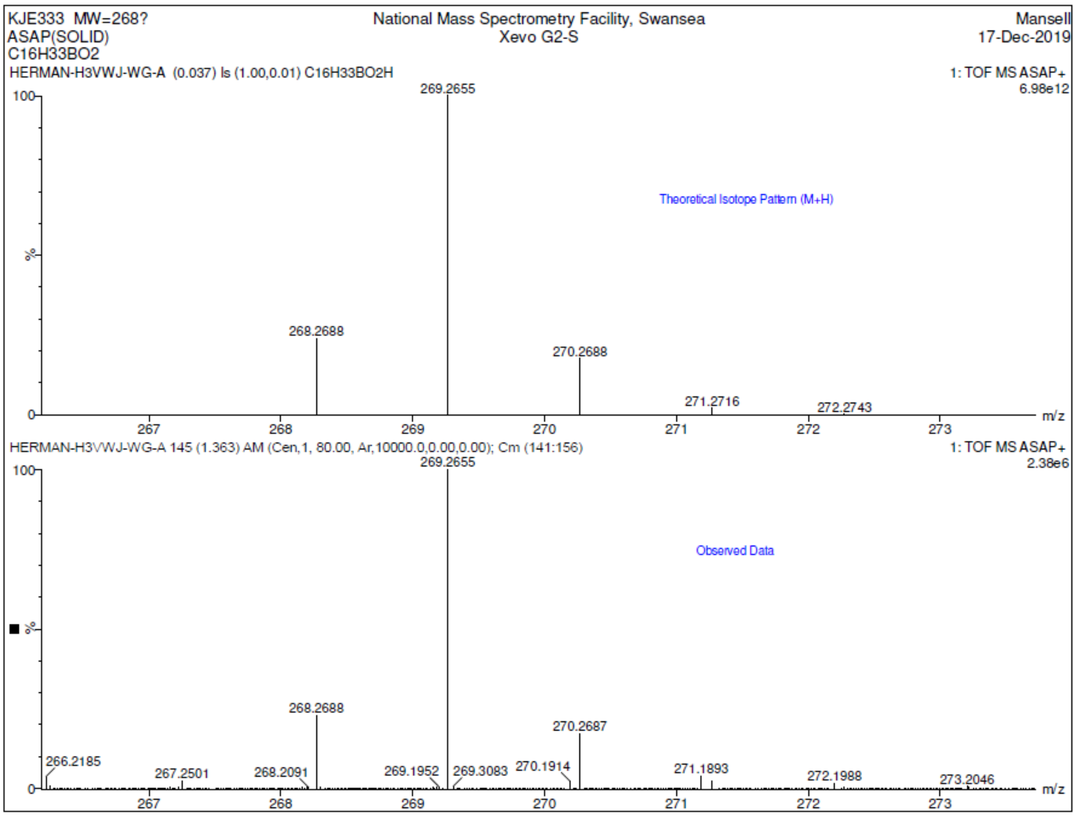

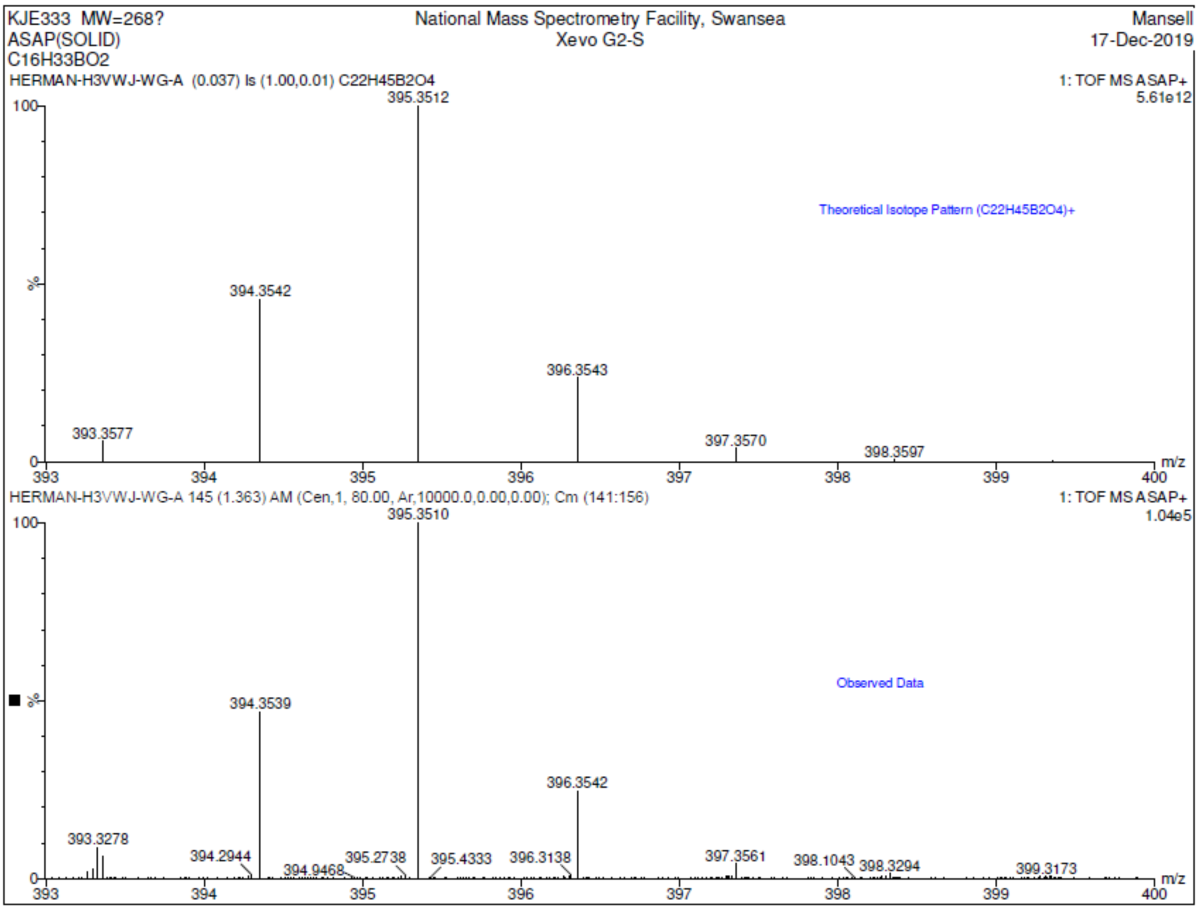

Figure S33. Isotope analysis for decylBpin (top) and diborylated product (bottom).

4.2.10 Borylation of cholestane

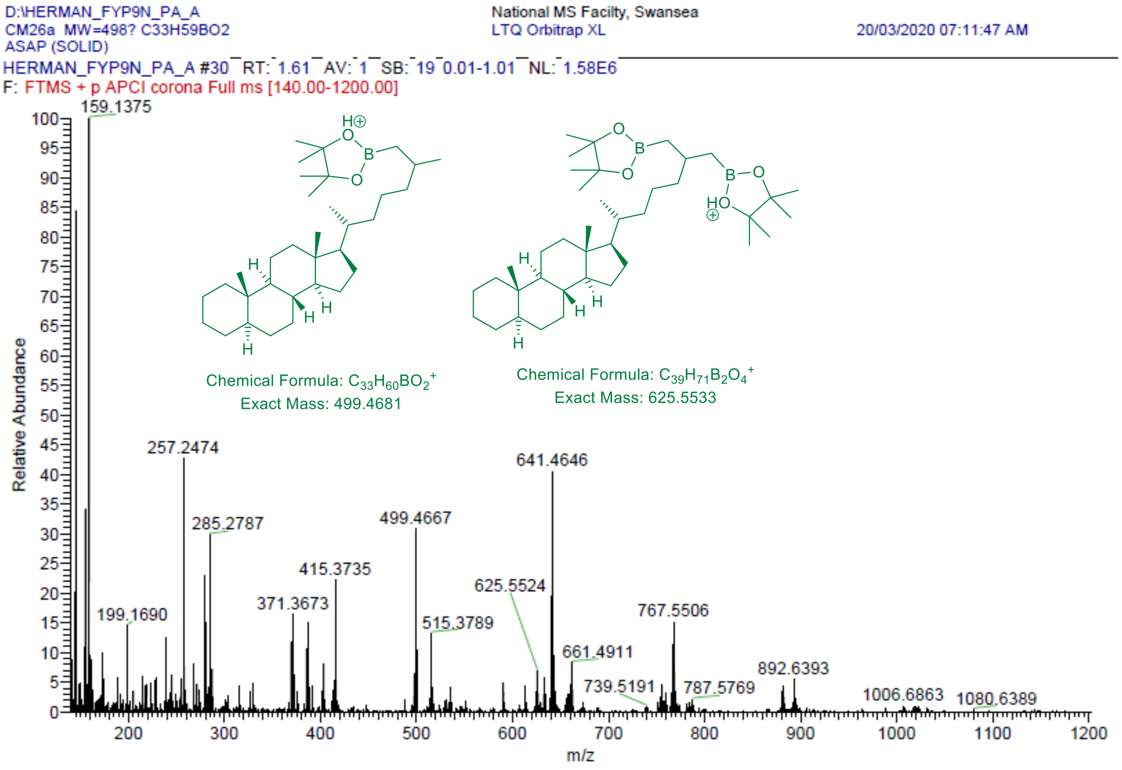

Figure S34. Mass spectrum of cholestylBpin.

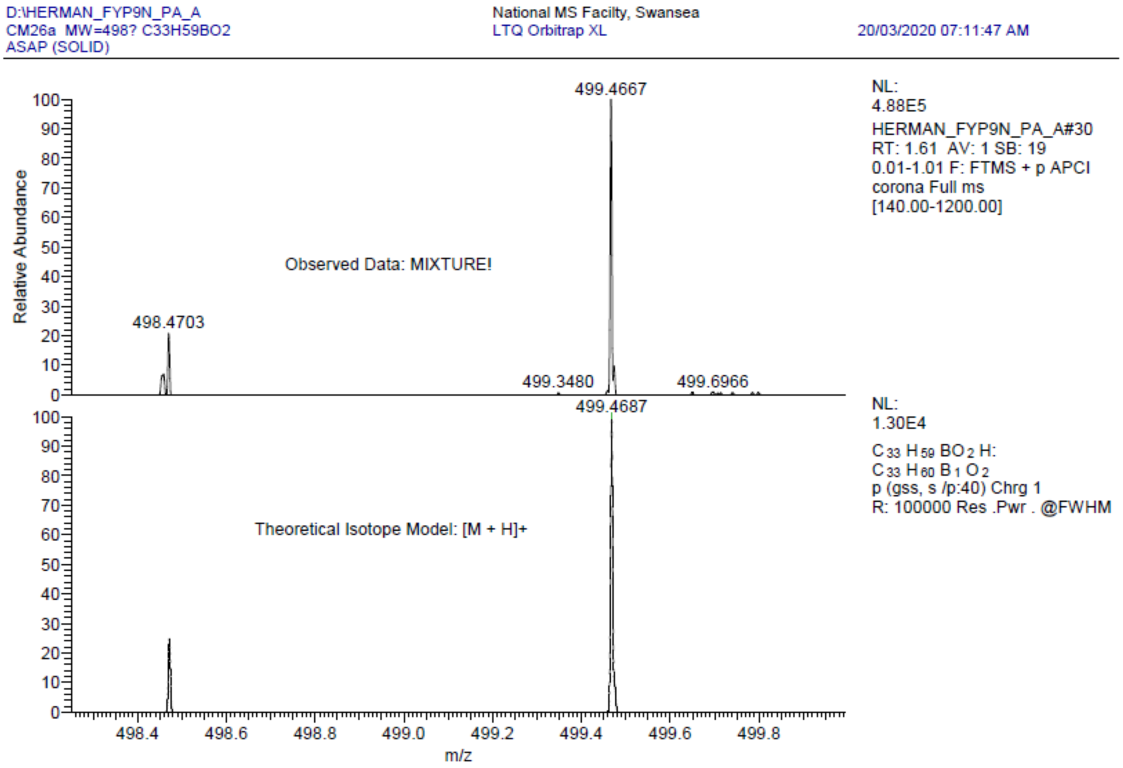

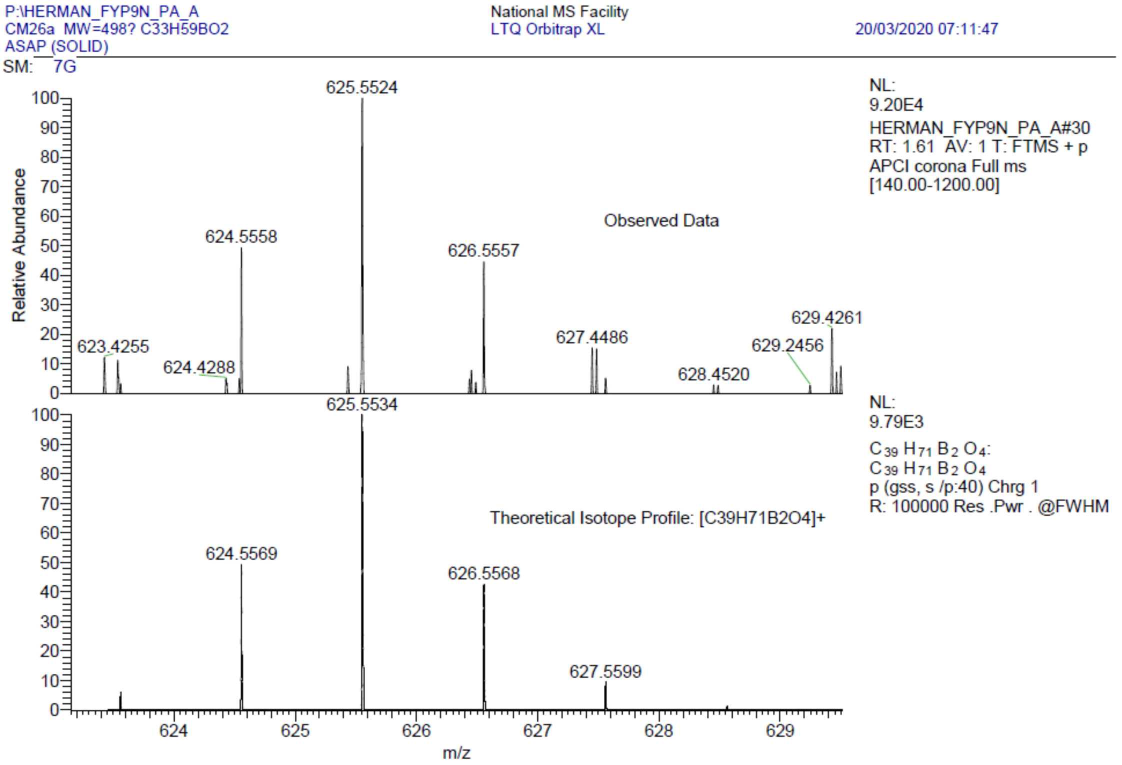

Figure S35. Isotope analysis for cholestylBpin (top) and diborylated product (bottom).

5 NMR spectra

5.1 Monodentate rhodium complexes

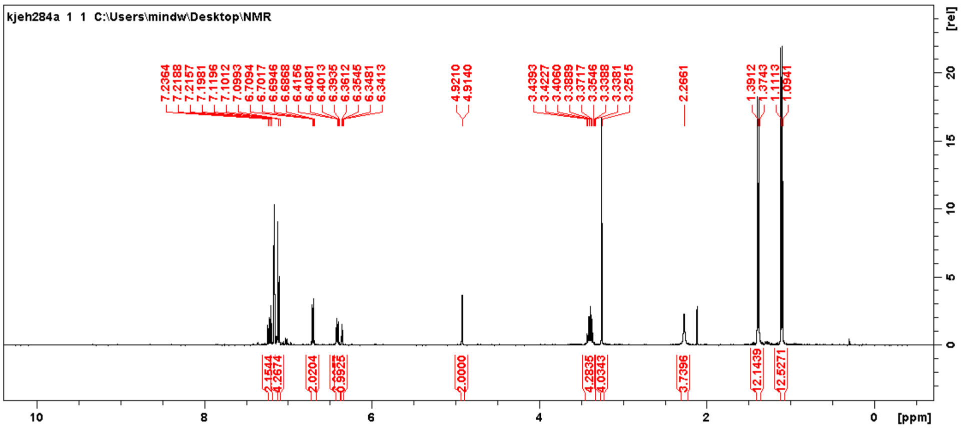

Figure S36. ^{1}H NMR spectrum (400 MHz, C_6D_6, 298 K) of [Rh(Ind)(SIPr)(C_2H_4)] (**1**).

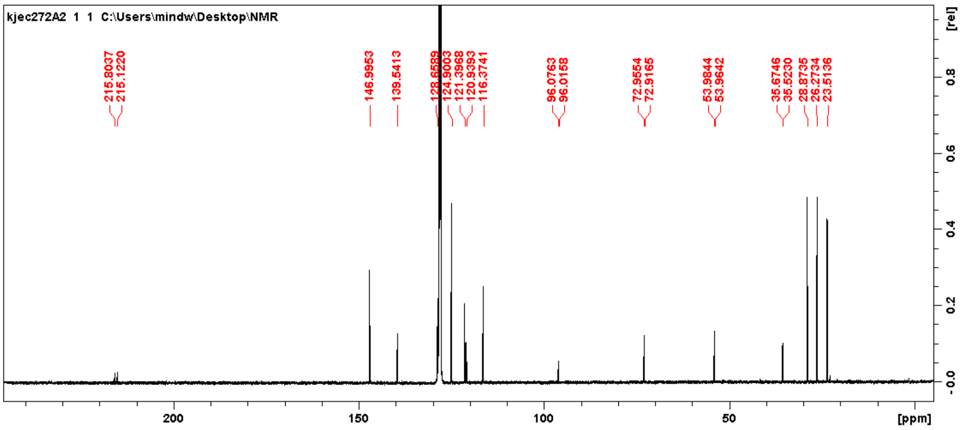

Figure S37. $^{13}C\{^1H\}$ NMR spectrum (101 MHz, C_6D_6, 298 K) of [Rh(Ind)(SIPr)(C_2H_4)] (**1**).

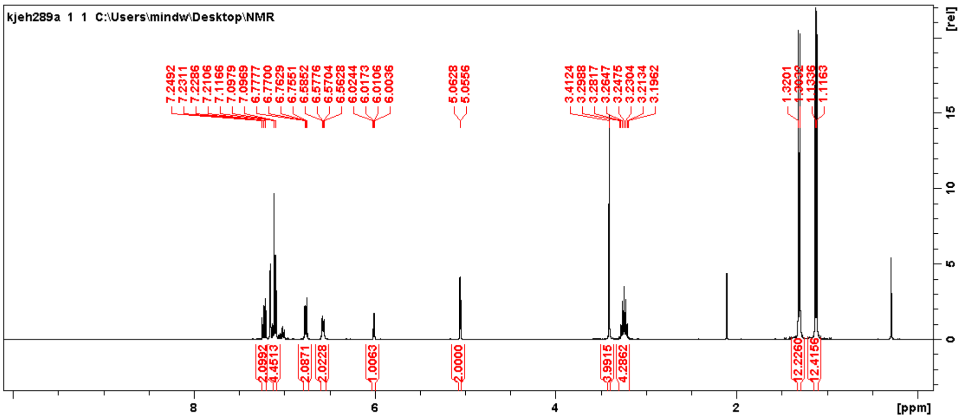

Figure S38. ^{1}H NMR spectrum (400 MHz, C_6D_6, 298 K) of [Rh(Ind)(SIPr)(CO)] (**2a**).

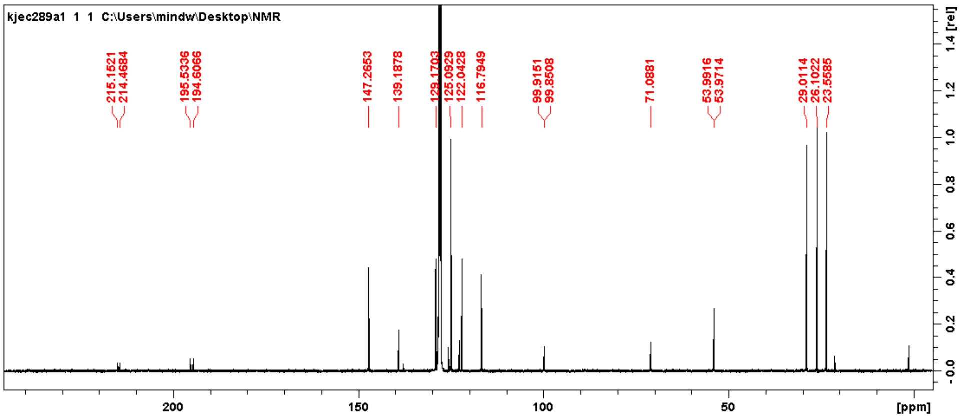

Figure S39. $^{13}C\{^{1}H\}$ NMR spectrum (101 MHz, C_6D_6, 298 K) of [Rh(Ind)(SIPr)(CO)] (**2a**).

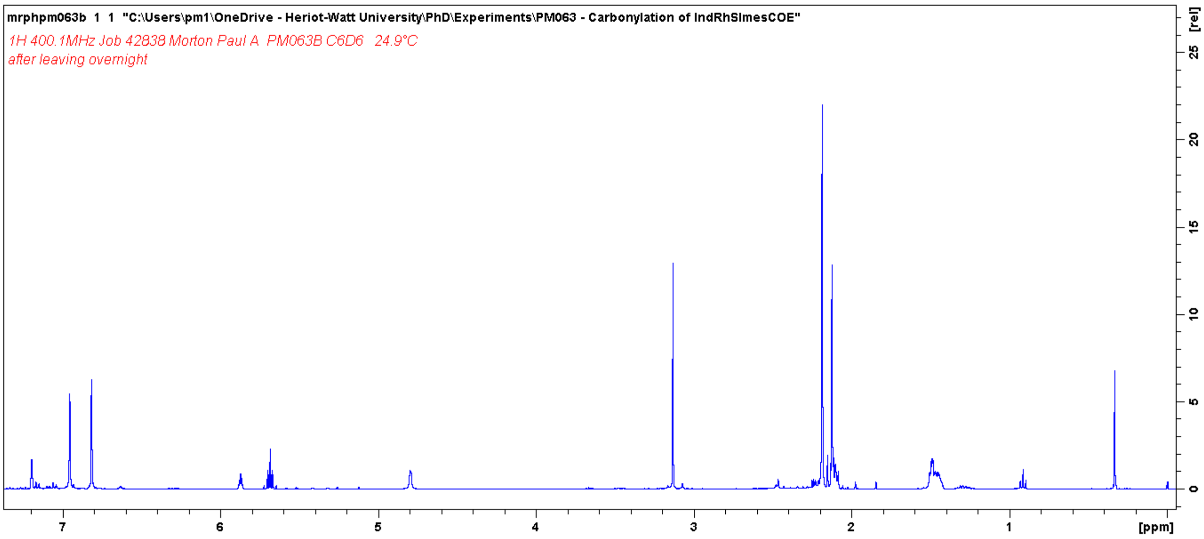

Figure S40. ^{1}H NMR spectrum (400 MHz, C_6D_6, 298 K) of [Rh(Ind)(SIMes)(CO)] (**2b**).

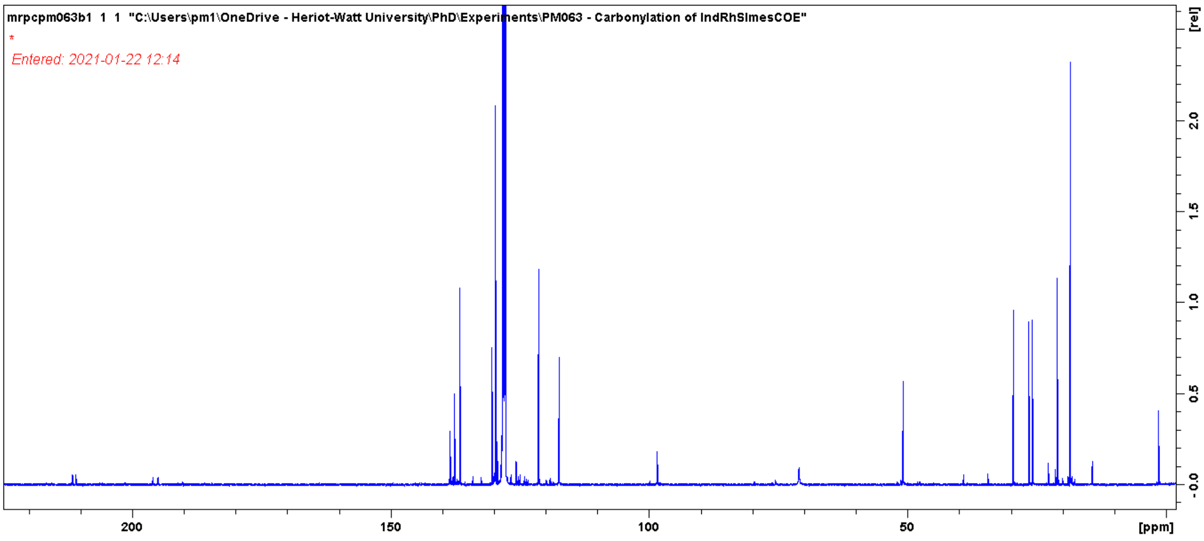

Figure S41. ^{13}C $\{^{1}H\}$NMR spectrum (101 MHz, C_6D_6, 298 K) of [Rh(Ind)(SIMes)(CO)] (**2b**).

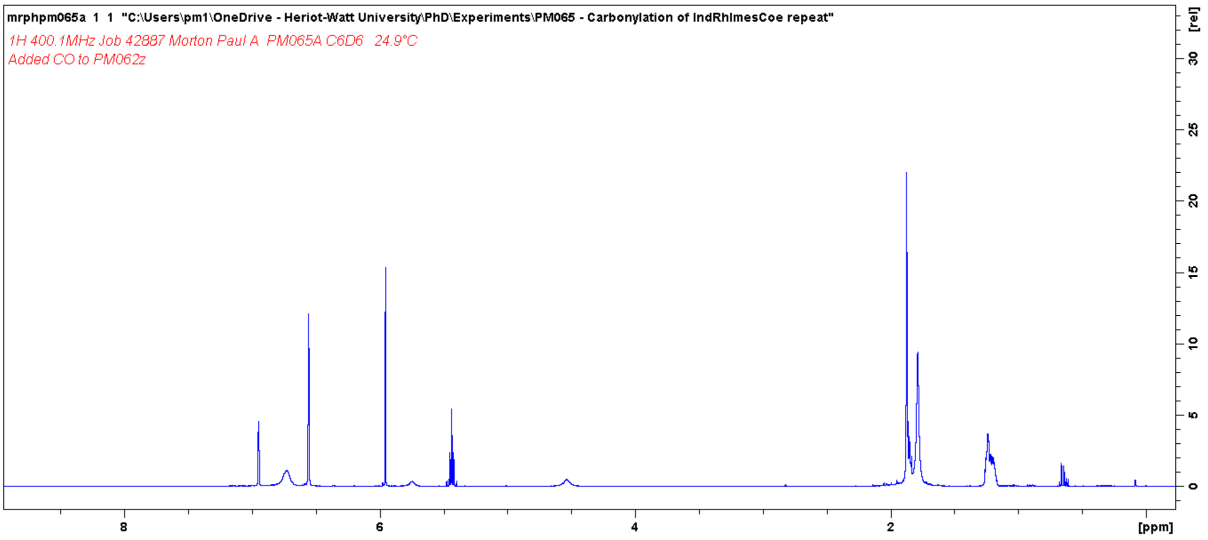

Figure S42. ^{1}H NMR spectrum (400 MHz, C_6D_6, 298 K) of [Rh(Ind)(IMes)(CO)] (**2c**).

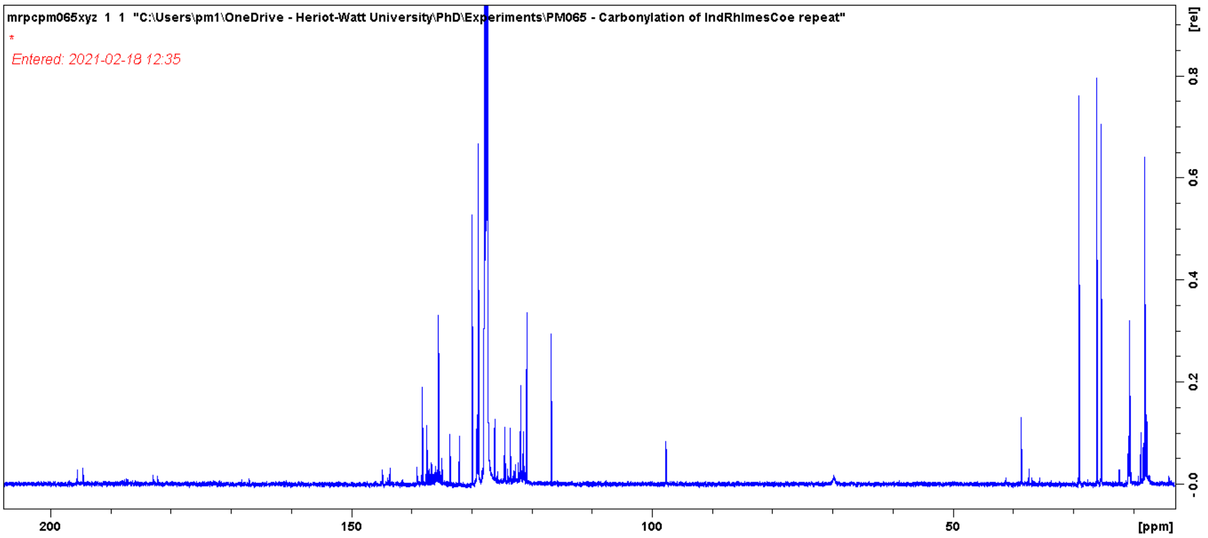

Figure S43. $^{13}C\{^{1}H\}$ NMR spectrum (101 MHz, C_6D_6, 298 K) of [Rh(Ind)(IMes)(CO)] (**2c**).

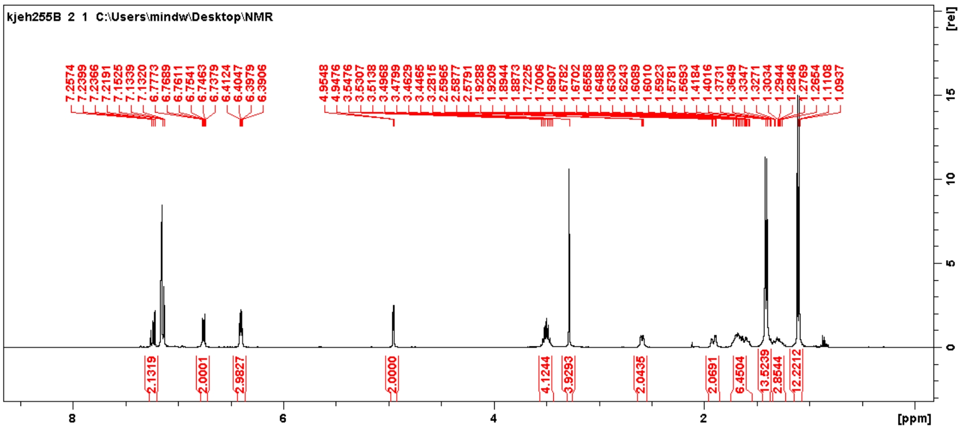

Figure S44. ^{1}H NMR spectrum (400 MHz, C_6D_6, 298 K) of [Rh(Ind)(SIPr)(COE)] (**3**).

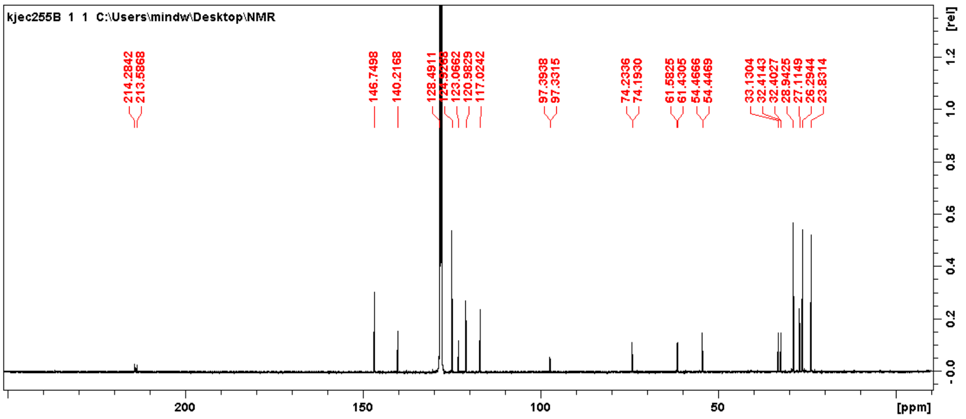

Figure S45. $^{13}C\{^{1}H\}$ NMR spectrum (101 MHz, C_6D_6, 298 K) of [Rh(Ind)(SIPr)(COE)] (**3**).

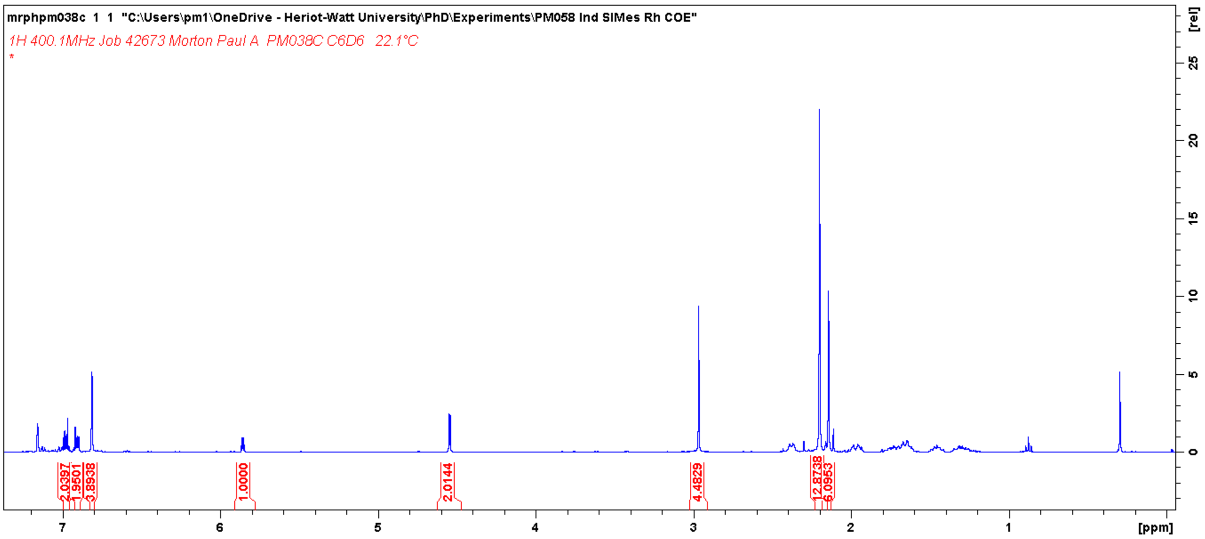

Figure S46. ^{1}H NMR spectrum (400 MHz, C_6D_6, 298 K) of [Rh(Ind)(SIMes)(COE)] (**4**).

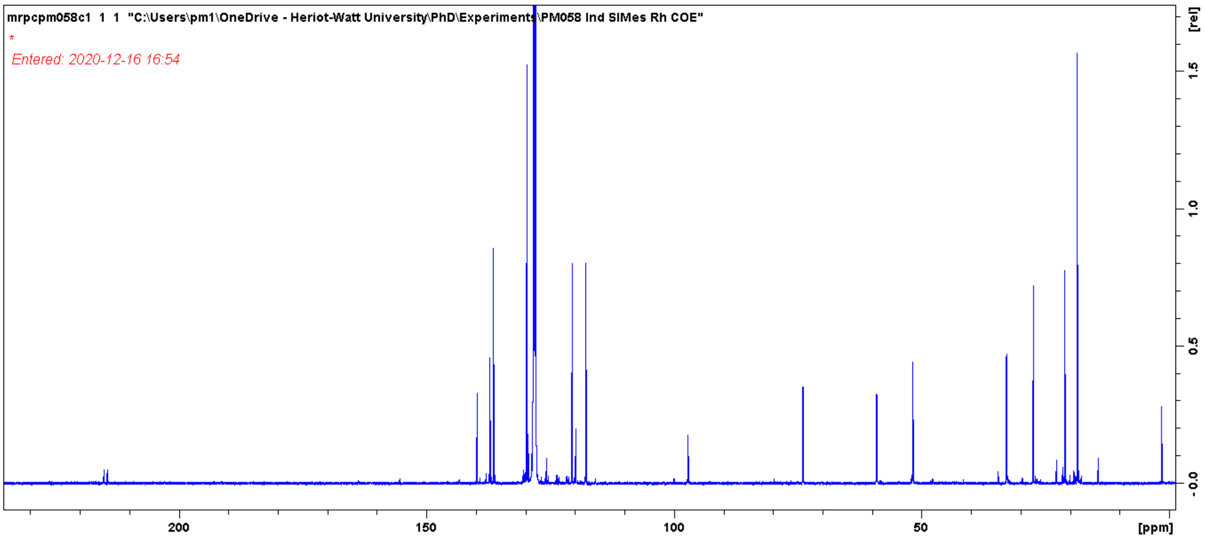

Figure S47. $^{13}C\{^{1}H\}$ NMR spectrum (101 MHz, C_6D_6, 298 K) of [Rh(Ind)(SIMes)(COE)] (**4**).

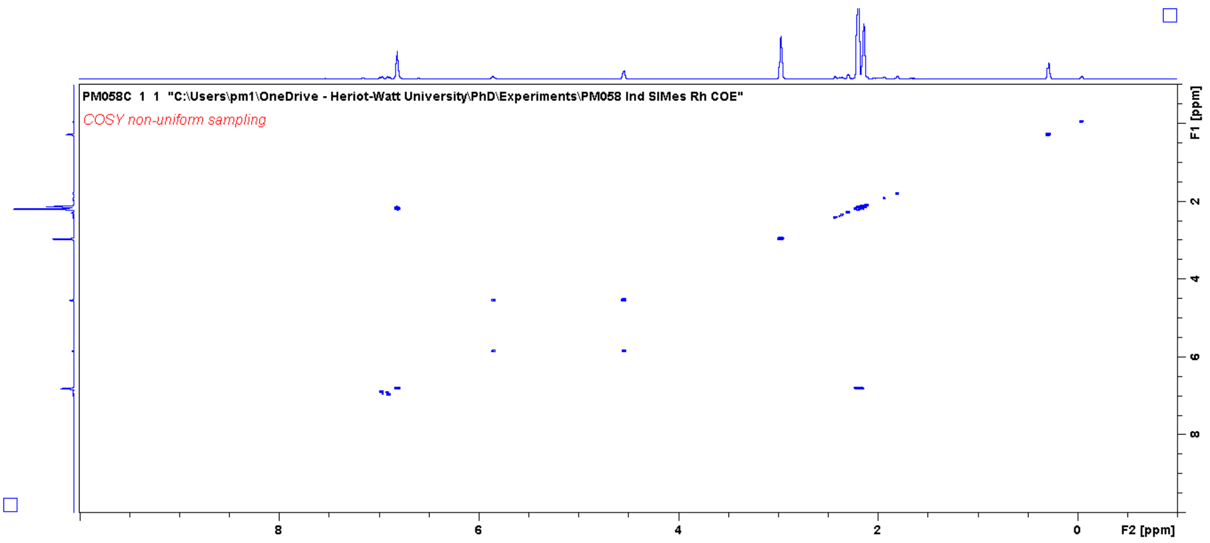
PM058C 1 1 "C:\Users\pm1\OneDrive - Heriot-Watt University\PhD\Experiments\PM058 Ind SIMes Rh COE"
COSY non-uniform sampling
F1 [ppm]
F2 [ppm]

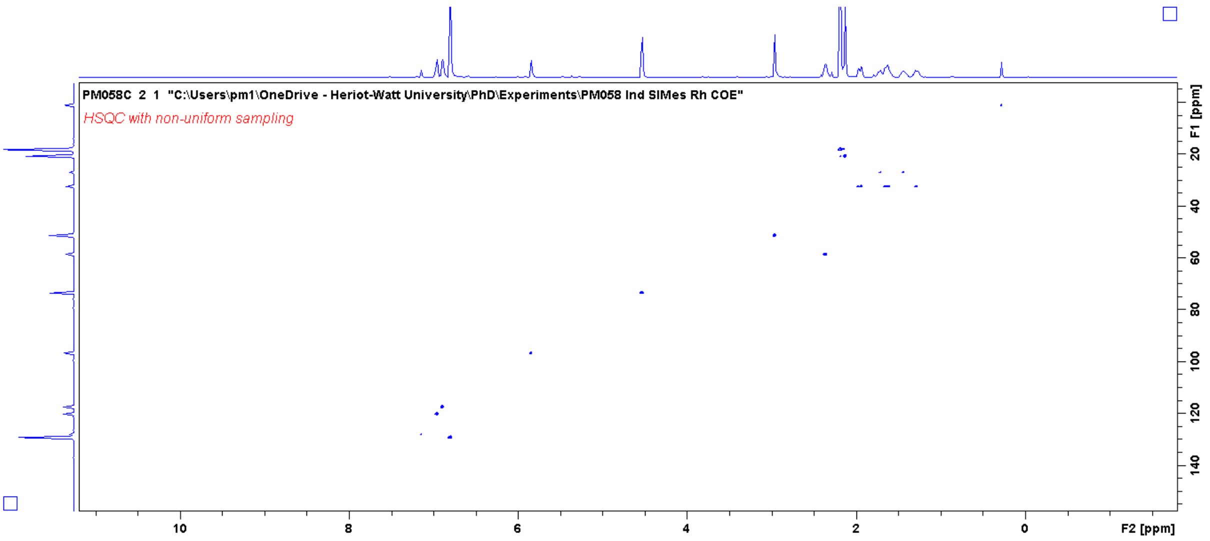
PM058C 2 1 "C:\Users\pm1\OneDrive - Heriot-Watt University\PhD\Experiments\PM058 Ind SIMes Rh COE"
HSQC with non-uniform sampling
F1 [ppm]
F2 [ppm]

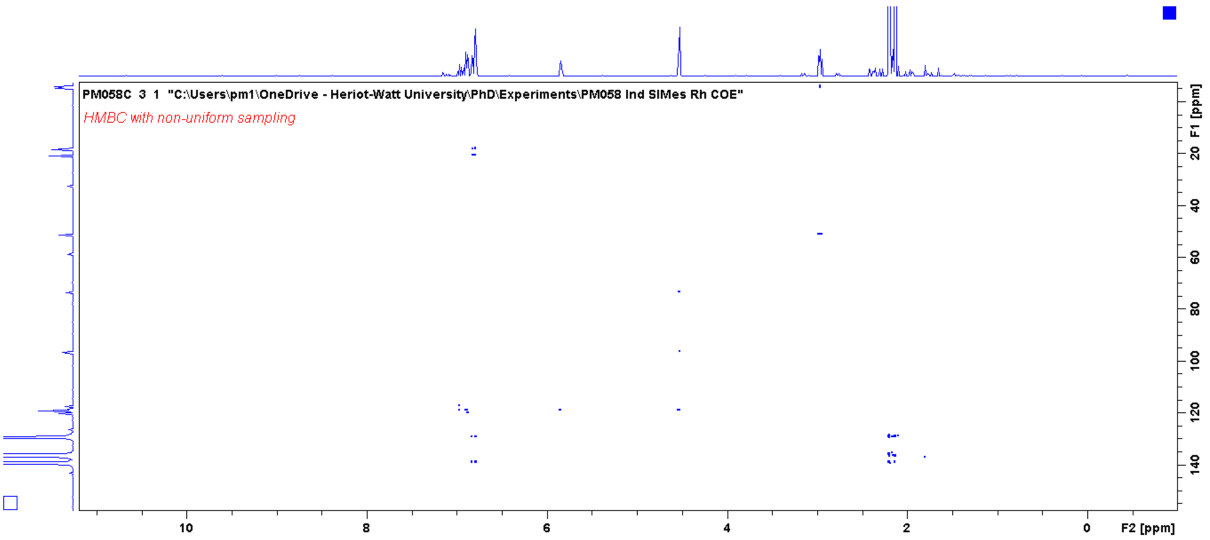
PM058C 3 1 "C:\Users\pm1\OneDrive - Heriot-Watt University\PhD\Experiments\PM058 Ind SIMes Rh COE"
HMBC with non-uniform sampling
F1 [ppm]
F2 [ppm]

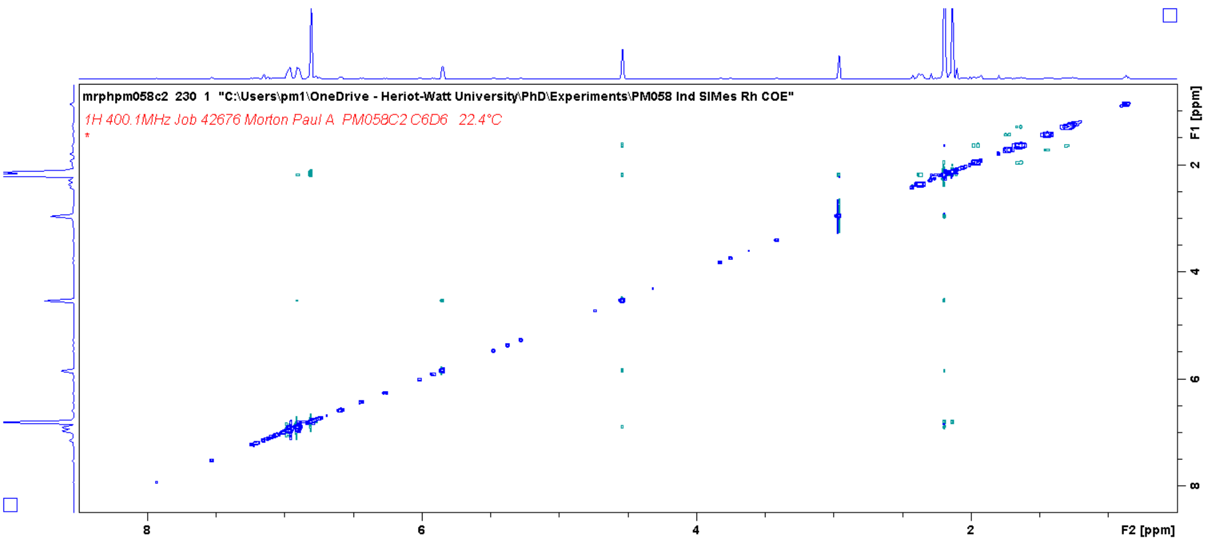

Figure S48. 2D NMR spectra (C_6D_6, 298 K) of [Rh(Ind)(SIMes)(COE)] (**4**).

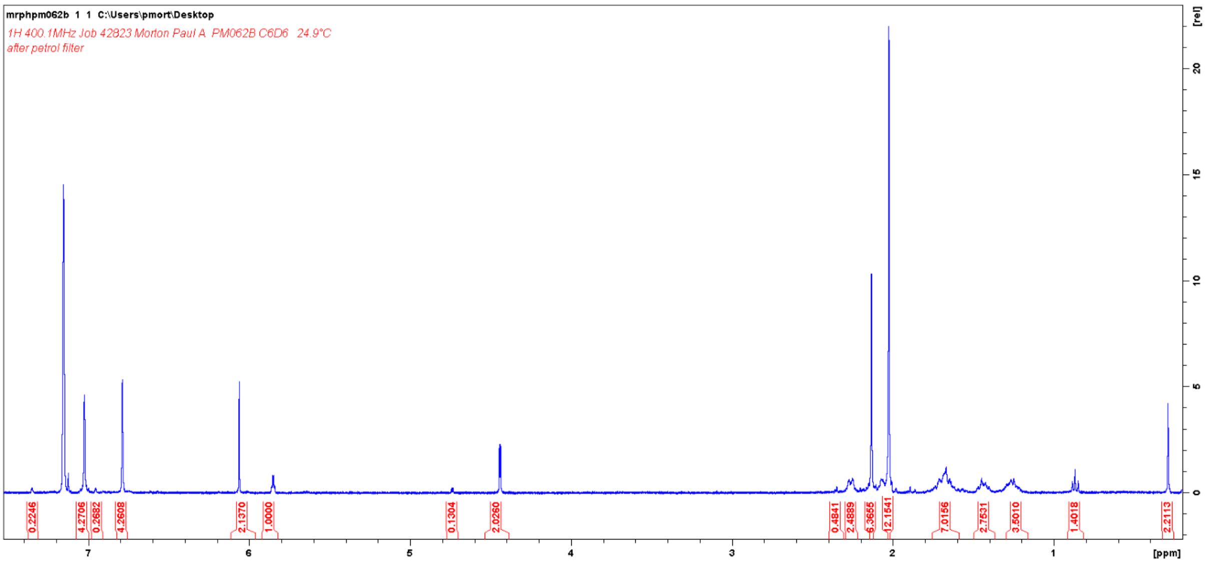

Figure S49. ^{1}H NMR spectrum (400 MHz, C_6D_6, 298 K) of [Rh(Ind)(IMes)(COE)] (**5**).

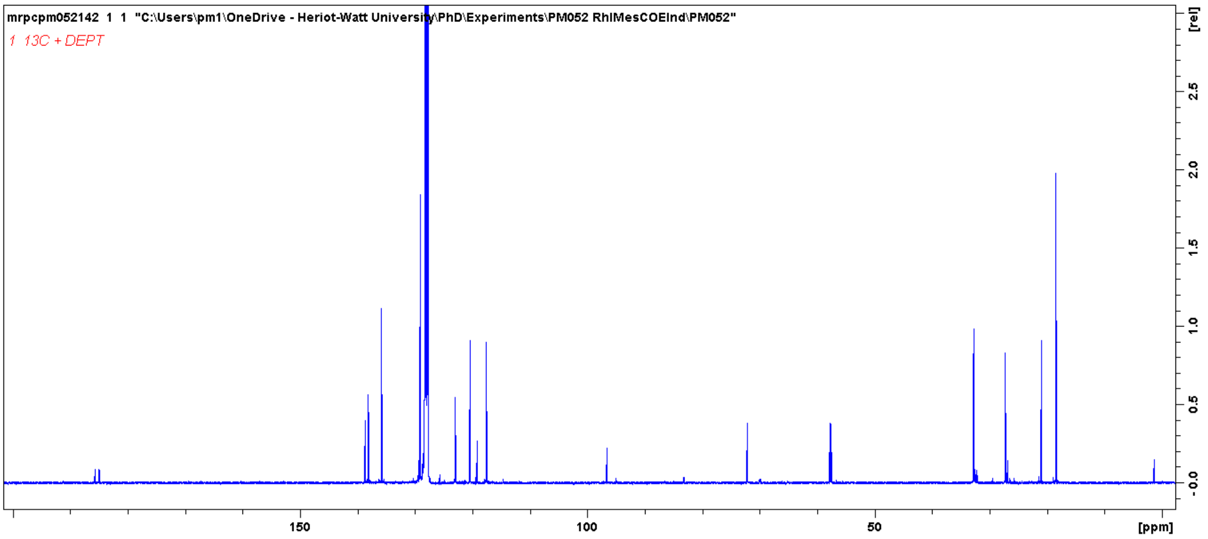

Figure S50. ^{13}C{^{1}H} NMR spectrum (101 MHz, C_6D_6, 298 K) of [Rh(Ind)(IMes)(COE)] (**5**).

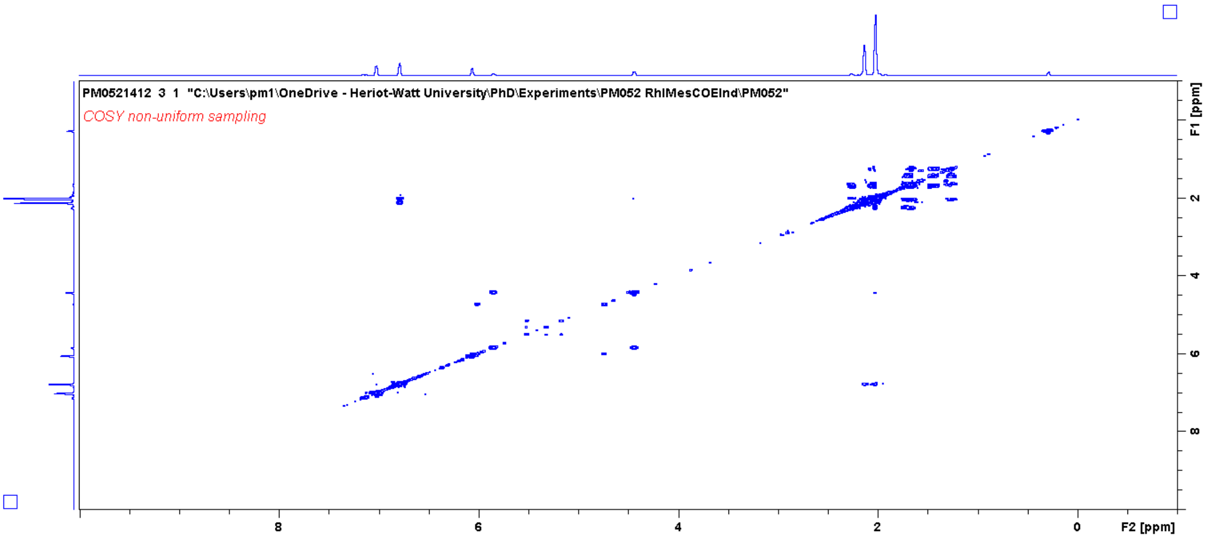
PM0521412 3 1 "C:\Users\pm1\OneDrive - Heriot-Watt University\PhD\Experiments\PM052 RhIMesCOEInd\PM052"
COSY non-uniform sampling
F1 [ppm]
F2 [ppm]

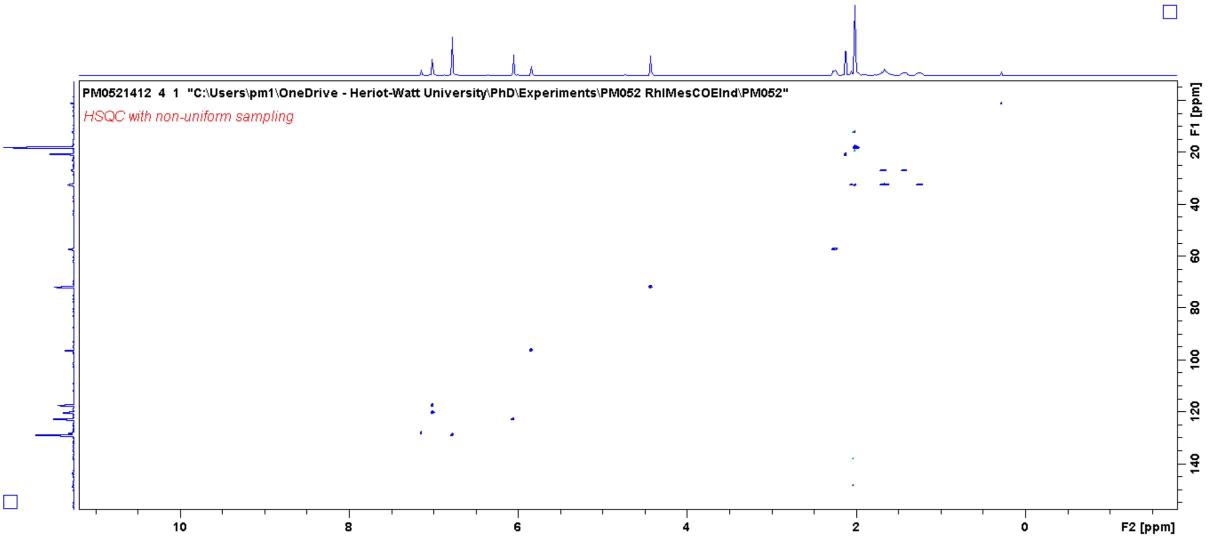
PM0521412 4 1 "C:\Users\pm1\OneDrive - Heriot-Watt University\PhD\Experiments\PM052 RhIMesCOEInd\PM052"
HSQC with non-uniform sampling
F1 [ppm]
F2 [ppm]

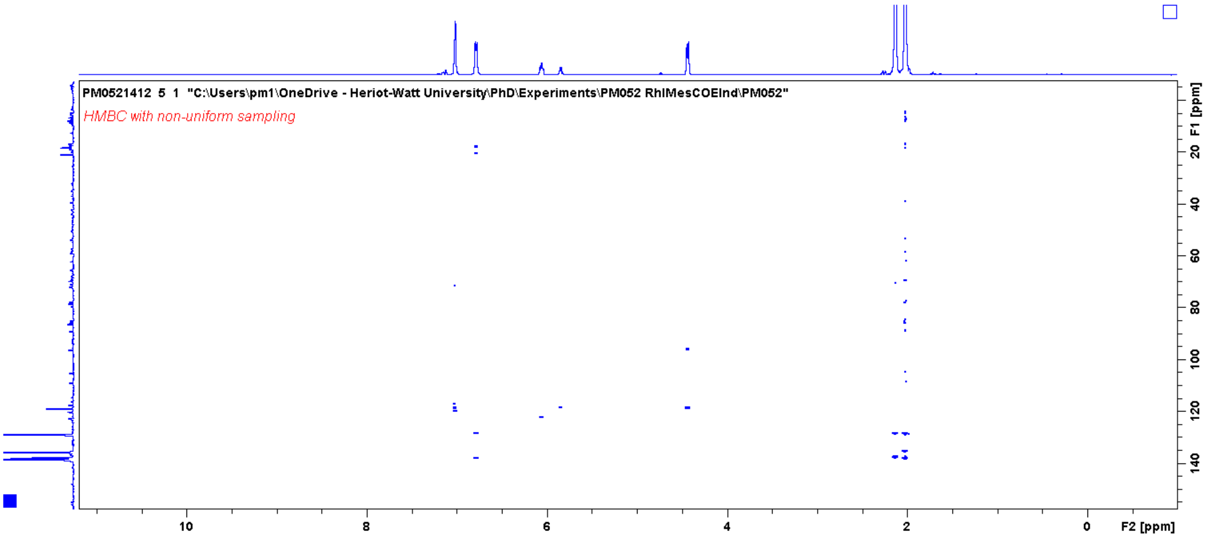
PM0521412 5 1 "C:\Users\pm1\OneDrive - Heriot-Watt University\PhD\Experiments\PM052 RhIMesCOEInd\PM052"
HMBC with non-uniform sampling
F1 [ppm]
F2 [ppm]

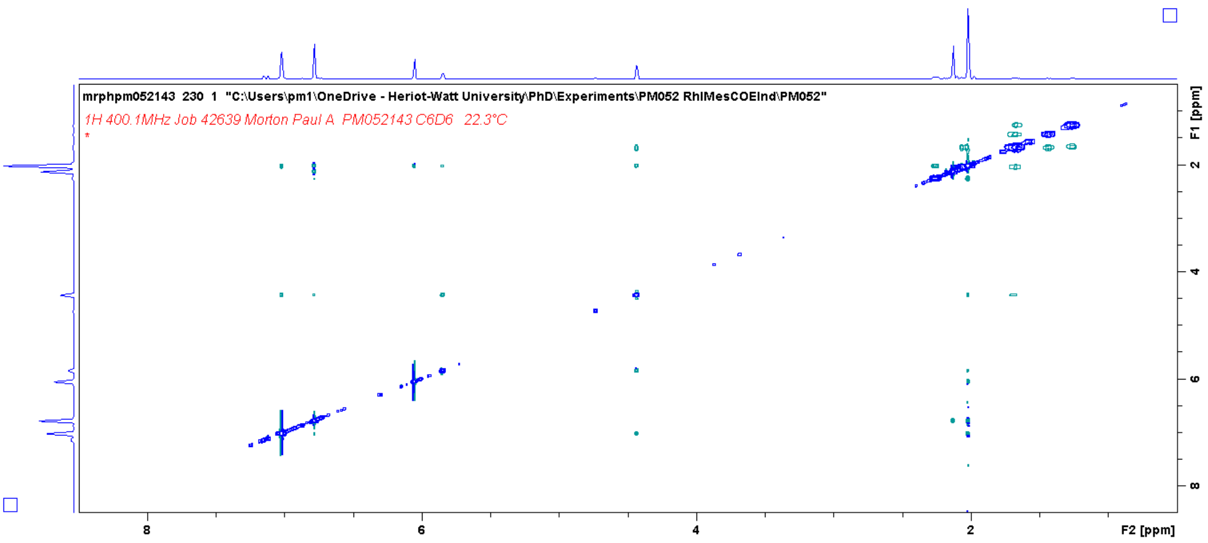

Figure S51. 2D NMR spectra (101 MHz, C_6D_6, 298 K) of [Rh(Ind)(IMes)(COE)] (**5**).

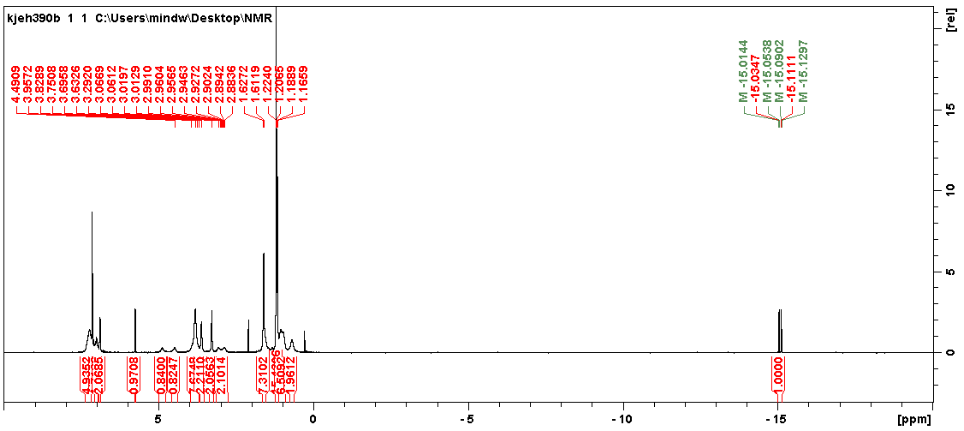

Figure S52. ^{1}H NMR spectrum (400 MHz, C_6D_6, 298 K) of [Rh(Ind){Si(OEt)$_3$}(H)(SIPr)] (**6**).

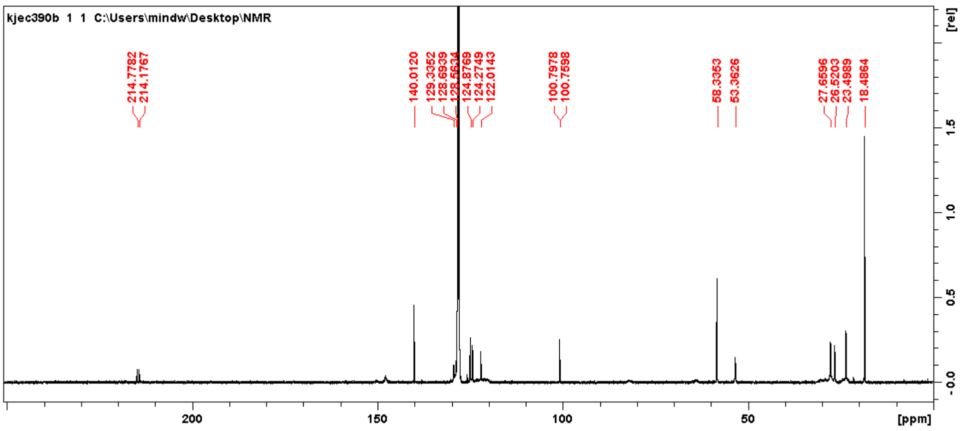

Figure S53. $^{13}C\{^{1}H\}$ NMR spectrum (101 MHz, C_6D_6, 298 K) of [Rh(Ind){Si(OEt)$_3$}(H)(SIPr)] (**6**).

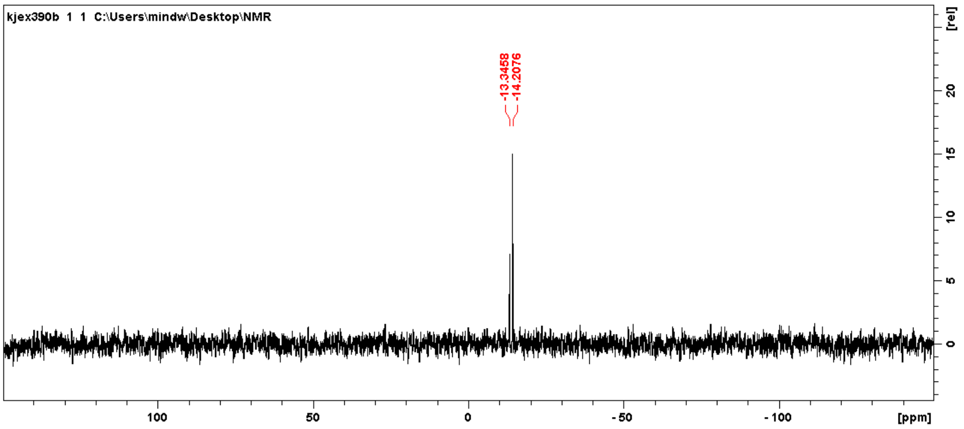

Figure S54. $^{29}Si\{^1H\}$ NMR spectrum (MHz, C_6D_6, 298 K) of [Rh(Ind){Si(OEt)$_3$}(H)(SIPr)] (**6**).

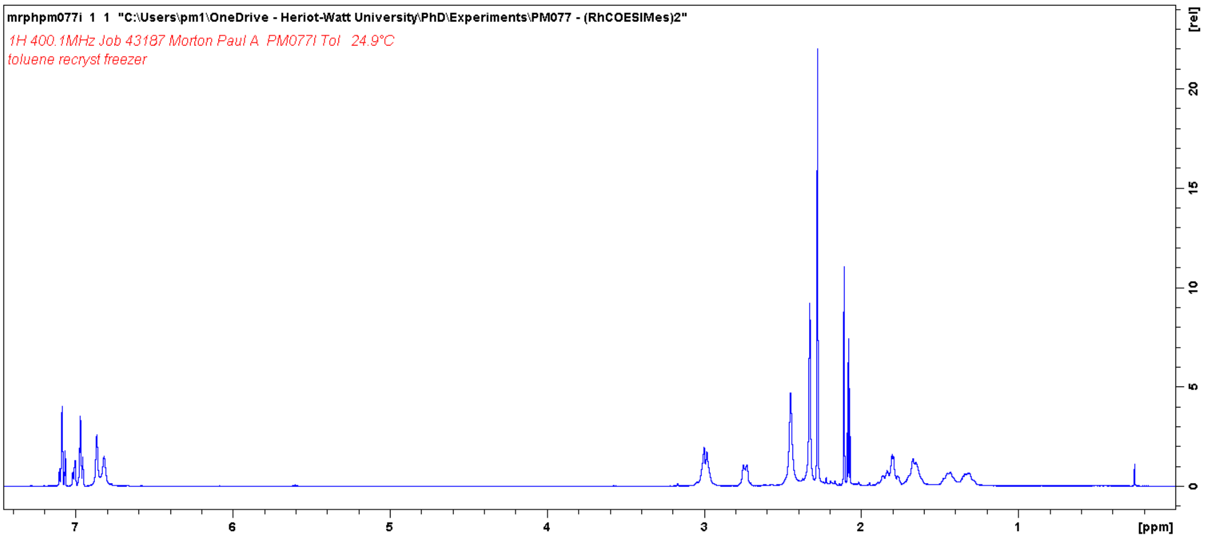

Figure S55. ^1H NMR spectrum (400 MHz, toluene-d_8, 298 K) of [Rh(μ-Cl)(SIMes)(COE)]$_2$ (**8**).

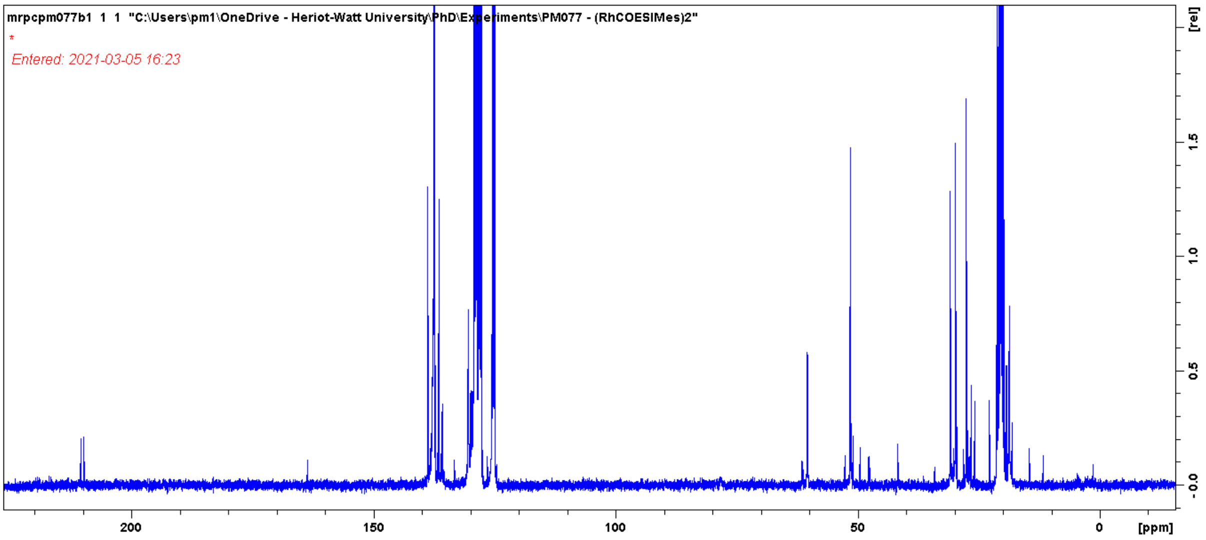

Figure S56. $^{13}C\{^1H\}$ NMR spectrum (101 MHz, toluene-d_8, 298 K) of [Rh(μ-Cl)(SIMes)(COE)]$_2$ (**8**).

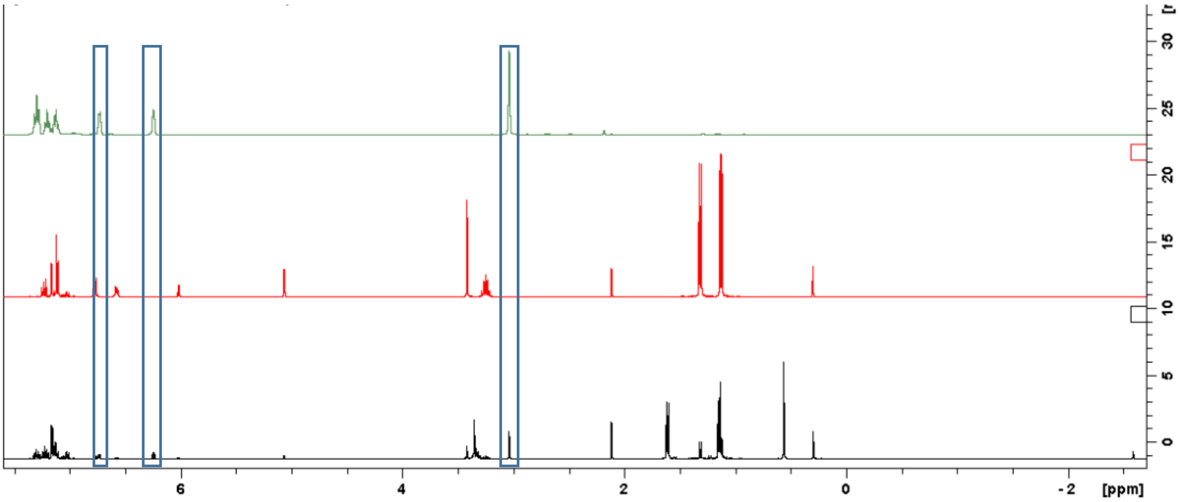

Figure S57. Stack plot of ^{1}H NMR spectra for indene in C_6D_6 (top, green), **2a** (middle, red) and **9** (bottom, black). The 3 resonances associated with the 5 membered ring of indene are highlighted, indicating its formation.

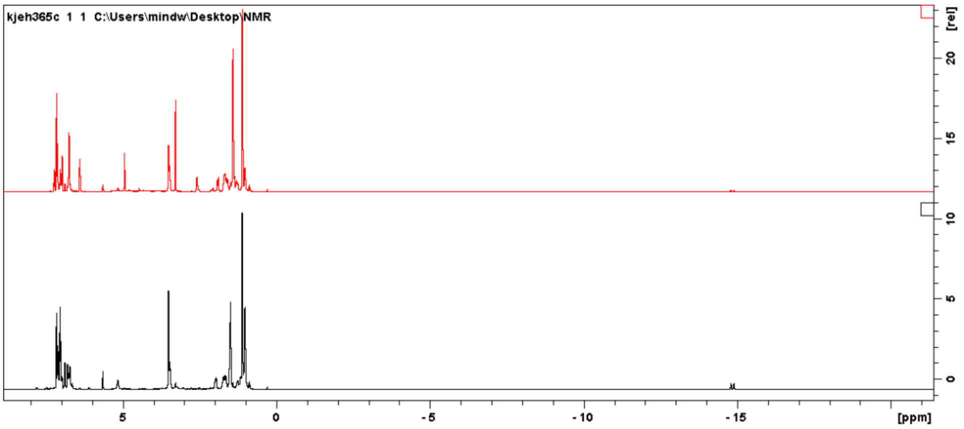

Figure S58. Stack plot of the ^{1}H NMR spectra for the reaction of **3** with 3 equiv. HBcat a) on mixing (top, red) and b) overnight at rt.(black, bottom).

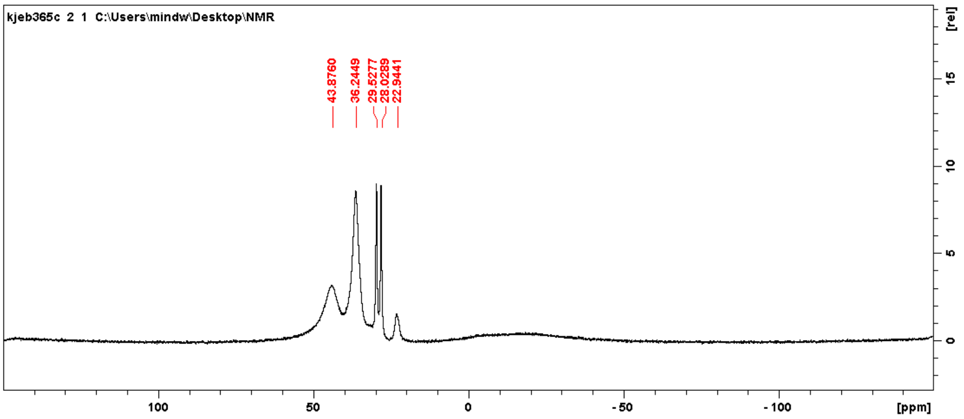

Figure S59. ^{11}B NMR spectrum (128 MHz, C_6D_6, 298 K) for the reaction of **3** with 3 eqiv. HBcat after overnight at room temperature. The doublet resonance at δ = 29 ppm is excess/unreacted HBcat.

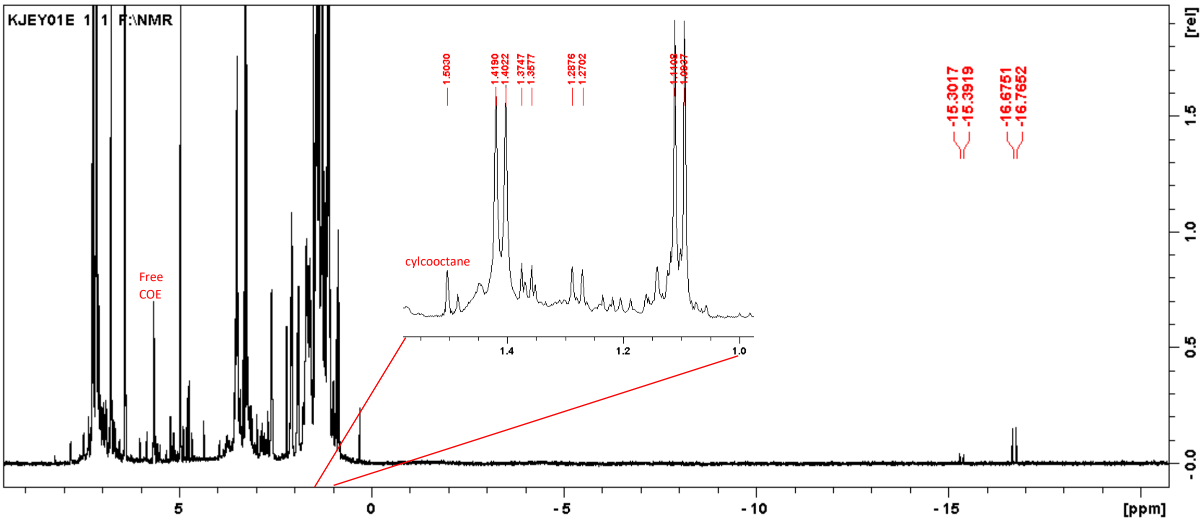

Figure S60. ^{1}H NMR spectrum (400 MHz, C_6D_6, 298K) of **3** after 4 hrs of photolysis. The major species is still **3**, but two new Rh hydride species are now present. Free COE is observed at 5.6 ppm and cyclooctane is observed at 1.5 ppm.

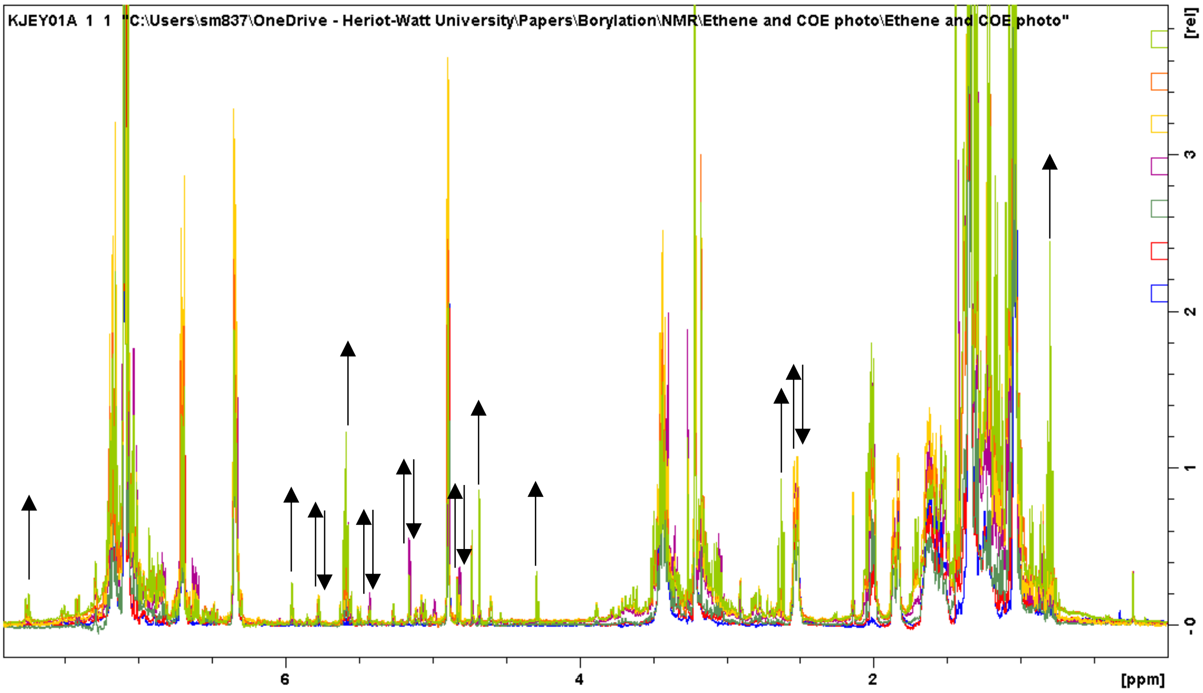

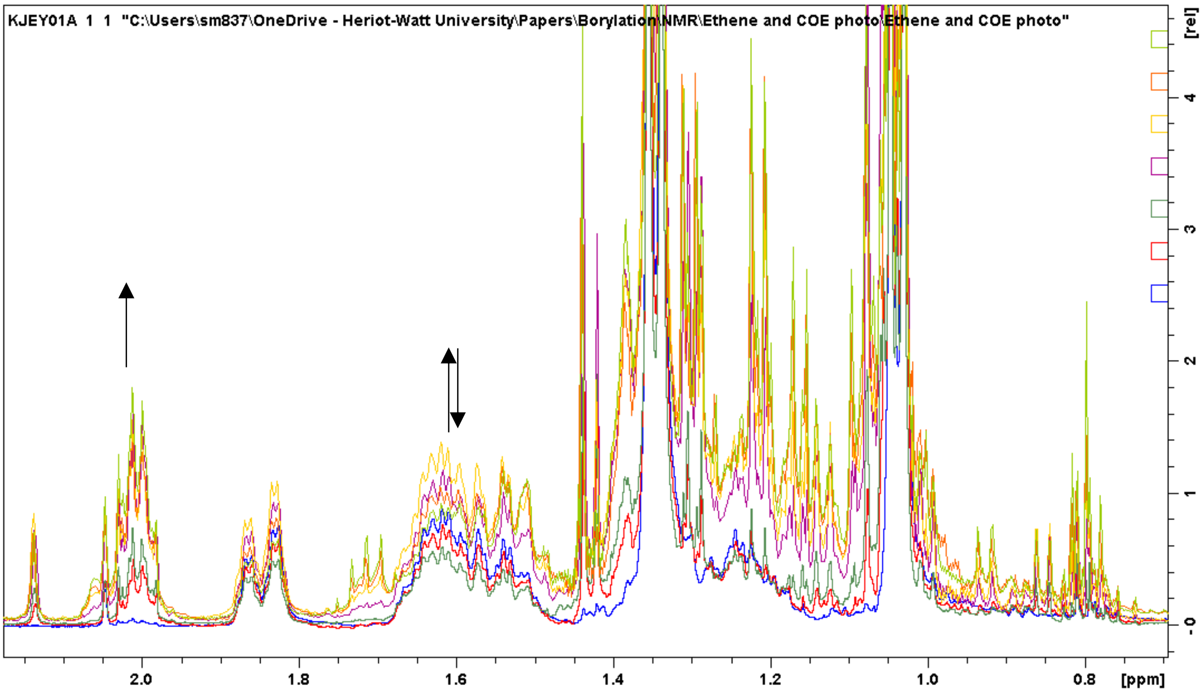

Figure S61. Stack plot of ^1H NMR spectra from the photolysis reactions of **3** (top spectrum). Bottom spectrum shows an enlargement of the alkyl region. Some resonances grow in (arrows pointing up), some resonances grow in then decrease in intensity (arrows pointing up and down).

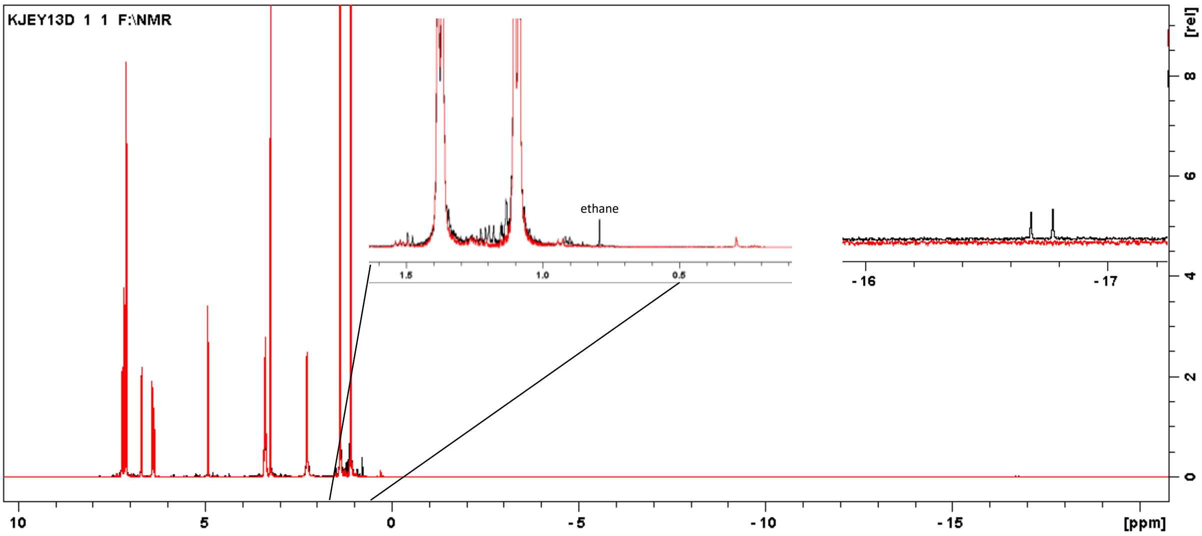

Figure S62. Comparison of the^{1}H NMR spectra (400 MHz, C_6D_6, 298K) of **1** before (red) and after (black) 4 hrs of photolysis. The major species is still **1**, but a new Rh hydride species are now present. Ethane is observed at 0.8 ppm.

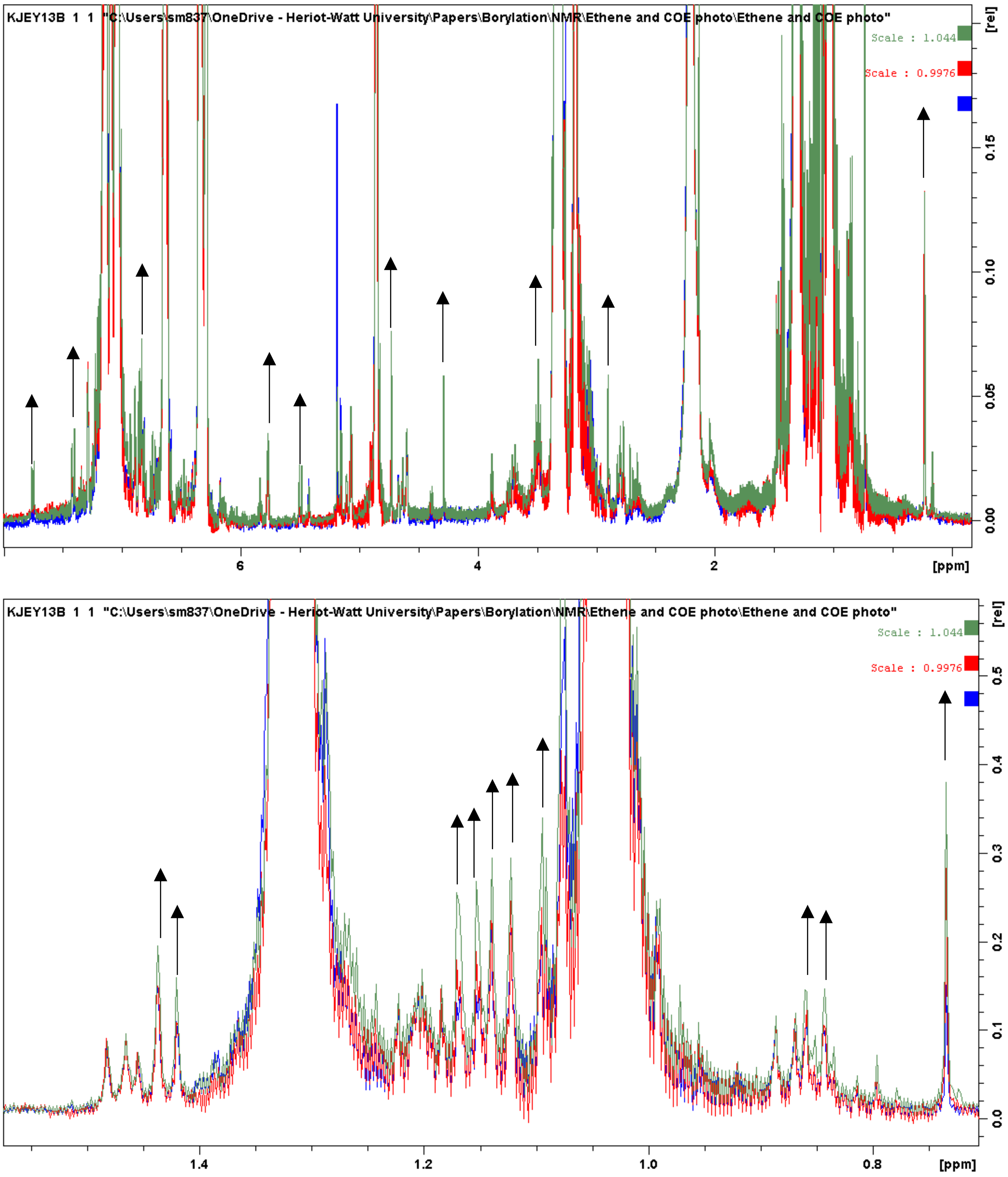

Figure S63. Stack plot of the^{1}H NMR spectra (400 MHz, C_6D_6, 298K) of **1** undergoing photolysis (top, bottom spectrum is enlargement on alkyl region).

5.2 Fluorenyl-tethered Rh complexes

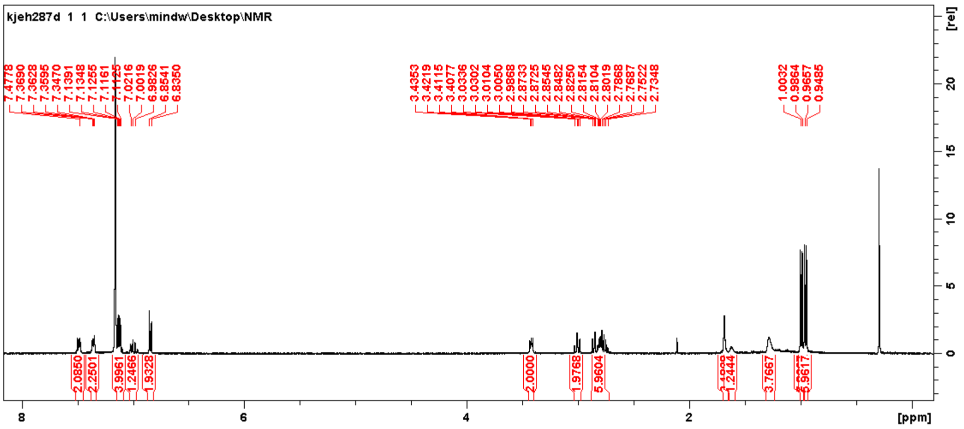

Figure S64. ^{1}H NMR spectrum (400 MHz, C_6D_6, 298 K) of [Rh(Flu-Dipp)(C_2H_4)] (**11**)

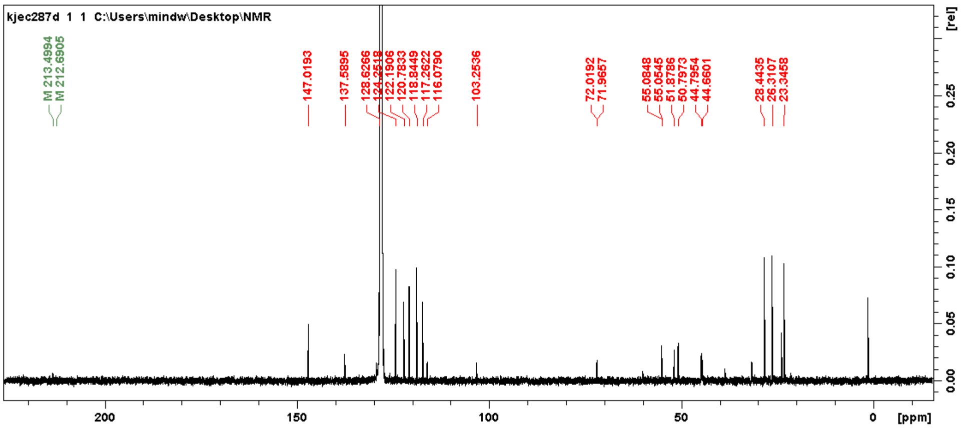

Figure S65. $^{13}C\{^{1}H\}$ NMR spectrum (101 MHz, C_6D_6, 298 K) of [Rh(Flu-Dipp)(C_2H_4)] (**11**)

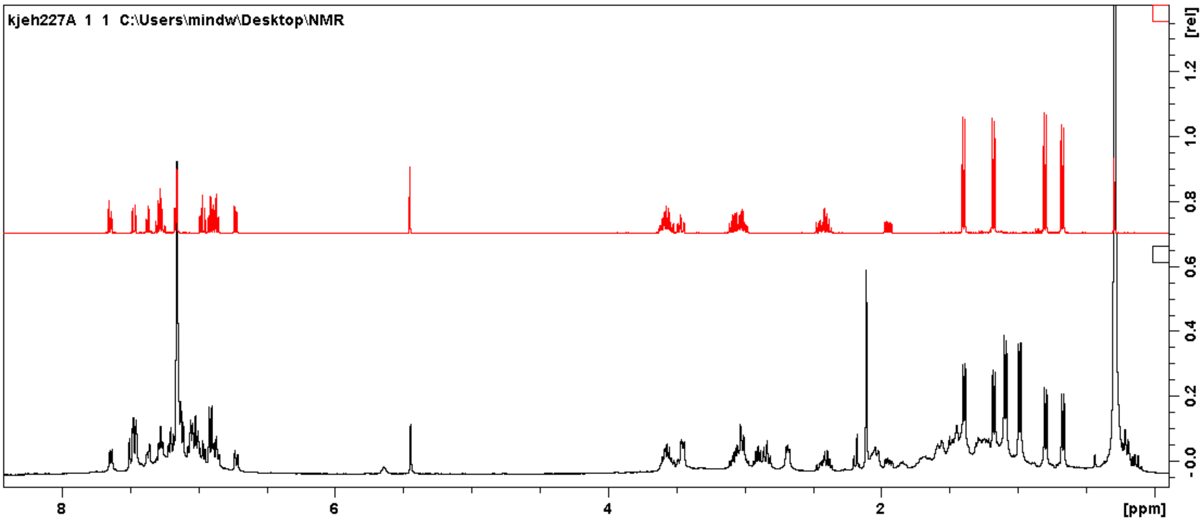

Figure S66. Comparison of ^{1}H NMR spectra for the crude reaction mixture from the synthesis of [Rh(Flu-Dipp)(COE)] (**12**) (bottom, black) with spiro[($C_{13}H_8$)C_2H_4N(CH)N(C_2H_4)(Dipp)] (top, red), which is the major impurity in the reaction mixture.

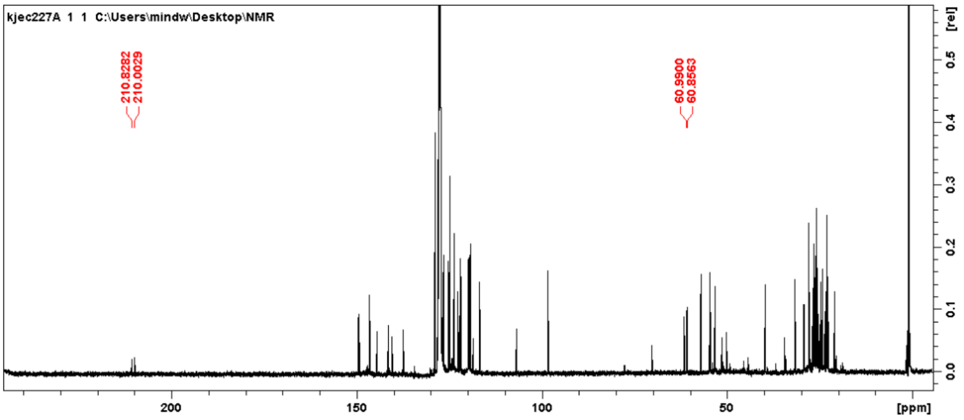

Figure S67. $^{13}C\{^{1}H\}$ NMR spectrum (101 MHz, C_6D_6, 298 K) of crude reaction mixture from the synthesis of [Rh(Flu-Dipp)(COE)] (**12**). Highlighted are the key resonances attributed to the product.

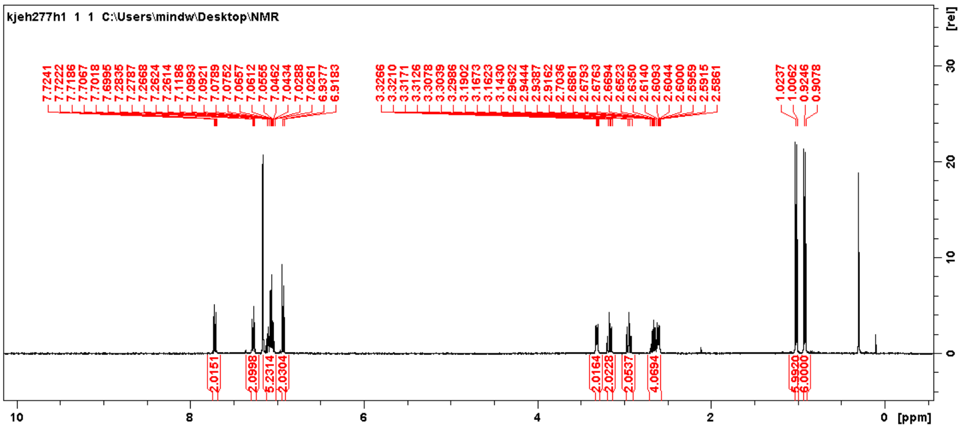

Figure S68. ^{1}H NMR spectrum (400 MHz, C_6D_6, 298 K) of [Rh(Flu-Dipp)(CO)] (**13**)

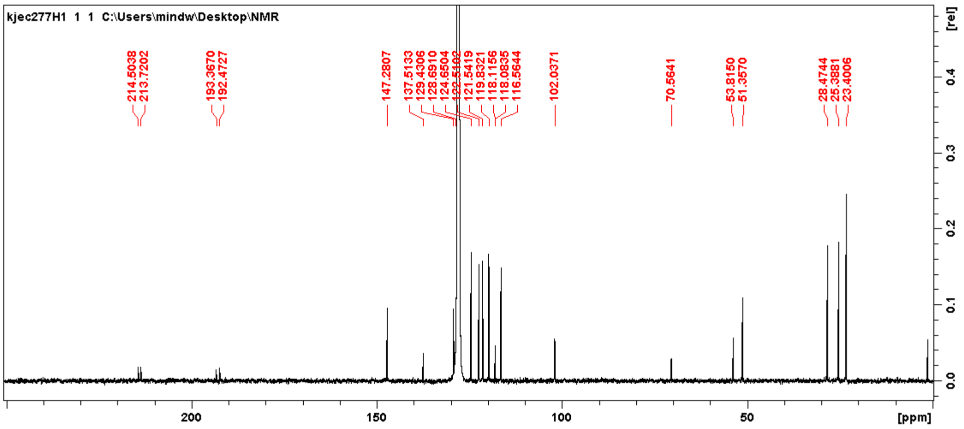

Figure S69. ^{13}C NMR spectrum (101 MHz, C_6D_6, 298 K) of [Rh(Flu-Dipp)(CO)] (**13**).

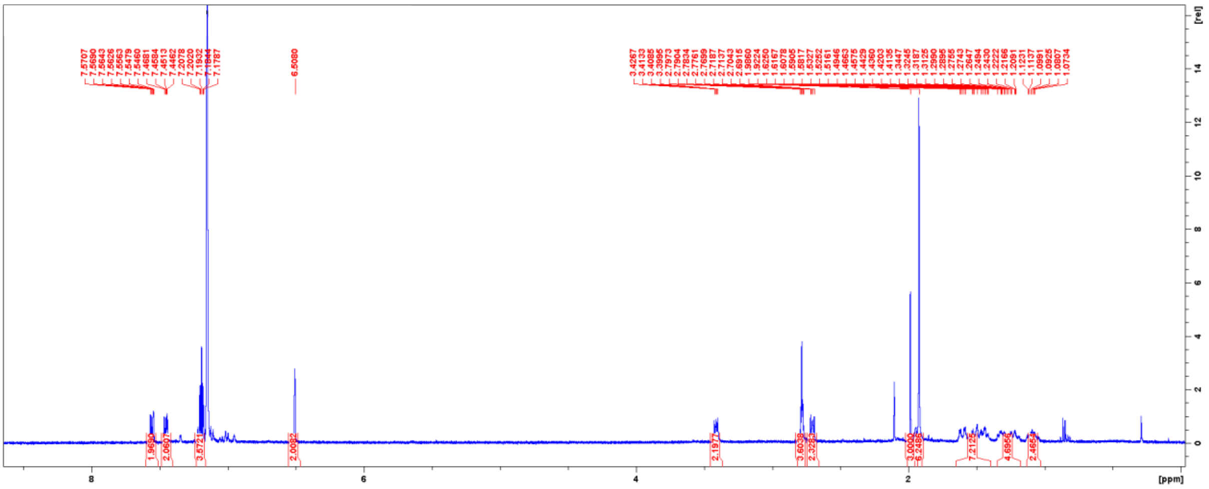

Figure S70. ^{1}H NMR spectrum (400 MHz, $CDCl_3$, 298K) for [Rh(Flu-Mes)(COE)] (**14**).

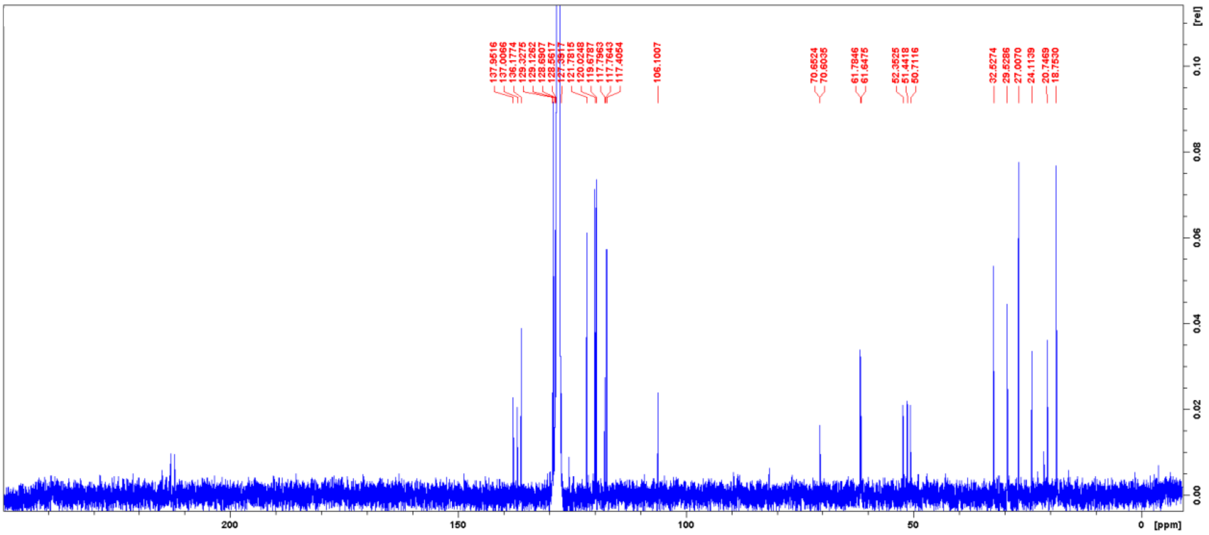

Figure S71. ^{13}C{^{1}H} NMR spectrum (101 MHz, $CDCl_3$, 298K) for [Rh(Flu-Mes)(COE)] (**14**).

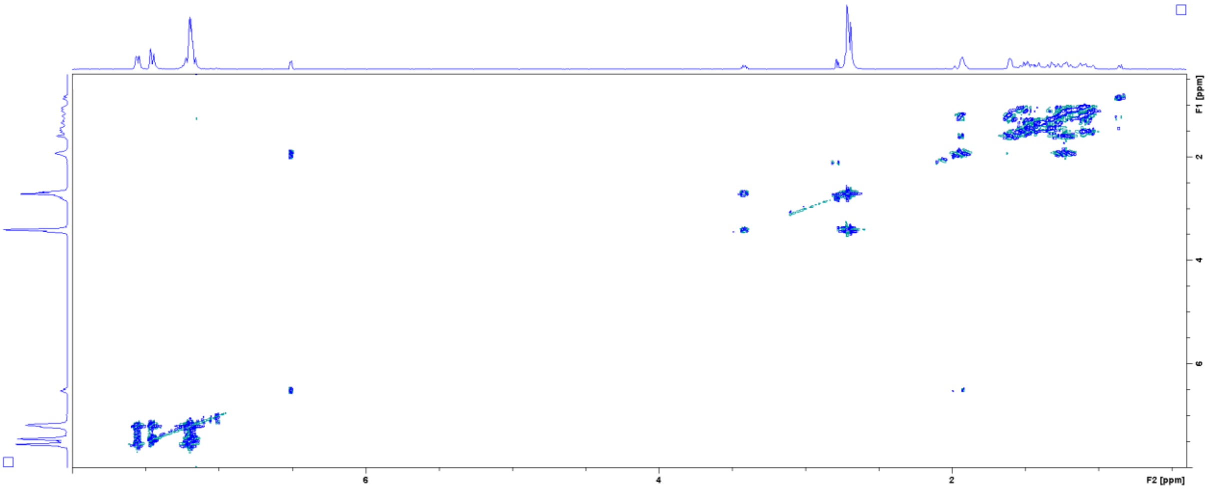

Figure S72. ^{1}H-^{1}H COSY for **14.**

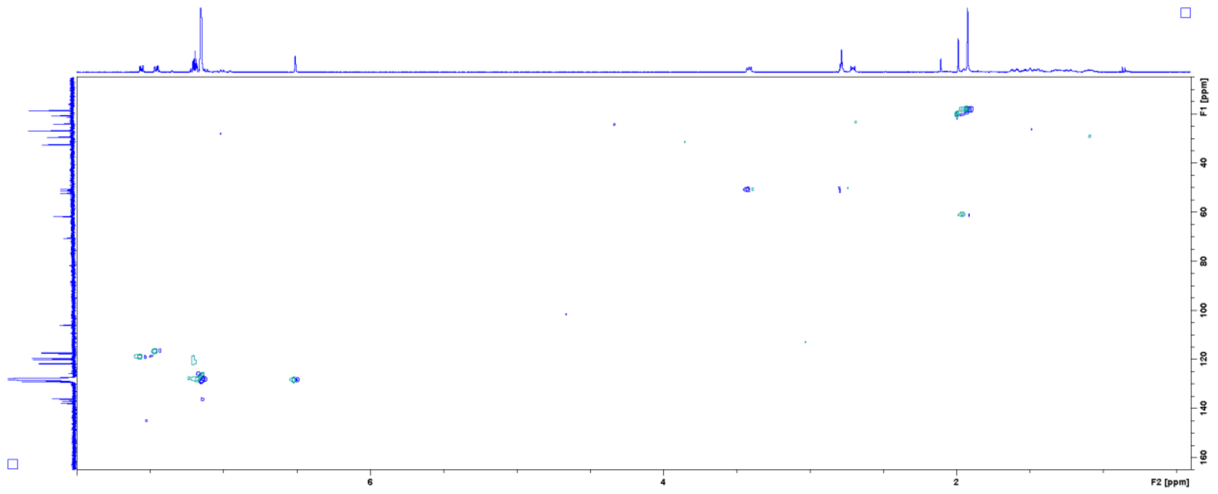

Figure S73. ^{1}H-^{13}C HSQC for **14.**

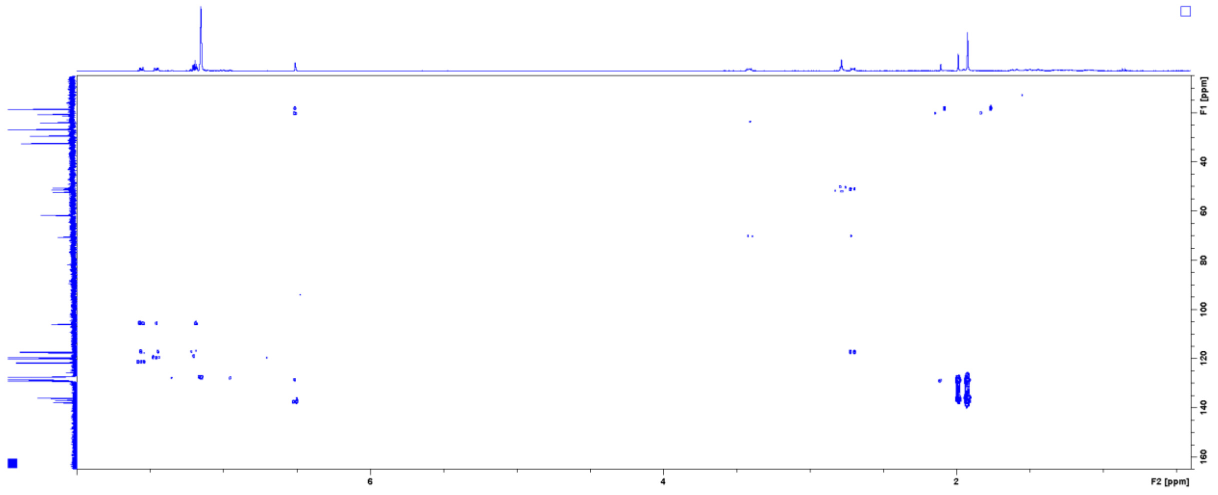

Figure S74. ^{1}H-^{13}C HMBC for **14.**

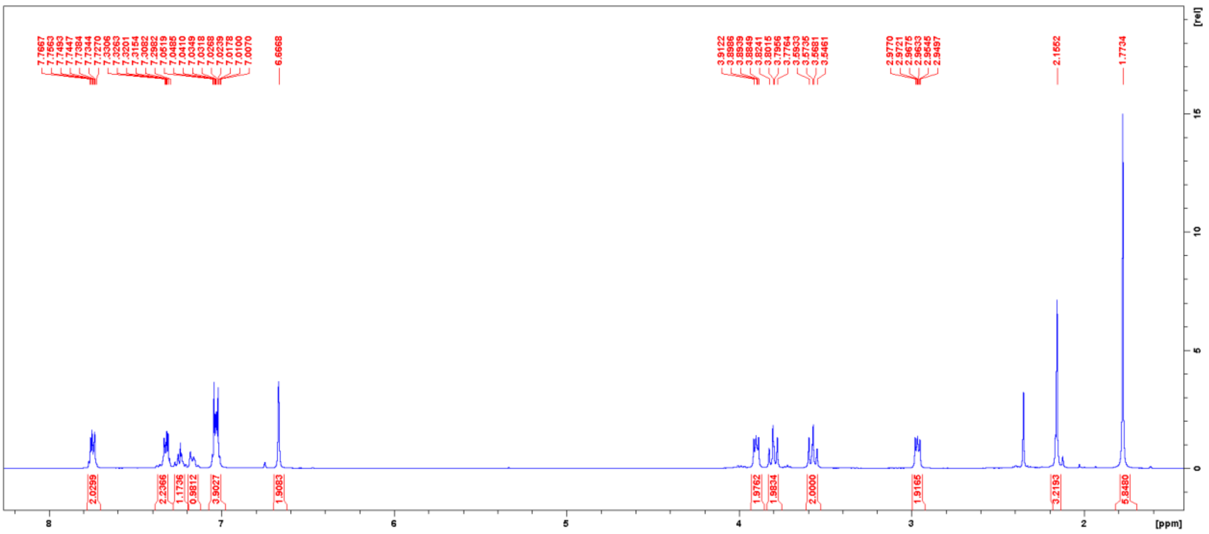

Figure S75. ^{1}H NMR spectrum (400 MHz, $CDCl_3$, 298K) for [Rh(Flu-Mes)(CO)] (**15**).

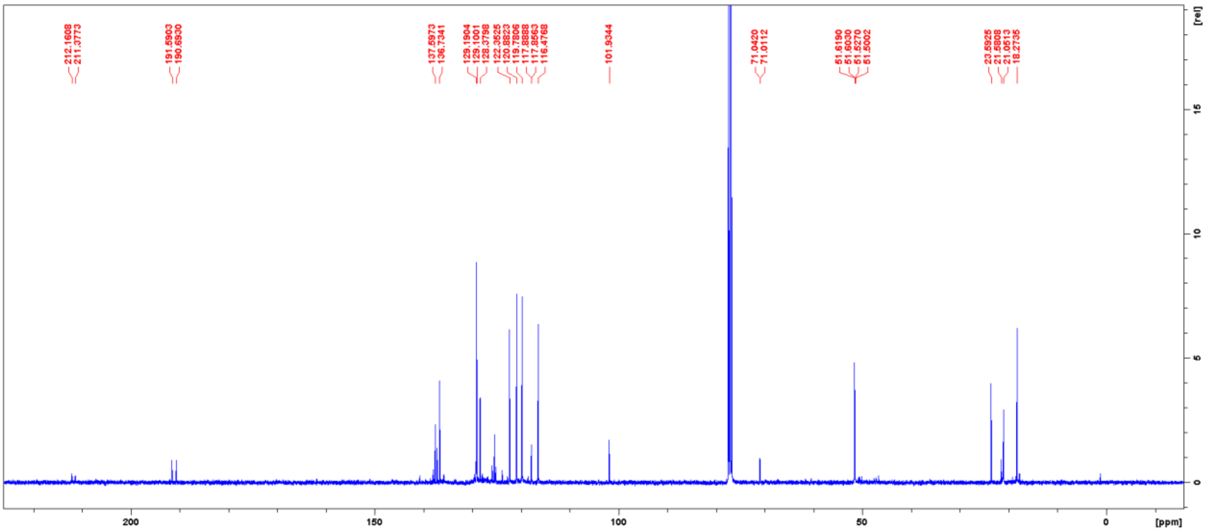

Figure S76. $^{13}C\{^{1}H\}$ NMR spectrum (101 MHz, $CDCl_3$, 298K) for [Rh(Flu-Mes)(CO)] (**15**).

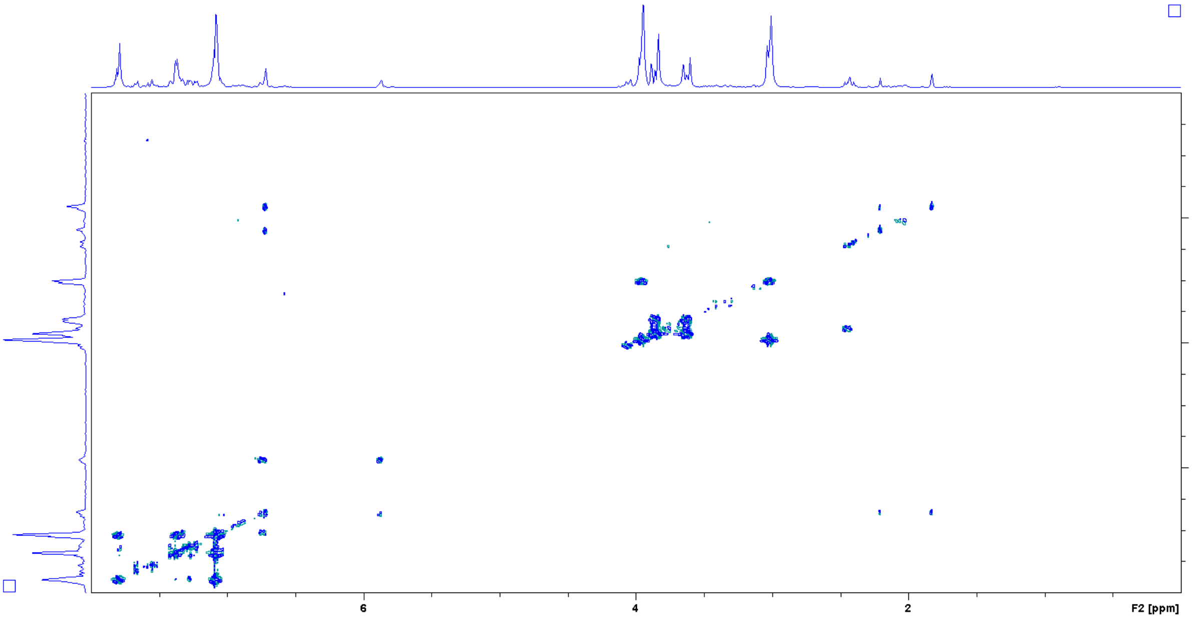

Figure S77. ^{1}H-^{1}H COSY for **15.**

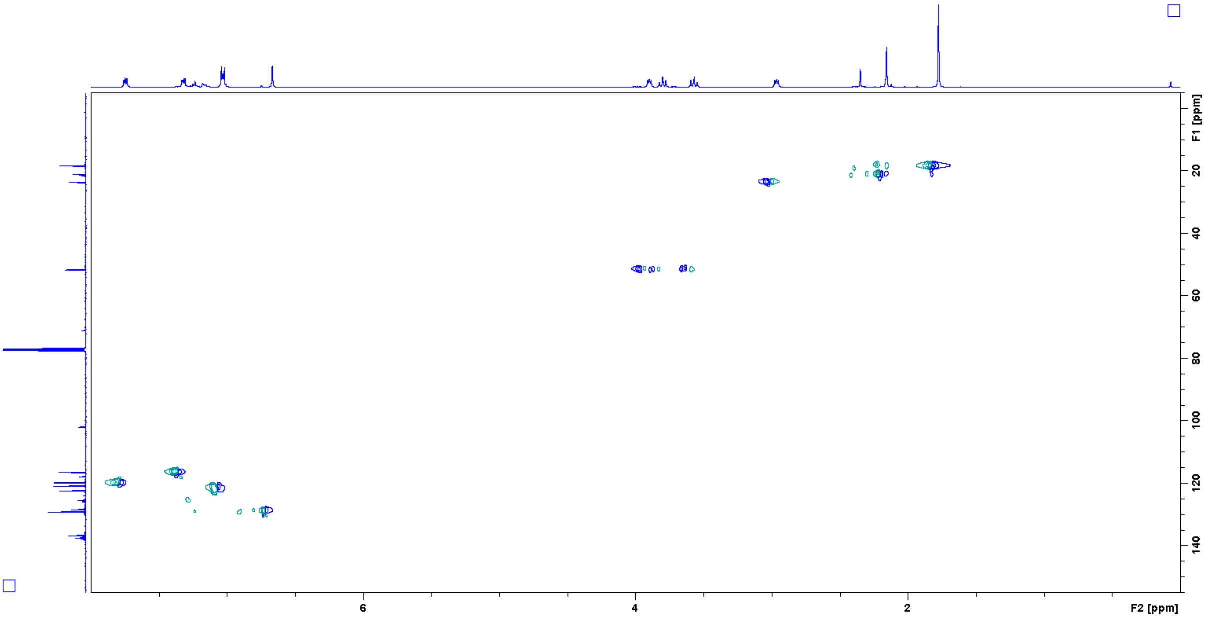

Figure S78. ^{1}H-^{13}C HSQC for **15.**

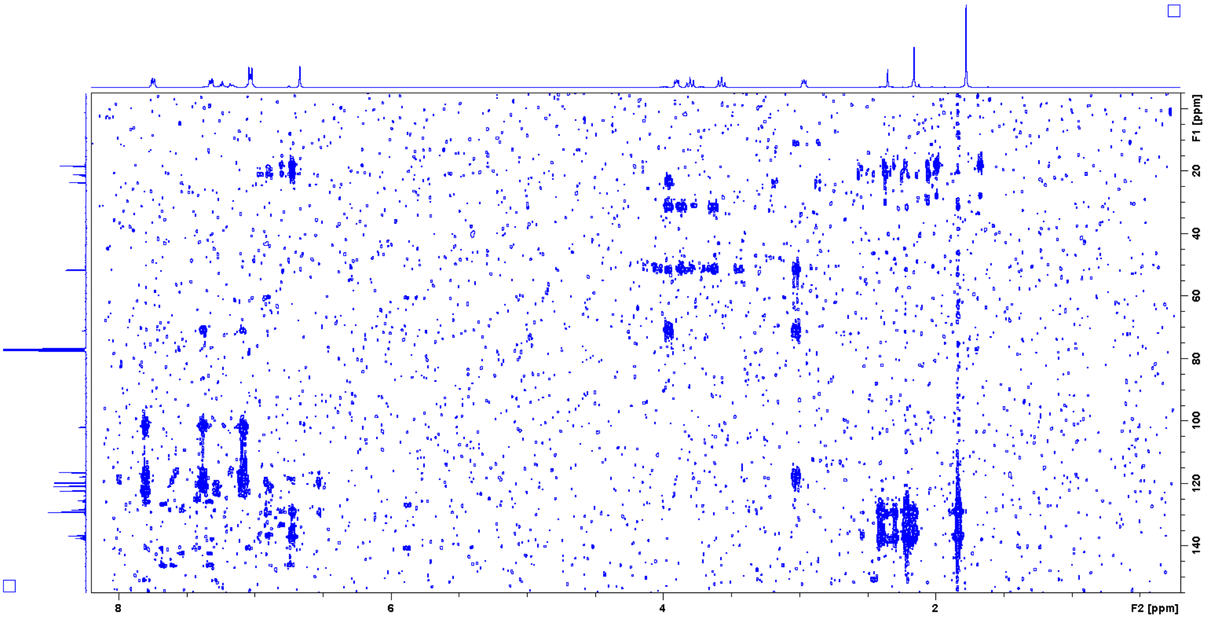

Figure S79. ^{1}H-^{13}C HMBC for **15.**

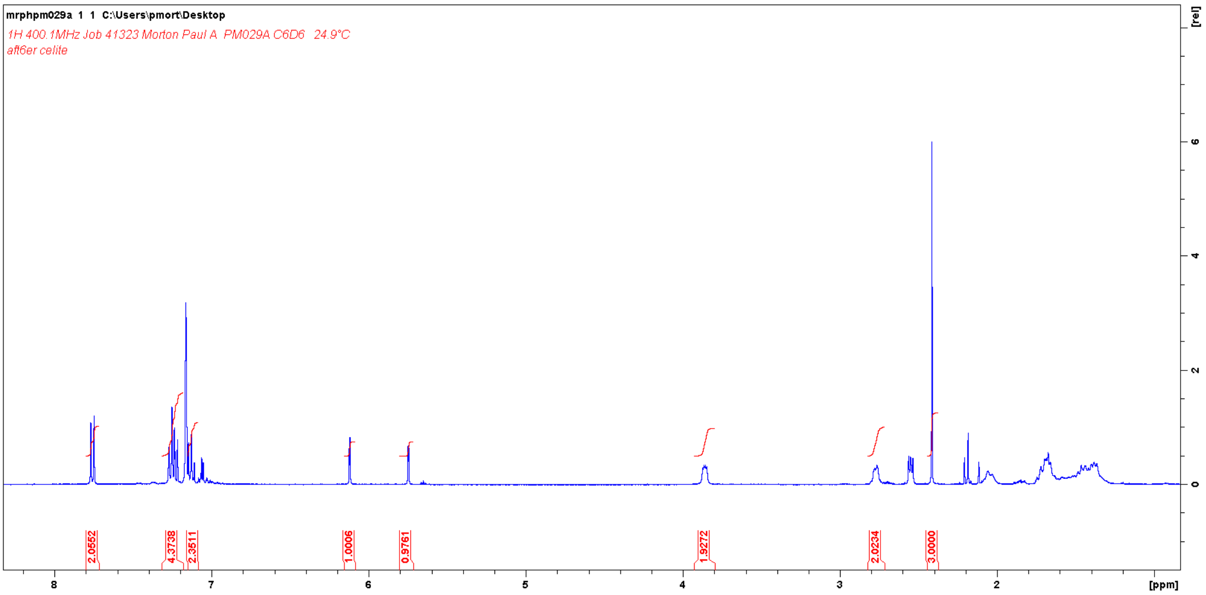

Figure S80. ^{1}H NMR spectrum (400 MHz, C_6D_6, 298K) for [Rh(Flu-Me)(COE)] (**16**).

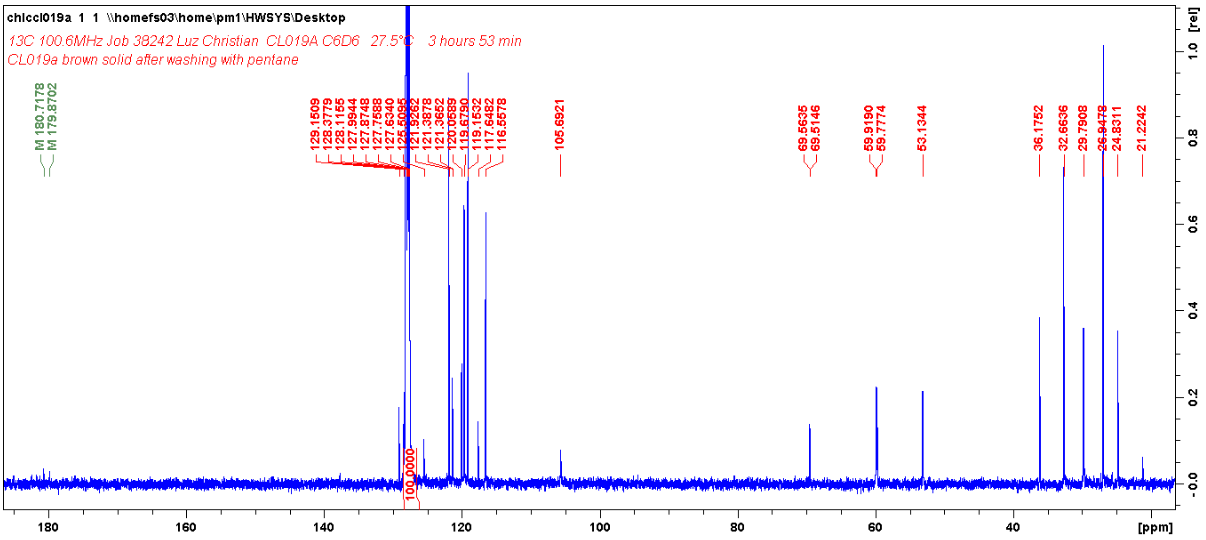

Figure S81. $^{13}C\{^{1}H\}$ NMR spectrum (101 MHz, C_6D_6, 298K) for [Rh(Flu-Me)(COE)] (**16**).

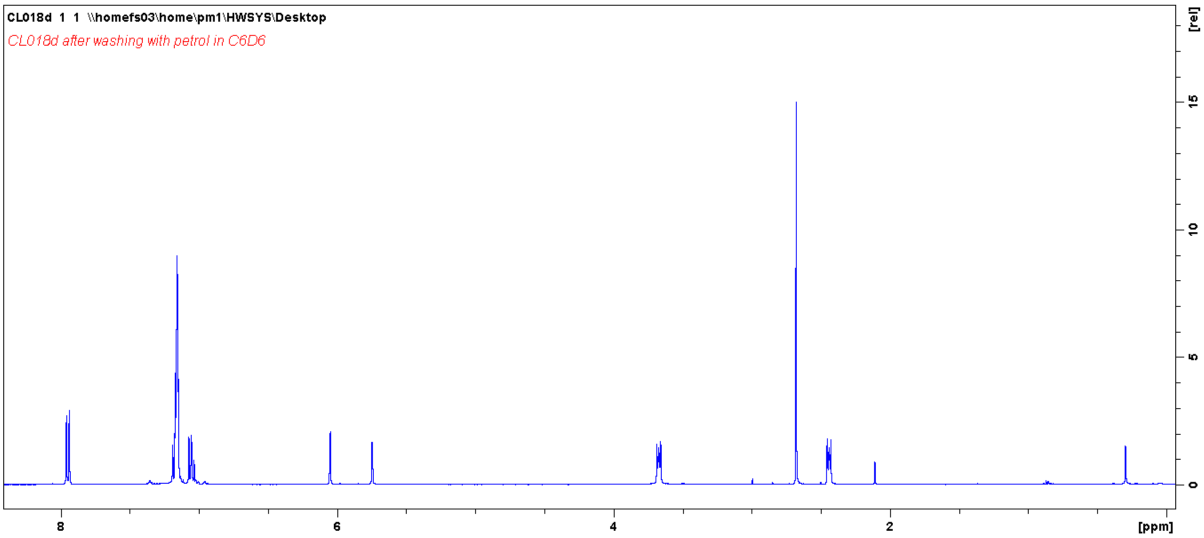

Figure S82. ^{1}H NMR spectrum (400 MHz, C_6D_6, 298K) for [Rh(Flu-Me)(CO)] (**17**).

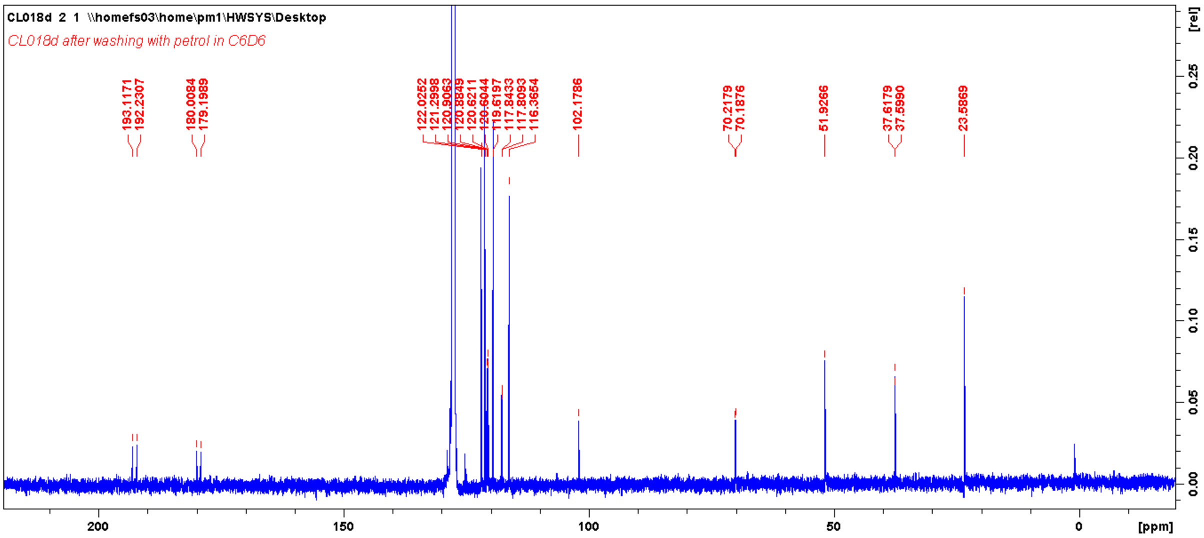

Figure S83. ^{13}C{^{1}H} NMR spectrum (101 MHz, C_6D_6, 298K) for [Rh(Flu-Me)(CO)] (**17**).

5.3 Arene borylation

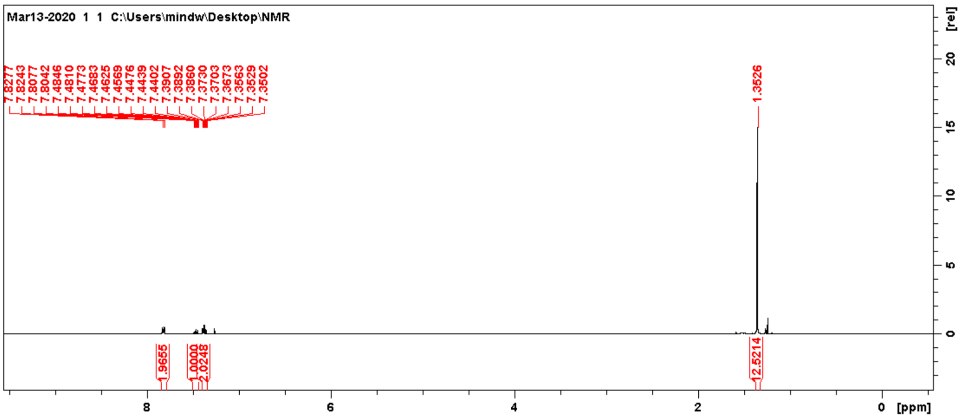

Figure S84. ^{1}H NMR spectrum (400 MHz, $CDCl_3$, 298 K) for isolated PhBpin.

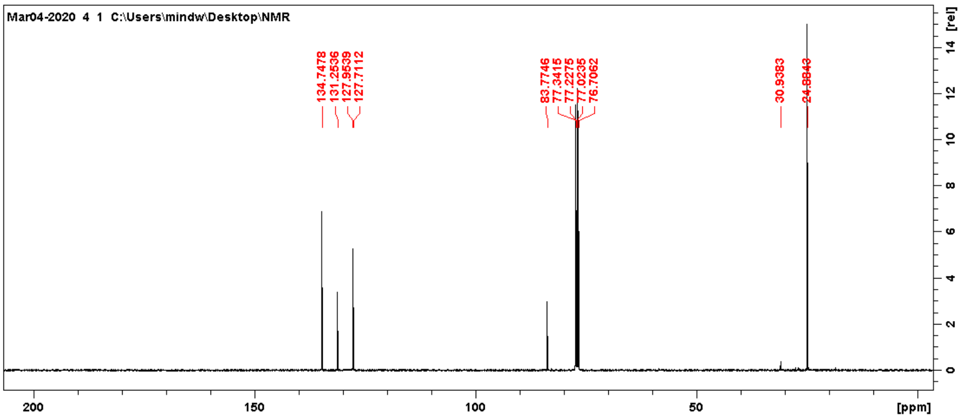

Figure S85. ^{13}C NMR spectrum (101 MHz, CDCl3, 298K) for isolated PhBpin.

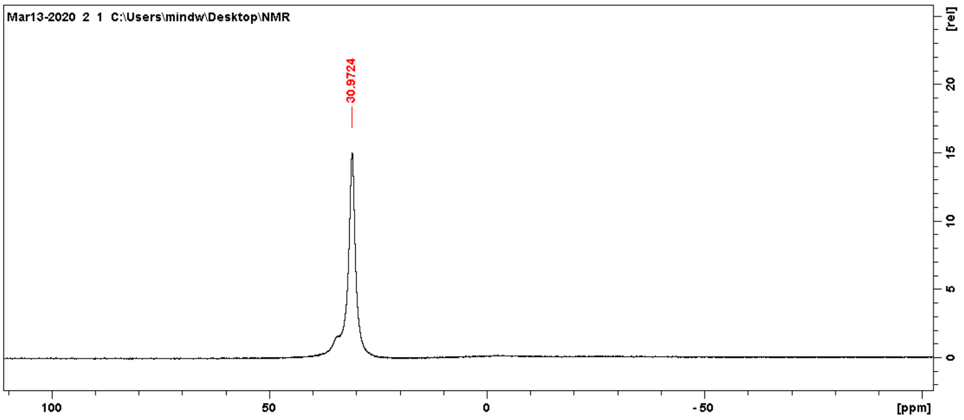

Figure S86. ^{11}B NMR spectrum (128 MHz, $CDCl_3$, 298 K) for isolated PhBpin.

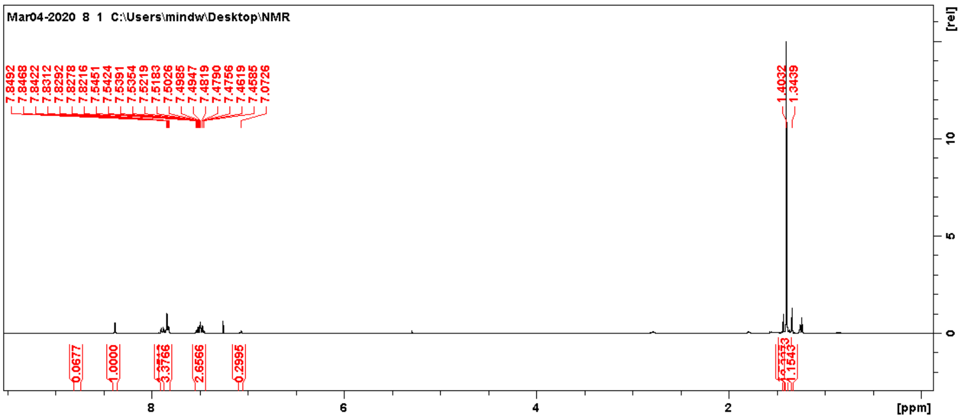

Figure S87. ^{1}H NMR spectrum (400 MHz, $CDCl_3$, 298K) for isolated naphthylBpin. Major product is the 2-position isomer.

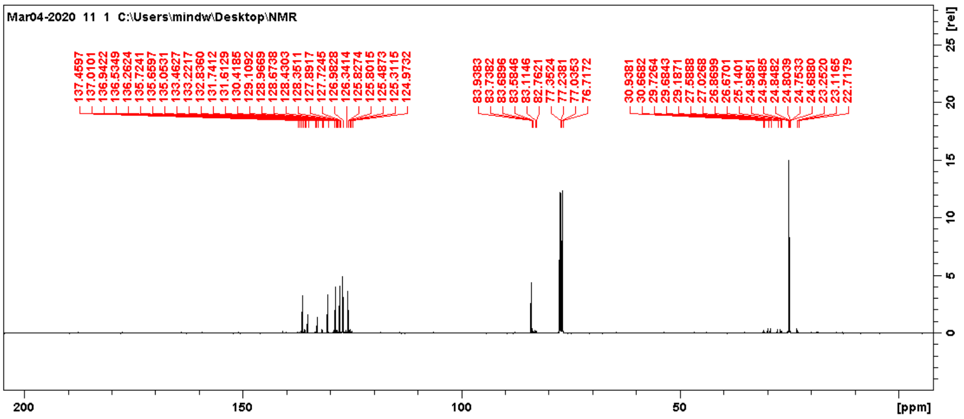

Figure S88. ^{13}C NMR spectrum (101 MHz, $CDCl_3$, 298K) for isolated naphthylBpin.

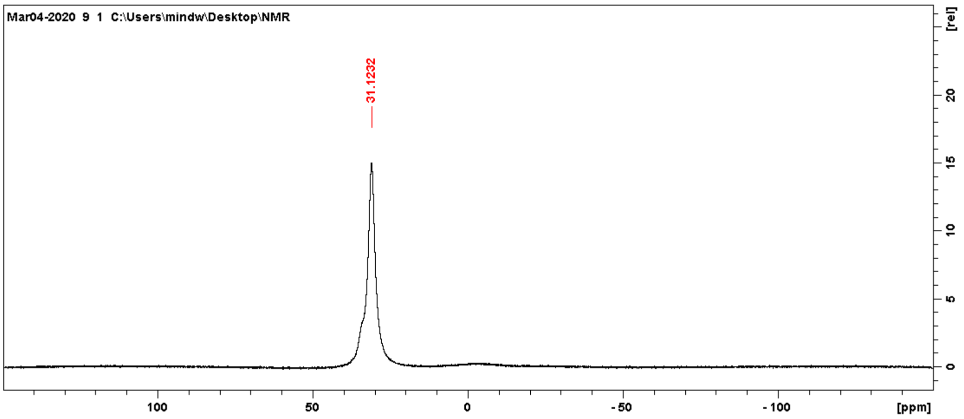

Figure S89. ^{11}B NMR spectrum (128 MHz, $CDCl_3$, 298K) for isolated naphthylBpin.

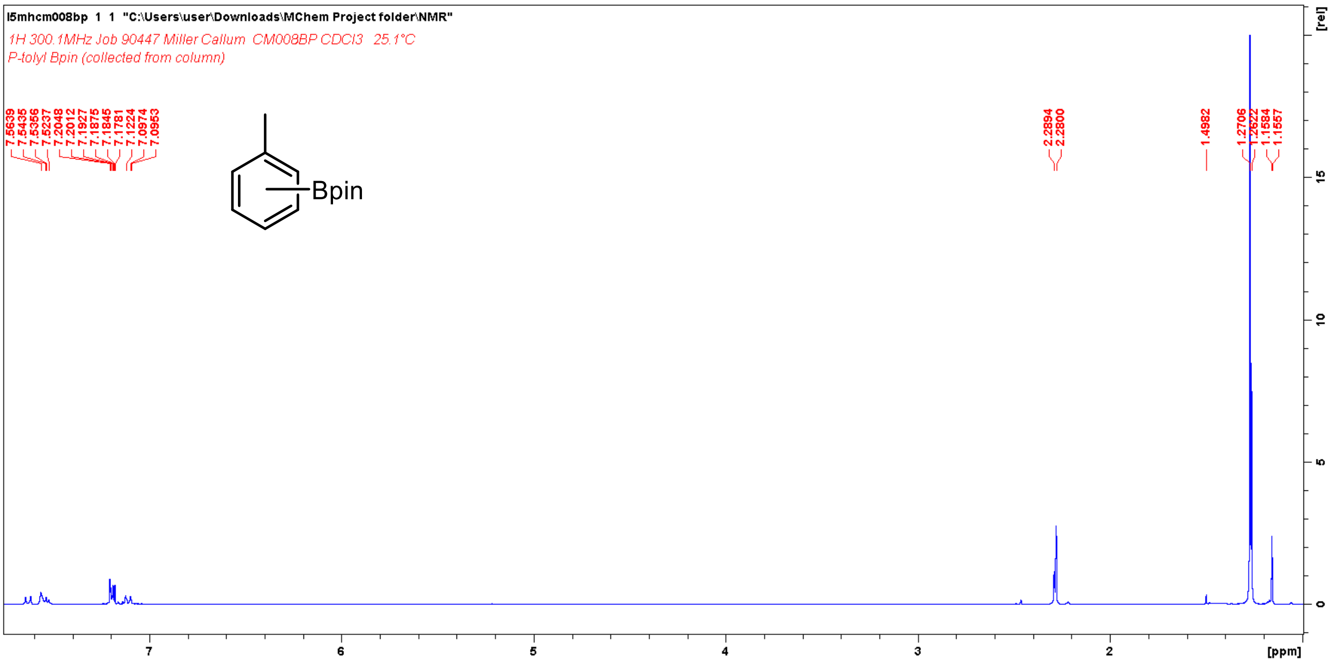

Figure S90.^{1}H NMR spectrum (300 MHz, $CDCl_3$, 298 K) of tolylBpin.

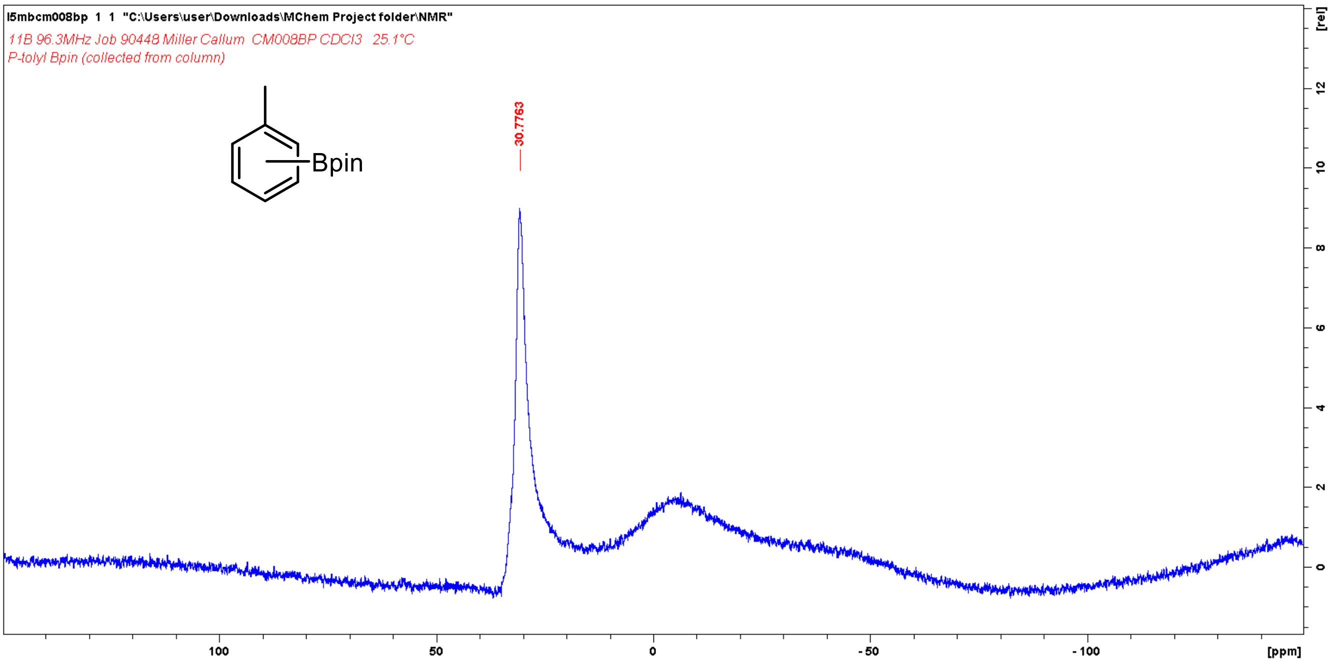

Figure S91. ^{11}B NMR spectrum (96.3 MHz, $CDCl_3$, 298 K)

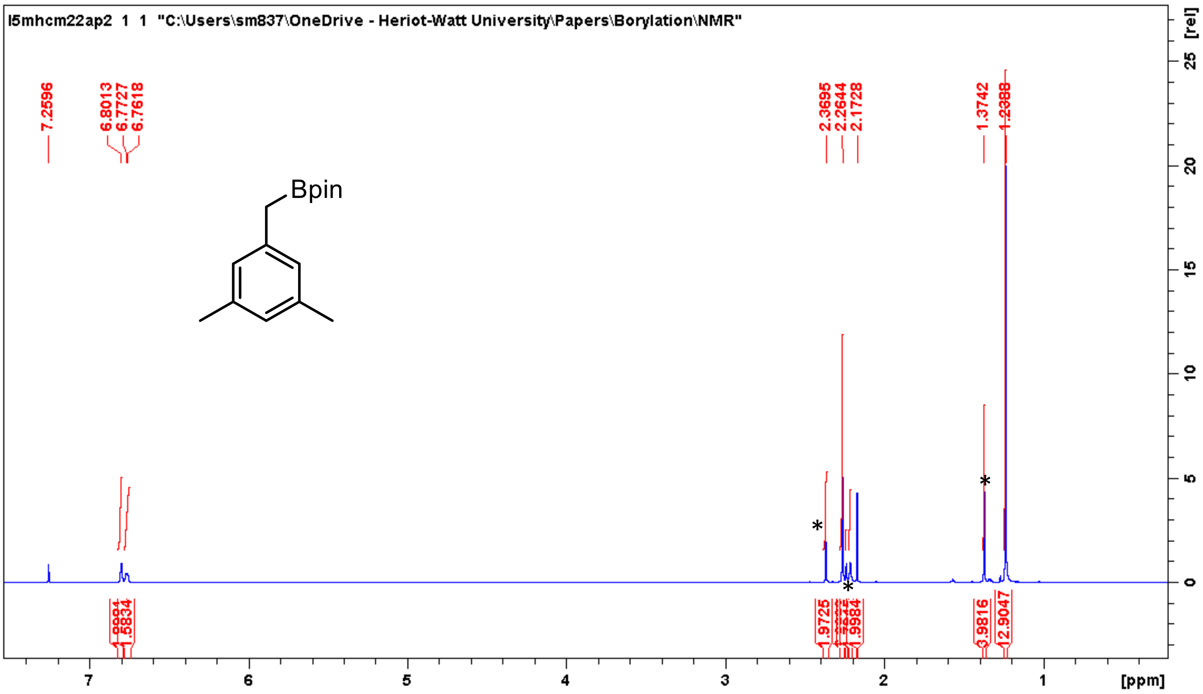

Figure S92. ^{1}H NMR spectrum (300 MHz, $CDCl_3$, 298 K). Impurities denoted with *

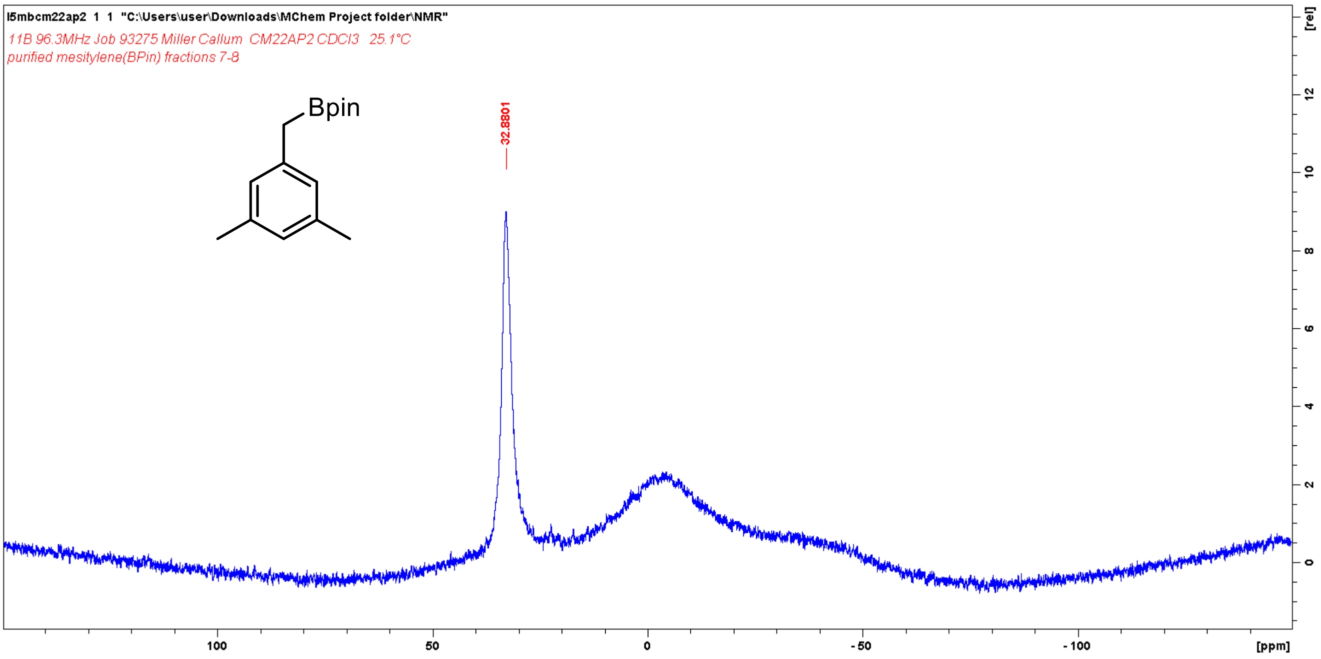

Figure S93. ^{11}B NMR spectrum (96.3 MHz, $CDCl_3$, 298 K)

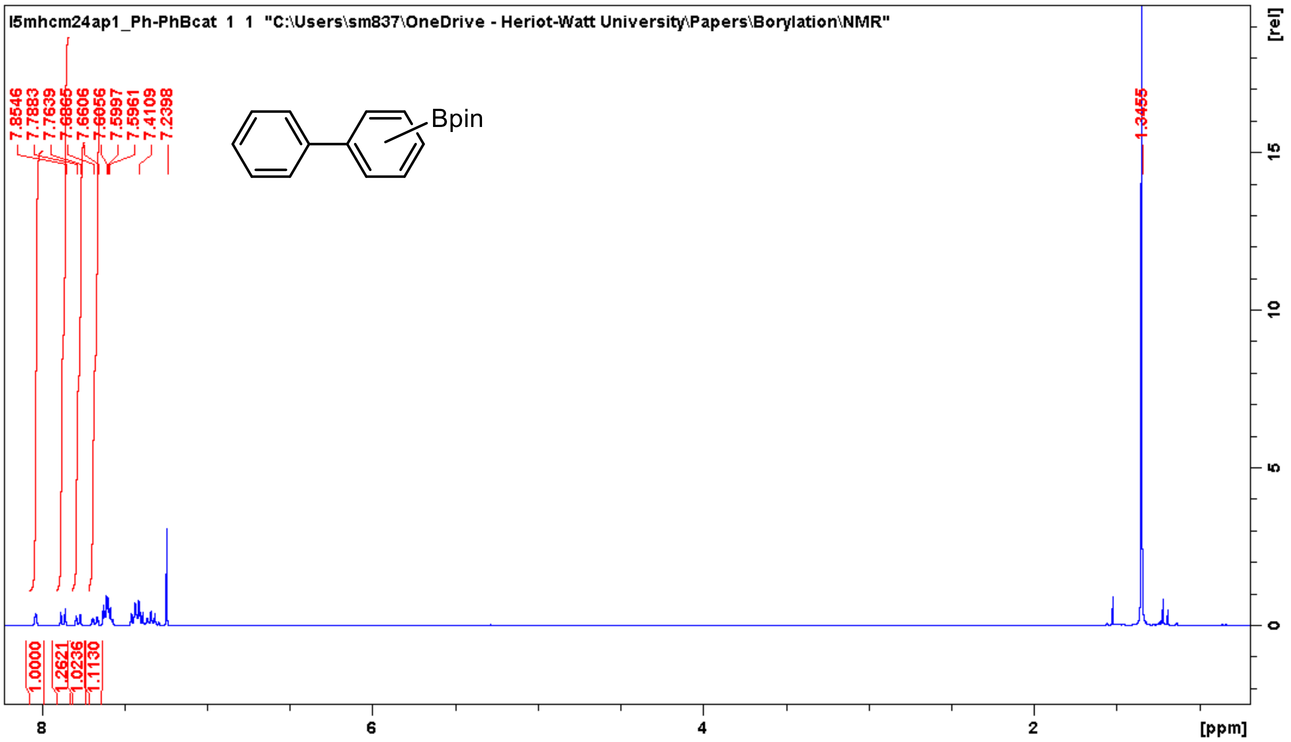

Figure S94. ^{1}H NMR spectrum (300 MHz, $CDCl_3$, 298 K)

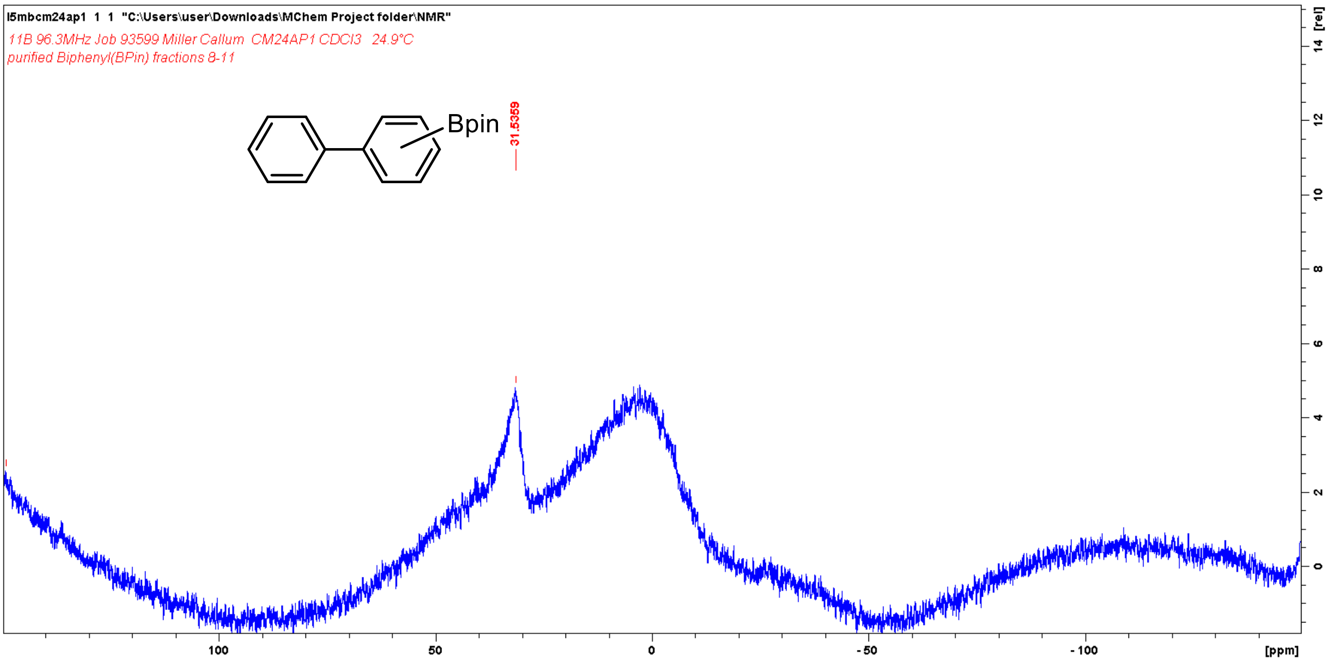

Figure S95. ^{11}B NMR spectrum (96.3 MHz, $CDCl_3$, 298 K)

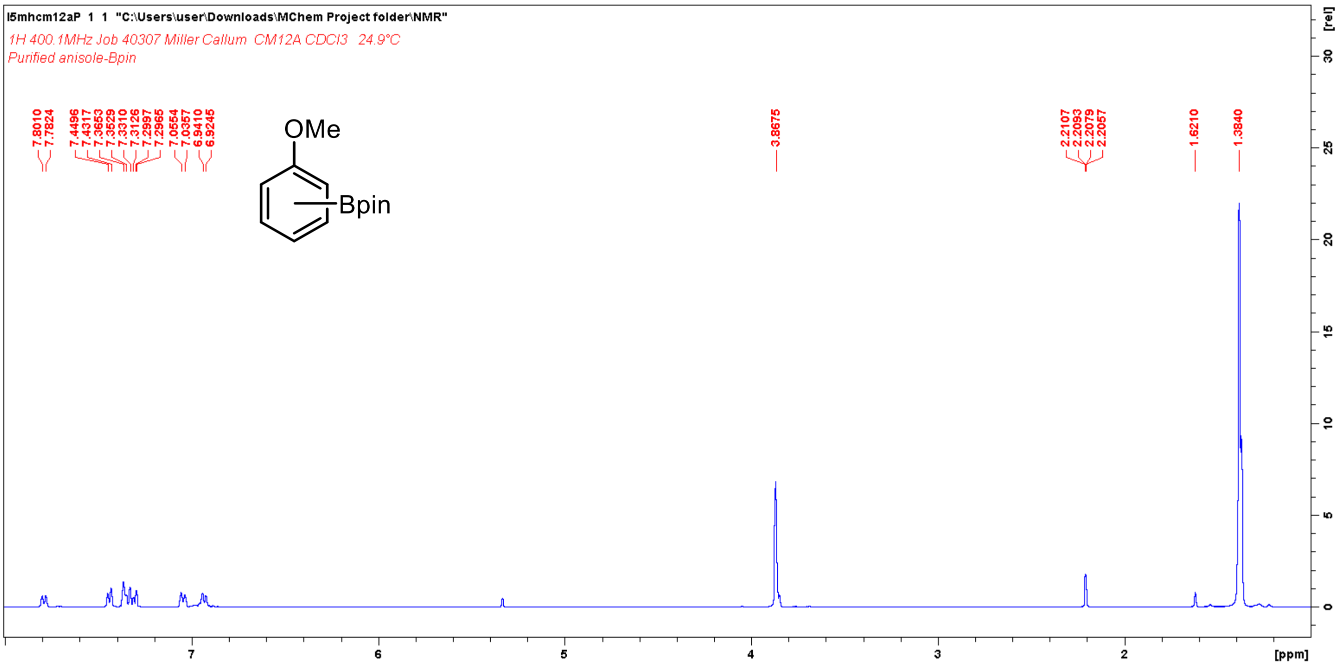

Figure S96. ^{1}H NMR spectrum (400 MHz, $CDCl_3$, 298 K)

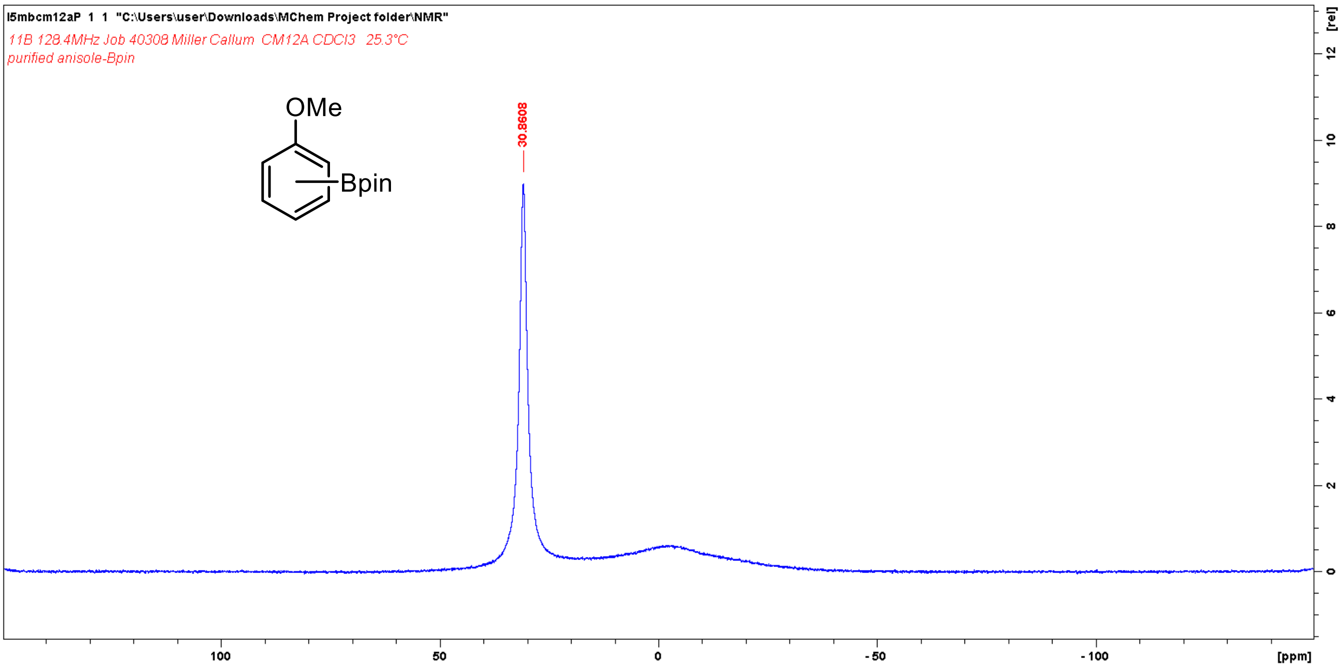

Figure S97. ^{11}B NMR spectrum (128.4 MHz, $CDCl_3$, 298 K)

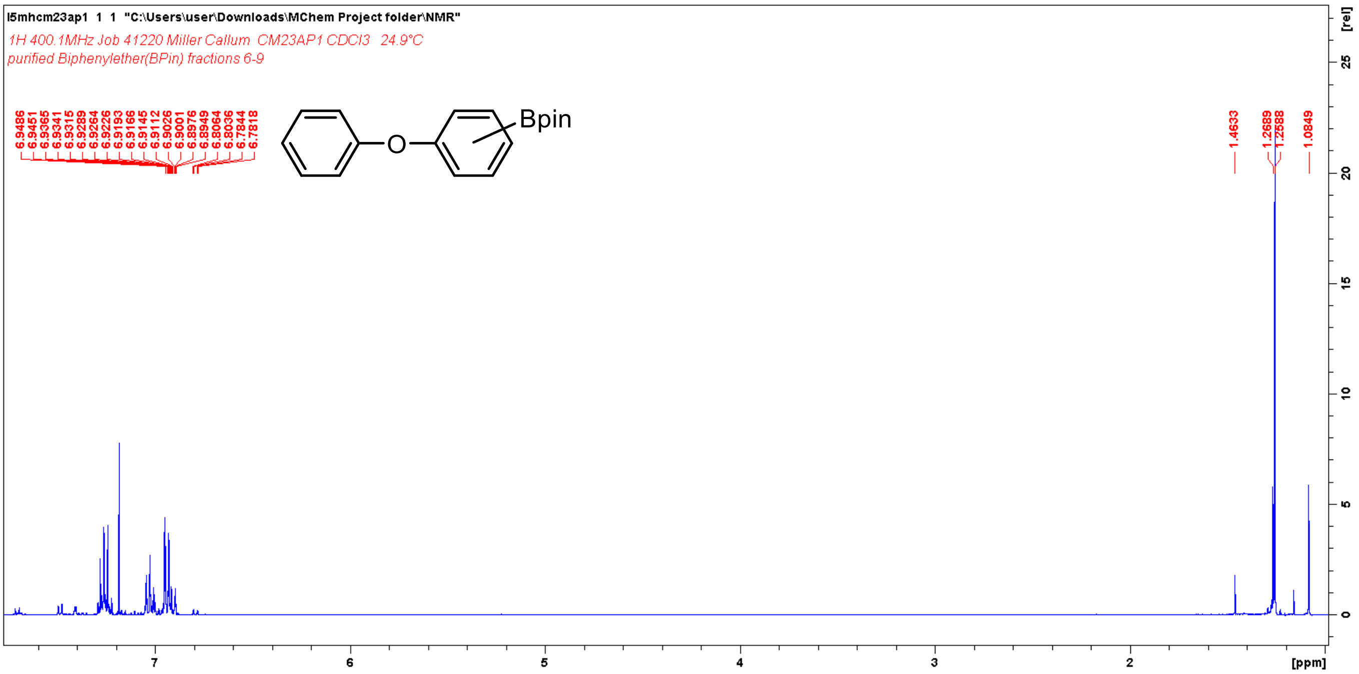

Figure S98. ^{1}H NMR (400 MHz, $CDCl_3$, 298 K)

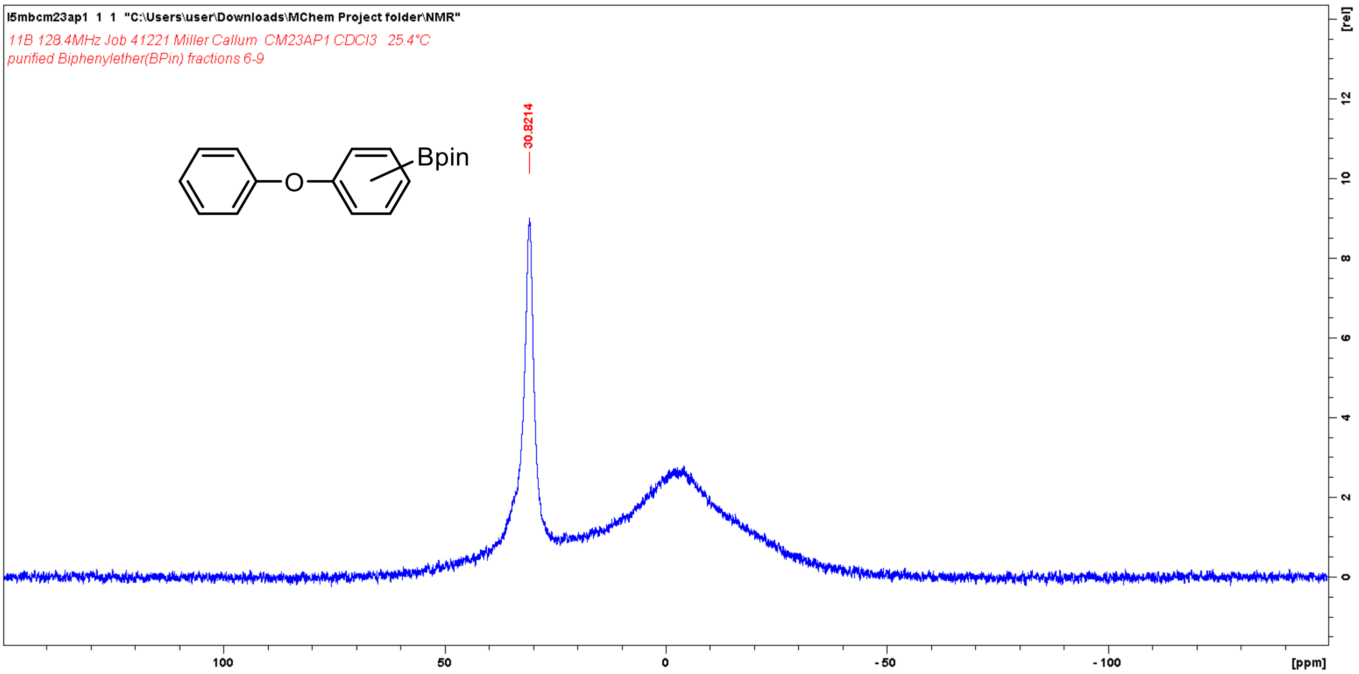

Figure S99. ^{11}B NMR (128.4 MHz, $CDCl_3$, 298 K)

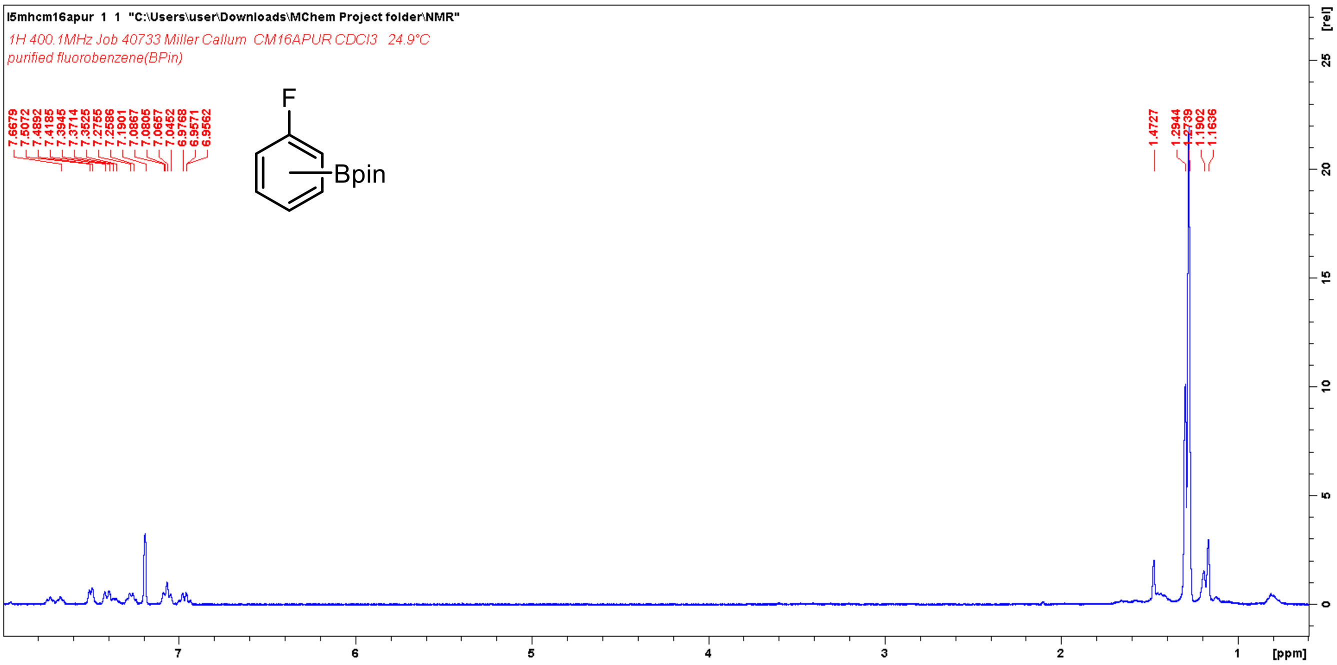

Figure S100. ^{1}H NMR spectrum (400 MHz, $CDCl_3$, 298 K)

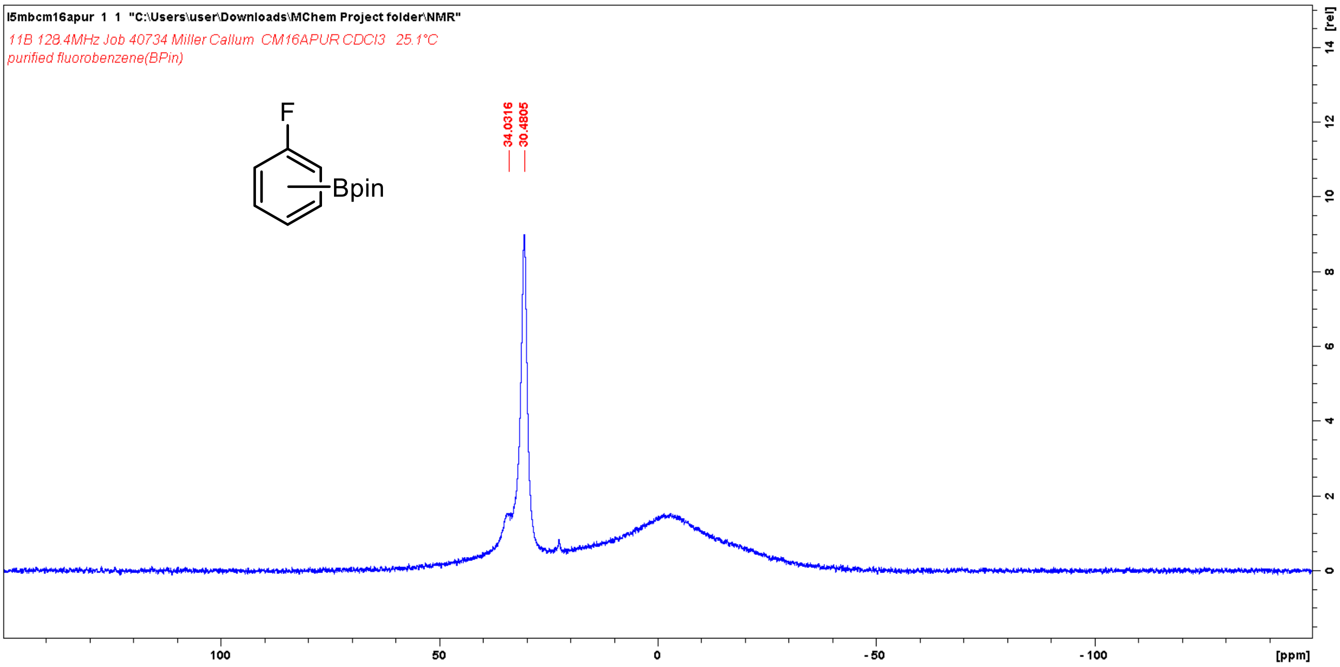

Figure S101. ^{11}B NMR spectrum (128.4 MHz, $CDCl_3$, 298 K)

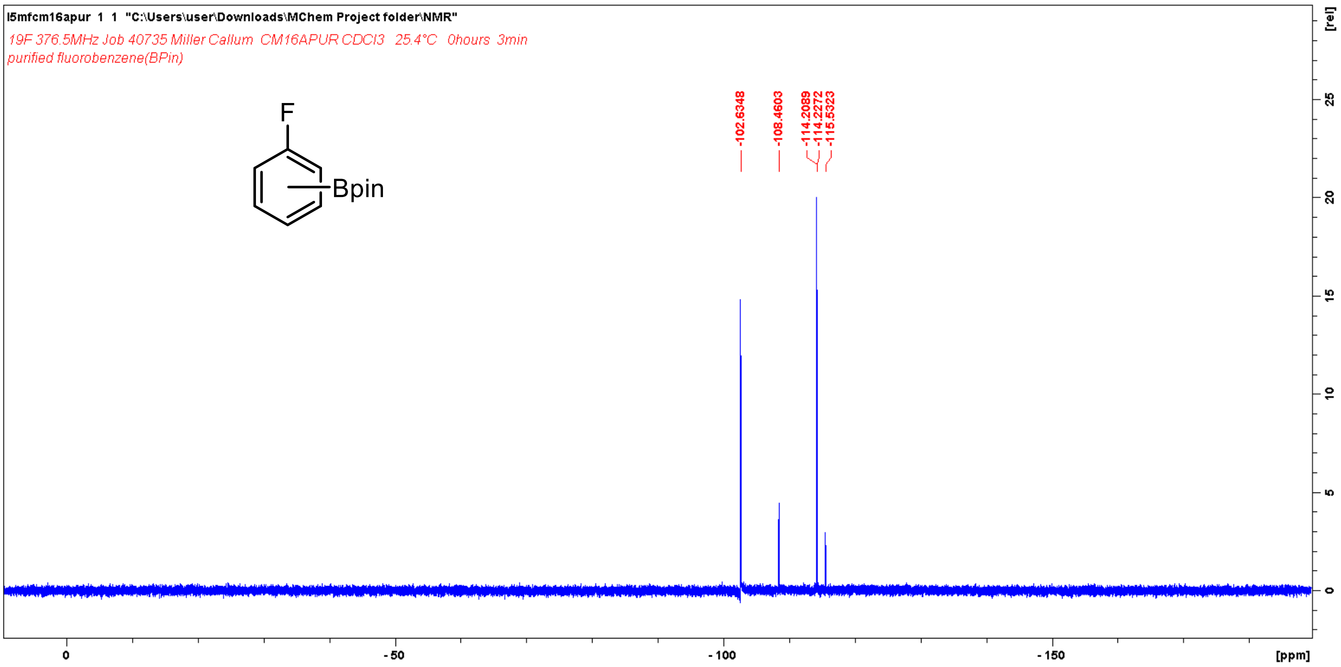

Figure S102. ^{19}F NMR spectrum (376.5 MHz, $CDCl_3$, 298 K)

5.4 Alkane borylation

$^{11}B\{^1H\}$ NMR spectra referenced to pinBOBpin impurity (21.65 ppm).

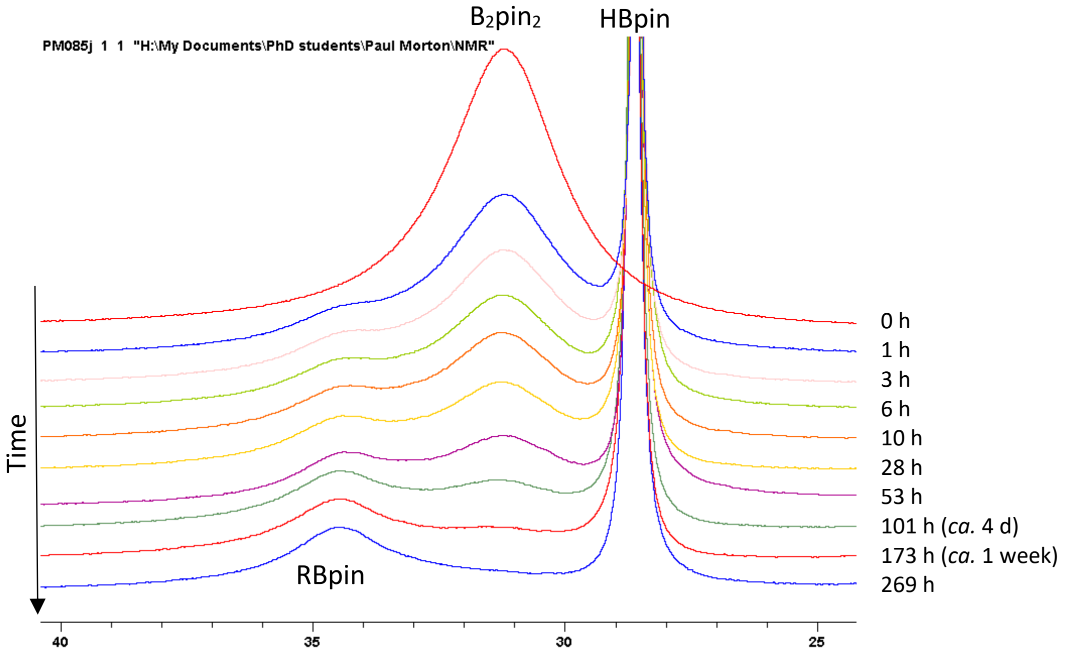

Figure S103. ^1H NMR stack plot of the borylation of decane with B_2pin_2 catalysed by 5 mol% [Rh(Ind)(SIPr)(COE)] at 140°C.

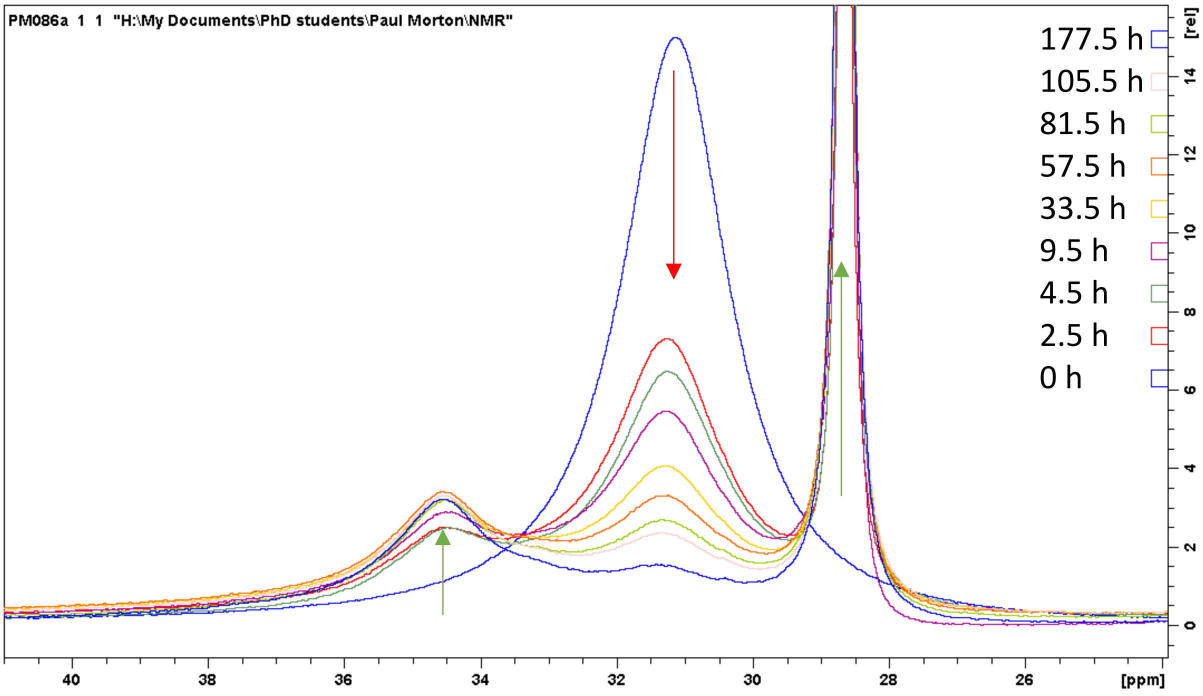

Figure S104. ^1H NMR stack plot of the borylation of octane with B_2pin_2 catalysed by 5 mol% [Rh(Ind)(SIPr)(COE)] at 120°C.

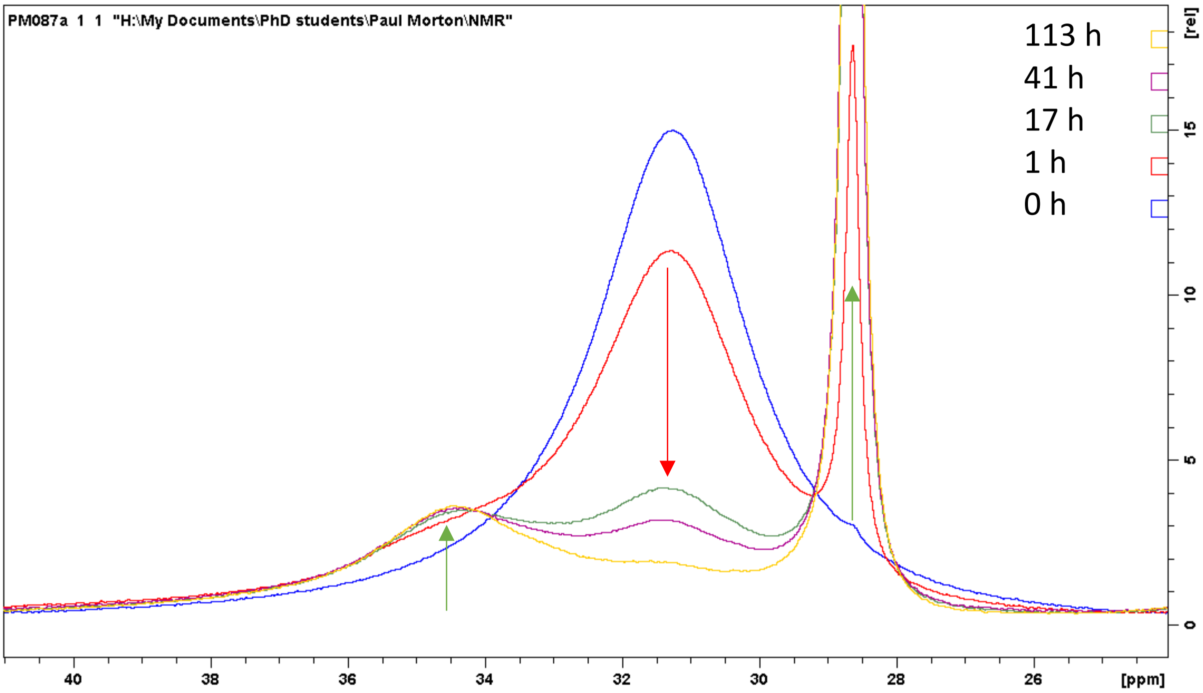

Figure S105. ^{1}H NMR stack plot of the borylation of decane with B_2pin_2 catalysed by 5 mol% [Rh(Ind)(IMes)(COE)] at 140°C.

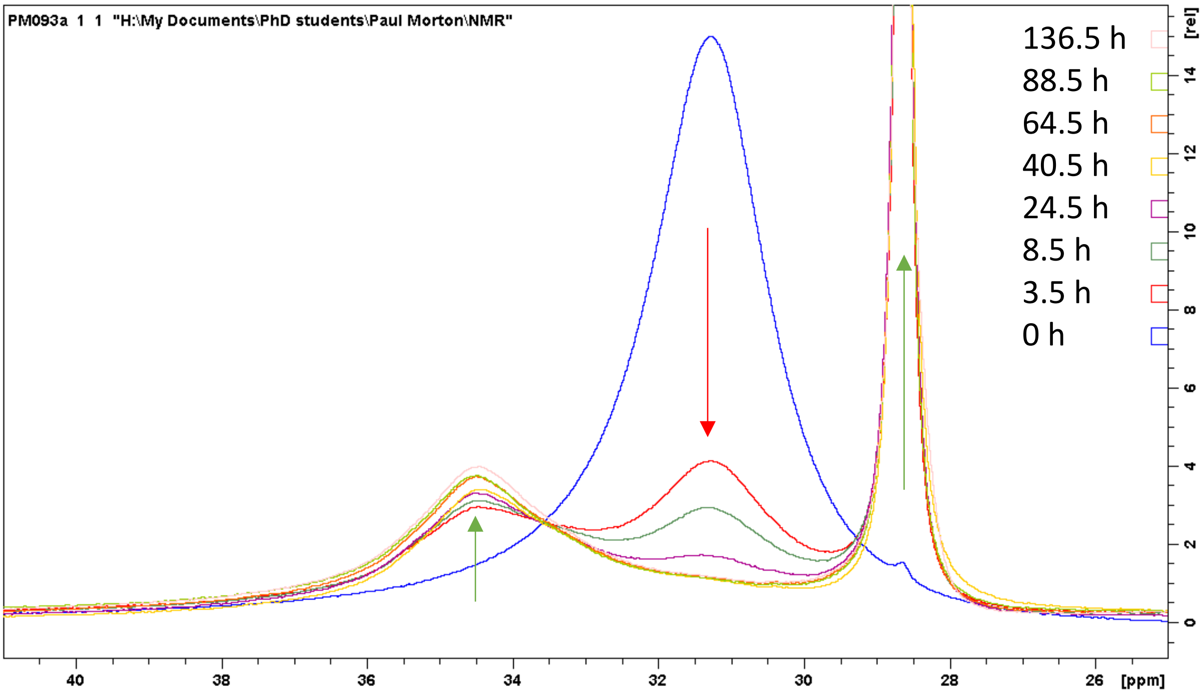

Figure S106. ^{1}H NMR stack plot of the borylation of octane with B_2pin_2 catalysed by 5 mol% [Rh(Ind)(IMes)(COE)] at 120°C.

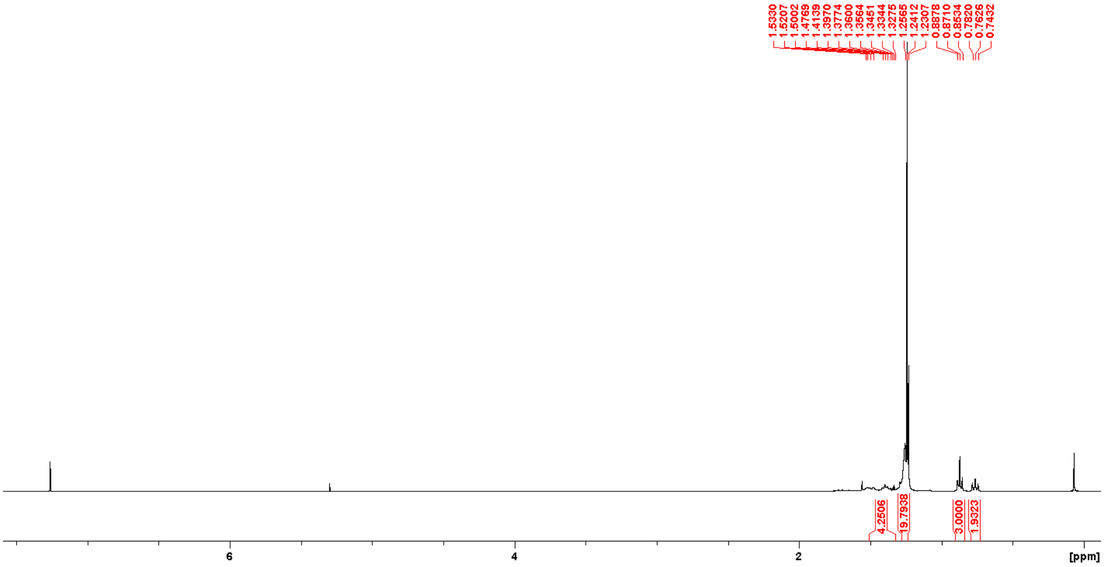

Figure S107. ^{1}H NMR spectrum of octylBpin obtained after purification.

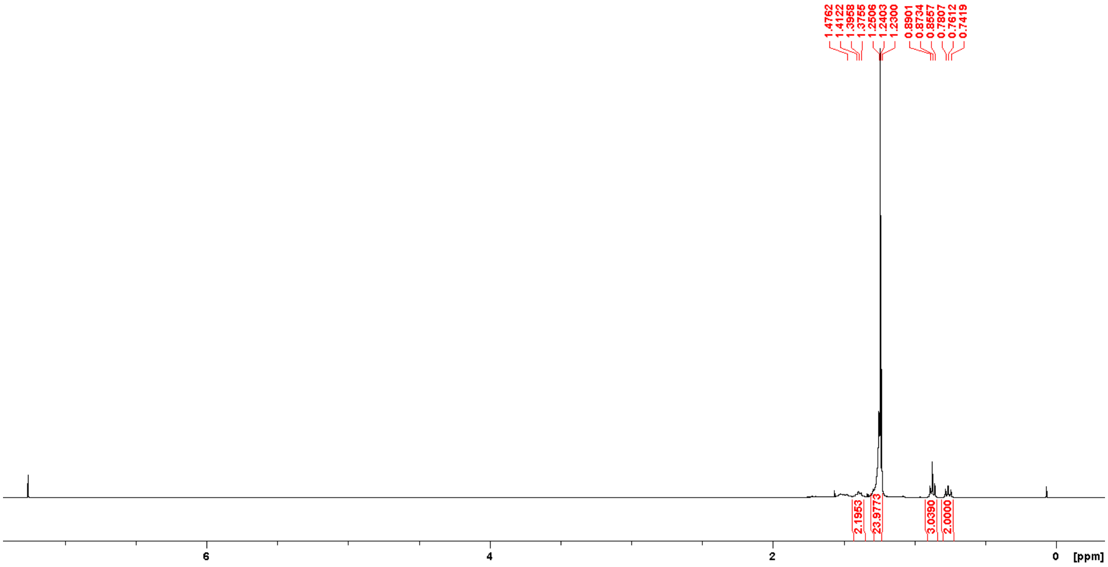

Figure S108. ^{1}H NMR spectrum of decylBpin obtained after purification.

6 UV-vis spectra

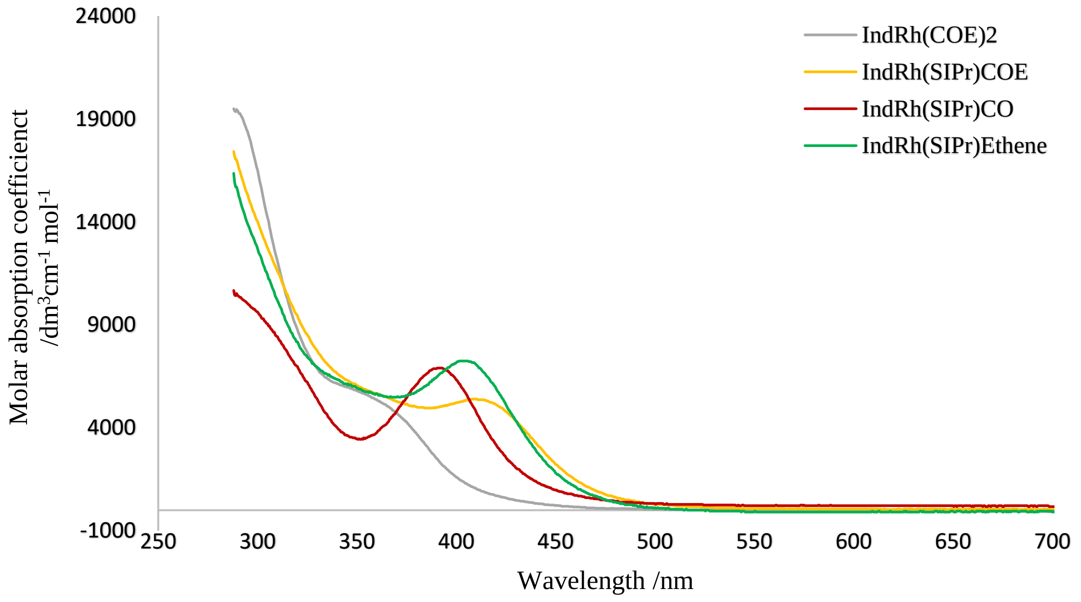

Figure S109. UV-vis spectra for toluene solutions of [Rh(Ind)(COE)$_2$], [Rh(Ind)(SIPr)(C_2H_4)] (**1**), [Rh(Ind)(SIPr)(CO)] (**2a**) and [Rh(Ind)(SIPr)(COE)] (**3**).

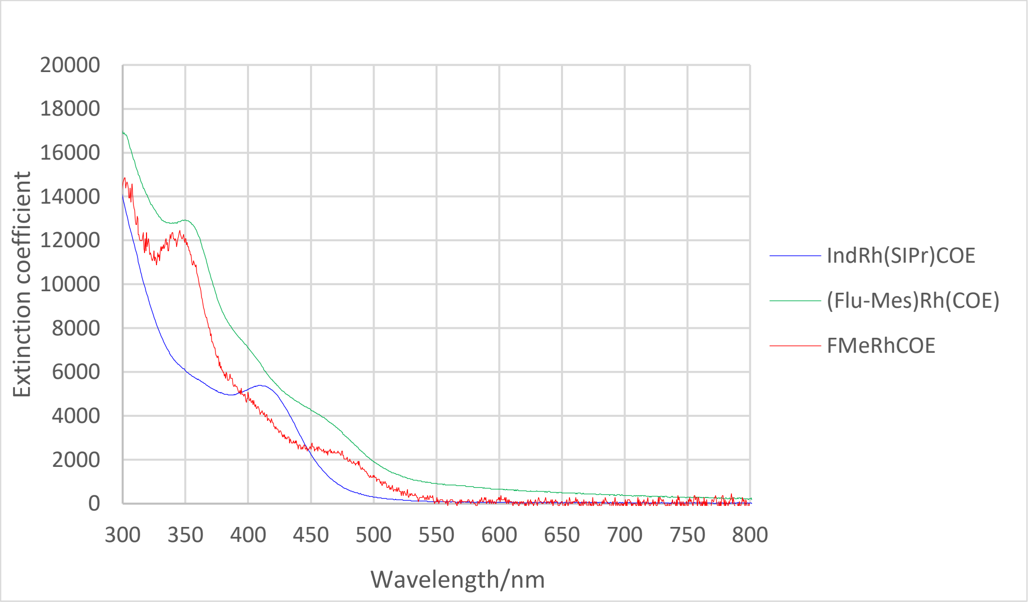

Figure S110. UV-vis spectra for toluene solutions of [Rh(Ind)(SIPr)(COE)] (**3**), [Rh(Flu-Mes)(COE)] (**14**) and [Rh(Flu-Mes)(COE)] (**16**).

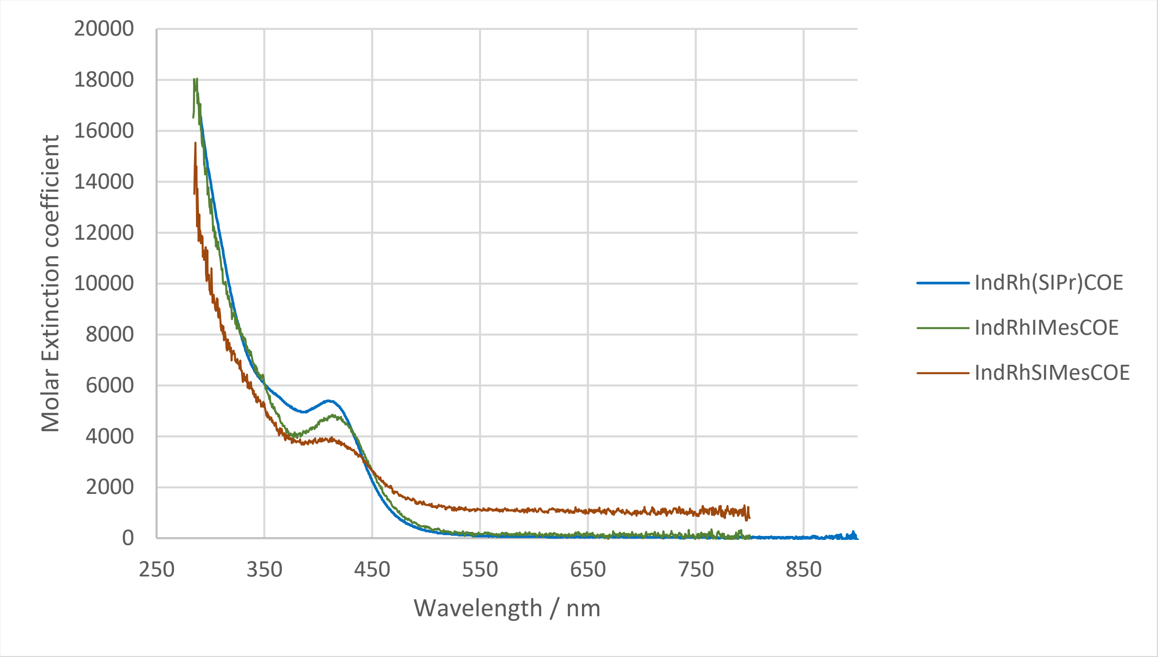

Figure S111. UV-vis spectra for toluene solutions of [Rh(Ind)(SIPr)(COE)] (**3**), [Rh(Ind)(SIMes)(COE)] (**4**) and [Rh(Ind)(IMes)(COE)] (**5**).

7 IR spectra

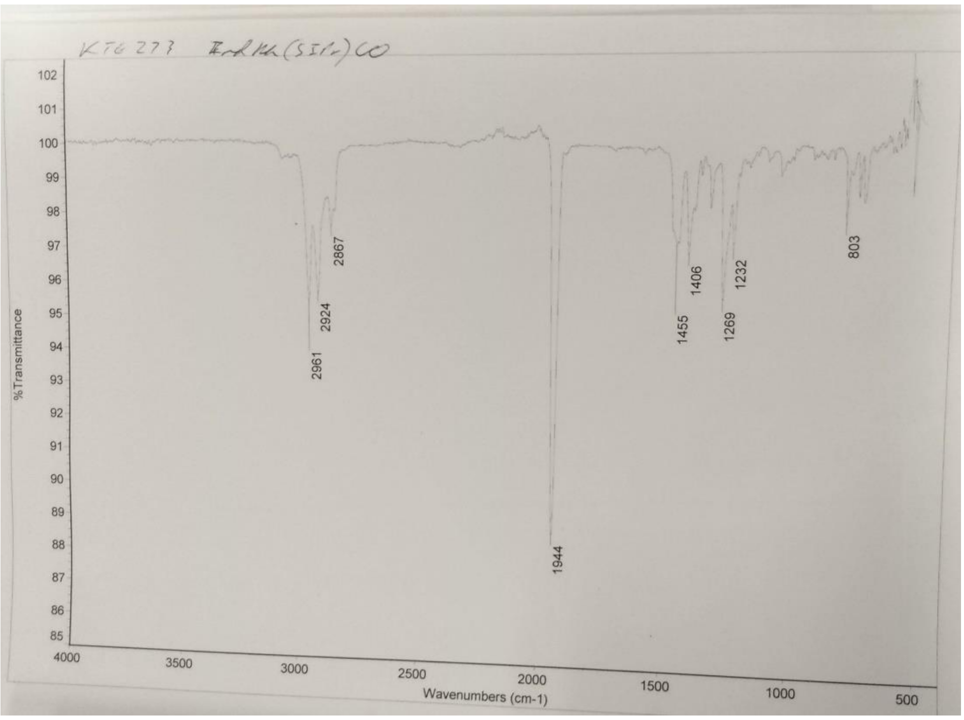

Figure S112: IR spectrum for [Rh(Ind)(SIPr)(CO)] **2a**.

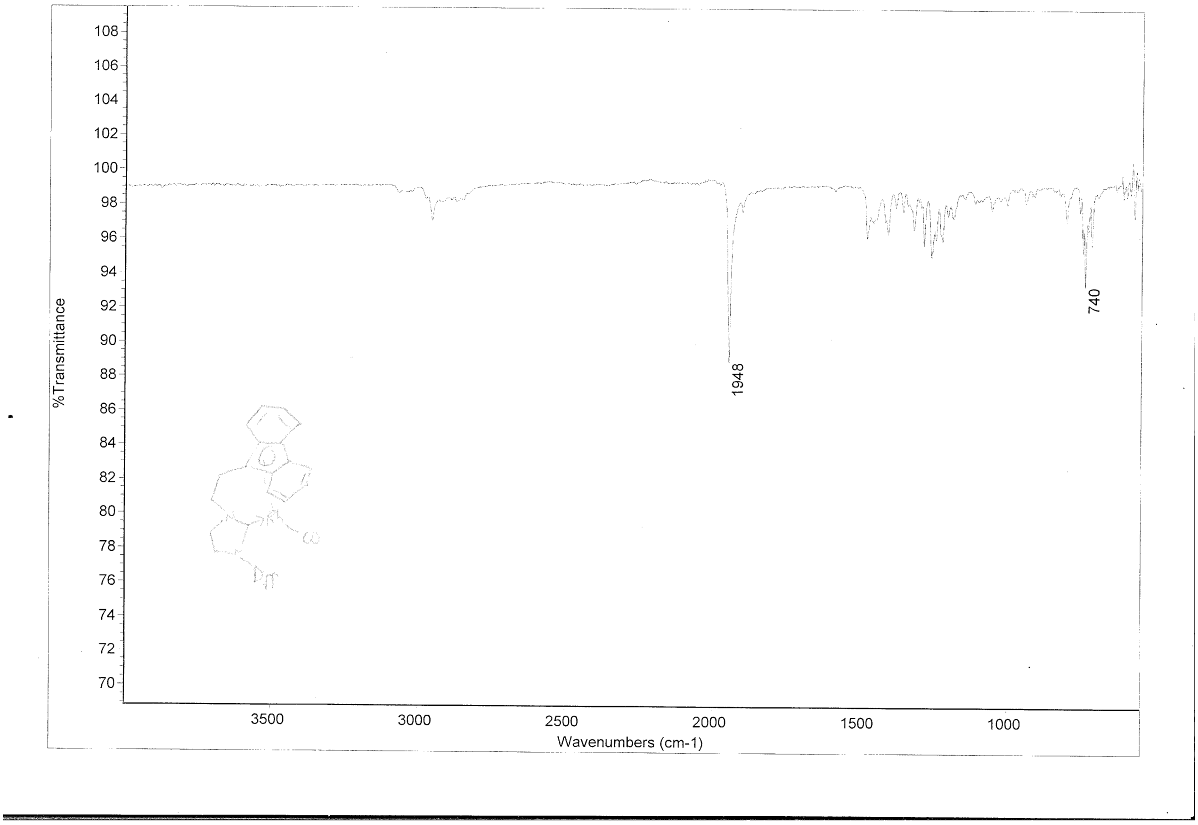

Figure S113: IR spectrum for [Rh(Flu-Dipp)(CO)] **13**.

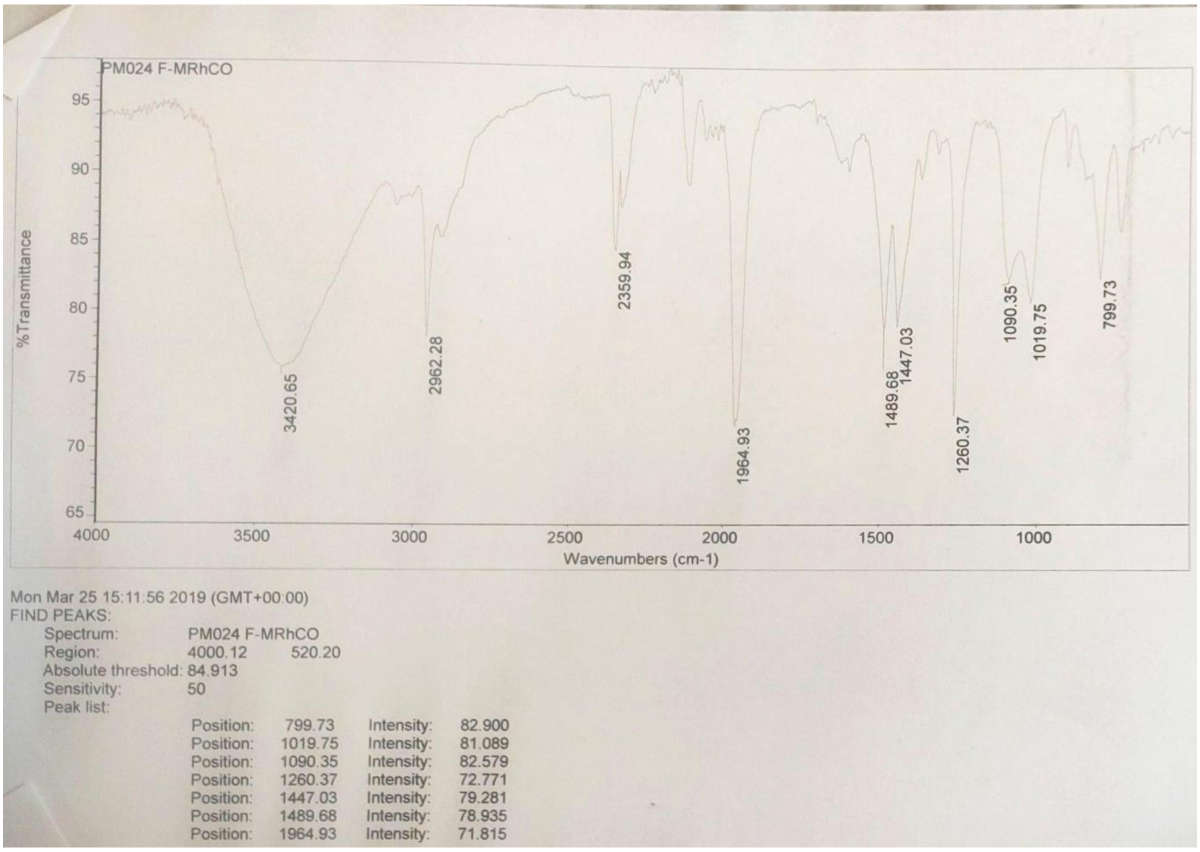

Figure S114. IR spectrum of [Rh(Flu-Mes)(CO)] (**15**).

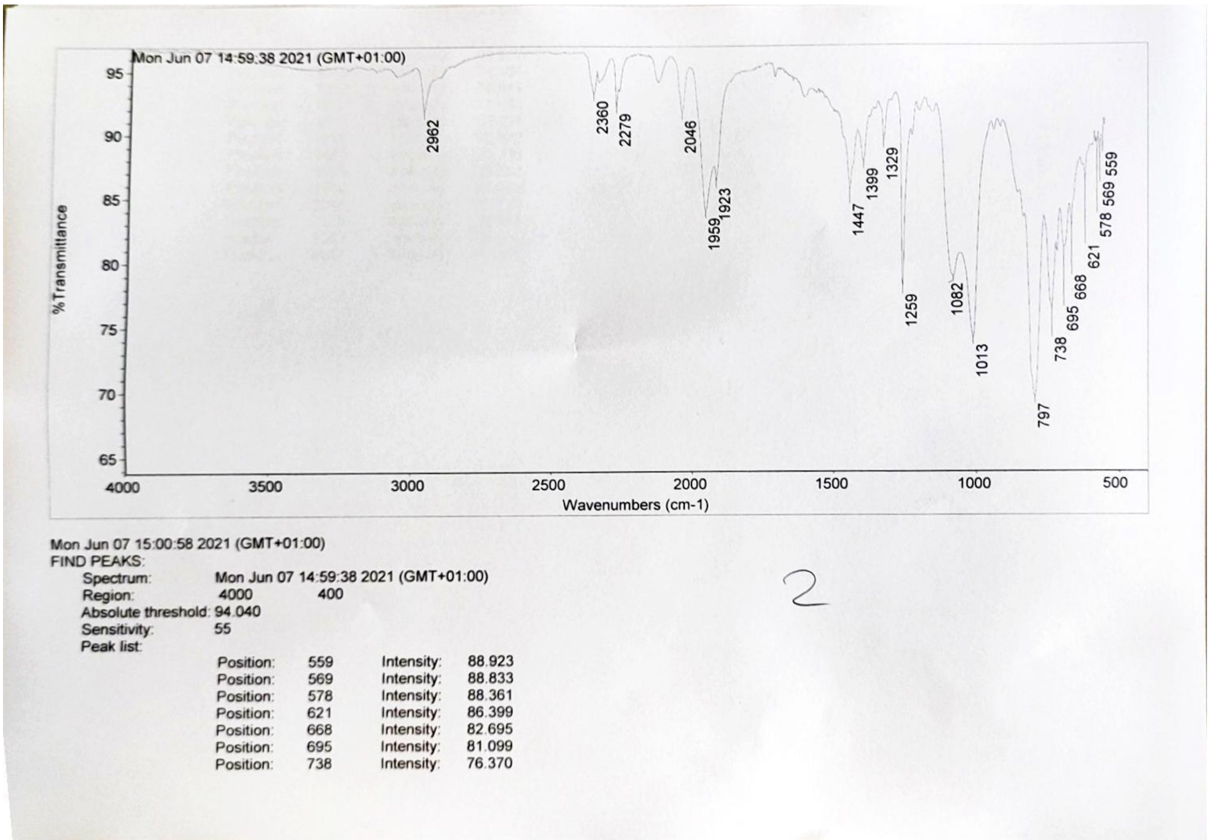

Figure S115. IR spectrum of [Rh(Flu-Me)(CO)] (**17**).

8 References

1. A. J. Arduengo, R. Krafczyk, R. Schmutzler, H. A. Craig, J. R. Goerlich, W. J. Marshall and M. Unverzagt, *Tetrahedron*, 1999, **55**, 14523.
2. M. S. Viciu, O. Navarro, R. F. Germaneau, R. A. Kelly, W. Sommer, N. Marion, E. D. Stevens, L. Cavallo and S. P. Nolan, *Organometallics*, 2004, **23**, 1629.
3. A. J. Arduengo, H. V. R. Dias, R. L. Harlow and M. Kline, *J. Am. Chem. Soc.*, 1992, **114**, 5530.
4. K. J. Evans, C. L. Campbell, M. F. Haddow, C. Luz, P. A. Morton and S. M. Mansell, *Eur. J. Inorg. Chem.*, 2019, **2019**, 4894.
5. K. J. Evans and S. M. Mansell, *Chem.-Eur. J.*, 2019, **25**, 3766.
6. R. Cramer, J. A. McCleverty and J. Bray, *Inorg. Synth.*, 1990, **28**, 86.
7. J. A. McCleverty, G. Wilkinson, L. G. Lipson, M. L. Maddox and H. D. Kaesz, *Inorg. Synth.*, 1990, **28**, 84.
8. C.-I. Lee, N. A. Hirscher, J. Zhou, N. Bhuvanesh and O. V. Ozerov, *Organometallics*, 2015, **34**, 3099.
9. P. Caddy, PhD, University of Bristol, 1977.
10. C. N. Garon, D. I. McIsaac, C. M. Vogels, A. Decken, I. D. Williams, C. Kleeberg, T. B. Marder and S. A. Westcott, *Dalton Trans.*, 2009, 1624.
11. K. Moseley, J. W. Kang and P. M. Maitlis, *J. Chem. Soc. A*, 1970, 2875.
12. J. S. Merola, R. T. Kacmarcik and D. Van Engen, *J. Am. Chem. Soc.*, 1986, **108**, 329.
13. T. Foo and R. G. Bergman, *Organometallics*, 1992, **11**, 1801.
14. T. Ishiyama, M. Murata and N. Miyaura, *J. Org. Chem.*, 1995, **60**, 7508.
15. W. Srimontree, L. Guo and M. Rueping, *Chem.-Eur. J.*, 2020, **26**, 423.
16. H. Kinuta, M. Tobisu and N. Chatani, *J. Am. Chem. Soc.*, 2015, **137**, 1593.
17. F. Mo, Y. Jiang, D. Qiu, Y. Zhang and J. Wang, *Angew. Chem., Int. Ed. Engl.*, 2010, **49**, 1846.
18. J.-Y. Cho, C. N. Iverson and M. R. Smith, *J. Am. Chem. Soc.*, 2000, **122**, 12868.
19. M. A. Larsen, C. V. Wilson and J. F. Hartwig, *J. Am. Chem. Soc.*, 2015, **137**, 8633.
20. C. B. Bheeter, A. D. Chowdhury, R. Adam, R. Jackstell and M. Beller, *Org. Biomol. Chem.*, 2015, **13**, 10336.
21. T. Ishiyama, J. Takagi, K. Ishida, N. Miyaura, N. R. Anastasi and J. F. Hartwig, *J. Am. Chem. Soc.*, 2002, **124**, 390.
22. J. M. Murphy, J. D. Lawrence, K. Kawamura, C. Incarvito and J. F. Hartwig, *J. Am. Chem. Soc.*, 2006, **128**, 13684.
23. M. Mlekuz, P. Bougeard, B. G. Sayer, M. J. McGlinchey, C. A. Rodger, M. R. Churchill, J. W. Ziller, S. K. Kang and T. A. Albright, *Organometallics*, 1986, **5**, 1656.
